# Investigating volatile compounds in the *Bacteroides* secretome

**DOI:** 10.3389/fmicb.2023.1164877

**Published:** 2023-05-03

**Authors:** Olga Yu Shagaleeva, Daria A. Kashatnikova, Dmitry A. Kardonsky, Dmitry N. Konanov, Boris A. Efimov, Dmitry V. Bagrov, Evgeniy G. Evtushenko, Andrei V. Chaplin, Artemiy S. Silantiev, Julia V. Filatova, Irina V. Kolesnikova, Anna A. Vanyushkina, Joanna Stimpson, Natalya B. Zakharzhevskaya

**Affiliations:** ^1^Laboratory of Molecular Pathophysiology, Lopukhin Federal Research and Clinical Center of Physical-Chemical Medicine of Federal Medical Biological Agency, Moscow, Russia; ^2^Laboratory of Mathematical Biology and Bioinformatics of Scientific Research Institute for Systems Biology and Medicine, Moscow, Russia; ^3^Department of Microbiology and Virology, Pirogov Russian National Research Medical University, Moscow, Russia; ^4^Department of Bioengineering, Faculty of Biology, Lomonosov Moscow State University, Moscow, Russia; ^5^Faculty of Chemistry, Lomonosov Moscow State University, Moscow, Russia; ^6^Vladimir Zelman Center for Neurobiology and Brain Rehabilitation, Skolkovo Institute of Science and Technology, Moscow, Russia; ^7^School of Health Sciences, Faculty of Biology, Medicine and Health, University of Manchester, Manchester, United Kingdom

**Keywords:** *Bacteroides* secretome, volatile compounds, HS-GC-MS, outer membrane vesicles (OMVs), bacterial media, nanoparticle tracking analysis (NTA)

## Abstract

Microorganisms and their hosts communicate with each other by secreting numerous components. This cross-kingdom cell-to-cell signaling involves proteins and small molecules, such as metabolites. These compounds can be secreted across the membrane via numerous transporters and may also be packaged in outer membrane vesicles (OMVs). Among the secreted components, volatile compounds (VOCs) are of particular interest, including butyrate and propionate, which have proven effects on intestinal, immune, and stem cells. Besides short fatty acids, other groups of volatile compounds can be either freely secreted or contained in OMVs. As vesicles might extend their activity far beyond the gastrointestinal tract, study of their cargo, including VOCs, is even more pertinent. This paper is devoted to the VOCs secretome of the *Bacteroides* genus. Although these bacteria are highly presented in the intestinal microbiota and are known to influence human physiology, their volatile secretome has been studied relatively poorly. The 16 most well-represented *Bacteroides* species were cultivated; their OMVs were isolated and characterized by NTA and TEM to determine particle morphology and their concentration. In order to analyze the VOCs secretome, we propose a headspace extraction with GC–MS analysis as a new tool for sample preparation and analysis of volatile compounds in culture media and isolated bacterial OMVs. A wide range of released VOCs, both previously characterized and newly described, have been revealed in media after cultivation. We identified more than 60 components of the volatile metabolome in bacterial media, including fatty acids, amino acids, and phenol derivatives, aldehydes and other components. We found active butyrate and indol producers among the analyzed *Bacteroides* species. For a number of Bacteroides species, OMVs have been isolated and characterized here for the first time as well as volatile compounds analysis in OMVs. We observed a completely different distribution of VOC in vesicles compared to the bacterial media for all analyzed *Bacteroides* species, including almost complete absence of fatty acids in vesicles. This article provides a comprehensive analysis of the VOCs secreted by *Bacteroides* species and explores new perspectives in the study of bacterial secretomes in relation the intercellular communication.

## Introduction

Intestinal bacteria actively produce nano-sized outer membrane vesicles (OMVs) as part of the bacterial secretory metabolome (Thaiss et al., 2016). Among various bacterial species, the *Bacteroidetes* phylum is highly represented, accounting for 10–20% of the bacterial population in the colon and thus contributing a high proportion to the total secretory activity (Ley et al., 2008). The enzymatic and immunomodulatory functions of some *Bacteroides* species are well known (Gibson and Macfarlane, 1988; Elhenawy et al., 2014). In particular, *Bacteroides fragilis* OMVs contain surface polysaccharide A (PSA), a microorganism-associated molecular pattern that is recognized by toll-like receptor 2 (TLR2) on Treg cells. The engagement of TLR2 and PSA leads to Treg cell induction and limits the TH17 response, thereby promoting tolerance and immune suppression in the gut (Telesford et al., 2015). Despite the ability of immune regulation by PSA, *B. fragilis* is also an opportunistic pathogen and the most commonly isolated anaerobe from human infections such as intra-abdominal and brain abscesses (Cheng et al., 2009; Fathi and Wu, 2016). Strong commensal relationships of *Bacteroides* and other bacterial species are well described. For instance, *B. thetaiotaomicron*, as a saccharolytic member, decomposes mucin, making it available to species within the microbiota that lack this capability. *B. thetaiotaomicron, B. caccae,* and *B. fragilis* exhibit their enzymatic properties via hydrolases contained in OMVs (Reeves et al., 1997; Shipman et al., 2000). In addition, metabolomic studies have made it possible to assert that vesicles are a kind of independent cellular prototypes in which metabolic reactions can occur without DNA replication and transcription (Spence et al., 2006; Zakharzhevskaya et al., 2017).

OMVs are extremely small structures, which allows them to function far beyond the intestine, transported through the bloodstream to target organs and presumably overcome the blood brain barrier in a bidirectional manner (Han et al., 2019). Thus, freely secreted components and vesicles can form the gut-brain axis actively studied today (Pirolli et al., 2021). OMVs investigation should be of the same priority as the structural and functional study of bacteria morphology and physiology. However, studies of individual species do not form a comprehensive view of the secretory activity of the genus as a whole. In addition, OMVs are largely unexplored in terms of metabolomic data. Metabolomes can be divided into volatile and nonvolatile fractions, which require different methods of sample preparation and analysis. (Kimball, 2016). While there is already data for the nonvolatile fraction, which includes amino acids, nucleotides, and sugars on their presence in OMV (Liebeke et al., 2012; Mielko et al., 2019); no such work has been carried out for vesicles’ volatile fraction.

It is known that short-chain fatty acids (SCFAs), being the main microbial volatile metabolites, mediate many regulatory effects. Acetic, propionic, and butyric acids are the most common ones, accounting for 90–95% of SCFAs in the colon (3:1:1 ratio) (Niccolai et al., 2019). Only a small fraction, about 5%, of SCFAs produced by intestinal epithelium can be found in feces. Butyrate is rapidly used as an energy source for colonocytes, while most of the acetate and propionate enter the portal circulation (Louis and Flint, 2017). Butyrate is a histone deacetylase inhibitor with potential effects on gene expression in human cells (Davie, 2003). Propionate crosses the blood–brain barrier (Wenzel et al., 2020) and, like butyrate, affects various physiological processes such as cell signaling, neurotransmitter synthesis and release, free radical production, and mitochondrial function. Propionate is also the preferred liver precursor for the regulation of cholesterol synthesis and gluconeogenesis (Kennedy et al., 2020). Acetate is the main SCFA in the blood and plays a key metabolic role in peripheral tissues, acting as a substrate for lipogenesis (den Besten et al., 2015). Volatile spectrum of metabolites is also represented by medium and long chain fatty acids, amino acid and phenol derivatives, etc. but most of the components have not yet been studied for biological effects (Rondanelli et al., 2019).

Gas chromatography combined with mass spectrometry (GC–MS) is usually used to analyze the volatile metabolome (Filipiak et al., 2022). Composition of SCFAs in stool samples are well described but most of the published data were obtained by the solid phase microextraction method (SPME) (Marrubini et al., 2020). The SPME method discriminates samples by composition due to the fact that different substances that make up the equilibrium vapor are absorbed differently. After desorption, the composition and ratio of the metabolome components are different from the initial ratio and composition (Karu et al., 2018). Conversely, the vapor-phase extraction method makes it possible to compare various biological samples, including feces, since the ratios of components in an equilibrium vapor do not depend on the amount of water contained in the samples. The main problem of HS-GC/MS method is the complex, non-linear dependence between the composition of the compounds in the source phase and the relative pressures in the vapor phase, which are directly measured by this method. Thermodynamic model that performs the reverse transformation of relative abundances in the vapor phase to relative concentrations in the liquid phase have been previously described by Konanov et al. (2022).

However, the described methods are widely used for stool analysis (Dixon et al., 2011). Here, we focused on the use of headspace extraction methods combined with GC–MS for the most represented *Bacteroides* species volatile secretome analysis. Using the available literature data, the following *Bacteroides* species/strains were selected: *Bacteroides vulgatus* EBA 3–9; *Bacteroides caccae* EBA 6–24; *Bacteroides dorei* EBA 7–24; *Bacteroides uniformis* EBA 5–20; *Bacteroides xylanisolvens* EBA 5–17; *Bacteroides coprocola* EBA 6–21; *Bacteroides finegoldii* EBA 6–28; *Bacteroides clarus* 606; *Bacteroides cellulosilyticus* 807; *Bacteroides salyersiae* 2697; *Bacteroides xylanisolvens* Pik; *Bacteroides thetaiotaomicron* 6237; *Bacteroides eggerthii* 91; *Bacteroides stercoris* 5888; *Bacteroides intestinalis* 181; *Bacteroides plebeius* 2436; *Bacteroides fragilis* BOB25; *Bacteroides fragilis* JIM10 (Kulagina et al., 2012; Tyakht et al., 2013).

## Results

### Isolation and morphological evaluation of *Bacteroides* OMVs

All analyzed *Bacteroides* species were isolated from stool samples of healthy volunteers and identified by 16S rRNA gene sequencing analysis. Sequenced data and links to sequenced genomes provided in Supplementary Table 1. It is known that the cultivation of anaerobes is not an easy task, however for *Bacteroides*, with the use of gas-generating packages and anaerobic jars, it was possible to cultivate all species. Cultivation on a liquid medium (Columbia Broth Base, HIMEDIA, India) was suitable for all species, except for *Bacteroides plebeius* 2436. For growing *Bacteroides plebeius* 2436, special base liquid medium was used (see methods). 48 h of continuous cultivation on a liquid media was recognized as the optimal duration for cultures to be ready for OMV isolation. The exceptions were for strains such as *Bacteroides plebeius* 2436; *Bacteroides clarus* 606; *Bacteroides dorei* EBA 7–24; *Bacteroides coprocola* EBA 6–21, the optimal cultivation time of which was more than two and a half days (60 h).

Isolation of OMVs was carried out according to the standard method using filtration and ultracentrifugation. The prepared samples were examined by NTA (nanoparticle tracking analysis) and TEM (transmission electron microscopy) methods.

**Figure 1:**
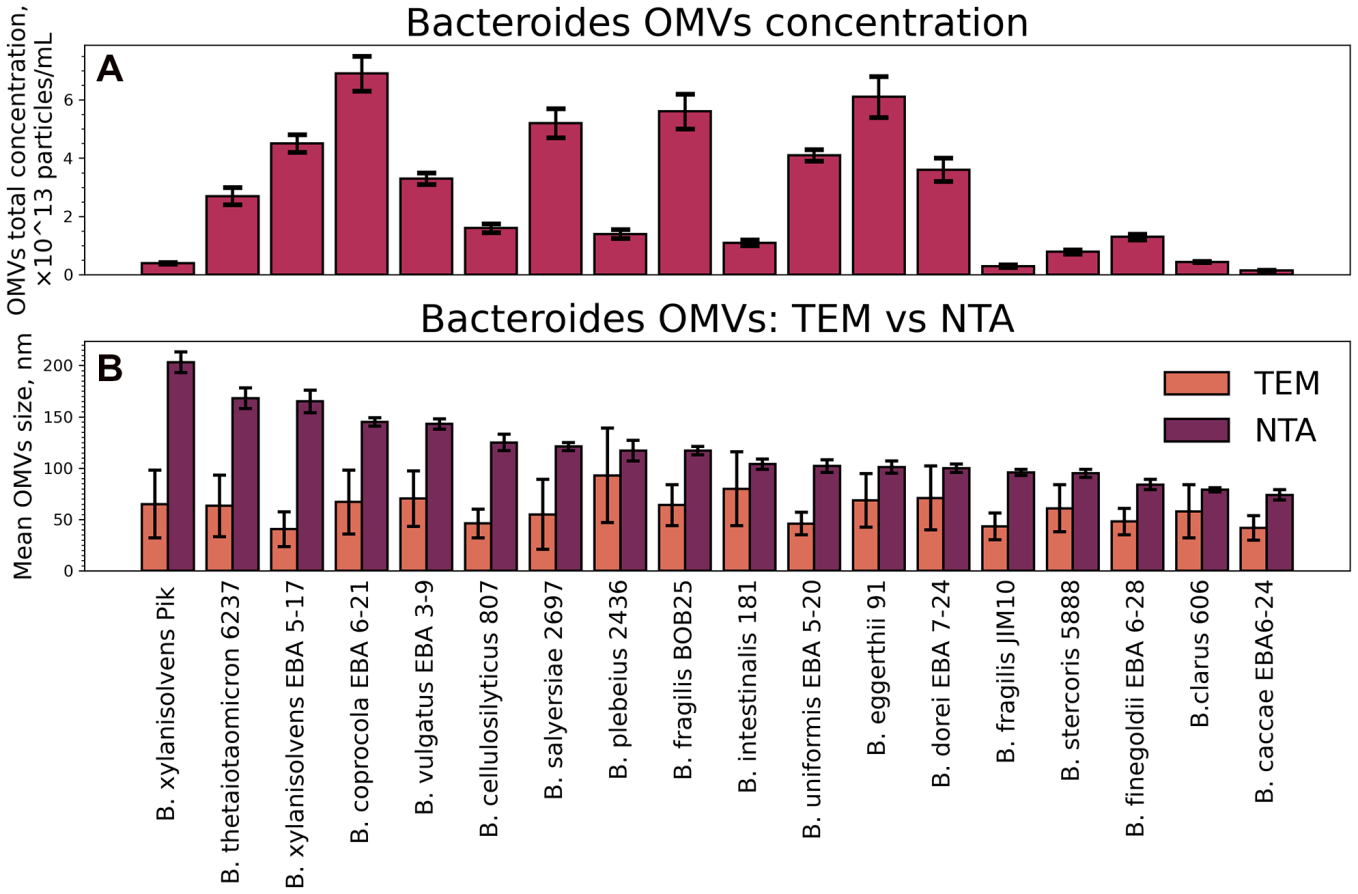
Total particle concentrations and mean sizes of isolated OMVs. **(A)** The NTA data on total particle concentrations of isolated OMVs. Error bars represent 95% confidence intervals calculated from *N* = 14–21 repeated measurements of each sample. **(B)** Mean sizes of isolated OMVs measured by TEM and NTA. Error bars for NTA represent 95% confidence intervals calculated the same way as for the total concentration. Error bars for TEM represent standard deviation as a measure of particle size distribution width.

To estimate the relative productivity of different *Bacteroides* species, concentration data from NTA measurements were used Supplementary Table 2 S1. Vesicles were isolated from 200 mL of bacterial media at the end of the logarithmic growth phase at the same optical density of bacterial media. The resulting particle concentration pattern is shown in Figure 1A. The lowest OMVs production was measured for *Bacteroides caccae* EBA 6–24, the largest number of particles has been counted for *Bacteroides coprocola* EBA 6–21.

**Figure 2:**
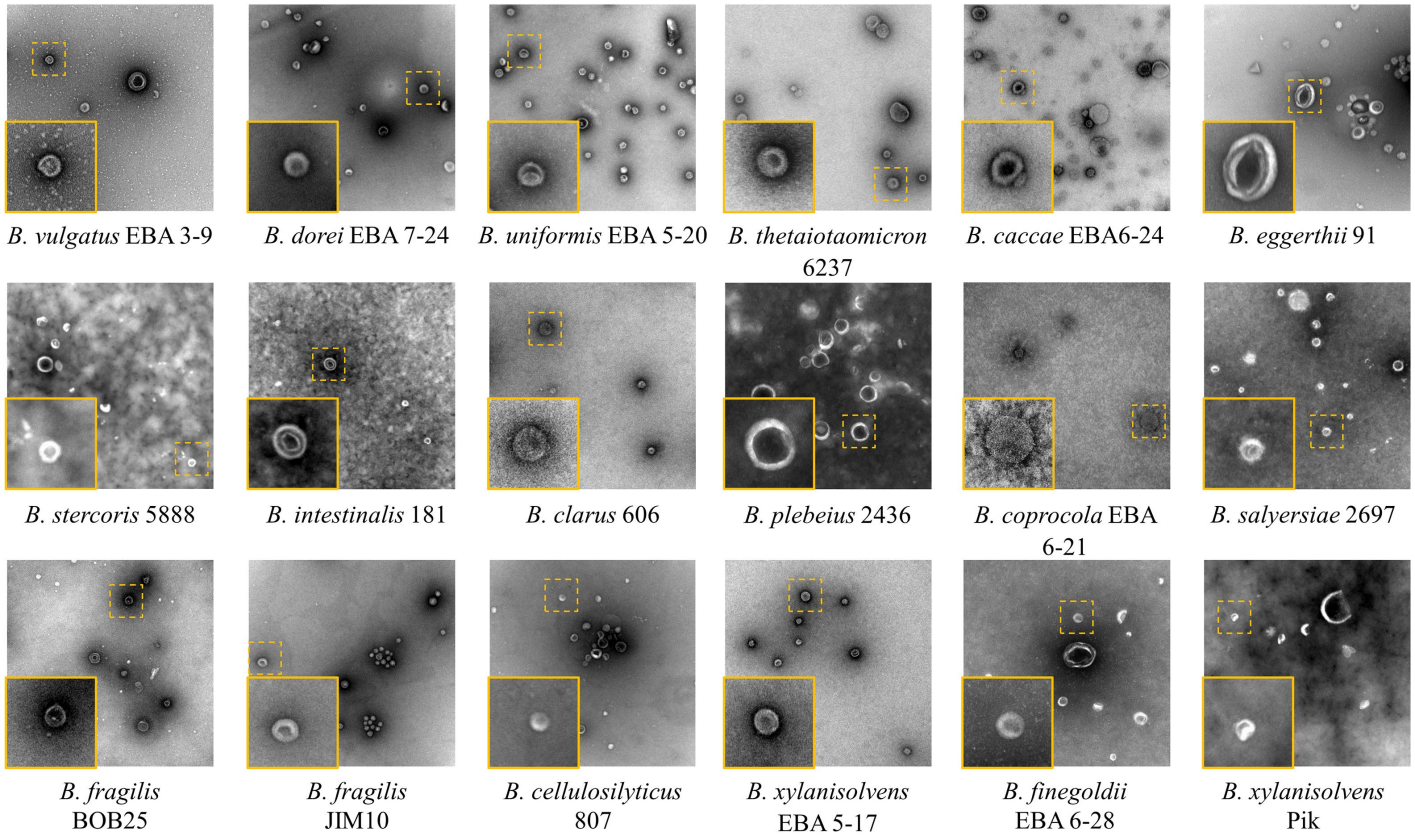
TEM images of the isolated *Bacteroides* species OMVs. The insets show individual vesicles magnified. Image size 1,260 × 1,260 nm^2^, inset size 200 × 200 nm^2^.

All the OMV samples were characterized using TEM (Figure 2). Individual OMVs were roughly spherical, and most particles had a relatively dark part at the center. This morphology is typical not only for bacterial OMVs but also for the small extracellular vesicles isolated from eukaryotic cells (Nikishin et al., 2021).

When comparing the sizes of OMVs, determined by the TEM and NTA, substantial differences between two techniques have been revealed (Figure 1B). For instance, OMVs from *B. fragilis* BOB25 have shown the mean hydrodynamic particle size of 117 nm, while TEM showed a mean diameter of 60 nm only. The common reason for such a discrepancy is a heavy aggregation of vesicles, but it was not supported by TEM. We propose that these differences might be caused by the presence of long polysaccharide chains on the vesicles’ surface. They contribute to the hydrodynamic size, but might be not visualized with TEM due to poor staining of polysaccharides with uranyl acetate.

### Gas chromatographic analysis of bacterial media

**Figure 3:**
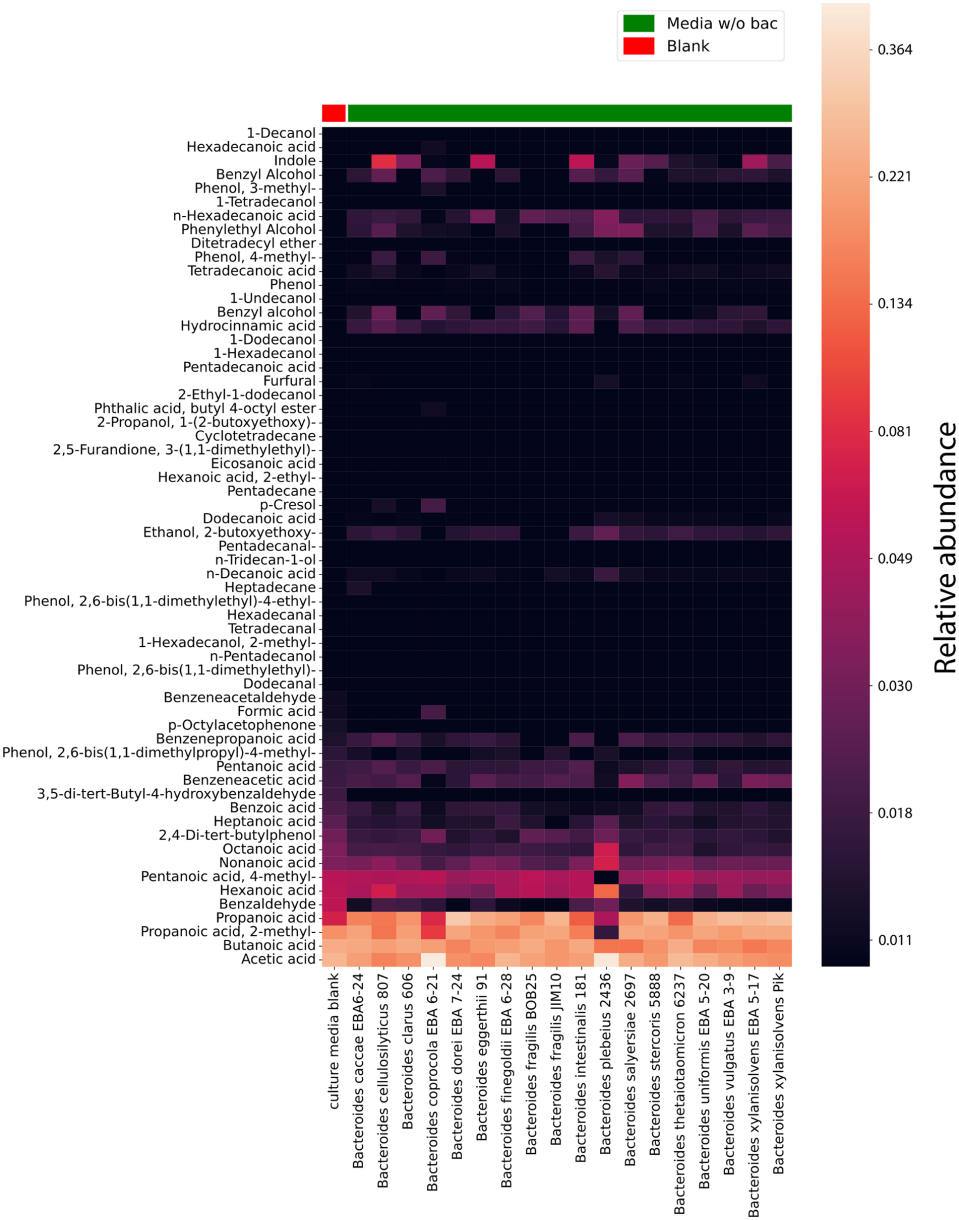
HS-GC/MS data for *Bacteroides* species media, cleared from bacteria (media without (w/o) bacteria). VOCs composition in cultivating media cleared from bacteria (after cultivation). The first column represents the VOCs composition in a sterile media before cultivation used as a control. The relative concentrations in the vapor phase are used.

At the first stage of the HS-GC/MS study of the volatile spectrum of *Bacteroides* secretome, the compositions of autoclaved media (culture media) and media after bacterial cultivation, cleared from bacteria by filtration (media w/o bac) Supplementary Table 2 S2, S3 were determined. The sample preparation for each media varied. In one case, the entire secreted volatile metabolome was of interest. Then, after cultivation, the bacterial media was centrifuged at 4,500*g* at 4°C for half an hour and the supernatant was additionally filtered using a 450 nm filter. Next, 200 μL of the bacterial media, which was cleaned from bacterial cells was used to extract volatile metabolites, followed by analysis and identification by HS-GC/MS. In the other case (media w/o bac), we obtained information from all freely secreted volatile compounds contained in the bacterial media at the end of the logarithmic phase as shown in Figure 3. However, media without OMVs were of particular interest. To obtain these preparations, bacterial media after cultivation was centrifuged at 4,500*g* at 4°C for half an hour and the supernatant was additionally filtered using a 450 nm filter. Next, the resulting filtrate was centrifuged at 100,000*g* at 4°C for 2 h. The obtained supernatant was filtered using a 220 nm filter. 200 μL of the resulting supernatant was used to extract volatile metabolites, followed by analysis and identification by HS-GC/MS. When analyzing the samples media w/o OMVs we could evaluate the contribution of OMVs to the total volatile secretory activity of bacteria.

According to the obtained data, 61 compounds were stably detected in media w/o bac, 20 of which could be detected in culture media Supplementary Table 2 S3. However, it should be noted that for the components identified in the culture media, a significant quantitative increase was observed during cultivation for most components.

Short-chain fatty acids and their derivatives, including propanoic, 2-methyl-propanoic, acetic, and butanoic acids, stand out among the entire spectrum of detectable components in the bacterial media after cultivation (media w/o bac). It was noted that if butanoic acid is produced by all analyzed species in approximately equal relative quantities, then acetic acid is produced in a larger relative amount by *Bacteroides plebeius* 2436 and *Bacteroides coprocola* EBA 6–21. Indole is produced to a lesser extent than SCFA by all studied bacteria. However, it can be noted that *Bacteroides vulgatus* EBA 3–9, *Bacteroides fragilis* BOB25, *Bacteroides fragilis* Jim10, *Bacteroides finegoldii* EBA 6–28, *Bacteroides dorei* EBA 7–24 and *Bacteroides caccae* EBA 6–24 produce relatively lower amounts of indole compared to other bacteria. Also, among the detected volatile compounds were medium and long chain fatty acids, including hexanoic, octanoic, heptanoic, hydrocinnamic, pentanoic, n-hexadecanoic, n-decanoic, and tetradecanoic acid have been identified.

Interestingly, among the detected compounds 2,4-di-tert-butylphenol has been identified. 2,4-di-tert-butylphenol or 2,4-bis-(1,1-dimethylethyl)-phenol (2,4-DTBP) is a common natural compound, showing strong toxicity against almost all tested organisms, including against its producers. The secretion of 2,4-DTBP for *Bacteroides* has not been previously described. At the same time, in a relatively small amount, most of all the studied *Bacteroides* species produce 2,4-DTBP with the exception of *Bacteroides xylanisolvens* PIK, *Bacteroides finegoldii* EBA 6–28, *Bacteroides dorei* EBA 7–24 and *Bacteroides cellulosilyticus* 807 (Zhao et al., 2020).

Hydrocinnamic acid (Phenylpropanoic acid) or hydroxycinnamic acid is a carboxylic acid belonging to the class of phenylpropanoid, previously regarded as an antimicrobial agent. As can be seen from the obtained data, it is also revealed to be among the main components of the bacterial media after cultivation. It is noteworthy that, similar to 2,4-di-tert-butylphenol, hydroxycinnamic acid should exhibit autotoxic activity against the producer, but *Bacteroides* apparently find a way to neutralize the toxic effect of the hydrocinnamic acid (Coman and Vodnar, 2020).

**Figure 4:**
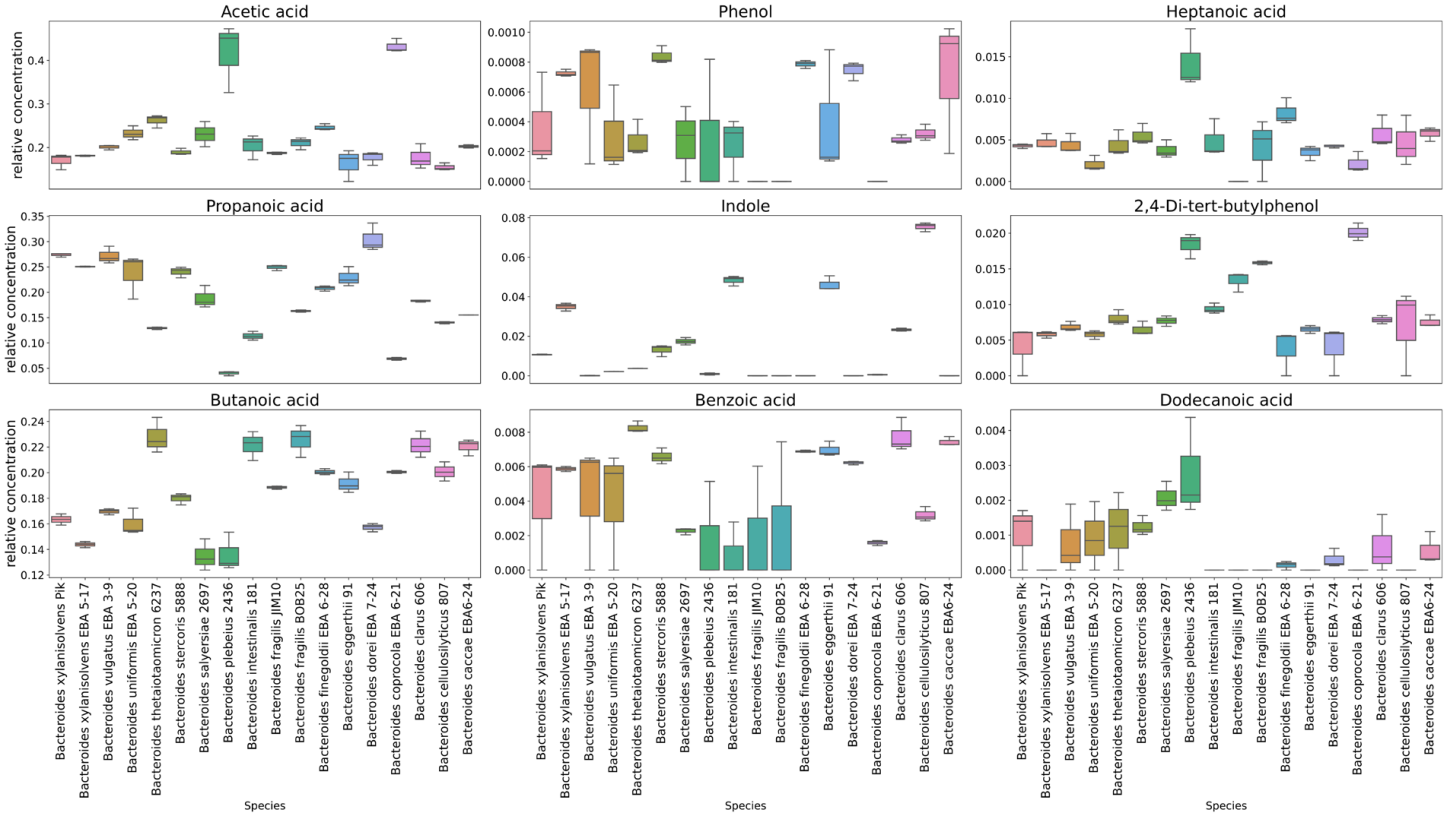
Representation of individual components quantitative differences in media cleared from bacteria. Box-plots show quantitative differences in the relative content for the most known volatile compounds secreted to the media, including short chain fatty acids, indole, phenol, as well as some medium chain fatty acids. Box plots generated in triplicates.

In a comparative analysis, it is also possible to see the relative content of the individual components in *Bacteroides* species media Figure 4 and Supplementary Data Sheet 1. A relatively higher amount of propionate was revealed in media for *Bacteroides dorei* EBA 7–24 than for other species. Despite the fact that all *Bacteroides* species were found to produce butyrate, in the cultivation bacterial media butyrate has been found for *Bacteroides thetaiotaomicron* 6237, *Bacteroides intestinalis* 181, *Bacteroides fragilis* BOB25, *Bacteroides caccae* EBA 6–24 and *Bacteroides clarus* 606 in a larger relative amount. A relatively higher quantitative amount of indole was found in the media of *Bacteroides cellulosilyticus* 807, *Bacteroides eggerthii* 91, *Bacteroides intestinalis* 181 and *Bacteroides xylanisolvens* EBA 5–17.

In a comparative analysis of bacterial media cleared from OMVs (media w/o OMV) Supplementary Table 2 S4 with media containing OMVs (media w/o bac) Supplementary Table 2 S3, interesting changes in the relative quantitative of the components were noted.

Relative quantitative change in some components content was noted for almost all *Bacteroides* species Supplementary Image 1. We observed the content reduction of medium and long chain acids in media w/o OMVs for *Bacteroides fragilis* BOB25, *Bacteroides clarus* 606, *Bacteroides vulgatus* EBA 3–9. It has been shown that quantitative changes affected more than three types of components in other *Bacteroides* species. Thus, *Bacteroides plebeius* 2436, *Bacteroides dorei* EBA 7–24 and *Bacteroides xylanisolvens* EBA 5–17 showed a greater relative quantitative change than other species in samples cleared from vesicles (media w/o OMVs). A decrease in the relative content of short-chain, medium-and long-chain acids and their derivatives, as well as indole, was noted in the bacterial media without OMVs. According to the obtained media analysis data, it can be indirectly concluded that indole is contained in vesicles of at least five analyzed species, including *Bacteroides cellulosilyticus* 807, *Bacteroides salyersiae* 2697, *Bacteroides xylanisolvens* Pik, *Bacteroides eggerthii* 91 and *Bacteroides xylanisolvens* EBA 5–17. At the same time, the exact composition of the components can only be judged by analyzing isolated OMVs. However, rather contradictory changes were noted, in particular the accumulation of components in the bacterial media cleared of vesicles (media w/o OMVs). Thus, for 2,4-di-tert-butylphenol, such a dependence was noted for most of the studied species, which is probably due to the peculiarities of the component extraction and sample preparation.

### HS-GC/MS analysis of OMVs

**Figure 5:**
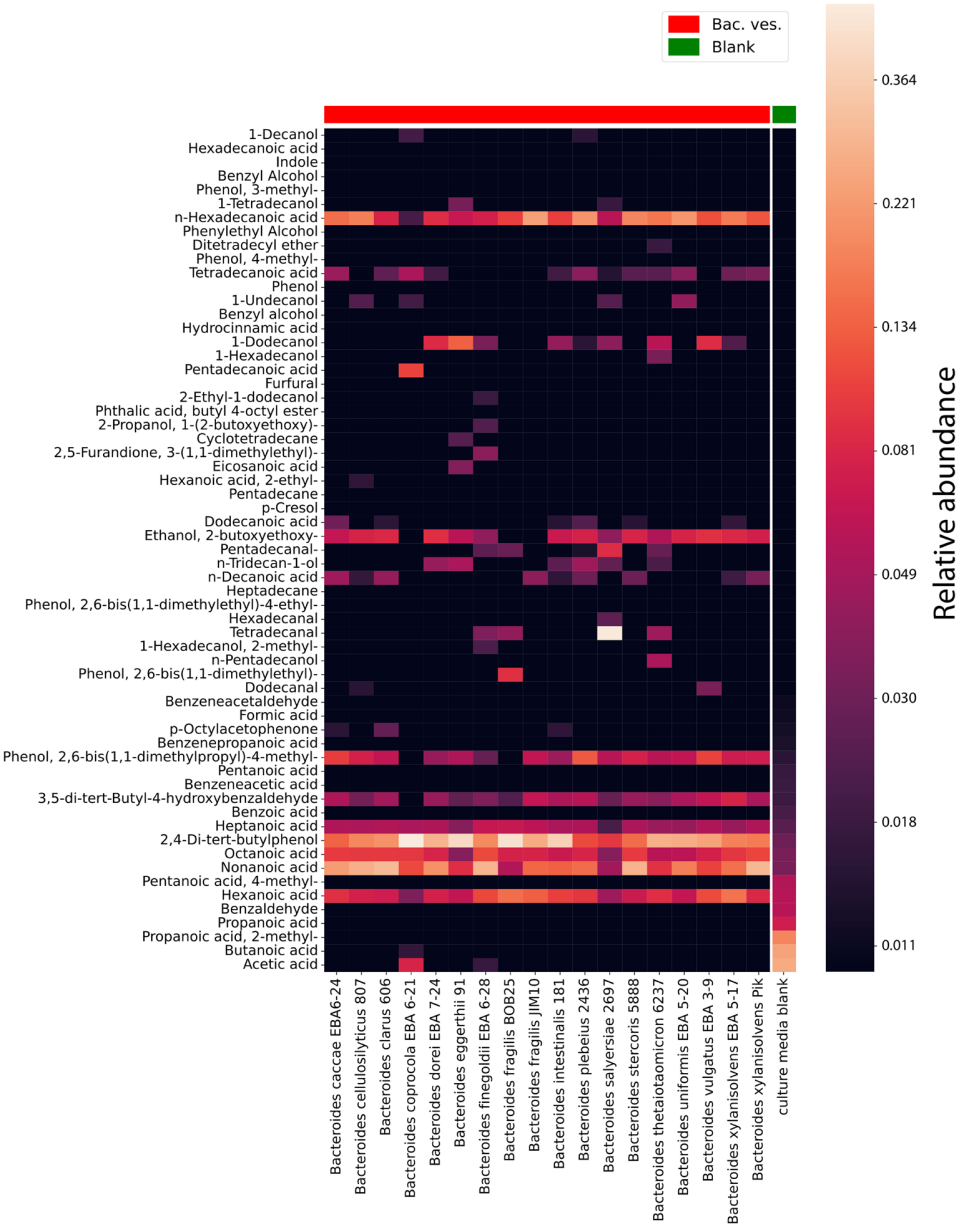
HS-GC/MS data for *Bacteroides* species OMVs. VOCs composition in isolated OMVs. The last column represents the VOCs composition in a sterile media before cultivation used as a control. The relative concentrations in the vapor phase are used.

To obtain OMVs for the volatile spectrum analysis we used ultracentrifugation and filtration methods. The OMVs containing samples were prepared from 200 mL of culture bacterial media purified from bacteria. According to the obtained data, vesicles of almost all analyzed strains contained 1-dodecanol, medium and long chain acids, including: heptanoic acid, n-hexadecanoic acid, octanoic acid, nonanoic acid, hexanoic acid Figure 5 and Supplementary Table 2 S5.

According to the obtained HS-GС/MS data for OMVs, vesicles were different in their component composition. 1-Hexadecanol was only found in vesicles in *Bacteroides thetaiotaomicron* 6237. Cyclotetradecane has been identified in the vesicles of *Bacteroides eggerthii* 91. The greatest differences in the OMVs composition were registered for *Bacteroides finegoldii* EBA 6–28. Among the identified metabolites, 2-methyl-1-hexadecanol, 3-(1,1-dimethylethyl)-2,5-furandione, 2-ethyl-1-dodecanol, 1-(2-butoxyethoxy)-2-propanol were detected.

**Figure 6:**
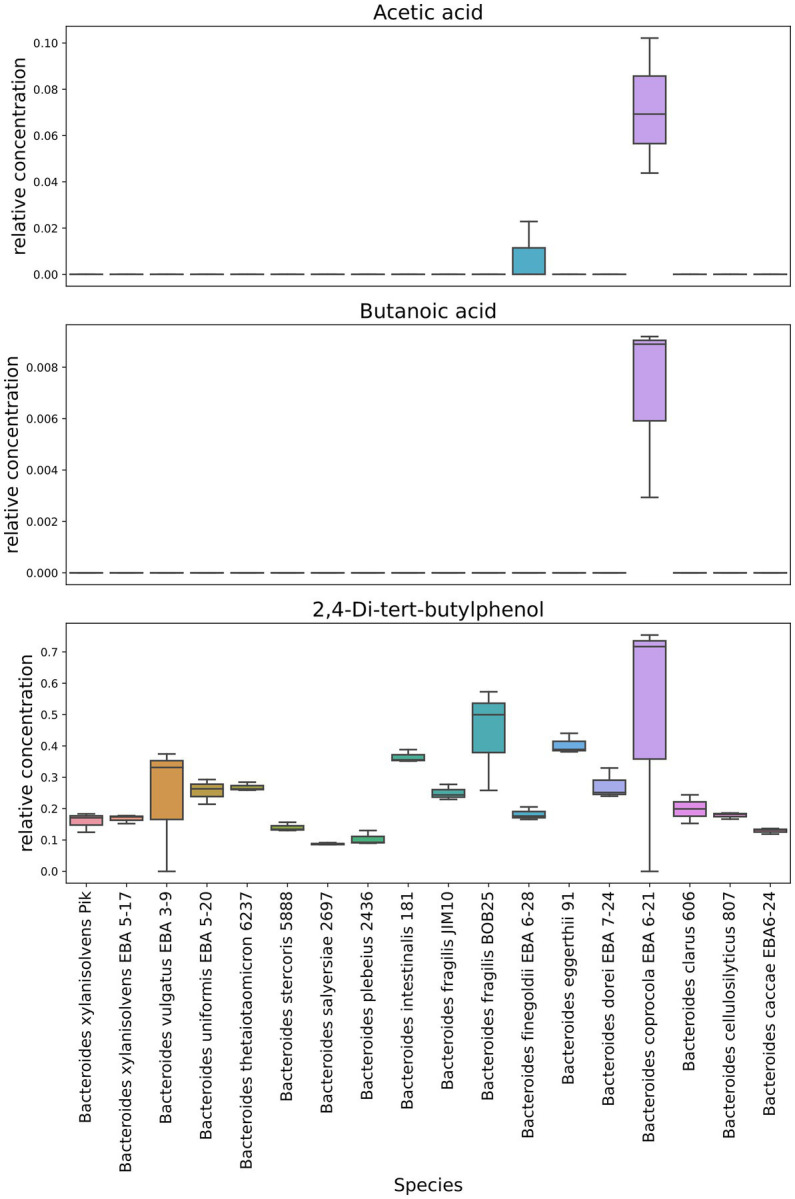
Representation of individual components quantitative differences in *Bacteroides* OMVs. Box-plots show quantitative differences in the relative content of short chain fatty acids, detected in *Bacteroides coprocola* and *Bacteroides finegoldii*. It also shows the distribution of the quantitative content of 2,4-Di-tert-butylphenol in *Bacteroides* OMVs. Box plots generated in triplicates.

Interestingly, 2,4-Di-tert-butylphenol has been identified in virtually all *Bacteroides* species. At the same time, this metabolite was largely detected in *Bacteroides fragilis* BOB25 and *Bacteroides coprocola* EBA 6–21 Figure 6. A detailed analysis of individual components of all analyzed species is presented in Supplementary Data Sheet 2.

In contrast to previously described HS-GC/MS data of culture media, no short-chain fatty acids were found in OMVs. We observed only small amounts of acetate and butyrate in *Bacteroides coprocola* EBA 6–21 and *Bacteroides finegoldii* EBA 6–28.

3,5-di-tert-butyl-4-hydroxybenzaldehyde was detected in a much larger relative amount in all vesicles. In a total comparison of the media component composition, we can see the almost complete absence or the minimum relative content of 3,5-di-tert-butyl-4-hydroxybenzaldehyde both in media purified from bacteria and in media purified from vesicles. The possibility of detection of this component was apparently obtained by way of concentrating the OMVs sample.

When analyzing the data obtained in Supplementary Image 2, it is important to evaluate the compositional ratio of metabolites, but not their exact amount in the analyzed samples. Obviously, vesicles differ to a large extent from media, which can be associated both with the specific origin of the analyzed metabolite and with the features of sample preparation.

**Figure 7:**
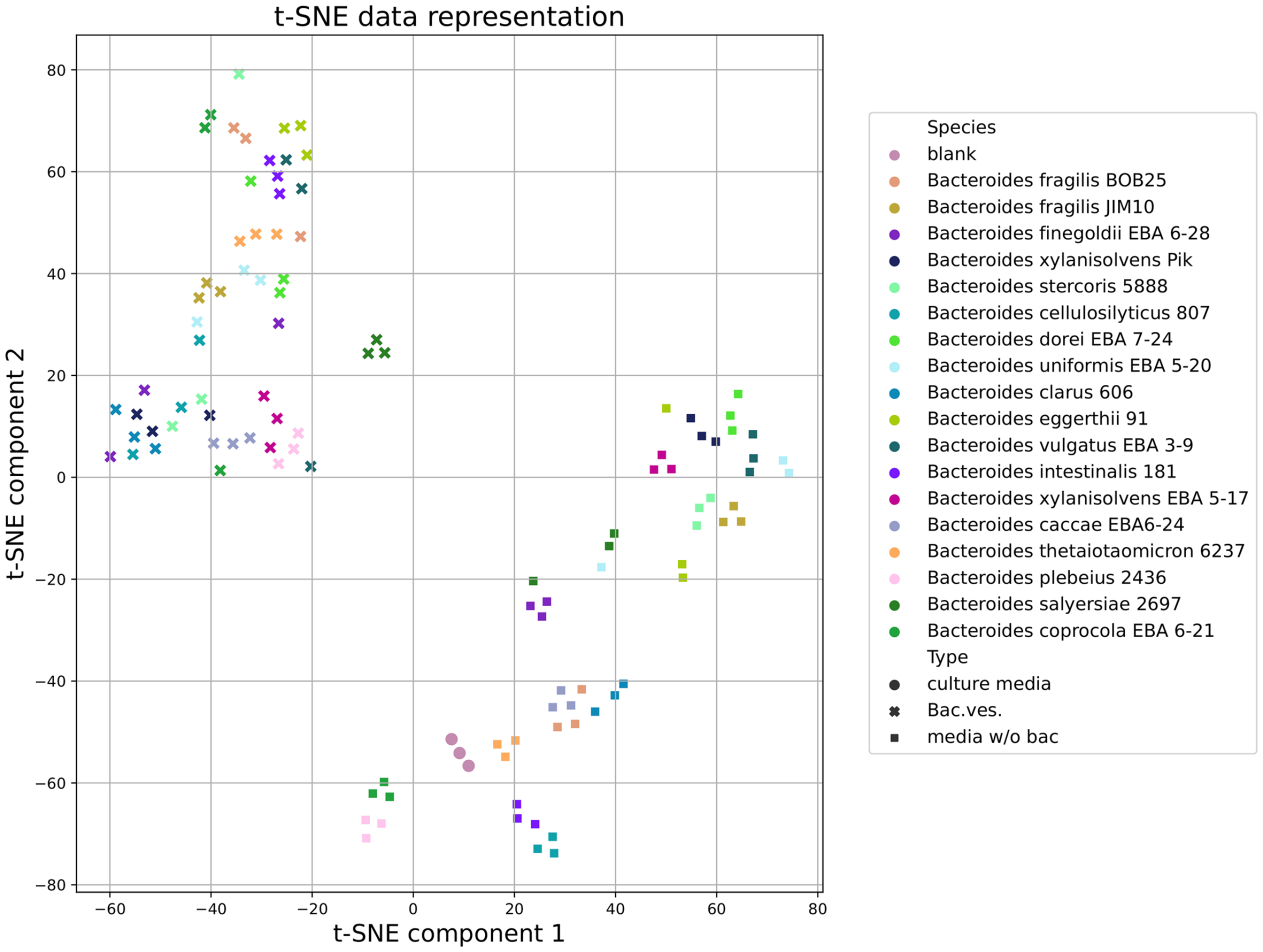
Total HS-GC/MS data for *Bacteroides* species. t-SNE (t-distributed Stochastic Neighbor Embedding) data demonstrates the clustering of samples (media w/o bacteria, OMVs and sterile media before cultivation used as a control) according to the degree of its similarity.

Both in the Heatmap and results obtained by the t-SNE method (t-distributed Stochastic Neighbor Embedding), Figure 7 shows that vesicles component composition significantly differs from the bacterial media. Analyzing the TSNE data, we got the opportunity to refine the degree of species clustering by the metabolome volatile composition. In particular, it can be seen that

- *Bacteroides vulgatus* EBA 3–9 and *Bacteroides dorei* EBA 7–24;
- *B. plebeuis* 2436 and *B. coprocola* EBA 6–21;
- *B. eggerthii* 91 and *B.stercoris* 5888 and *B. clarus* 606,

which are genetically close to each other (Hahnke et al., 2016), are localized in close proximity, which indicates a high degree of similarity between the data obtained for these strains’ combinations Figure 7.

## Discussion

A comprehensive study of the morphological and functional properties of the gastrointestinal microbiota is largely determined by the variety of methodological approaches used for sample analysis. Sequencing methods, proteomic and transcriptomic studies are actively used in research. Metabolic methods are rarely used, especially for secretome and vesicles analysis and such approaches as gas–liquid chromatography analysis of volatile spectrum practically are not presented in the literature. Identification of the biological properties of short-chain fatty acids has opened up prospects for assessing the regulatory mechanisms of the bacterial influence on the intestinal epithelial cells. Today there is a toward the practical application of the accumulated knowledge on bacterial communications. Creating complex probiotics underlies maintenance therapy, and in some cases, potential immunomodulatory therapy for inflammatory bowel diseases. In this regard, the study of *Bacteroides* species can contribute not only to the accumulation of fundamental knowledge about the functional activity of the widely represented genus in the gut microbiota, but also to the acquisition of essential new information for new age probiotic creation.

In this research, we studied the volatile spectrum of the secretory metabolome of the most represented species of the genus *Bacteroides*. For the first time bacterial vesicles were examined by headspace extraction methods. According to the data obtained, the headspace extraction method made it possible to identify more than 60 components of the volatile metabolome in the bacterial media. In the course of our research, we evaluated the fatty acids exposed to the bacterial media, as well as various amino acids and phenol derivatives, aldehydes and other components. Since the bacterial secretome is also represented by OMVs, we separately assessed media purified from vesicles and OMVs concentrated by ultracentrifugation. For a correct assessment of the relative amounts of the media components, we analyzed volatile compounds of the sterile culture bacterial media. One of the most important tasks of this research was the characterization of short-chain fatty acids released into the culture bacterial media. In most studies, when analyzing stool samples by gas–liquid chromatography, a considerable emphasis is placed on the quantitative content of butyrate and propionate. Therefore, the assessment of these two components in the most represented *Bacteroides* species was of our priority interest, especially since such a characterization had not previously been carried out for most of the species analyzed in this research. Thus, we have shown that all bacterial species actively produce butyrate, while *B. thetaiotaomicron* 6237, *B. intestinalis* 181, *B. fragilis* BOB25, *B. caccae* EBA 6–24 and *B. clarus* 606 produce relatively more butyrate. In the absence of pathogenic properties, these microorganisms can be used to create a probiotic due to the active production of butyrate, which cannot be used for the toxigenic strain of *B. fragilis*. Remarkably, the non-toxigenic strain of *Bacteroides fragilis* JIM10 produces relatively less butyrate, although the genetic homology of both strains is considerable (Zakharzhevskaya et al., 2017). It was not possible to identify the most active producers of propionate, as well as for acetate, despite the fact that the latter is produced in relatively large quantities by bacteria such as *B. plebeius* 2436 and *B. coprocola* EBA 6–21. While *B. thetaiotaomicron*, *B. fragilis* BOB25, *B. fragilis* JIM10 and *B. intestinalis* have been extensively studied, the importance of *B. plebeius* in maintaining gastrointestinal homeostasis has not been previously assessed. Today it is known that *B. plebeius* can enhance the barrier function of the intestinal mucosa and modify the intestinal microbiome (Pei et al., 2022). Indole also has a significant number of reported biological properties beneficial to the human. As a derivative of tryptophan, indole is used by bacteria as a signaling molecule. In addition, indole helps maintain the integrity of the intestinal barrier (Lee and Lee, 2010). In our study, we detected the indole production by the most of the analyzed bacterial species. In *B. cellulosilyticus* 807, *B. eggerthii* 91, *B. intestinalis* 181 and *B. xylanisolvens* EBA 5–17 large amount of indole were detected.

An interesting find was the discovery of 2,4-di-tert-butylphenol among the secreted volatile components. 2,4-di-tert-butylphenol or 2,4-bis-(1,1-dimethylethyl)-phenol (2,4-DTBP) is a common toxic secondary metabolite produced by various groups of organisms. The biosources and bioactivity of 2,4-DTBP are well studied; however, the question arises as to whether it is reasonable to accumulate 2,4-DTBP by producers, taking into account its toxic effects. However, there is an opinion that endocytic regulation seems to be the main function of phenols in producing organisms (Zhao et al., 2020). Some studies have also noted the antioxidant activity of the class of lipophilic phenols, with the focus of research shifted toward the analysis of the antioxidant properties of 2,4-DTBP in human plasma (Yoon et al., 2006). It was also found that the administration of LPS in a mouse macrophage cell line model significantly increased the expression of the interleukin IL-6 and IL-1b tumor necrosis factor alpha (TNF-α) genes, while the administration of 2,4-DTBP reduced the expression of all three genes (Nair et al., 2020). Another unique property of 2,4-DTBP is its antiviral activity against herpesvirus type 2 (HSV-2) (Leila et al., 2019). Such an abundance of unique properties of 2,4-DTBP confirms the need for a detailed study of the biological properties of all components found in the bacterial metabolome.

Hydroxycinnamic acid (HCAs), found in the volatile metabolome, are the main class of phenolic acids with a characteristic C6-C3 phenylpropanoid chain. Hydroxycinnamic acids are typically conjugated with plant cell wall components (Leonard et al., 2021). *In vitro*, *in vivo*, and epidemiological studies cover the considerable biological activities of hydroxycinnamic acids such as antioxidant, antimicrobial, anti-inflammatory and risk-reducing effects on a number of human diseases, including cardiovascular disease, diabetes, depression, hypertension, and colorectal cancer (Zhao et al., 2020). Similar to 2,4-di-tert-butylphenol, hydroxycinnamic acid can exhibit auto toxic activity against the producer. For this reason, apparently, *Bacteroides* also have protection mechanisms of the hydroxycinnamic acids toxic effects.

In this research, for the first time, we have evaluated the volatile spectrum of the bacterial vesicle’s metabolome. We described volatile vesicles composition in two ways. Taking into account that the samples containing vesicles can affect the composition of the volatile spectrum of the metabolome, we separately studied the bacterial media purified from vesicles in comparison with the bacterial media containing them, and accordingly determined the composition by subtracting the components from the richer bacterial media. In addition, we conducted the first study of the OMVs volatile metabolome of all analyzed *Bacteroides* species. When comparing the media with and without vesicles, we found a change, mainly toward a decrease in the relative content of the components in the samples purified from vesicles. For *B. plebeius* 2436, *B. dorei* EBA 7–24 and *B. xylanisolvens* EBA 5–17, the decrease in the relative content of the components was more noticeable. And again, the emphasis was placed in favor of a preferential assessment of the potential content of SCFAs in vesicles. As expected, we observed a decrease in the relative amount of short-chain, medium-and long-chain acids and their derivatives in media purified from OMVs. However, we have not identified SCFAs in vesicles. We concluded that SCFAs are presented in vesicles, but sample preparation, which included several procedures of ultracentrifugation and washing can affect the ratio of SCFAs in OMVs. At the same time, we cannot accurately state the presence of SCFAs in vesicles, despite the fact that acetate and butyrate were detected in *B. coprocola* EBA 6–21 and *B. finegoldii* EBA 6–28 vesicles, and we need additional experiments to confirm or refute this observation. Perhaps the use of ultrafiltration cartridges and pumps will provide a clearer answer to the question of the presence of SCFA in OMVs. The same statement is true for indole. Indeed, relative content decrease of indole in the bacterial media purified from OMVs was noted. It is possibly indirectly associated with the presence of this metabolite in the OMVs. However, indole was not found in vesicles. Nevertheless, taking into account that its relative concentration has been decreased in media after vesicles purification we can assume that indole is present in *B. cellulosilyticus* 807, *B. salyersiae* 2697, *Bacteroides xylanisolvens* Pik, *B. eggerthii* 91, *B. xylanisolvens* EBA5-17 OMVs.

As in bacterial media, 2,4-di-tert-butylphenol was detected in a significant relative amount in OMVs mostly in *B. fragilis* BOB25 and *B. coprocola* EBA 6–21 vesicles. At the same time, it is obvious that, unlike the most other components, the process of OMVs preparation contributed to the 2,4-Di-tert-butylphenol concentration. It can be assumed that 2,4-Di-tert-butylphenol possibly exhibits its activity in OMVs far beyond the intestine.

## Conclusion

In the course of our research, we have characterized the volatile fraction of the secretory metabolome of the most represented *Bacteroides* species. We were able to evaluate not only the spectrum of volatile components in bacterial media, but also for the first time the volatile spectrum of secreted vesicles was characterized. Based on our data, we can assume the secretory activity of bacteria potentially suitable for the formation of probiotics with described producers. In addition, we receive a new layer of fundamental data that can be studied in detail in additional experiments.

## Methods

### Bacterial strains and growth conditions

Bacterial strains used in this study can be found in Supplementary Table 1. The Sanger sequencing of the 16S rRNA amplified gene fragment was performed using 27F (5′-AGAGTTTGATCCTGGCTCAG-3′) and 1492R (5′-ACGGYTACCTTGTTACGACTT-3′) universal primers. The *Bacteroides* strains were stored at −80°C in a freeze-dried state in 20% (w/v) sucrose and 1% (w/v) gelatin solution. All strains were cultured anaerobically in Anaerobe Basal agar (Oxoid) supplemented with 5% (v/v) defibrinated sheep blood under anaerobic conditions that were generated by Anaerogen™ sachets placed in anaerobic 3.5-l jars (Oxoid; Thermo Fisher Scientific, Inc.) or in anaerobic jars (Schuett-Biotec, Germany) at 37°C until stationary phase. For liquid culture, pre-cultures of the *Bacteroides* strains were grown anaerobically in Columbia Broth Base (Hi Media, India) supplemented 5 mg/L hemin in 40 mL broth (in 50 mL conical flasks) at 37°C until stationary phase. These cultures then were subcultured the end of the logarithmic growth phase by transferring aliquot of 10 mL into 200 mL broth, the growth conditions were the same. Curves of growth were controlled by measuring optical density at 600 nm. For cultivation of strain *Bacteroides plebeius* 2436 we used pre-reduced basal broth containing (g l − 1): tryptone (Difco), 10.0; papaic digest of soybean meal (BBL Phytone Peptone, BD Biosciences), 5.0; yeast extract (Difco), 10.0; Gelysate (BBL), 2.0; NaCl, 5.0; d-glucose, 2.0; sodium pyruvate, 1.0; sodium succinate, 0.5; sodium bicarbonate, 0.4; cysteine-HCl monohydrate, 0.25 and arginine hydrochloride, 1.0 supplemented 5 mg/L hemin.

### Ethics statement

Informed consents from volunteers were obtained and approved by the ethical committee of the Lopukhin Federal Research and Clinical Center of Physical-Chemical Medicine of Federal Medical Biological Agency, Moscow, Russian Federation (protocol No. 2018/05, dated 08.05.2018).

### OMV isolation

Two hundred fifty milliliters of 24 h liquid bacterial culture of each *Bacteroides* species were centrifuged at 4,500*g* at 4°C. In order to remove residual cells, the supernatant was filtered using a 0.45 μm pore membrane. (Millex GV; Millipore) The filtrate was subjected to ultracentrifugation at 100,000*g_n_* for 2 h (Optima L-90 K ultracentrifuge; Beckman Coulter). The supernatant was discarded; the pellet was washed with sterile PBS and filtered through a sterile 0.2 μm-pore polyvinylidene difluoride (PVDF) membrane (Millex GV; Millipore). The ultracentrifugation step was repeated twice. The vesicle pellet was resuspended in distilled water or 150 mM NaCl (pH 6.5). Protein concentration was quantified using the 2D-quant kit (GE Healthcare Life Sciences).

### Nanoparticle tracking analysis

Hydrodynamic particle size distribution and concentration were measured with nanoparticle tracking analysis. Nanosight LM10 HS-BF instrument (Nanosight Ltd., Salisbury, United Kingdom) was used in the following configuration: 405 nm, 65 mW laser unit with a passive temperature readout, high sensitivity camera of EMCCD type, and NTA 2.3 build 33 software. Samples were diluted with PBS (pH 7.4) to reach the optimal concentration for NTA. As long as vesicles from different species vary strongly in scattering intensity, three camera and processing settings were used (in descending sensitivity): (1) Shutter = 1,230, Gain = 450, Lower Threshold = 1,560, Higher Threshold = 7,150, Detection Threshold = 14, (2) Shutter = 850, Gain = 450, Lower Threshold = 910, Higher Threshold = 8,060, Detection Threshold = 10, and (3) Shutter = 850, Gain = 450, Lower Threshold = 910, Higher Threshold = 10,920, Detection Threshold = 10. For each sample selection was made based on the apparent brightness of scattering spots. Min track length = Auto and Min expected particle size of 30 nm were used for all samples. Measurements were done in several repeats (14 to 21) to get at least 5,000 tracks in total. Data from all repeats were merged to get the joint particle size distribution and total particle concentration corrected by dilution factor.

### Electron microscopy (TEM)

The OMV were diluted to a concentration in the range of 2–5 × 10^11^ particles/mL and deposited onto TEM grids (copper grids with a formvar-carbon film, Ted Pella, Redding, United States). To increase the adsorption of the particles, the grids were pretreated using a glow discharge device Emitech K100X (Quorum Technologies Ltd., Lewes, United Kingdom) at 25 mA current for 45 s. The suspension of OMVs was deposited onto the grid for ~1 min, and the liquid was blotted using filter paper. Then the grids stained with 1% uranyl acetate for 1–2 min, blotted again and dried. Images were obtained using a JEM-1400 (Jeol, Tokyo, Japan) transmission electron microscope equipped with a Rio-9 camera (Gatan Inc., Pleasanton, CA, United States) at 120 kV. The sizes of the OMVs were measured using ScanEV software (Kulagina et al., 2012).

### HS-GC/MS

200 of μL of culture media samples or 100 μL OMV plus 100 μL water samples were placed into 10 mL screw-cap vials for a Shimadzu HS-20 headspace extractor. 0.2 g of a mixture of salts (ammonium sulfate and potassium dihydrogen phosphate in a ratio of 4:1) was added to increase solution ionic strength. Headspace extractor settings used: oven temperature 80°C, sample line temperature 220°C, transfer line temperature 220°C, equilibrating time 15 min, pressurizing time 2 min, load time 0.5 min, injection time 1 min, needle flush time 7 min. The vials were sealed and analyzed on a Shimadzu.

QP2010 Ultra GC / MS with a Shimadzu HS-20 headspace extractor, a VF-WAX MS column with a length of 30 m, a diameter of 0.25 mm, and a phase thickness of 0.25 microns. Initial column temperature 80°C, heating rate 20°C/min to 240°C, exposure 20 min. Carrier gas – helium 99.9999, injection mode – splitless, flow rate 1 mL/min. Ion source temperature – 230°C. Interface temperature 240°C. Total ionic current (TIC) monitoring mode was used. To analyze the obtained mass spectra, the NIST 2014 mass spectra library with automated mass spectral deconvolution and identification system (AMDIS version 2.72) was used.

### Data processing

The HS-GC/MS data were processed as follows. Peak areas computed by AMDIS for the selected compounds were converted to relative abundances. To build heatmaps, three biological replicates obtained for each sample were averaged. The t-SNE decomposition algorithm was used to additionally show the difference between samples.

## Data availability statement

The datasets presented in this study can be found in online repositories. The names of the repository/repositories and accession number(s) can be found in the article/Supplementary material.

## Ethics statement

The studies involving human participants were reviewed and approved by Ethical Committee of the Lopukhin Federal Research and Clinical Center of Physical-Chemical Medicine of Federal Medical Biological Agency, Moscow, Russian Federation (protocol no. 2018/05, dated 08.05.2018). The patients/participants provided their written informed consent to participate in this study.

## Author contributions

OS, DaK, DmK, DNK, BE, DB, EE, and NZ designed and performed the experiments, analyzed the data, and wrote the paper. AC, AS, JF, and IK performed the experiments. AV and JS wrote the paper. NZ wrote the paper and supervised the project. All authors contributed to the article and approved the submitted version.

## Funding

This research was supported by RSF grant 21-75-10172.

## Conflict of interest

The authors declare that the research was conducted in the absence of any commercial or financial relationships that could be construed as a potential conflict of interest.

## Publisher’s note

All claims expressed in this article are solely those of the authors and do not necessarily represent those of their affiliated organizations, or those of the publisher, the editors and the reviewers. Any product that may be evaluated in this article, or claim that may be made by its manufacturer, is not guaranteed or endorsed by the publisher.

## References

[ref1] Cheng C. W. Lin H. S. Ye J. J. Yang C. C. Chiang P. C. Wu T. S.. (2009). Clinical significance of and outcomes for *Bacteroides fragilis* bacteremia. J. Microbiol. Immunol. Infect. 42, 243–250. 19812858

[ref2] Coman V. Vodnar C. (2020). Hydroxycinnamic acids and human health: recent advances. J. Sci. Food Agric. 100, 483–499. doi: 10.1002/jsfa.10010, PMID: 31472019

[ref3] Davie J. R. (2003). Inhibition of histone deacetylase activity by butyrate. J. Nutr. 133, 2485S–2493S. doi: 10.1093/jn/133.7.2485S 12840228

[ref4] den Besten G. Bleeker A. Gerding A. van Eunen K. Havinga R. van Dijk T. H.. (2015). Short-chain fatty acids protect against high-fat diet-induced obesity via a PPARγ-dependent switch from lipogenesis to fat oxidation. Diabetes 64, 2398–2408. doi: 10.2337/db14-1213 25695945

[ref5] Dixon E. Clubb C. Pittman S. Ammann L. Rasheed Z. Kazmi N.. (2011). Solid-phase microextraction and the human fecal VOC metabolome. PLoS One 6:4. doi: 10.1371/journal.pone.0018471, PMID: 21494609 PMC3072975

[ref6] Elhenawy W. Debelyy M. O. Feldman M. F. (2014). Preferential packing of acidic glycosidases and proteases into Bacteroides outer membrane vesicles. mBio 5, e00909–e00914. doi: 10.1128/mBio.00909-14, PMID: 24618254 PMC3952158

[ref7] Fathi P. Wu S. (2016). Isolation, detection, and characterization of enterotoxigenic *Bacteroides fragilis* in clinical samples. Open Microbiol. J. 10, 57–63. doi: 10.2174/1874285801610010057, PMID: 27335618 PMC4899533

[ref8] Filipiak W. Żuchowska K. Marszałek M. Depka D. Bogiel T. Warmuzińska N.. (2022). GC–MS profiling of volatile metabolites produced by *Klebsiella pneumoniae*. Front. Mol. Biosci. 9:1019290. doi: 10.3389/fmolb.2022.1019290, PMID: 36330222 PMC9623108

[ref9] Gibson S. A. Macfarlane G. T. (1988). Characterization of proteases formed by *Bacteroides fragilis*. J. Gen. Microbiol. 134, 2231–2240. doi: 10.1099/00221287-134-8-2231, PMID: 3075656

[ref10] Hahnke R. L. Meier-Kolthoff J. P. García-López M. Mukherjee S. Huntemann M. Ivanova N. N.. (2016). Markus Göker genome-based taxonomic classification of Bacteroidetes. Front. Microbiol. 7:2003. doi: 10.3389/fmicb.2016.02003, eCollection 2016 28066339 PMC5167729

[ref11] Han E. C. Choi S. Y. Lee Y. Park J. W. Hong S. H. Lee H. J. (2019). Extracellular RNAs in periodontopathogenic outer membrane vesicles promote TNF-α production in human macrophages and cross the blood-brain barrier in mice. FASEB J. 33, 13412–13422. doi: 10.1096/fj.201901575R 31545910 PMC6894046

[ref12] Karu N. Deng L. Slae M. Guo A. C. Sajed T. Huynh H.. (2018). A review on human fecal metabolomics: methods, applications and the human fecal metabolome database. Anal. Chim. Acta 1030, 1–24. doi: 10.1016/j.aca.2018.05.031, Epub 2018 May 12 30032758

[ref13] Kennedy K. M. Donkin S. S. Allen M. S. (2020). Effects of propionate concentration on short-term metabolism in liver explants from dairy cows in the postpartum period. J. Dairy Sci. 103, 11449–11460. doi: 10.3168/jds.2020-18914, PMID: 33222857

[ref14] Kimball B. A. (2016). Volatile metabolome: problems and prospects. Bioanalysis 8:19. doi: 10.4155/bio-2016-0203 27532599

[ref15] Konanov D. Zakharzhevskaya N. Kardonsky D. Zhgun E. Kislun Y. Silantyev S.. (2022). UniqPy: a tool for estimation of short-chain fatty acids composition by gas-chromatography/mass-spectrometry with headspace extraction. J. Pharm. Biomed. Anal. 212:114681. doi: 10.1016/j.jpba.2022.114681, PMID: 35202943

[ref16] Kulagina E. V. Efimov B. A. Maximov P. Y. Kafarskaia L. I. Chaplin A. V. Shkoporov A. N. (2012). Species composition of Bacteroidales order bacteria in the feces of healthy people of various ages. Biosci. Biotechnol. Biochem. 76, 169–171. doi: 10.1271/bbb.110434, PMID: 22232251

[ref17] Lee J. H. Lee J. (2010). Indole as an intercellular signal in microbial communities. FEMS Microbiol. Rev. 34:4. doi: 10.1111/j.1574-6976.2009.00204.x 20070374

[ref18] Leila A. Lamjed B. Roudaina B. Najla T. Taamalli A. Jellouli S.. (2019). Isolation of an antiviral compound from Tunisian olive twig cultivars. Microb. Pathog. 128, 245–249. doi: 10.1016/j.micpath.2019.01.012, PMID: 30633983

[ref19] Leonard W. Zhang P. Ying D. Fang Z. (2021). Hydroxycinnamic acids on gut microbiota and health. Compr. Rev. Food Sci. Food Saf. 20, 710–737. doi: 10.1111/1541-4337.12663, PMID: 33443803

[ref20] Ley R. E. Hamady M. Lozupone C. Turnbaugh P. J. Ramey R. R. Bircher J. S.. (2008). Evolution of mammals and their gut microbes. Science 320, 1647–1651. doi: 10.1126/science.1155725, PMID: 18497261 PMC2649005

[ref21] Liebeke M. Dörries K. Meyer H. Lalk M. (2012). Metabolome analysis of gram-positive bacteria such as *Staphylococcus aureus* by GC–MS and LC-MS. Methods Mol. Biol. 815:377. doi: 10.1007/978-1-61779-424-7_28 22131006

[ref22] Louis P. Flint H. J. (2017). Formation of propionate and butyrate by the human colonic microbiota. Environ. Microbiol. 19, 29–41. doi: 10.1111/1462-2920.13589 27928878

[ref23] Marrubini G. Dugheri S. Cappelli G. Arcangeli G. Mucci N. Appelblad P.. (2020). Experimental designs for solid-phase microextraction method development in bioanalysis: a review. Anal. Chim. Acta 1119, 77–100. doi: 10.1016/j.aca.2020.04.012, PMID: 32439057

[ref24] Mielko K. A. Jabłoński S. J. Milczewska J. Sands D. Łukaszewicz M. Młynarz P. (2019). Metabolomic studies of *Pseudomonas aeruginosa*. World J. Microbiol. Biotechnol. 35:178. doi: 10.1007/s11274-019-2739-1, PMID: 31701321 PMC6838043

[ref25] Nair R. V. R. Jayasree D. V. Biju P. G. Baby S. (2020). Anti-inflammatory and anticancer activities of erythrodiol-3-acetate and 2,4-di-tert-butylphenol isolated from Humboldtia unijuga. Nat. Prod. Res. 34, 2319–2322. doi: 10.1080/14786419.2018.1531406, PMID: 30475646

[ref26] Niccolai E. Baldi S. Ricci F. Russo E. Nannini G. Menicatti M.. (2019). Evaluation and comparison of short chain fatty acids composition in gut diseases. World J. Gastroenterol. 25, 5543–5558. doi: 10.3748/wjg.v25.i36.5543, PMID: 31576099 PMC6767983

[ref27] Nikishin I. Dulimov R. Skryabin G. Galetsky S. Tchevkina E. Bagrov D. (2021). ScanEV – a neural network-based tool for the automated detection of extracellular vesicles in TEM images. Micron 145:103044. doi: 10.1016/j.micron.2021.103044, Epub 2021 Feb 26 33676158

[ref28] Pei T. Zhu D. Yang S. Hu R. Wang F. Zhang J.. (2022). *Bacteroides plebeius* improves muscle wasting in chronic kidney disease by modulating the gut-renal muscle axis. J. Cell. Mol. Med. 26, 6066–6078. doi: 10.1111/jcmm.17626, PMID: 36458537 PMC9753468

[ref29] Pirolli N. H. Bentley W. E. Jay S. M. (2021). Bacterial extracellular vesicles and the gut-microbiota brain axis: emerging roles in communication and potential as therapeutics. Adv. Biol. 5:7. doi: 10.1002/adbi.202000540, PMID: 33857347

[ref30] Reeves A. R. Wang G. R. Salyers A. A. (1997). Characterization of four outer membrane proteins that play a role in utilization of starch by *Bacteroides thetaiotaomicron*. J. Bacteriol. 179, 643–649. doi: 10.1128/jb.179.3.643-649.1997, PMID: 9006015 PMC178742

[ref31] Rondanelli M. Perdoni F. Infantino V. Faliva M. A. Peroni G. Iannello G.. (2019). Volatile organic compounds as biomarkers of gastrointestinal diseases and nutritional status. J. Anal. Methods Chem. 2019, 7247802–7247814. doi: 10.1155/2019/7247802, PMID: 31583160 PMC6754926

[ref32] Shipman J. A. Berleman J. E. Salyers A. A. (2000). Characterization of four outer membrane proteins involved in binding starch to the cell surface of *Bacteroides thetaiotaomicron*. J. Bacteriol. 182, 5365–5372. doi: 10.1128/JB.182.19.5365-5372.2000, PMID: 10986238 PMC110978

[ref33] Spence C. Wells W. G. Smith C. J. (2006). Characterization of the primary starch utilization operon in the obligate anaerobe *Bacteroides fragilis*: regulation by carbon source and oxygen. J. Bacteriol. 188, 4663–4672. doi: 10.1128/JB.00125-06, PMID: 16788175 PMC1482989

[ref34] Telesford K. M. Yan W. Ochoa-Reparaz J. Pant A. Kircher C. Christy M. A.. (2015). A commensal symbiotic factor derived from *Bacteroides fragilis* promotes human CD39(+) Foxp3(+) T cells and Treg function. Gut Microbes 6, 234–242. doi: 10.1080/19490976.2015.1056973, PMID: 26230152 PMC4615798

[ref35] Thaiss C. A. Zmora N. Levy M. Elinav E. (2016). The microbiome and innate immunity. Nature 535, 65–74. doi: 10.1038/nature18847, PMID: 27383981

[ref36] Tyakht A. V. Kostryukova E. S. Popenko A. S. Belenikin M. S. Pavlenko A. V. Larin A. K.. (2013). Human gut microbiota community structures in urban and rural populations in Russia. Nat. Commun. 4:2469. doi: 10.1038/ncomms3469 24036685 PMC3778515

[ref37] Wenzel T. J. Gates E. J. Ranger A. L. Klegeris A. (2020). Short-chain fatty acids (SCFAs) alone or in combination regulate select immune functions of microglia-like cells. Mol. Cell. Neurosci. 105:103493. doi: 10.1016/j.mcn.2020.103493 32333962

[ref38] Yoon M. A. Jeong T. S. Park D. S. Xu M. Z. Oh H. W. Song K. B.. (2006). Antioxidant effects of quinoline alkaloids and 2,4-di-tert-butylphenol isolated from *Scolopendra subspinipes*. Biol. Pharm. Bull. 29, 735–739. doi: 10.1248/bpb.29.735 16595909

[ref39] Zakharzhevskaya N. B. Vanyushkina A. A. Altukhov I. A. Shavarda A. L. Butenko I. O. Rakitina D. V.. (2017). Outer membrane vesicles secreted by pathogenic and nonpathogenic *Bacteroides fragilis* represent different metabolic activities. Sci. Rep. 7:5008. doi: 10.1038/s41598-017-05264-6, PMID: 28694488 PMC5503946

[ref40] Zhao F. Wang P. Lucardi R. D. Su Z. Li S. (2020). Natural sources and bioactivities of 2,4-Di-Tert-Butylphenol and its analogs. Toxins 12:1. doi: 10.3390/toxins12010035, PMID: 31935944 PMC7020479

