# Supplementary material for: Investigating volatile compounds in the Bacteroides secretome
Source: Front Microbiol. 2023 May 3;14:1164877. doi: 10.3389/fmicb.2023.1164877 (PMC10189065; doi:10.3389/fmicb.2023.1164877)
Supplement: Supplementary file 1 [file Table_1.DOCX]

**Details of *Bacteroides* strains utilized in this study for experimental analysis**

| **No.** | **Strain** | **Isolation source** | **Submitting Institute** | **Genome or 16S rRNA gene accession number** | **References** |
| --- | --- | --- | --- | --- | --- |
| 1 | *Bacteroides uniformis* EBA5-20 | feces, 2005, Russia: Moscow | PRNRMU^*)^ | EF608193 | Shkoporov A.N. et al., 2008 |
| 2 | *Bacteroides thetaiotaomicron* 6-237 | feces, 2013, Russia: Moscow | PRNRMU | OQ645440 |  |
| 3 | *Bacteroides caccae* EBA6-24 | feces, 2005, Russia: Moscow | PRNRMU | EF608195 | Shkoporov A.N. et al., 2008 |
| 4 | *Bacteroides eggerthii* 91 | feces, 2013, Russia: Moscow | PRNRMU | OQ645445 |  |
| 5 | *Bacteroides stercoris* 5888 | feces, 2020, Russia: Moscow | PRNRMU | OQ645437 |  |
| 6 | *Bacteroides intestinalis* 181 | feces, 2013, Russia: Moscow | PRNRMU | OQ645441 |  |
| 7 | *Bacteroides clarus* 606 | feces, 2013, Russia: Moscow | PRNRMU | OQ645443 |  |
| 8 | *Bacteroides salyersiae* 2697 | feces, 2018, Russia: Moscow | PRNRMU | OQ645439 | Efimov, B.A. et al., 2019 |
| 9 | *Bacteroides xylanisolvens* EBA5-17 | feces, 2005, Russia: Moscow | PRNRMU | EF608211 | Shkoporov A.N. et al., 2008 |
| 10 | *Bacteroides хylanisolvens* Pik | feces, 2016, Russia: Moscow | PRNRMU | OQ645438 |  |
| 11 | *Bacteroides finegoldii* EBA6-28 | feces, 2005, Russia: Moscow | PRNRMU | EF608207 | Shkoporov A.N. et al., 2008 |
| 12 | *Bacteroides cellulosilyticus* 807 | feces, 2013, Russia: Moscow | PRNRMU | OQ645444 |  |
| 13 | *Bacteroides fragilis* BOB25 | stool sample, 2013, Russia: Saint Petersburg | LFRCC PCM^**)^ | CP011073 | Zakharzhevskaya N.B. et al., 2017 |
| 14 | *Bacteroides fragilis* JIM10 | stool sample, 2013, Russia: Moscow | LFRCC PCM | MBRB00000000 | Zakharzhevskaya N.B. et al., 2017 |
| 15 | *Bacteroides dorei* EBA7-24 | feces, 2005, Russia: Moscow | PRNRMU | EF608204 | Shkoporov A.N. et al., 2008 |
| 16 | *Bacteroides vulgatus* EBA3-9 | feces, 2005, Russia: Moscow | PRNRMU | EF608202 | Shkoporov A.N. et al., 2008 |
| 17 | *Bacteroides coprocola* EBA6-21 | feces, 2005, Russia: Moscow | PRNRMU | EF608194 | Shkoporov A.N. et al., 2008 |
| 18 | *Bacteroides plebeius* 2436 | feces, 2017, Russia: Moscow | PRNRMU | OQ645442 | Efimov, B.A. et al., 2019 |

^*)^ PRNRMU - Pirogov Russian National Research Medical University of the Ministry of Health of the Russian Federation, Moscow, Russian Federation

^**)^ LFRCC PCM – Lopukhin Federal Research and Clinical Center of Physical-Chemical Medicine, Federal Medical and Biological Agency, Moscow, Russian Federation

Shkoporov, A.N., Khokhlova, E.V., Kulagina, E.V., Smeianov, V.V., Kafarskaia, L.I. and Efimov, B.A.

Application of several molecular techniques to study numerically predominant Bifidobacterium spp. and Bacteroidales order strains in the feces of healthy children. (2008) Biosci. Biotechnol. Biochem. 72:(3)742-748

Zakharzhevskaya, N.B., Vanyushkina, A.A., Altukhov, I.A., Shavarda, Aleksey L., Butenko, I.O., Rakitina, D.V., Nikitina, A.S., Manolov, A.I., Egorova, A.N., Kulikov, E.E., Vishnyakov, I.E., Fisunov, G.Y., Govorun, V.M. Outer membrane vesicles secreted by pathogenic and nonpathogenic *Bacteroides fragilis* represent different metabolic activities. (2017) Sci Rep 7, 5008

Efimov, B. A., Chaplin, A. V., Sokolova, S. R., Chernaia, Z. A., Pikina, A. P., Savilova, A. M., Kafarskaya, L.I. (2019), "Application of culture-based, mass spectrometry and molecular methods to the study of gut microbiota in children", Bulletin of RSMU, no. 4, p. 54–65.
