# Supplementary material for: Investigating volatile compounds in the Bacteroides secretome
Source: Front Microbiol. 2023 May 3;14:1164877. doi: 10.3389/fmicb.2023.1164877 (PMC10189065; doi:10.3389/fmicb.2023.1164877)
Supplement: Supplementary file 3 [file Data_Sheet_1.PDF]

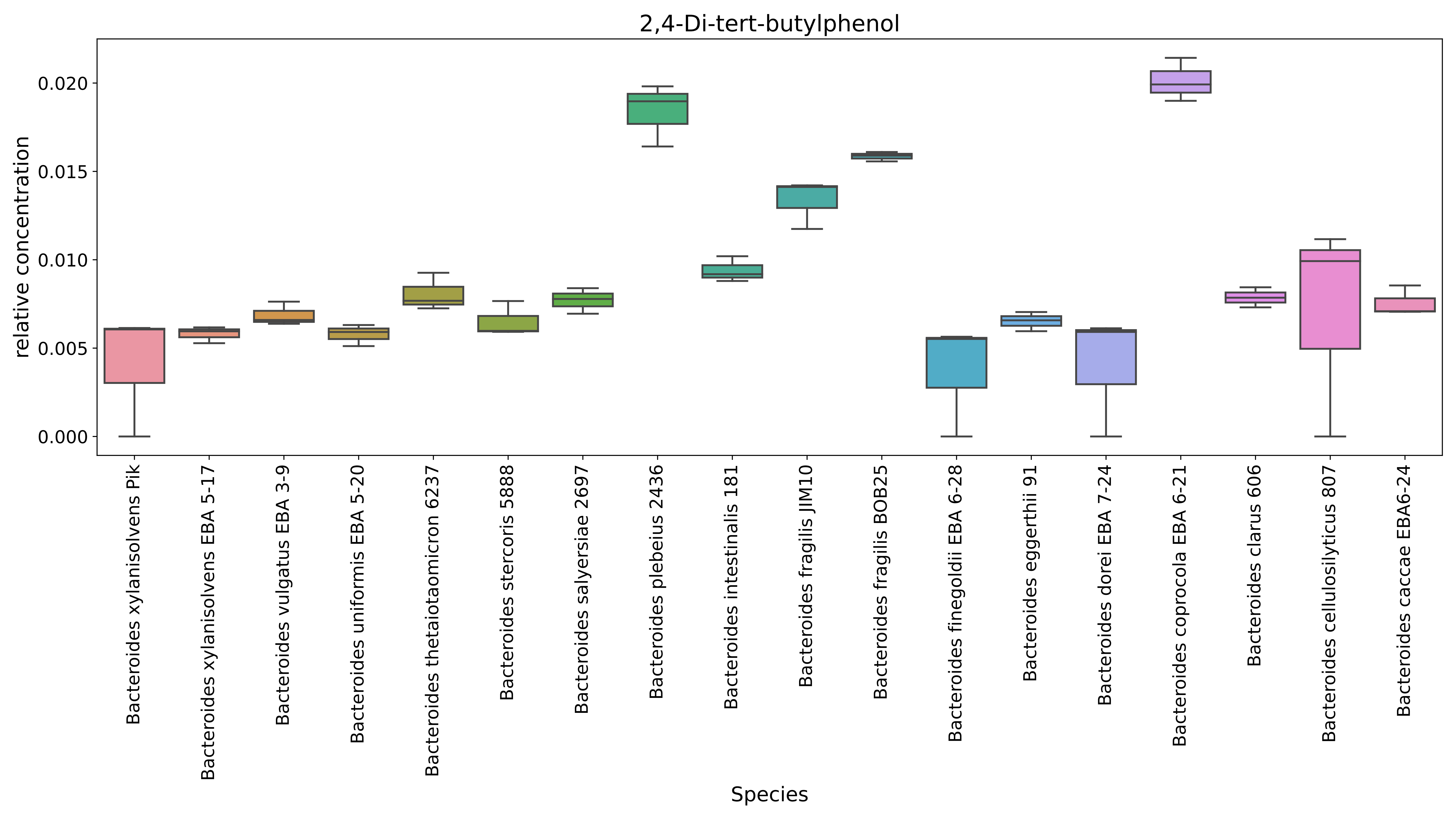
2,4-Di-tert-butylphenol
relative concentration
0.000
0.005
0.010
0.015
0.020
Bacteroides xylanisolvens Pik
Bacteroides xylanisolvens EBA 5-17
Bacteroides vulgatus EBA 3-9
Bacteroides uniformis EBA 5-20
Bacteroides thetaiotaomicron 6237
Bacteroides stercoris 5888
Bacteroides salyersiae 2697
Bacteroides plebeius 2436
Bacteroides intestinalis 181
Bacteroides fragilis JIM10
Bacteroides fragilis BOB25
Bacteroides finegoldii EBA 6-28
Bacteroides eggerthii 91
Bacteroides dorei EBA 7-24
Bacteroides coprocola EBA 6-21
Bacteroides clarus 606
Bacteroides cellulosilyticus 807
Bacteroides caccae EBA6-24
Species

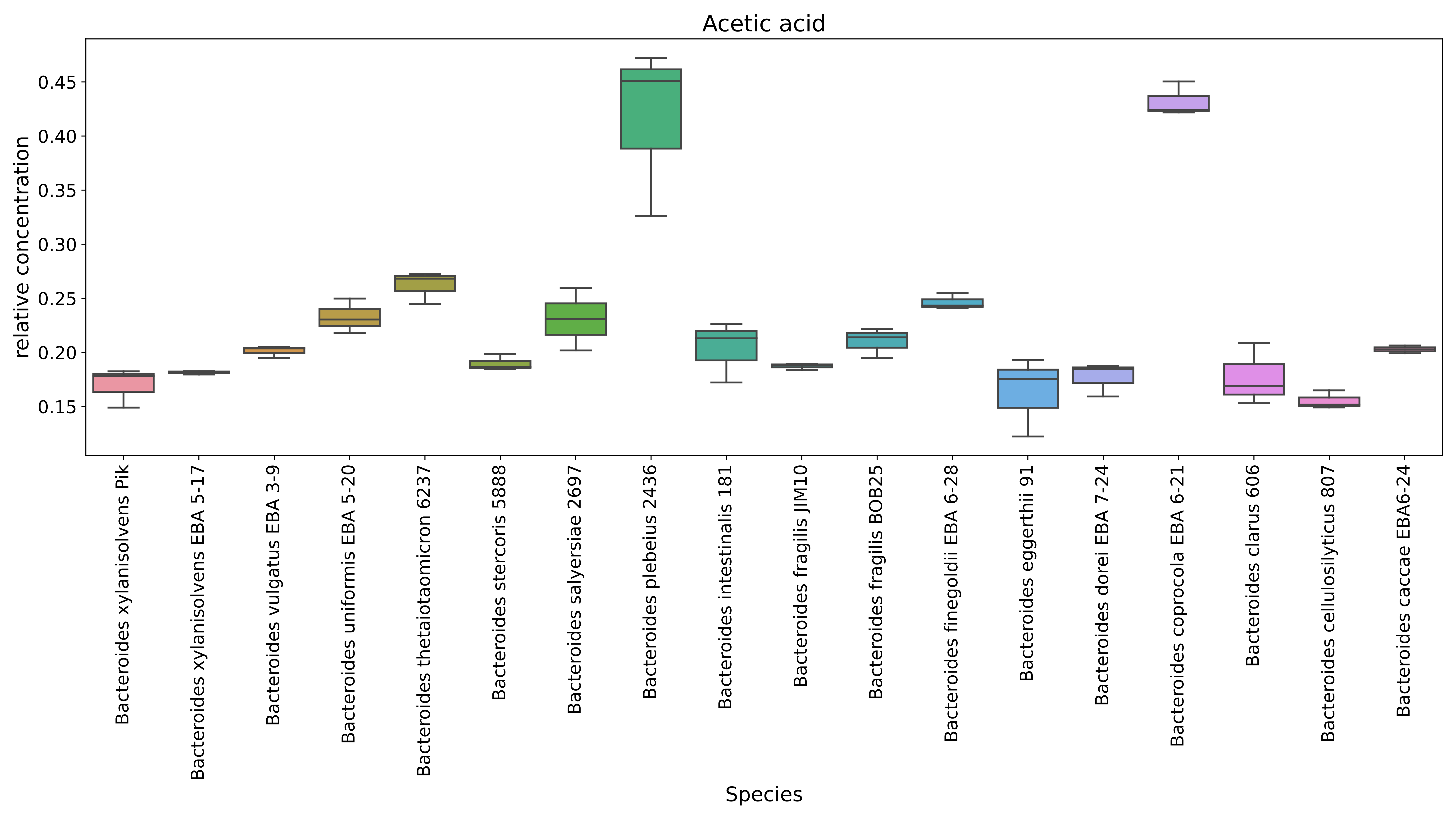
Acetic acid
relative concentration
0.45
0.40
0.35
0.30
0.25
0.20
0.15
Bacteroides xylanisolvens Pik
Bacteroides xylanisolvens EBA 5-17
Bacteroides vulgatus EBA 3-9
Bacteroides uniformis EBA 5-20
Bacteroides thetaiotaomicron 6237
Bacteroides stercoris 5888
Bacteroides salyersiae 2697
Bacteroides plebeius 2436
Bacteroides intestinalis 181
Bacteroides fragilis JIM10
Bacteroides fragilis BOB25
Bacteroides finegoldii EBA 6-28
Bacteroides eggerthii 91
Bacteroides dorei EBA 7-24
Bacteroides coprocola EBA 6-21
Bacteroides clarus 606
Bacteroides cellulosilyticus 807
Bacteroides caccae EBA6-24
Species

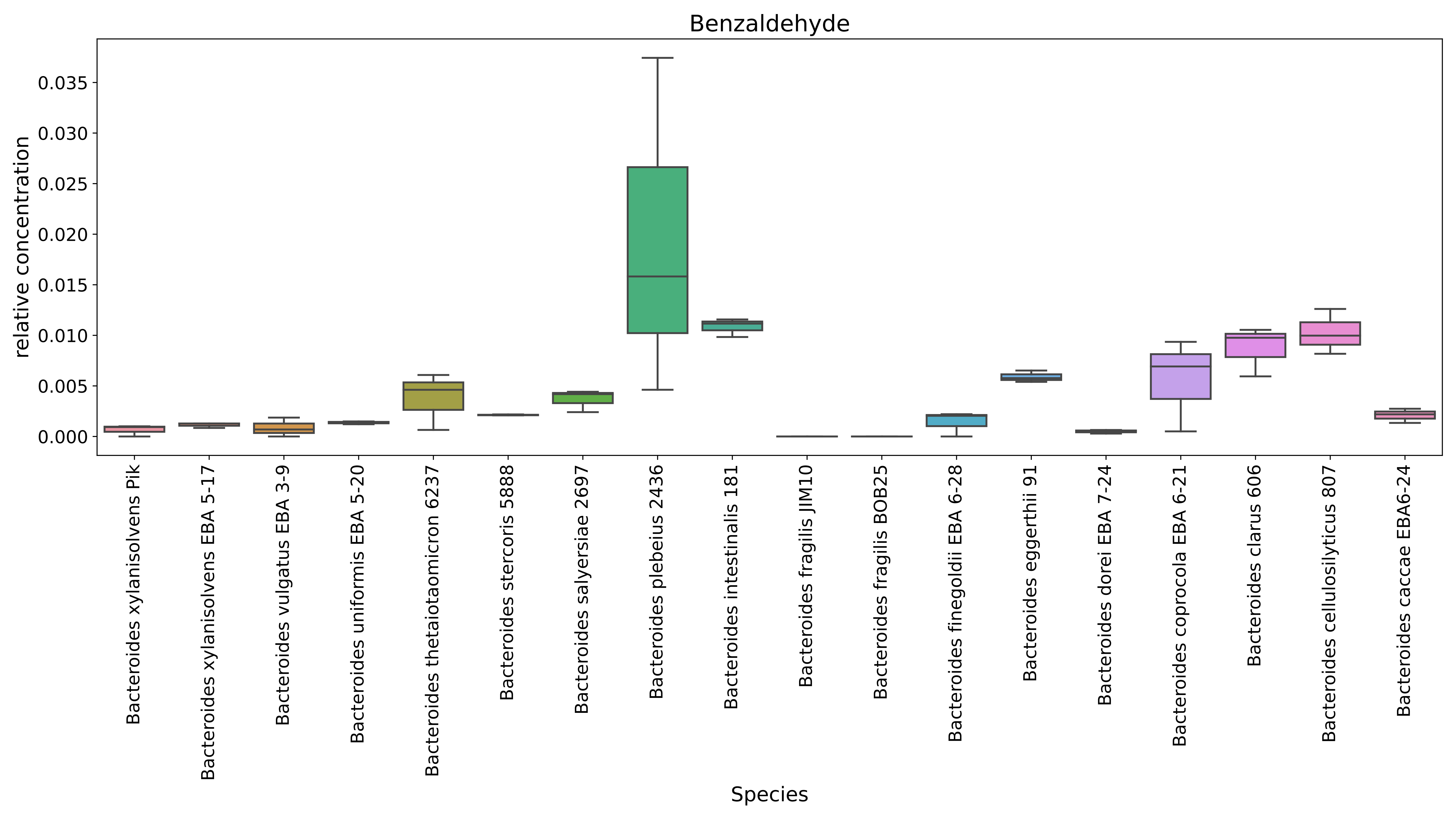
Benzaldehyde
relative concentration
0.035
0.030
0.025
0.020
0.015
0.010
0.005
0.000
Bacteroides xylanisolvens Pik
Bacteroides xylanisolvens EBA 5-17
Bacteroides vulgatus EBA 3-9
Bacteroides uniformis EBA 5-20
Bacteroides thetaiotaomicron 6237
Bacteroides stercoris 5888
Bacteroides salyersiae 2697
Bacteroides plebeius 2436
Bacteroides intestinalis 181
Bacteroides fragilis JIM10
Bacteroides fragilis BOB25
Bacteroides finegoldii EBA 6-28
Bacteroides eggerthii 91
Bacteroides dorei EBA 7-24
Bacteroides coprocola EBA 6-21
Bacteroides clarus 606
Bacteroides cellulosilyticus 807
Bacteroides caccae EBA6-24
Species

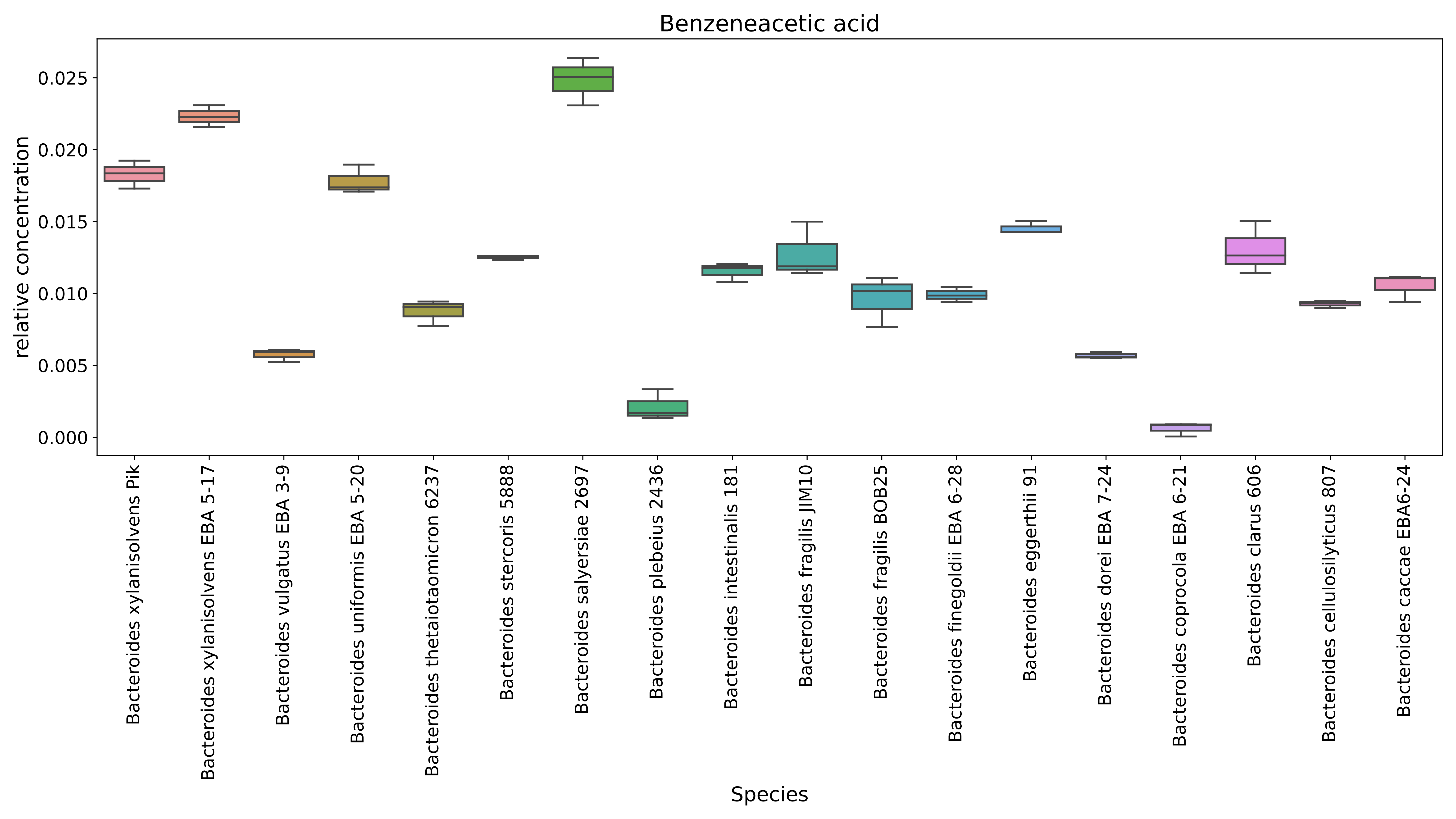

Benzeneacetic acid
relative concentration
0.000
0.005
0.010
0.015
0.020
0.025
Bacteroides xylanisolvens Pik
Bacteroides xylanisolvens EBA 5-17
Bacteroides vulgatus EBA 3-9
Bacteroides uniformis EBA 5-20
Bacteroides thetaiotaomicron 6237
Bacteroides stercoris 5888
Bacteroides salyersiae 2697
Bacteroides plebeius 2436
Bacteroides intestinalis 181
Bacteroides fragilis JIM10
Bacteroides fragilis BOB25
Bacteroides finegoldii EBA 6-28
Bacteroides eggerthii 91
Bacteroides dorei EBA 7-24
Bacteroides coprocola EBA 6-21
Bacteroides clarus 606
Bacteroides cellulosilyticus 807
Bacteroides caccae EBA6-24
Species

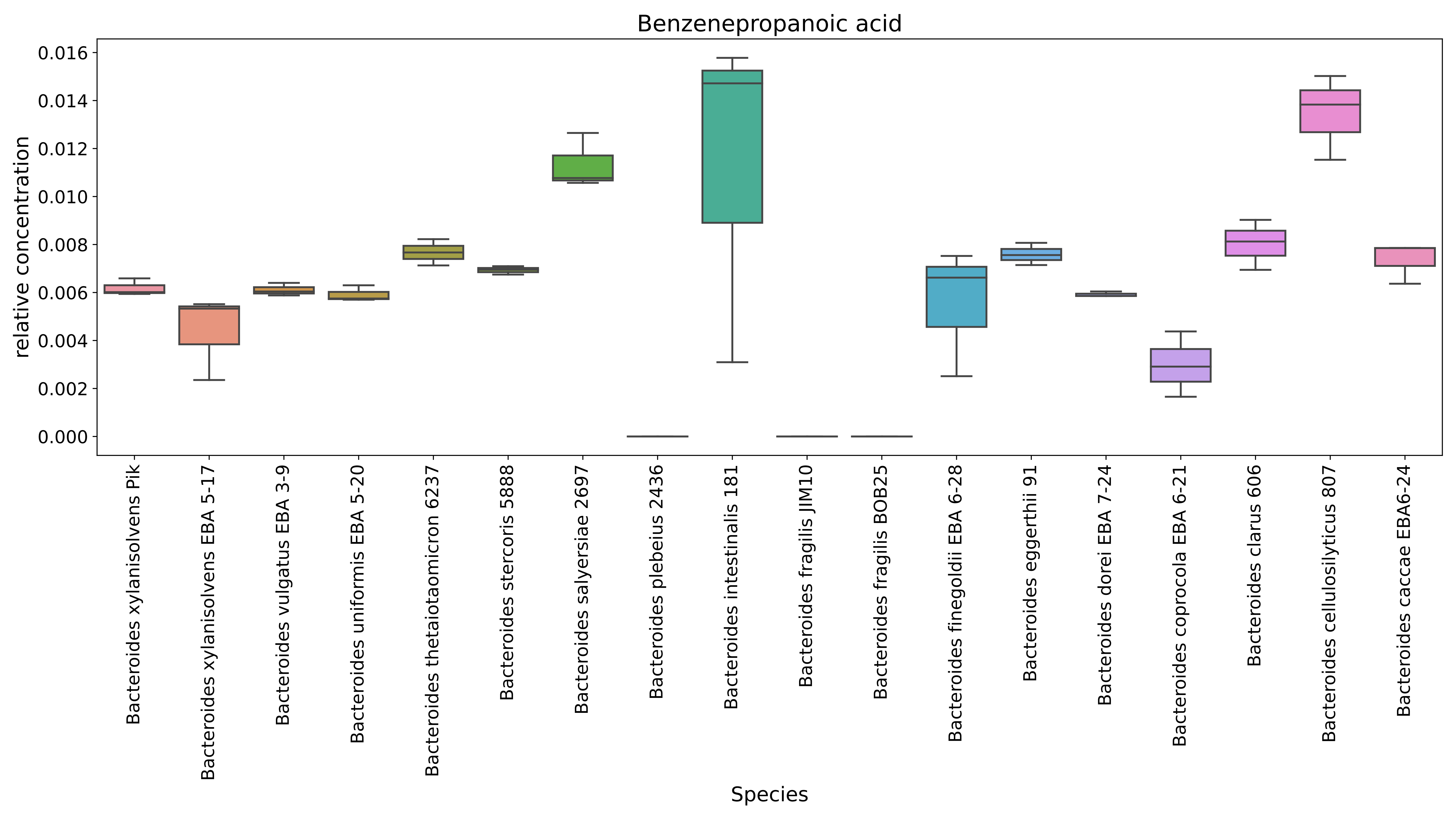

Benzenepropanoic acid
relative concentration
0.016
0.014
0.012
0.010
0.008
0.006
0.004
0.002
0.000
Bacteroides xylanisolvens Pik
Bacteroides xylanisolvens EBA 5-17
Bacteroides vulgatus EBA 3-9
Bacteroides uniformis EBA 5-20
Bacteroides thetaiotaomicron 6237
Bacteroides stercoris 5888
Bacteroides salyersiae 2697
Bacteroides plebeius 2436
Bacteroides intestinalis 181
Bacteroides fragilis JIM10
Bacteroides fragilis BOB25
Bacteroides finegoldii EBA 6-28
Bacteroides eggerthii 91
Bacteroides dorei EBA 7-24
Bacteroides coprocola EBA 6-21
Bacteroides clarus 606
Bacteroides cellulosilyticus 807
Bacteroides caccae EBA6-24
Species

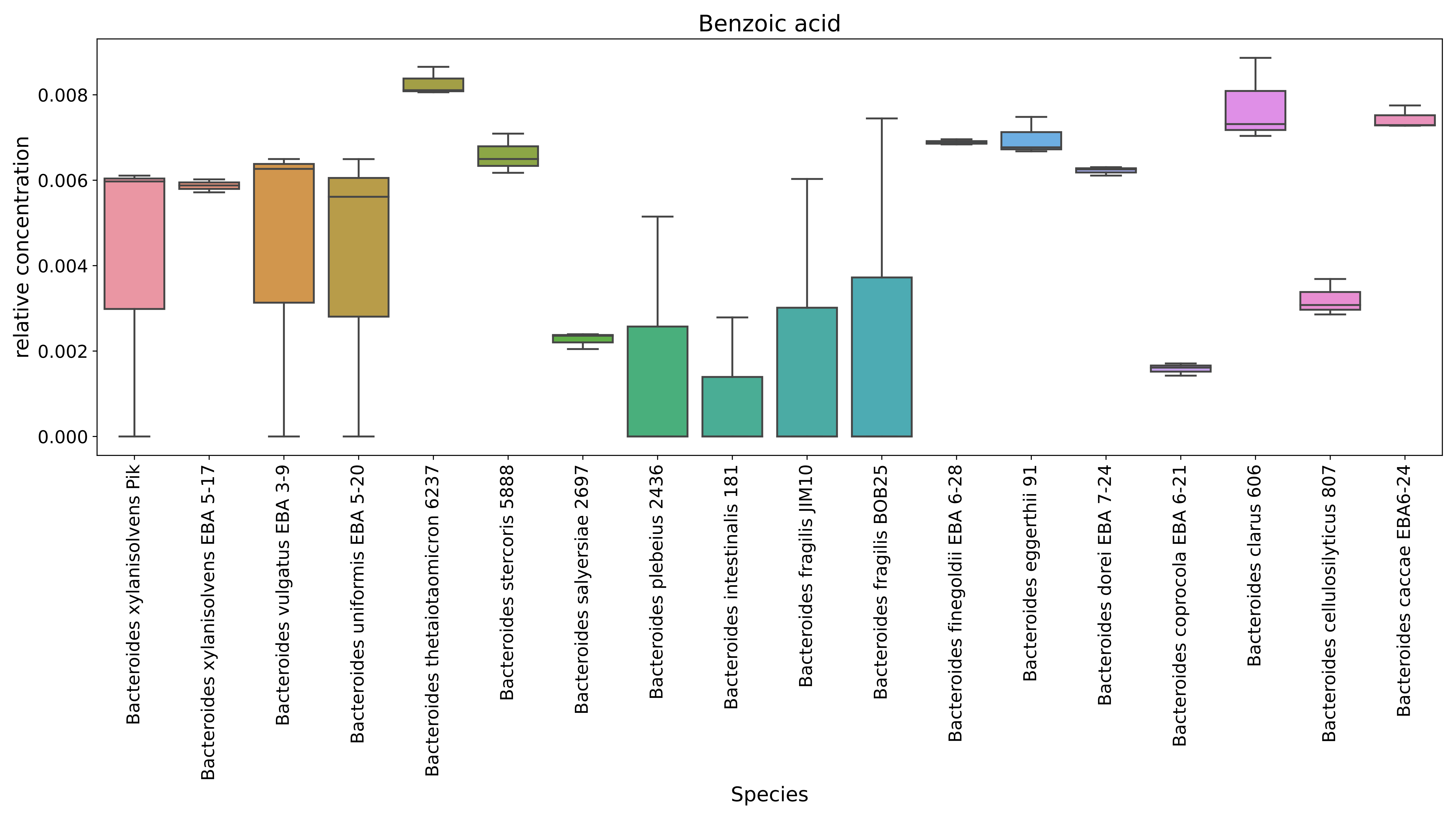
Benzoic acid
relative concentration
0.000
0.002
0.004
0.006
0.008
Bacteroides xylanisolvens Pik
Bacteroides xylanisolvens EBA 5-17
Bacteroides vulgatus EBA 3-9
Bacteroides uniformis EBA 5-20
Bacteroides thetaiotaomicron 6237
Bacteroides stercoris 5888
Bacteroides salyersiae 2697
Bacteroides plebeius 2436
Bacteroides intestinalis 181
Bacteroides fragilis JIM10
Bacteroides fragilis BOB25
Bacteroides finegoldii EBA 6-28
Bacteroides eggerthii 91
Bacteroides dorei EBA 7-24
Bacteroides coprocola EBA 6-21
Bacteroides clarus 606
Bacteroides cellulosilyticus 807
Bacteroides caccae EBA6-24
Species

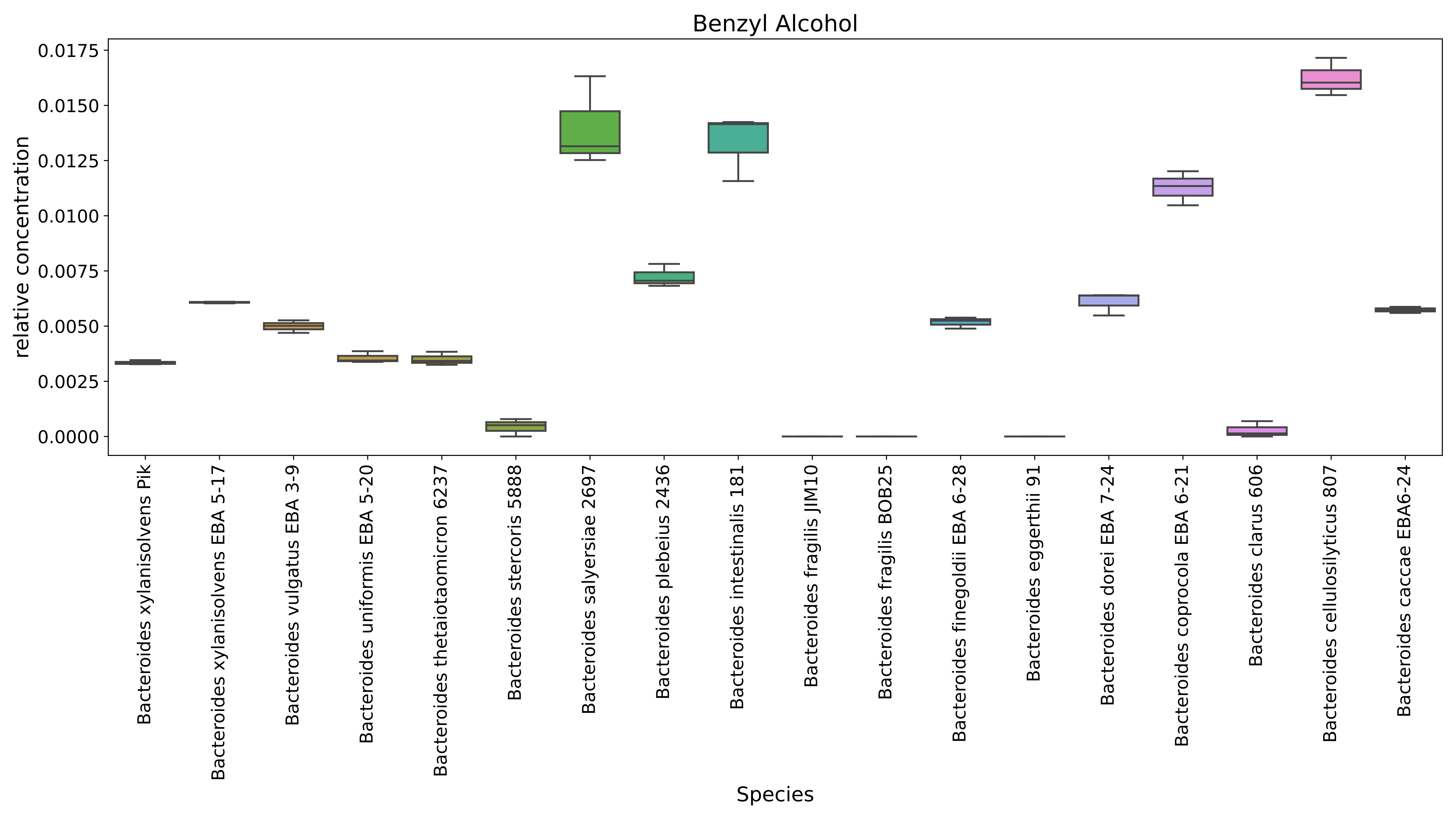
Benzyl Alcohol
relative concentration
0.0175
0.0150
0.0125
0.0100
0.0075
0.0050
0.0025
0.0000
Bacteroides xylanisolvens Pik
Bacteroides xylanisolvens EBA 5-17
Bacteroides vulgatus EBA 3-9
Bacteroides uniformis EBA 5-20
Bacteroides thetaiotaomicron 6237
Bacteroides stercoris 5888
Bacteroides salyersiae 2697
Bacteroides plebeius 2436
Bacteroides intestinalis 181
Bacteroides fragilis JIM10
Bacteroides fragilis BOB25
Bacteroides finegoldii EBA 6-28
Bacteroides eggerthii 91
Bacteroides dorei EBA 7-24
Bacteroides coprocola EBA 6-21
Bacteroides clarus 606
Bacteroides cellulosilyticus 807
Bacteroides caccae EBA6-24
Species

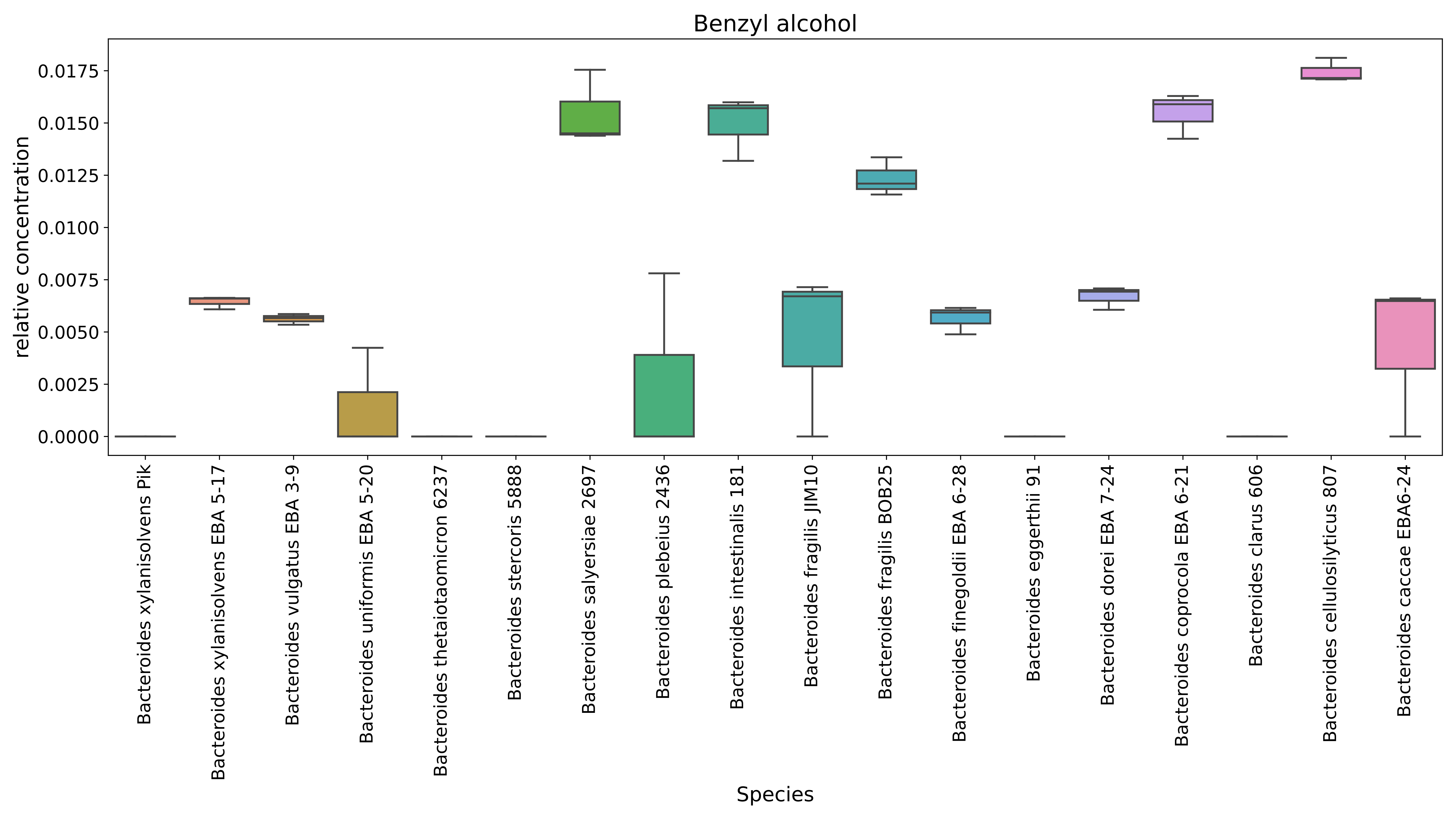
Benzyl alcohol
relative concentration
0.0000
0.0025
0.0050
0.0075
0.0100
0.0125
0.0150
0.0175
Species
Bacteroides xylanisolvens Pik
Bacteroides xylanisolvens EBA 5-17
Bacteroides vulgatus EBA 3-9
Bacteroides uniformis EBA 5-20
Bacteroides thetaiotaomicron 6237
Bacteroides stercoris 5888
Bacteroides salyersiae 2697
Bacteroides plebeius 2436
Bacteroides intestinalis 181
Bacteroides fragilis JIM10
Bacteroides fragilis BOB25
Bacteroides finegoldii EBA 6-28
Bacteroides eggerthii 91
Bacteroides dorei EBA 7-24
Bacteroides coprocola EBA 6-21
Bacteroides clarus 606
Bacteroides cellulosilyticus 807
Bacteroides caccae EBA6-24

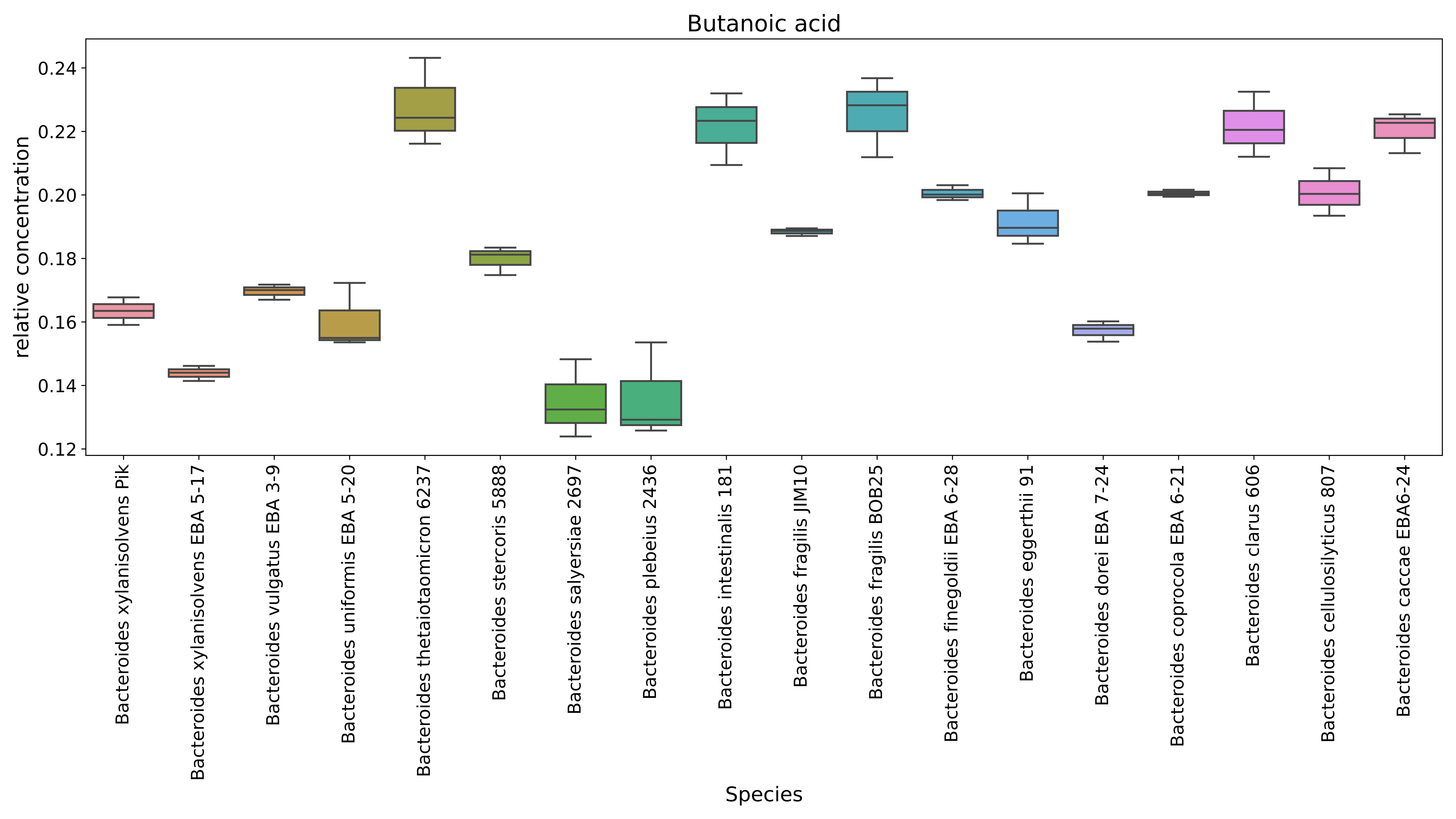

Butanoic acid
relative concentration
0.12
0.14
0.16
0.18
0.20
0.22
0.24
Species
Bacteroides xylanisolvens Pik
Bacteroides xylanisolvens EBA 5-17
Bacteroides vulgatus EBA 3-9
Bacteroides uniformis EBA 5-20
Bacteroides thetaiotaomicron 6237
Bacteroides stercoris 5888
Bacteroides salyersiae 2697
Bacteroides plebeius 2436
Bacteroides intestinalis 181
Bacteroides fragilis JIM10
Bacteroides fragilis BOB25
Bacteroides finegoldii EBA 6-28
Bacteroides eggerthii 91
Bacteroides dorei EBA 7-24
Bacteroides coprocola EBA 6-21
Bacteroides clarus 606
Bacteroides cellulosilyticus 807
Bacteroides caccae EBA6-24

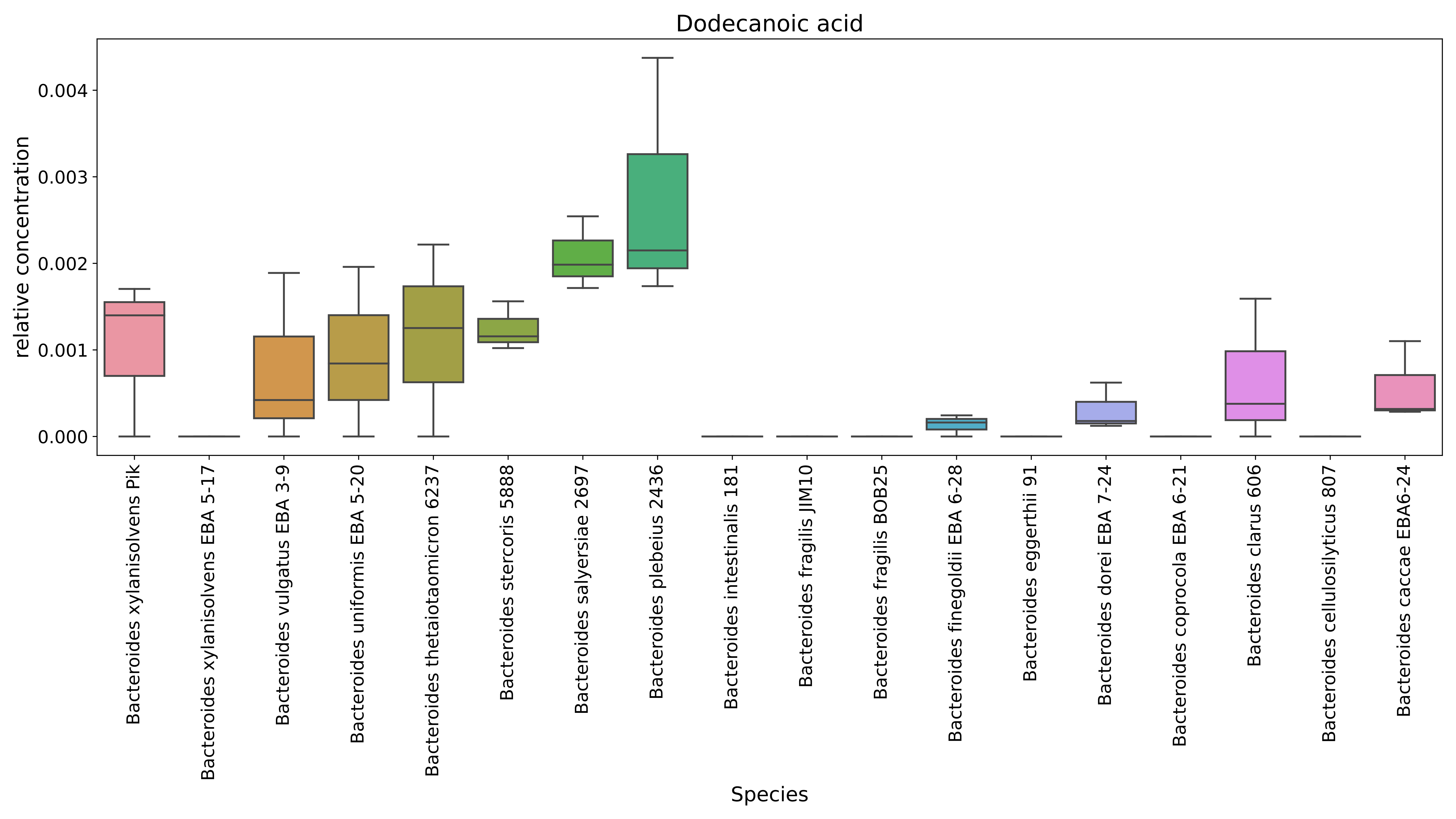
Dodecanoic acid
relative concentration
0.004
0.003
0.002
0.001
0.000
Species
Bacteroides xylanisolvens Pik
Bacteroides xylanisolvens EBA 5-17
Bacteroides vulgatus EBA 3-9
Bacteroides uniformis EBA 5-20
Bacteroides thetaiotaomicron 6237
Bacteroides stercoris 5888
Bacteroides salyersiae 2697
Bacteroides plebeius 2436
Bacteroides intestinalis 181
Bacteroides fragilis JIM10
Bacteroides fragilis BOB25
Bacteroides finegoldii EBA 6-28
Bacteroides eggerthii 91
Bacteroides dorei EBA 7-24
Bacteroides coprocola EBA 6-21
Bacteroides clarus 606
Bacteroides cellulosilyticus 807
Bacteroides caccae EBA6-24

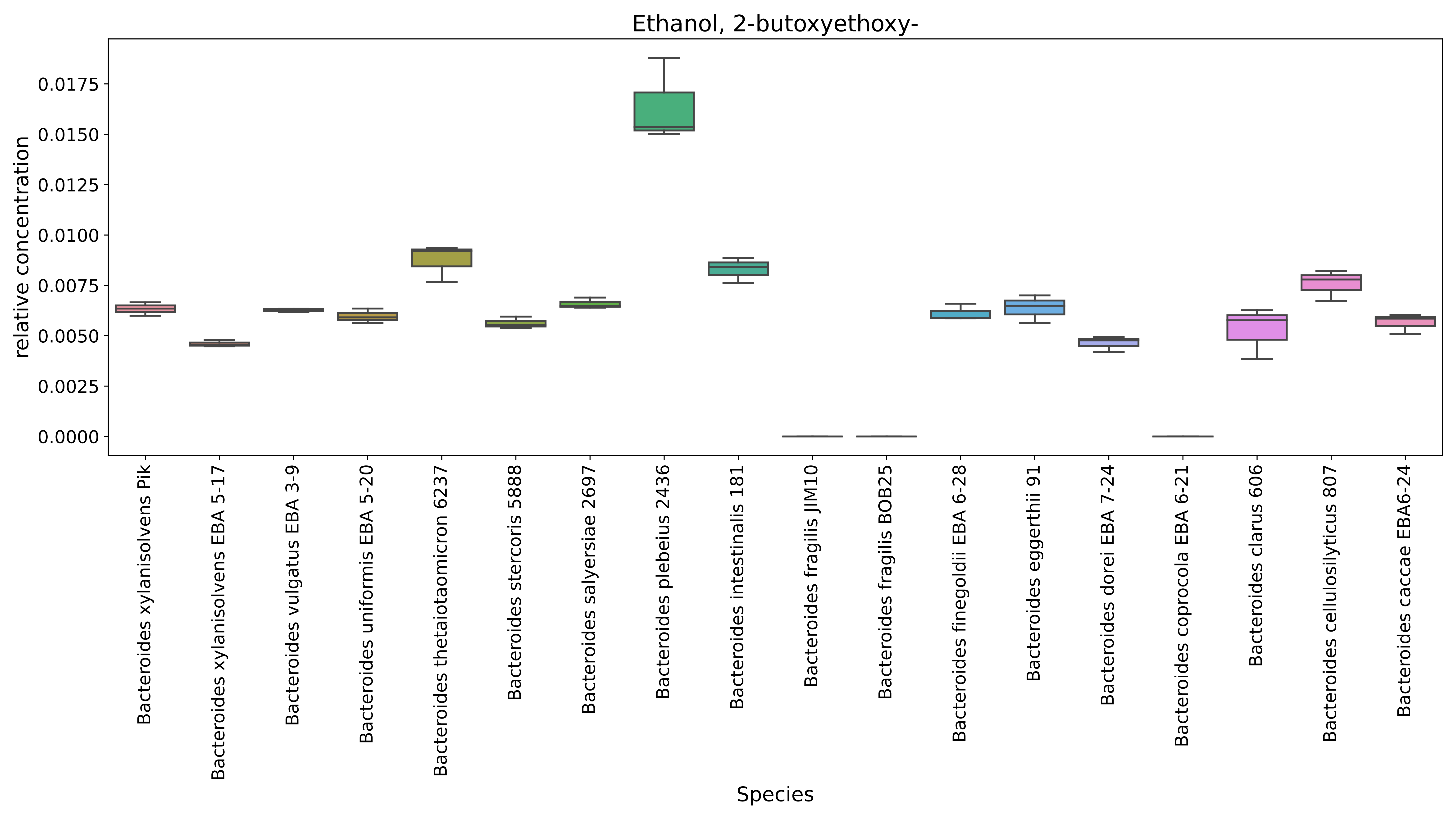
Ethanol, 2-butoxyethoxy-
relative concentration
0.0000
0.0025
0.0050
0.0075
0.0100
0.0125
0.0150
0.0175
Bacteroides xylanisolvens Pik
Bacteroides xylanisolvens EBA 5-17
Bacteroides vulgatus EBA 3-9
Bacteroides uniformis EBA 5-20
Bacteroides thetaiotaomicron 6237
Bacteroides stercoris 5888
Bacteroides salyersiae 2697
Bacteroides plebeius 2436
Bacteroides intestinalis 181
Bacteroides fragilis JIM10
Bacteroides fragilis BOB25
Bacteroides finegoldii EBA 6-28
Bacteroides eggerthii 91
Bacteroides dorei EBA 7-24
Bacteroides coprocola EBA 6-21
Bacteroides clarus 606
Bacteroides cellulosilyticus 807
Bacteroides caccae EBA6-24
Species

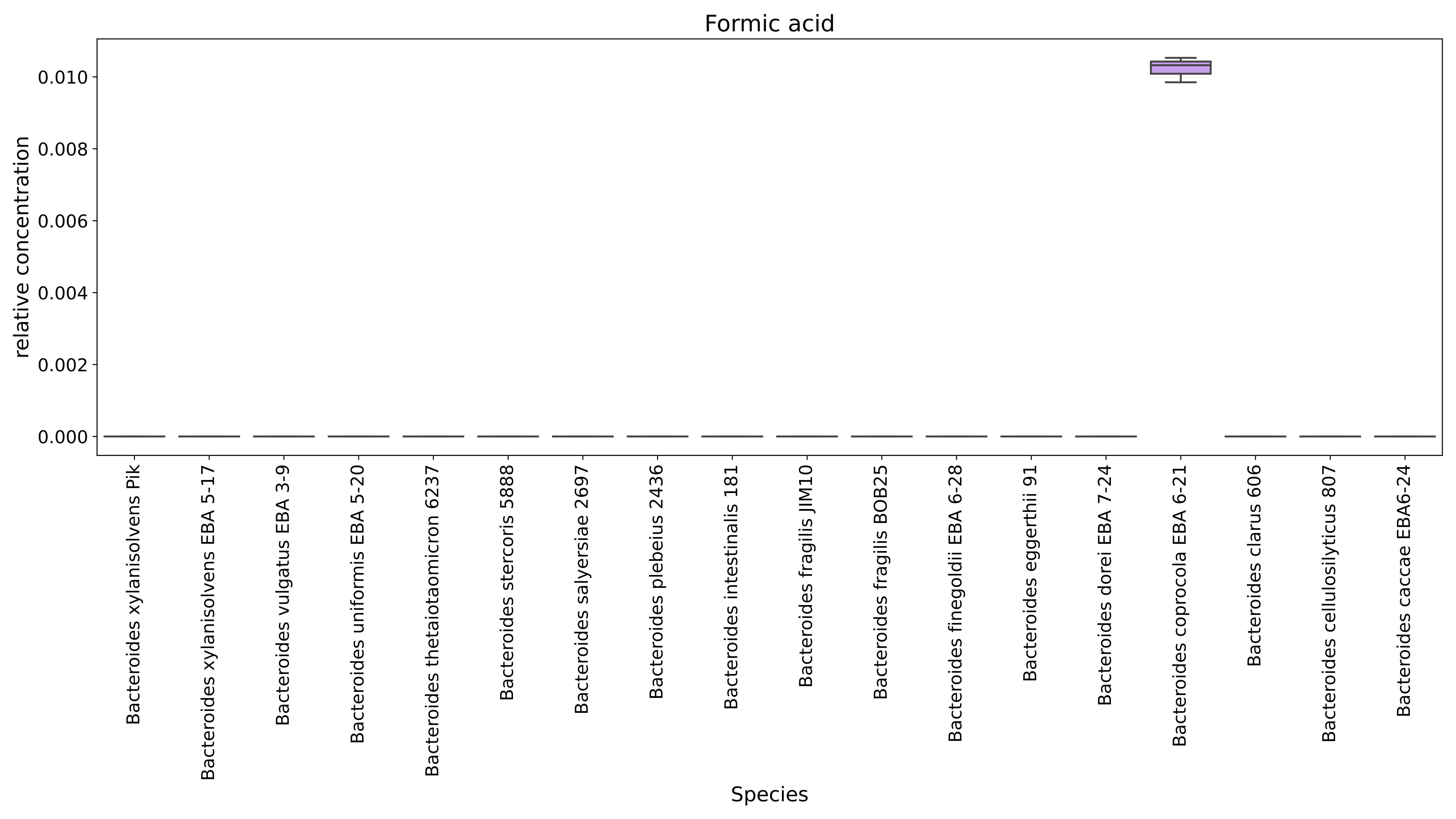
Formic acid
relative concentration
0.010
0.008
0.006
0.004
0.002
0.000
Species
Bacteroides xylanisolvens Pik
Bacteroides xylanisolvens EBA 5-17
Bacteroides vulgatus EBA 3-9
Bacteroides uniformis EBA 5-20
Bacteroides thetaiotaomicron 6237
Bacteroides stercoris 5888
Bacteroides salyersiae 2697
Bacteroides plebeius 2436
Bacteroides intestinalis 181
Bacteroides fragilis JIM10
Bacteroides fragilis BOB25
Bacteroides finegoldii EBA 6-28
Bacteroides eggerthii 91
Bacteroides dorei EBA 7-24
Bacteroides coprocola EBA 6-21
Bacteroides clarus 606
Bacteroides cellulosilyticus 807
Bacteroides caccae EBA6-24

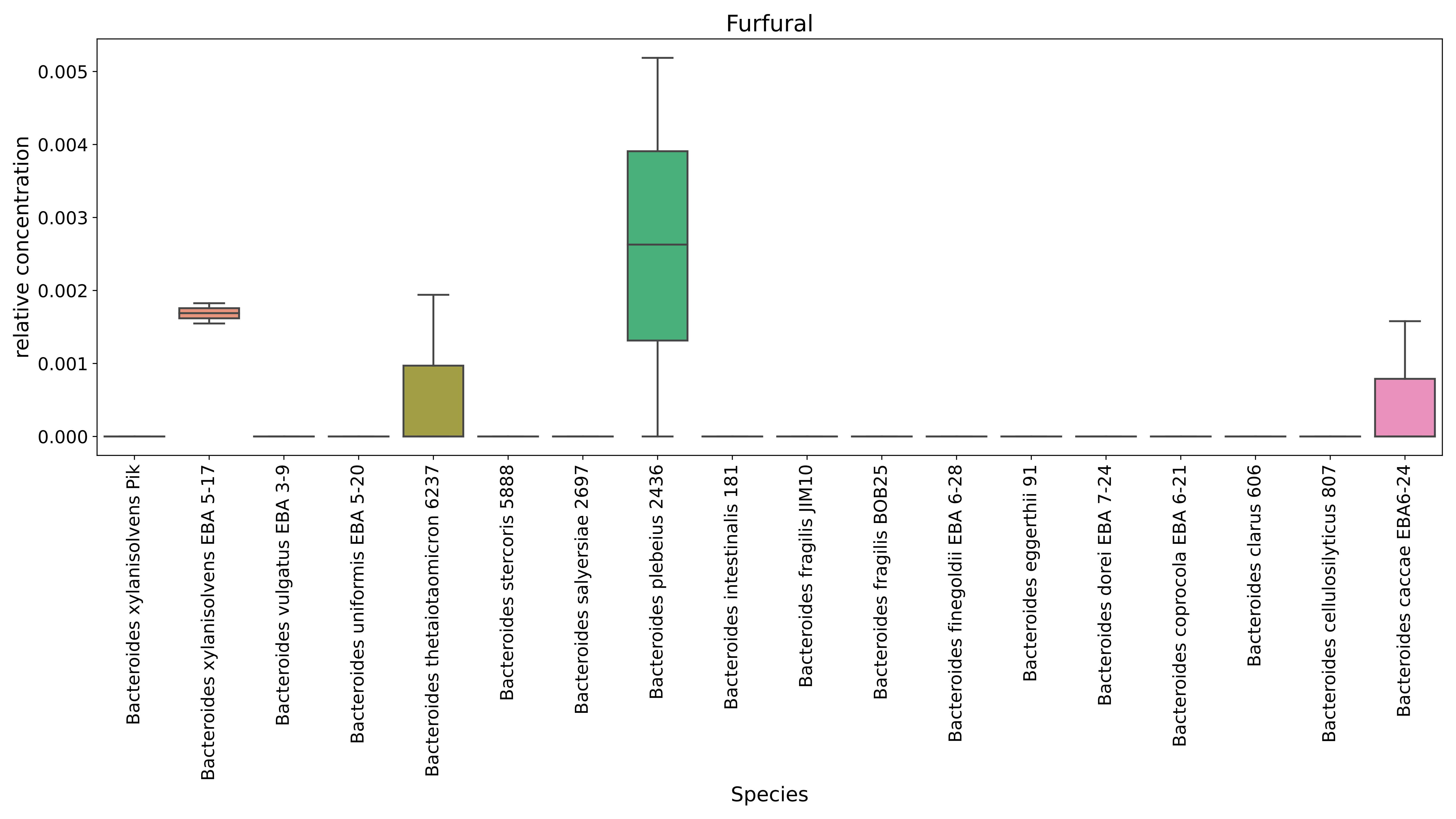
Furfural
relative concentration
0.005
0.004
0.003
0.002
0.001
0.000
Bacteroides xylanisolvens Pik
Bacteroides xylanisolvens EBA 5-17
Bacteroides vulgatus EBA 3-9
Bacteroides uniformis EBA 5-20
Bacteroides thetaiotaomicron 6237
Bacteroides stercoris 5888
Bacteroides salyersiae 2697
Bacteroides plebeius 2436
Bacteroides intestinalis 181
Bacteroides fragilis JIM10
Bacteroides fragilis BOB25
Bacteroides finegoldii EBA 6-28
Bacteroides eggerthii 91
Bacteroides dorei EBA 7-24
Bacteroides coprocola EBA 6-21
Bacteroides clarus 606
Bacteroides cellulosilyticus 807
Bacteroides caccae EBA6-24
Species

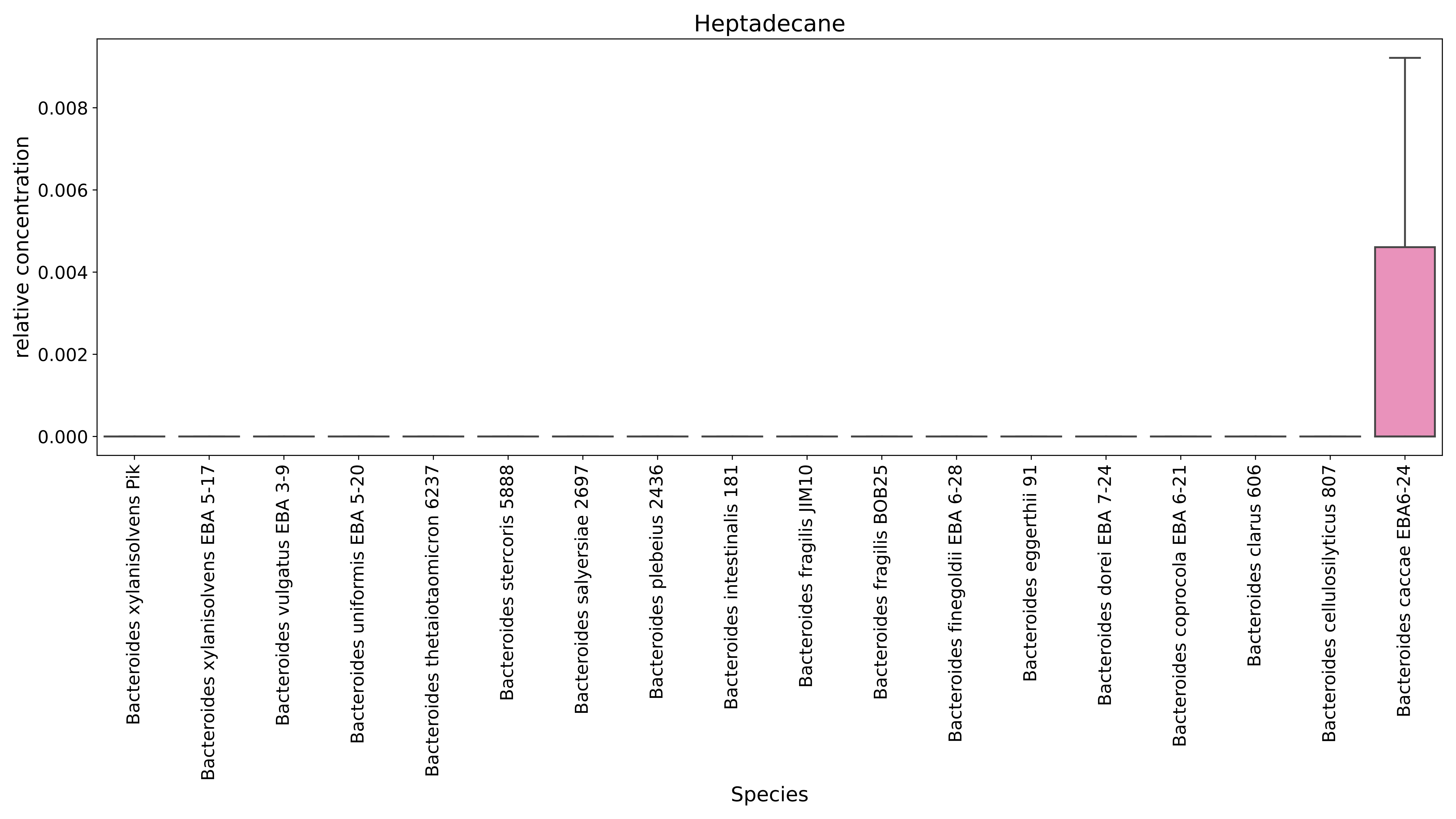
Heptadecane
relative concentration
0.008
0.006
0.004
0.002
0.000
Bacteroides xylanisolvens Pik
Bacteroides xylanisolvens EBA 5-17
Bacteroides vulgatus EBA 3-9
Bacteroides uniformis EBA 5-20
Bacteroides thetaiotaomicron 6237
Bacteroides stercoris 5888
Bacteroides salyersiae 2697
Bacteroides plebeius 2436
Bacteroides intestinalis 181
Bacteroides fragilis JIM10
Bacteroides fragilis BOB25
Bacteroides finegoldii EBA 6-28
Bacteroides eggerthii 91
Bacteroides dorei EBA 7-24
Bacteroides coprocola EBA 6-21
Bacteroides clarus 606
Bacteroides cellulosilyticus 807
Bacteroides caccae EBA6-24
Species

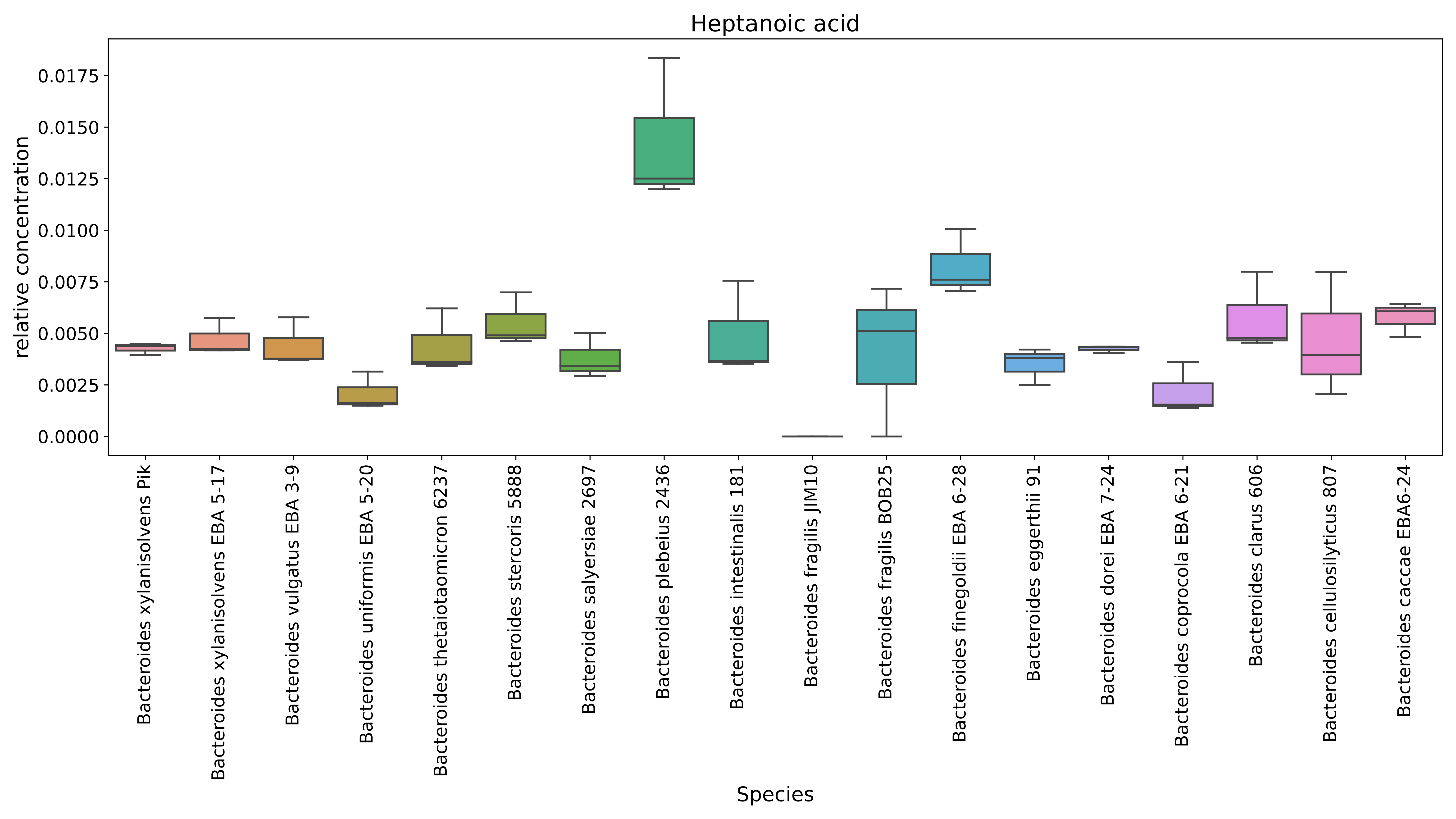
Heptanoic acid
relative concentration
0.0000
0.0025
0.0050
0.0075
0.0100
0.0125
0.0150
0.0175
Bacteroides xylanisolvens Pik
Bacteroides xylanisolvens EBA 5-17
Bacteroides vulgatus EBA 3-9
Bacteroides uniformis EBA 5-20
Bacteroides thetaiotaomicron 6237
Bacteroides stercoris 5888
Bacteroides salyersiae 2697
Bacteroides plebeius 2436
Bacteroides intestinalis 181
Bacteroides fragilis JIM10
Bacteroides fragilis BOB25
Bacteroides finegoldii EBA 6-28
Bacteroides eggerthii 91
Bacteroides dorei EBA 7-24
Bacteroides coprocola EBA 6-21
Bacteroides clarus 606
Bacteroides cellulosilyticus 807
Bacteroides caccae EBA6-24
Species

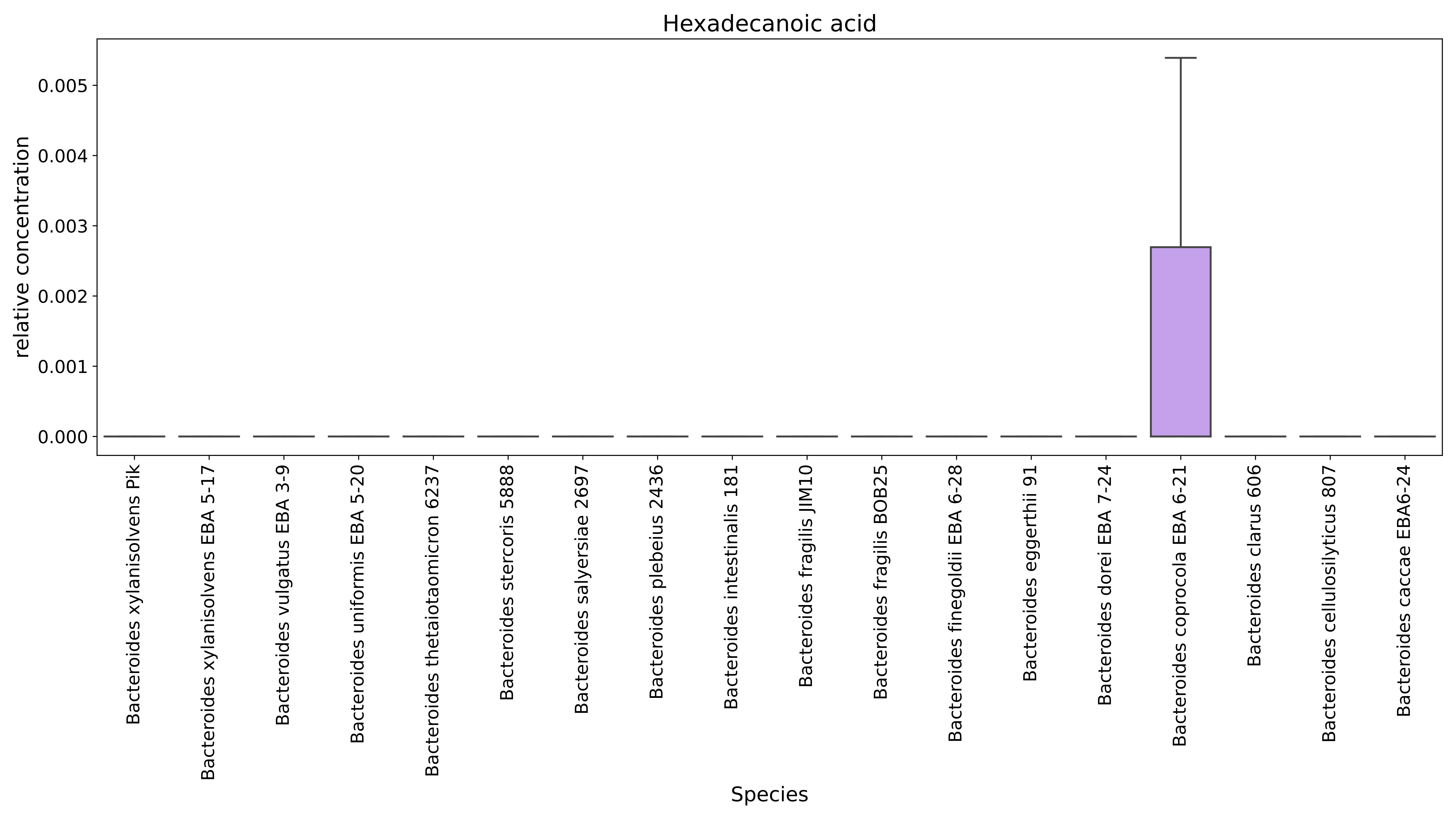

Hexadecanoic acid
relative concentration
0.005
0.004
0.003
0.002
0.001
0.000
Bacteroides xylanisolvens Pik
Bacteroides xylanisolvens EBA 5-17
Bacteroides vulgatus EBA 3-9
Bacteroides uniformis EBA 5-20
Bacteroides thetaiotaomicron 6237
Bacteroides stercoris 5888
Bacteroides salyersiae 2697
Bacteroides plebeius 2436
Bacteroides intestinalis 181
Bacteroides fragilis JIM10
Bacteroides fragilis BOB25
Bacteroides finegoldii EBA 6-28
Bacteroides eggerthii 91
Bacteroides dorei EBA 7-24
Bacteroides coprocola EBA 6-21
Bacteroides clarus 606
Bacteroides cellulosilyticus 807
Bacteroides caccae EBA6-24
Species

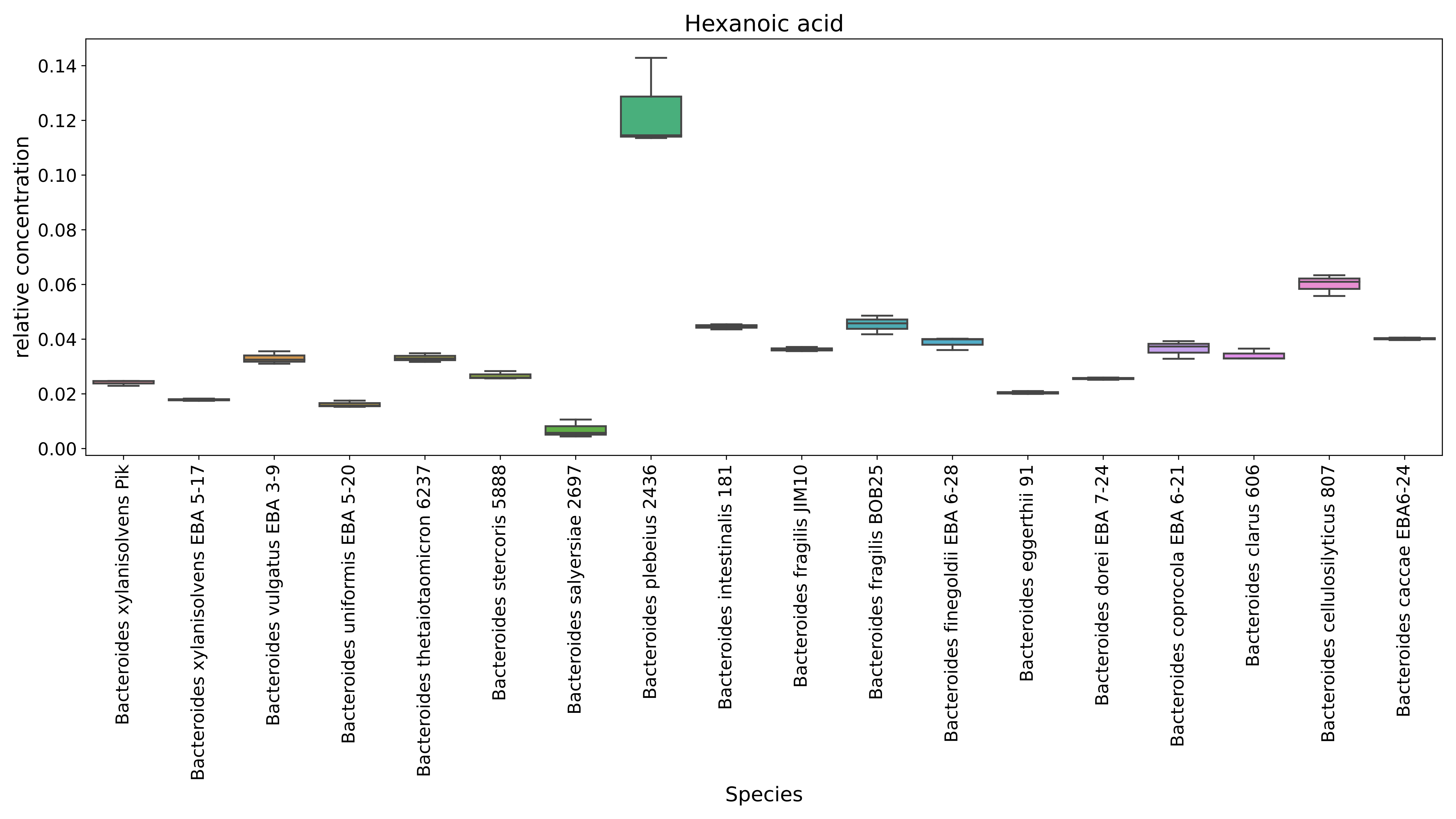
Hexanoic acid
relative concentration
0.00
0.02
0.04
0.06
0.08
0.10
0.12
0.14
Bacteroides xylanisolvens Pik
Bacteroides xylanisolvens EBA 5-17
Bacteroides vulgatus EBA 3-9
Bacteroides uniformis EBA 5-20
Bacteroides thetaiotaomicron 6237
Bacteroides stercoris 5888
Bacteroides salyersiae 2697
Bacteroides plebeius 2436
Bacteroides intestinalis 181
Bacteroides fragilis JIM10
Bacteroides fragilis BOB25
Bacteroides finegoldii EBA 6-28
Bacteroides eggerthii 91
Bacteroides dorei EBA 7-24
Bacteroides coprocola EBA 6-21
Bacteroides clarus 606
Bacteroides cellulosilyticus 807
Bacteroides caccae EBA6-24
Species

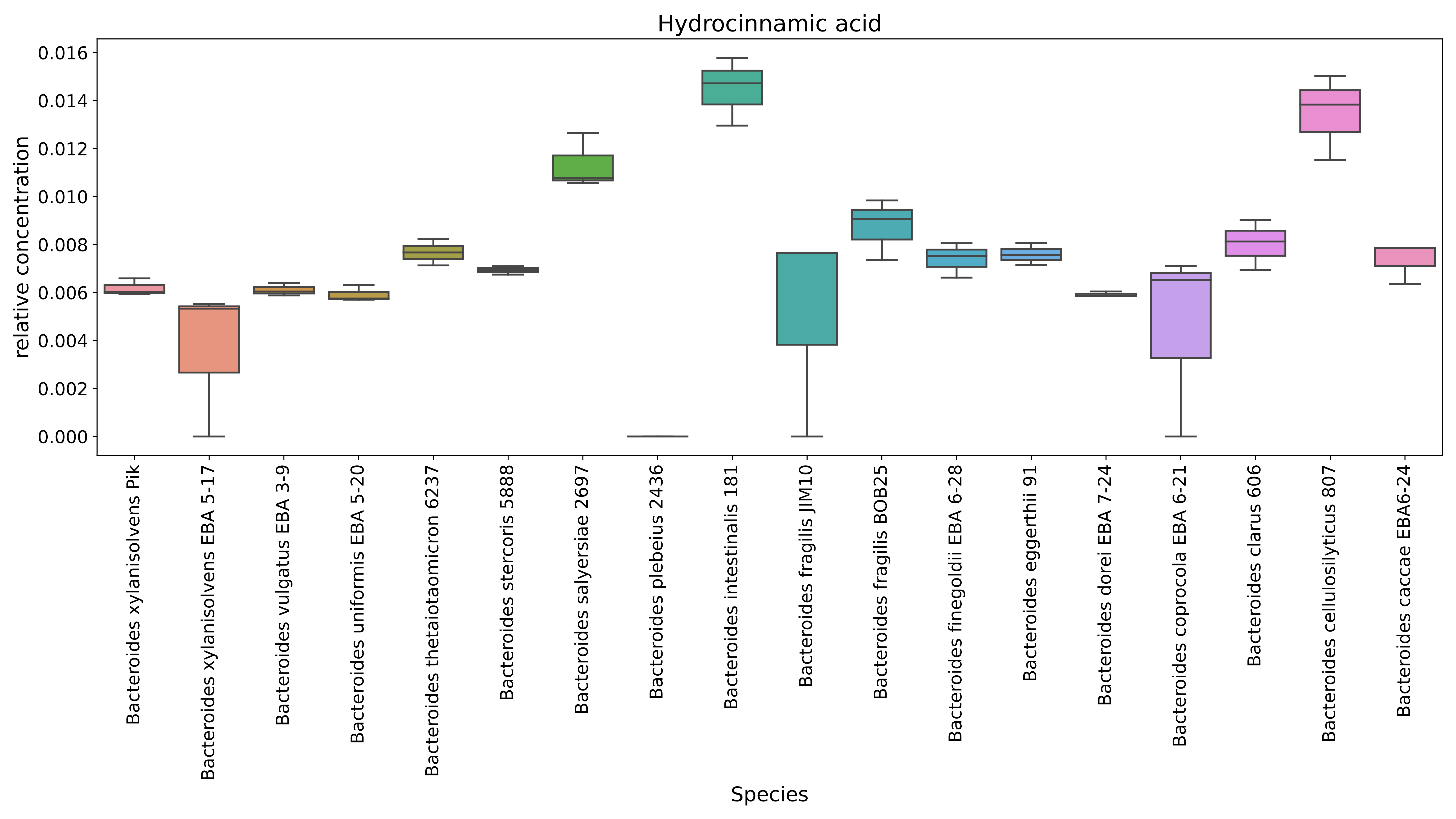

Hydrocinnamic acid
relative concentration
0.016
0.014
0.012
0.010
0.008
0.006
0.004
0.002
0.000
Bacteroides xylanisolvens Pik
Bacteroides xylanisolvens EBA 5-17
Bacteroides vulgatus EBA 3-9
Bacteroides uniformis EBA 5-20
Bacteroides thetaiotaomicron 6237
Bacteroides stercoris 5888
Bacteroides salyersiae 2697
Bacteroides plebeius 2436
Bacteroides intestinalis 181
Bacteroides fragilis JIM10
Bacteroides fragilis BOB25
Bacteroides finegoldii EBA 6-28
Bacteroides eggerthii 91
Bacteroides dorei EBA 7-24
Bacteroides coprocola EBA 6-21
Bacteroides clarus 606
Bacteroides cellulosilyticus 807
Bacteroides caccae EBA6-24
Species

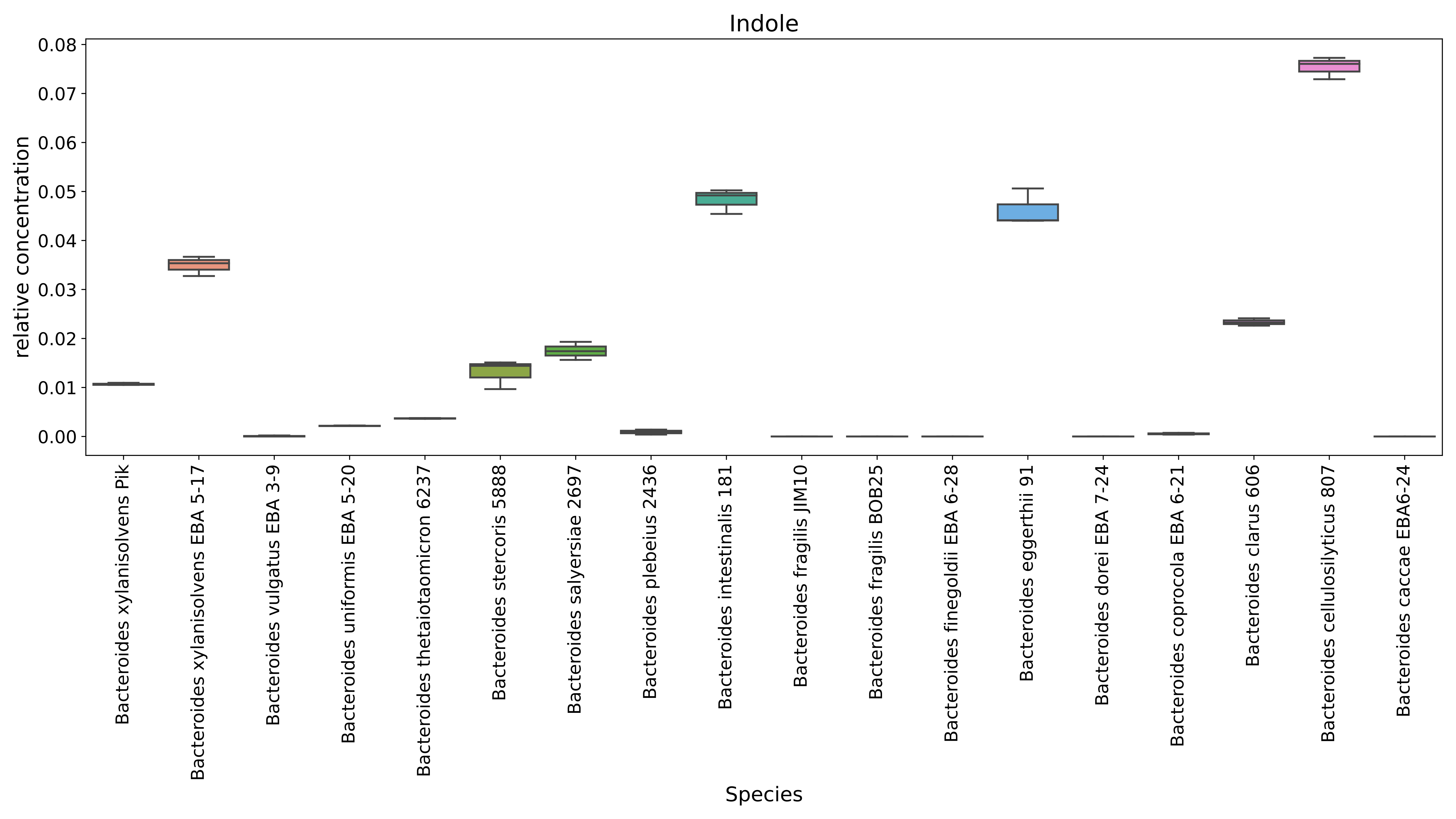
Indole
relative concentration
0.00
0.01
0.02
0.03
0.04
0.05
0.06
0.07
0.08
Species
Bacteroides xylanisolvens Pik
Bacteroides xylanisolvens EBA 5-17
Bacteroides vulgatus EBA 3-9
Bacteroides uniformis EBA 5-20
Bacteroides thetaiotaomicron 6237
Bacteroides stercoris 5888
Bacteroides salyersiae 2697
Bacteroides plebeius 2436
Bacteroides intestinalis 181
Bacteroides fragilis JIM10
Bacteroides fragilis BOB25
Bacteroides finegoldii EBA 6-28
Bacteroides eggerthii 91
Bacteroides dorei EBA 7-24
Bacteroides coprocola EBA 6-21
Bacteroides clarus 606
Bacteroides cellulosilyticus 807
Bacteroides caccae EBA6-24

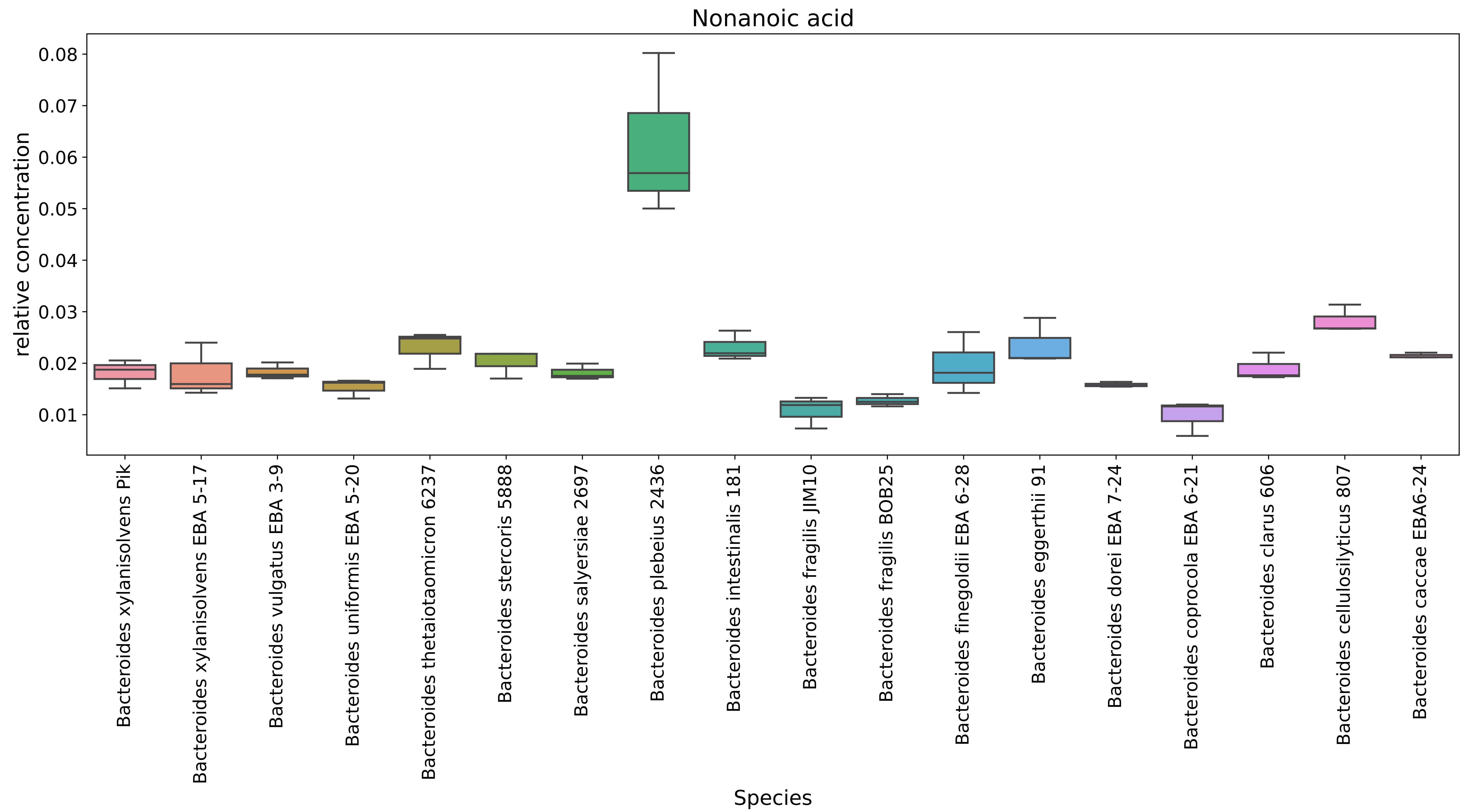
Nonanoic acid
relative concentration
0.08
0.07
0.06
0.05
0.04
0.03
0.02
0.01
Bacteroides xylanisolvens Pik
Bacteroides xylanisolvens EBA 5-17
Bacteroides vulgatus EBA 3-9
Bacteroides uniformis EBA 5-20
Bacteroides thetaiotaomicron 6237
Bacteroides stercoris 5888
Bacteroides salyersiae 2697
Bacteroides plebeius 2436
Bacteroides intestinalis 181
Bacteroides fragilis JIM10
Bacteroides fragilis BOB25
Bacteroides finegoldii EBA 6-28
Bacteroides eggerthii 91
Bacteroides dorei EBA 7-24
Bacteroides coprocola EBA 6-21
Bacteroides clarus 606
Bacteroides cellulosilyticus 807
Bacteroides caccae EBA6-24
Species

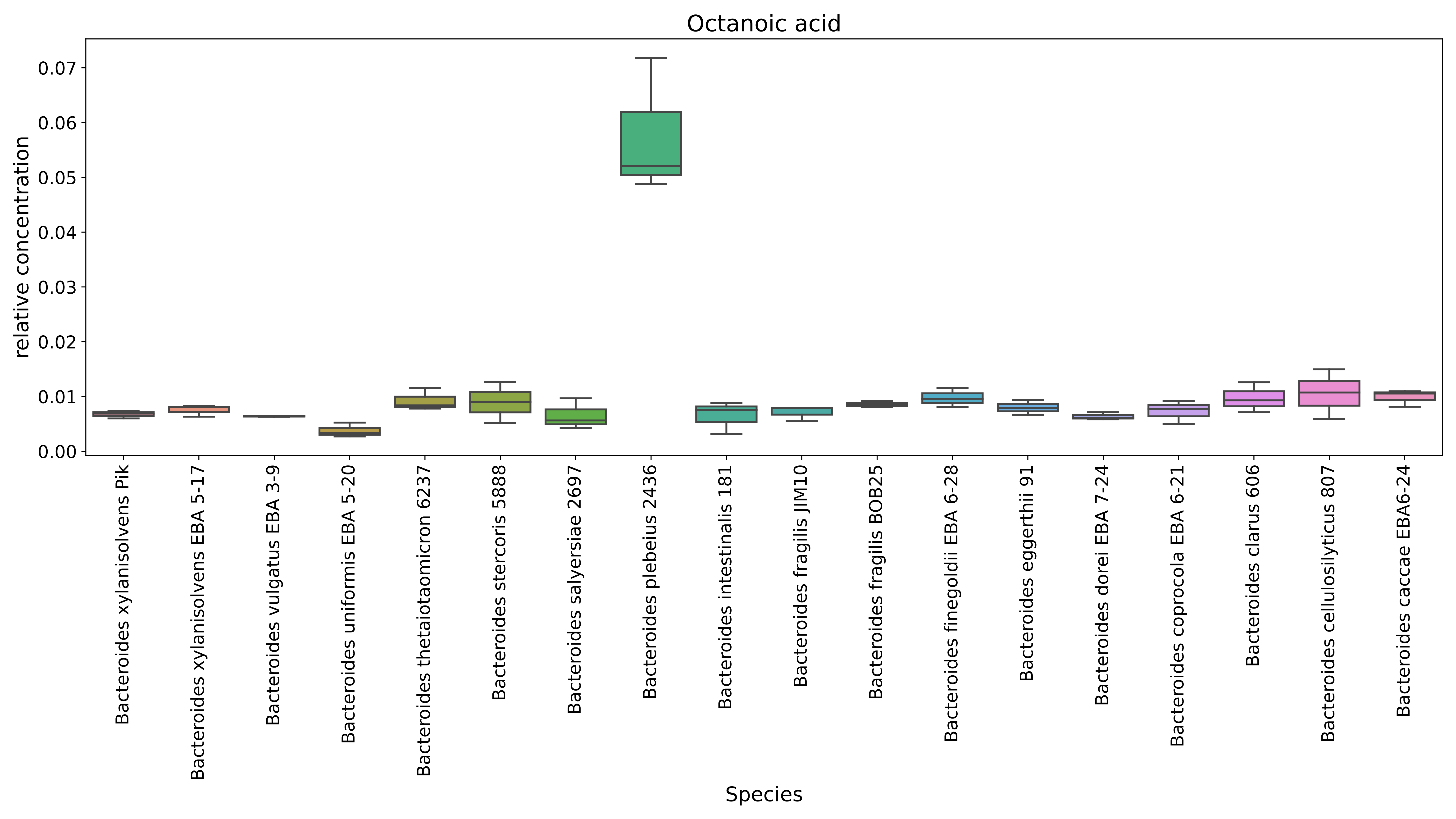
Octanoic acid
relative concentration
0.07
0.06
0.05
0.04
0.03
0.02
0.01
0.00
Bacteroides xylanisolvens Pik
Bacteroides xylanisolvens EBA 5-17
Bacteroides vulgatus EBA 3-9
Bacteroides uniformis EBA 5-20
Bacteroides thetaiotaomicron 6237
Bacteroides stercoris 5888
Bacteroides salyersiae 2697
Bacteroides plebeius 2436
Bacteroides intestinalis 181
Bacteroides fragilis JIM10
Bacteroides fragilis BOB25
Bacteroides finegoldii EBA 6-28
Bacteroides eggerthii 91
Bacteroides dorei EBA 7-24
Bacteroides coprocola EBA 6-21
Bacteroides clarus 606
Bacteroides cellulosilyticus 807
Bacteroides caccae EBA6-24
Species

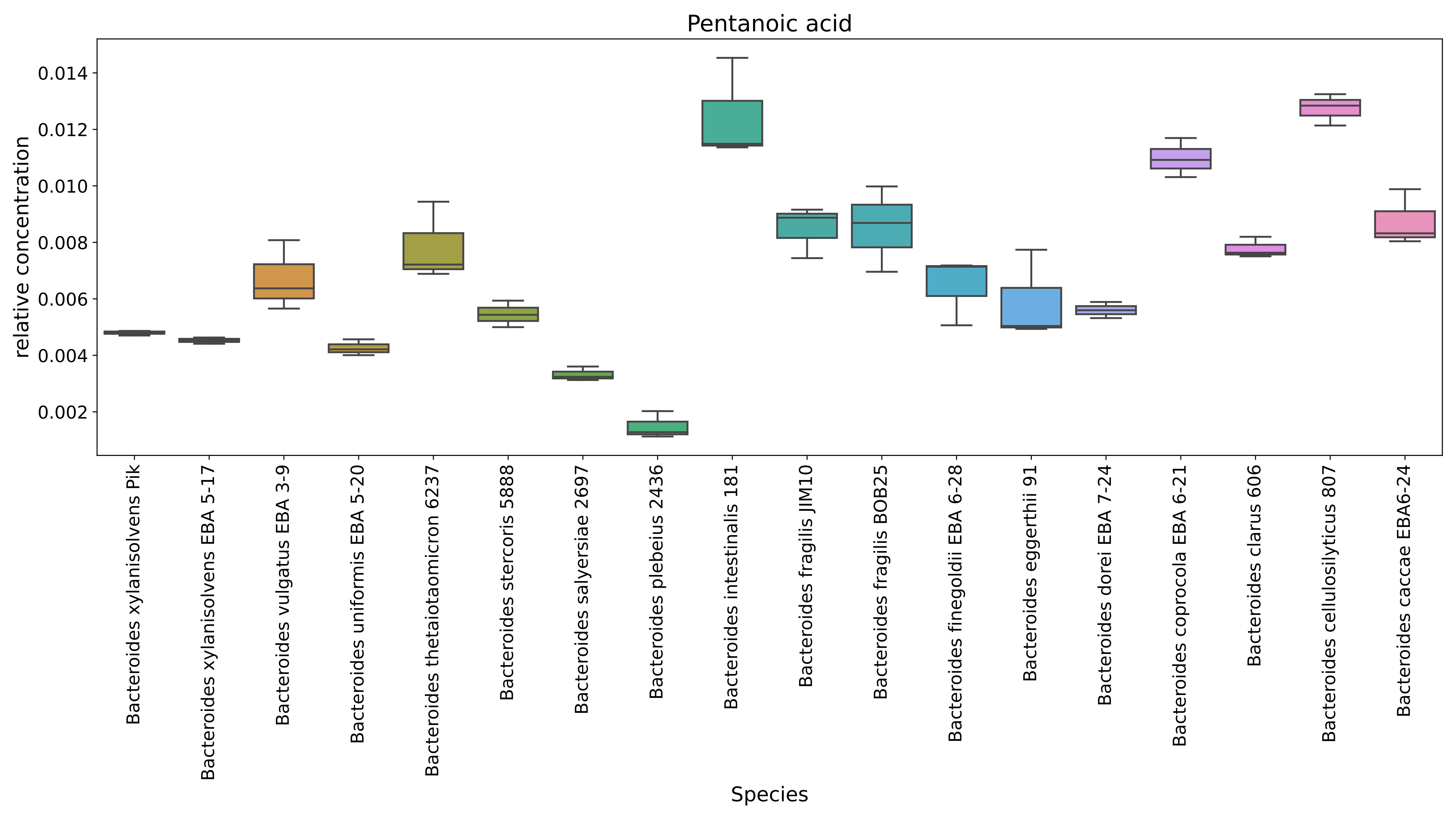
Pentanoic acid
relative concentration
0.014
0.012
0.010
0.008
0.006
0.004
0.002
Bacteroides xylanisolvens Pik
Bacteroides xylanisolvens EBA 5-17
Bacteroides vulgatus EBA 3-9
Bacteroides uniformis EBA 5-20
Bacteroides thetaiotaomicron 6237
Bacteroides stercoris 5888
Bacteroides salyersiae 2697
Bacteroides plebeius 2436
Bacteroides intestinalis 181
Bacteroides fragilis JIM10
Bacteroides fragilis BOB25
Bacteroides finegoldii EBA 6-28
Bacteroides eggerthii 91
Bacteroides dorei EBA 7-24
Bacteroides coprocola EBA 6-21
Bacteroides clarus 606
Bacteroides cellulosilyticus 807
Bacteroides caccae EBA6-24
Species

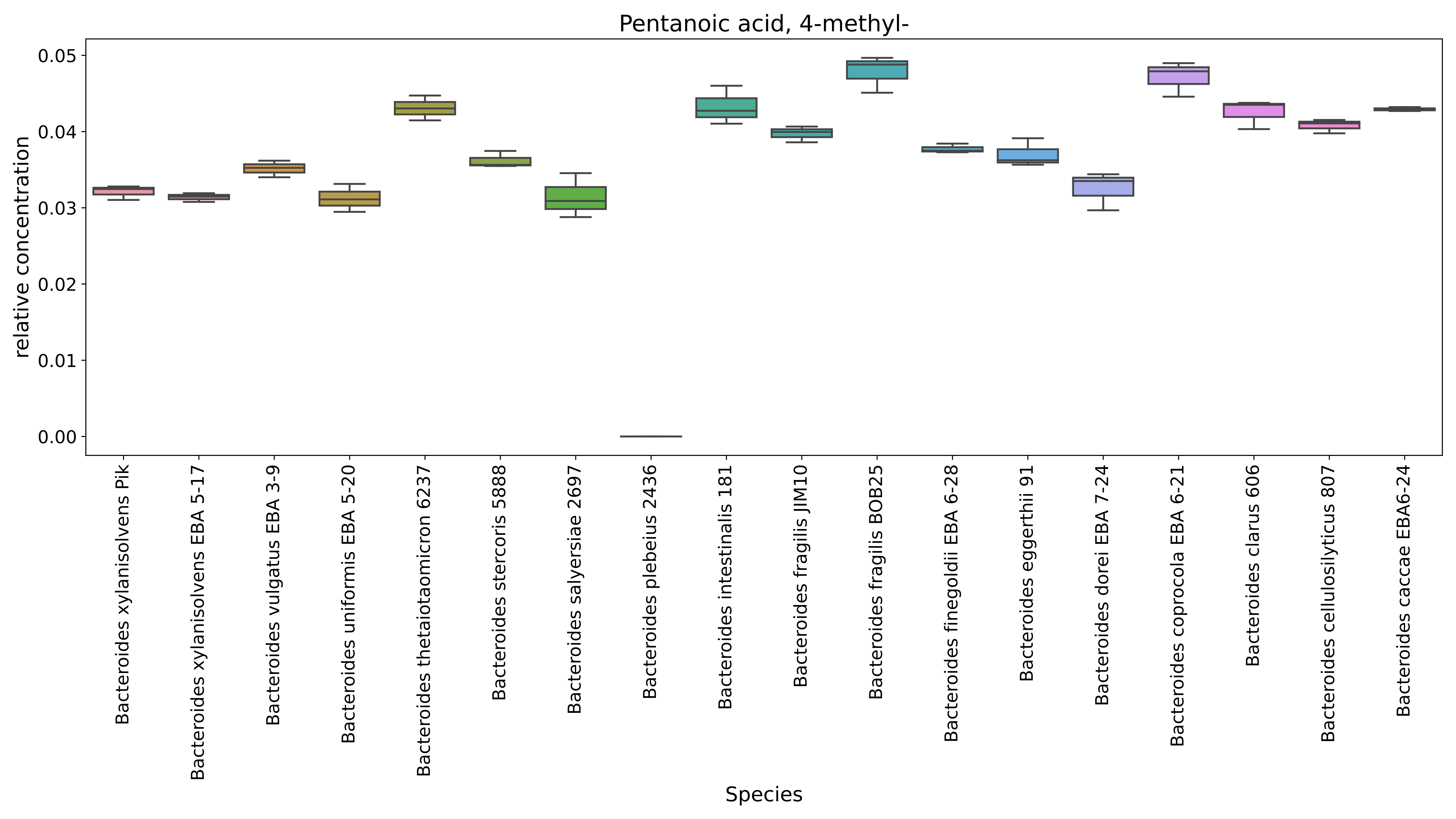
Pentanoic acid, 4-methyl-
relative concentration
0.05
0.04
0.03
0.02
0.01
0.00
Bacteroides xylanisolvens Pik
Bacteroides xylanisolvens EBA 5-17
Bacteroides vulgatus EBA 3-9
Bacteroides uniformis EBA 5-20
Bacteroides thetaiotaomicron 6237
Bacteroides stercoris 5888
Bacteroides salyersiae 2697
Bacteroides plebeius 2436
Bacteroides intestinalis 181
Bacteroides fragilis JIM10
Bacteroides fragilis BOB25
Bacteroides finegoldii EBA 6-28
Bacteroides eggerthii 91
Bacteroides dorei EBA 7-24
Bacteroides coprocola EBA 6-21
Bacteroides clarus 606
Bacteroides cellulosilyticus 807
Bacteroides caccae EBA6-24
Species

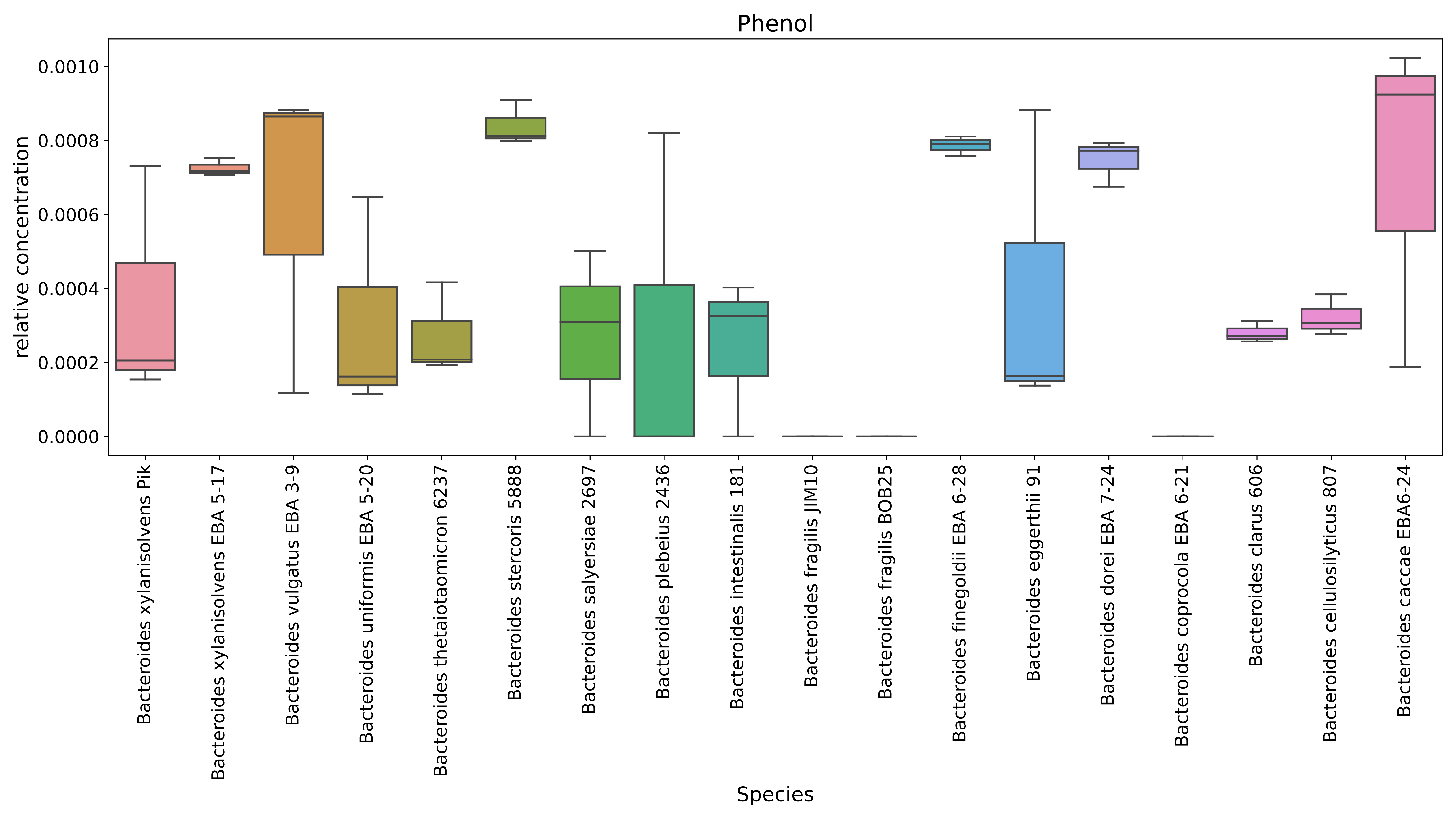
Phenol
relative concentration
0.0010
0.0008
0.0006
0.0004
0.0002
0.0000
Bacteroides xylanisolvens Pik
Bacteroides xylanisolvens EBA 5-17
Bacteroides vulgatus EBA 3-9
Bacteroides uniformis EBA 5-20
Bacteroides thetaiotaomicron 6237
Bacteroides stercoris 5888
Bacteroides salyersiae 2697
Bacteroides plebeius 2436
Bacteroides intestinalis 181
Bacteroides fragilis JIM10
Bacteroides fragilis BOB25
Bacteroides finegoldii EBA 6-28
Bacteroides eggerthii 91
Bacteroides dorei EBA 7-24
Bacteroides coprocola EBA 6-21
Bacteroides clarus 606
Bacteroides cellulosilyticus 807
Bacteroides caccae EBA6-24
Species

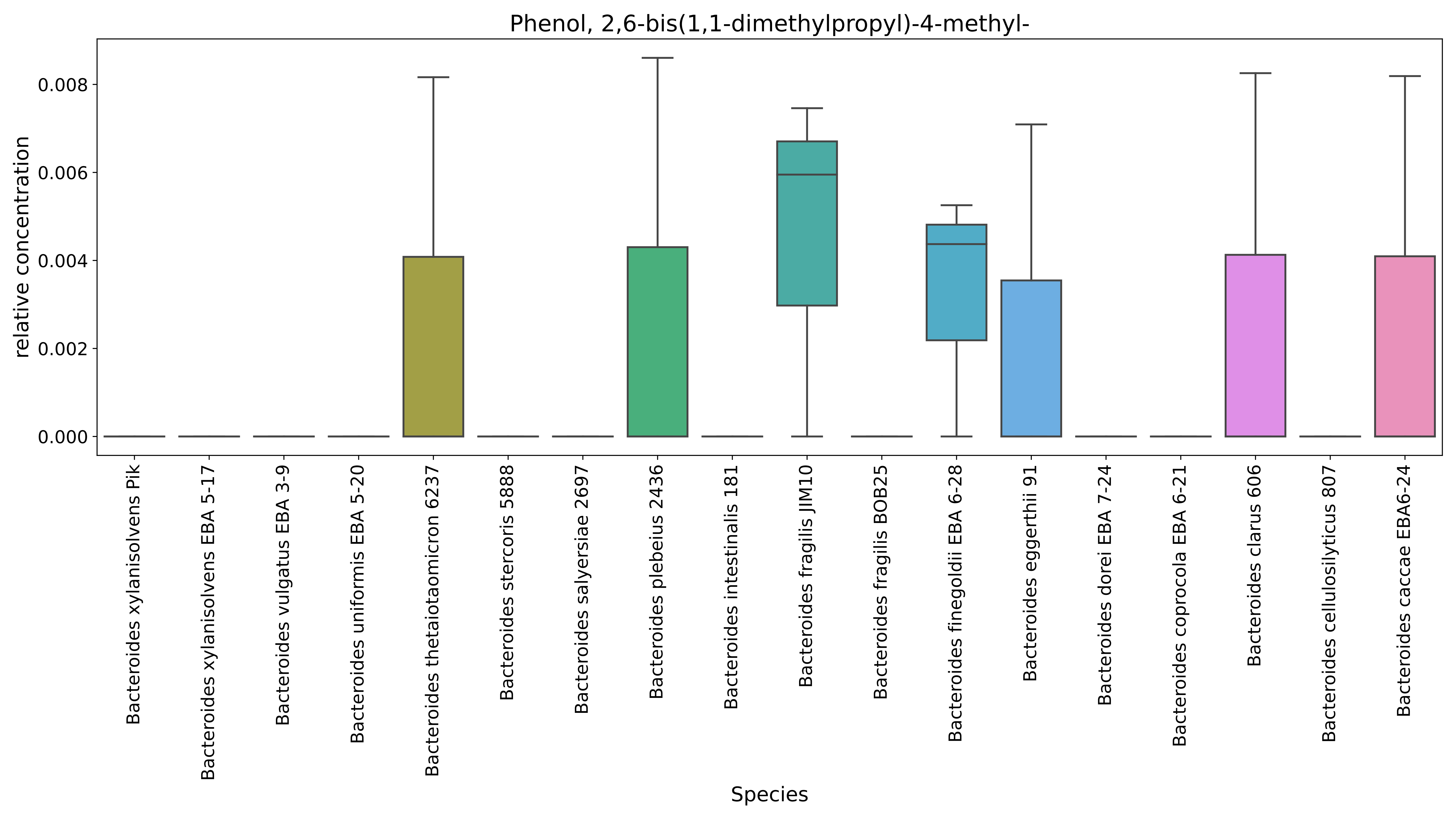
Phenol, 2,6-bis(1,1-dimethylpropyl)-4-methyl-
relative concentration
0.000
0.002
0.004
0.006
0.008
Species
Bacteroides xylanisolvens Pik
Bacteroides xylanisolvens EBA 5-17
Bacteroides vulgatus EBA 3-9
Bacteroides uniformis EBA 5-20
Bacteroides thetaiotaomicron 6237
Bacteroides stercoris 5888
Bacteroides salyersiae 2697
Bacteroides plebeius 2436
Bacteroides intestinalis 181
Bacteroides fragilis JIM10
Bacteroides fragilis BOB25
Bacteroides finegoldii EBA 6-28
Bacteroides eggerthii 91
Bacteroides dorei EBA 7-24
Bacteroides coprocola EBA 6-21
Bacteroides clarus 606
Bacteroides cellulosilyticus 807
Bacteroides caccae EBA6-24

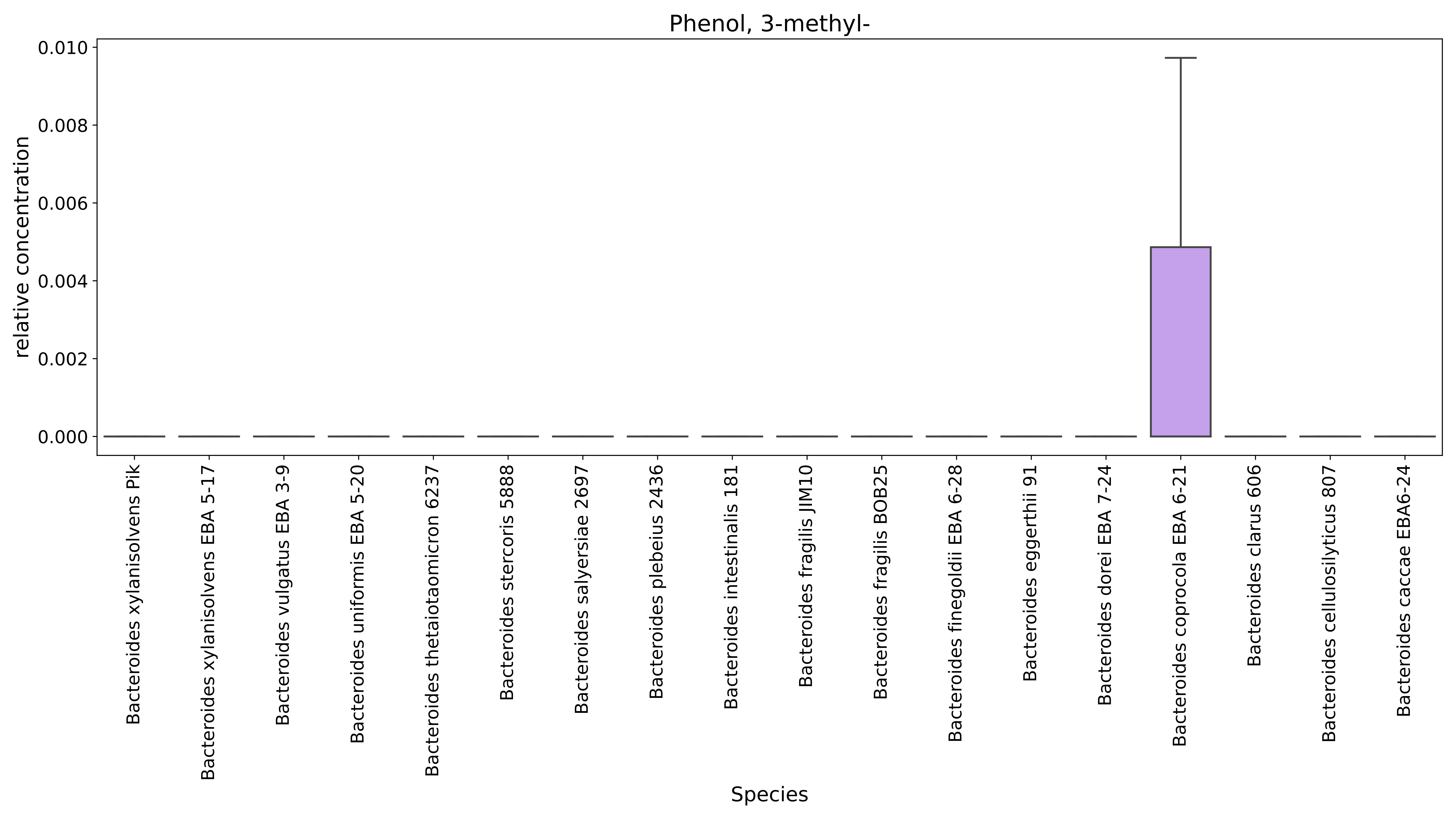
Phenol, 3-methyl-
relative concentration
0.010
0.008
0.006
0.004
0.002
0.000
Bacteroides xylanisolvens Pik
Bacteroides xylanisolvens EBA 5-17
Bacteroides vulgatus EBA 3-9
Bacteroides uniformis EBA 5-20
Bacteroides thetaiotaomicron 6237
Bacteroides stercoris 5888
Bacteroides salyersiae 2697
Bacteroides plebeius 2436
Bacteroides intestinalis 181
Bacteroides fragilis JIM10
Bacteroides fragilis BOB25
Bacteroides finegoldii EBA 6-28
Bacteroides eggerthii 91
Bacteroides dorei EBA 7-24
Bacteroides coprocola EBA 6-21
Bacteroides clarus 606
Bacteroides cellulosilyticus 807
Bacteroides caccae EBA6-24
Species

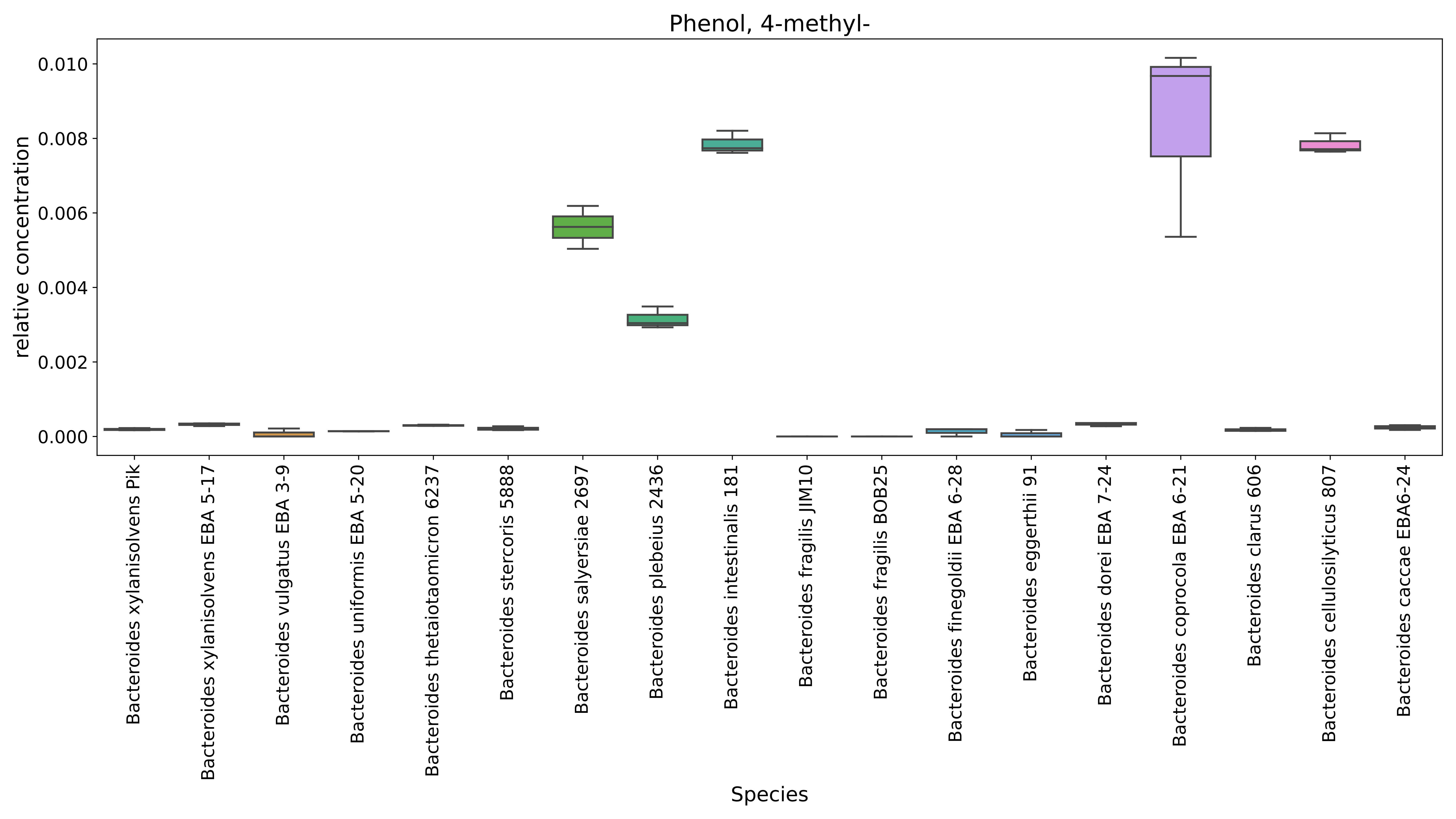

Phenol, 4-methyl-
relative concentration
0.010
0.008
0.006
0.004
0.002
0.000
Bacteroides xylanisolvens Pik
Bacteroides xylanisolvens EBA 5-17
Bacteroides vulgatus EBA 3-9
Bacteroides uniformis EBA 5-20
Bacteroides thetaiotaomicron 6237
Bacteroides stercoris 5888
Bacteroides salyersiae 2697
Bacteroides plebeius 2436
Bacteroides intestinalis 181
Bacteroides fragilis JIM10
Bacteroides fragilis BOB25
Bacteroides finegoldii EBA 6-28
Bacteroides eggerthii 91
Bacteroides dorei EBA 7-24
Bacteroides coprocola EBA 6-21
Bacteroides clarus 606
Bacteroides cellulosilyticus 807
Bacteroides caccae EBA6-24
Species

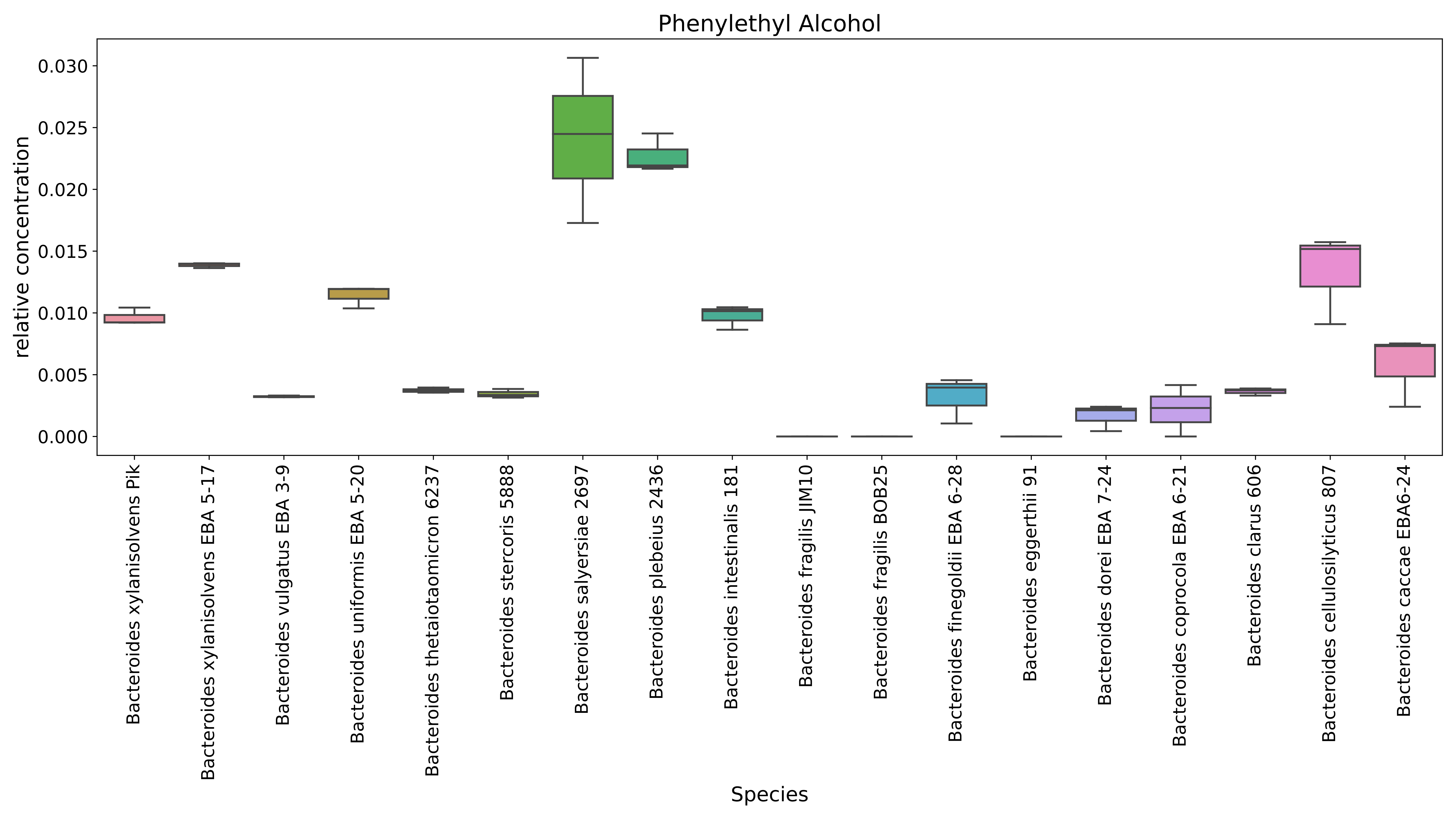
Phenylethyl Alcohol
relative concentration
0.030
0.025
0.020
0.015
0.010
0.005
0.000
Bacteroides xylanisolvens Pik
Bacteroides xylanisolvens EBA 5-17
Bacteroides vulgatus EBA 3-9
Bacteroides uniformis EBA 5-20
Bacteroides thetaiotaomicron 6237
Bacteroides stercoris 5888
Bacteroides salyersiae 2697
Bacteroides plebeius 2436
Bacteroides intestinalis 181
Bacteroides fragilis JIM10
Bacteroides fragilis BOB25
Bacteroides finegoldii EBA 6-28
Bacteroides eggerthii 91
Bacteroides dorei EBA 7-24
Bacteroides coprocola EBA 6-21
Bacteroides clarus 606
Bacteroides cellulosilyticus 807
Bacteroides caccae EBA6-24
Species

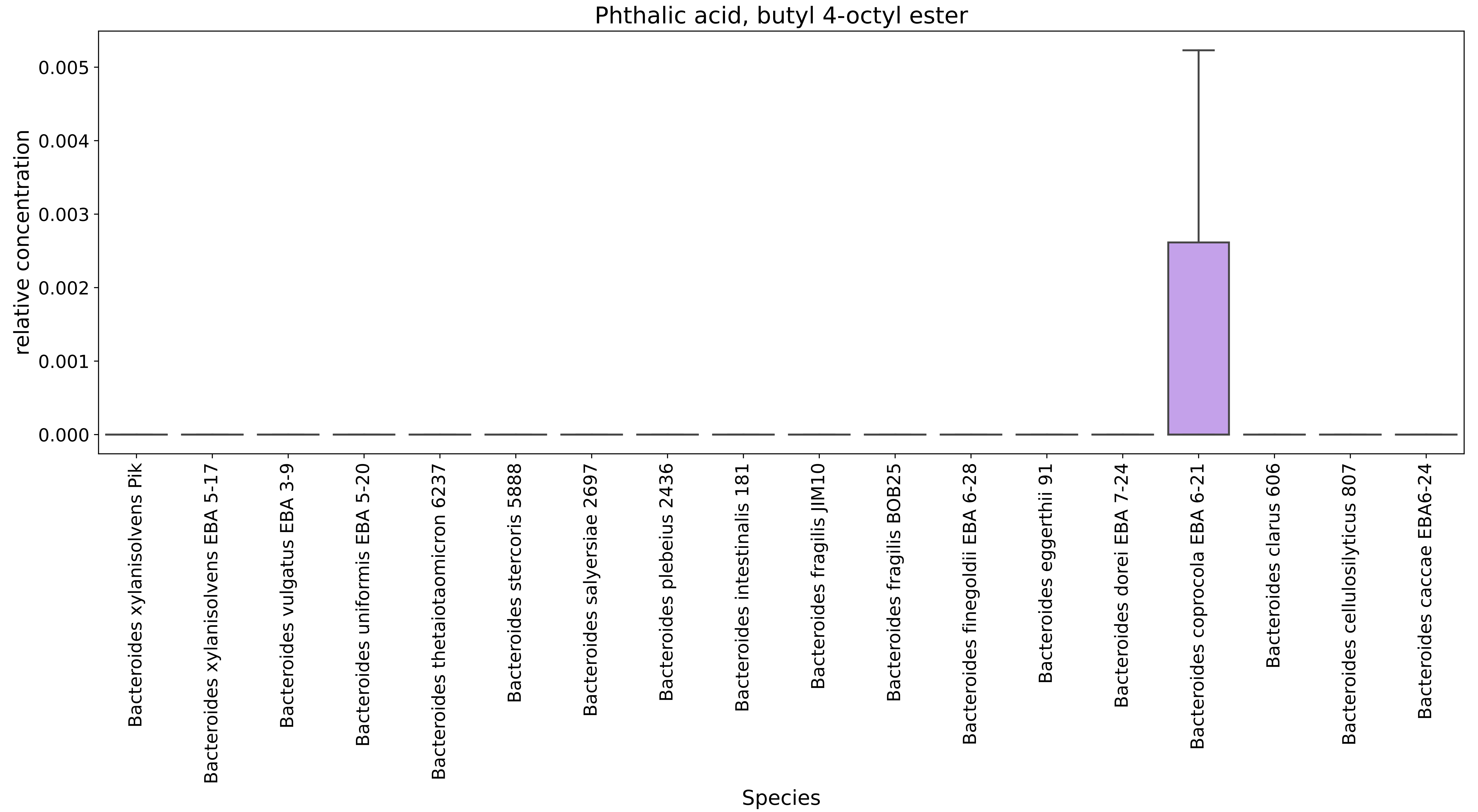
Phthalic acid, butyl 4-octyl ester
relative concentration
0.000
0.001
0.002
0.003
0.004
0.005
Species
Bacteroides xylanisolvens Pik
Bacteroides xylanisolvens EBA 5-17
Bacteroides vulgatus EBA 3-9
Bacteroides uniformis EBA 5-20
Bacteroides thetaiotaomicron 6237
Bacteroides stercoris 5888
Bacteroides salyersiae 2697
Bacteroides plebeius 2436
Bacteroides intestinalis 181
Bacteroides fragilis JIM10
Bacteroides fragilis BOB25
Bacteroides finegoldii EBA 6-28
Bacteroides eggerthii 91
Bacteroides dorei EBA 7-24
Bacteroides coprocola EBA 6-21
Bacteroides clarus 606
Bacteroides cellulosilyticus 807
Bacteroides caccae EBA6-24

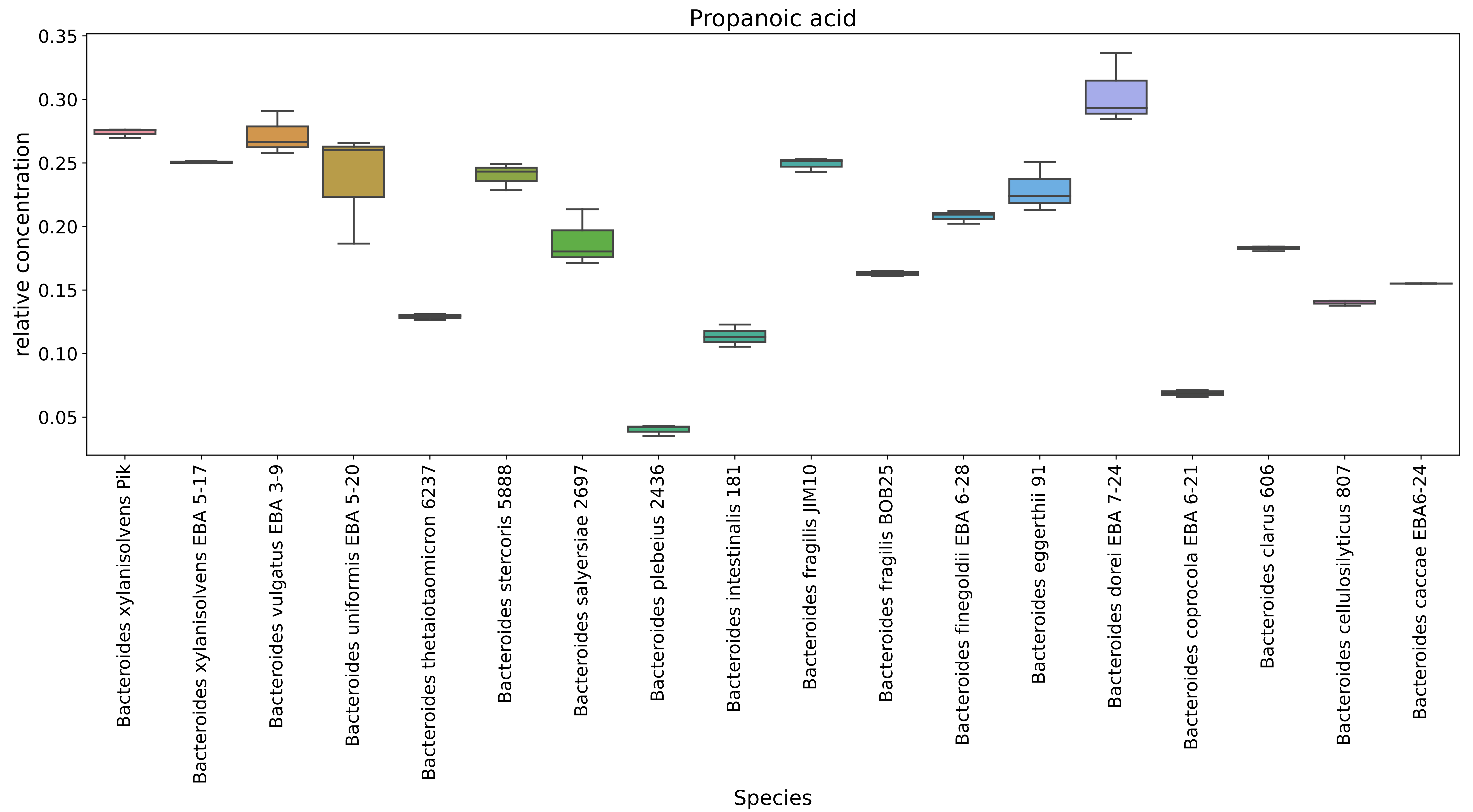
Propanoic acid
relative concentration
0.35
0.30
0.25
0.20
0.15
0.10
0.05
Species
Bacteroides xylanisolvens Pik
Bacteroides xylanisolvens EBA 5-17
Bacteroides vulgatus EBA 3-9
Bacteroides uniformis EBA 5-20
Bacteroides thetaiotaomicron 6237
Bacteroides stercoris 5888
Bacteroides salyersiae 2697
Bacteroides plebeius 2436
Bacteroides intestinalis 181
Bacteroides fragilis JIM10
Bacteroides fragilis BOB25
Bacteroides finegoldii EBA 6-28
Bacteroides eggerthii 91
Bacteroides dorei EBA 7-24
Bacteroides coprocola EBA 6-21
Bacteroides clarus 606
Bacteroides cellulosilyticus 807
Bacteroides caccae EBA6-24

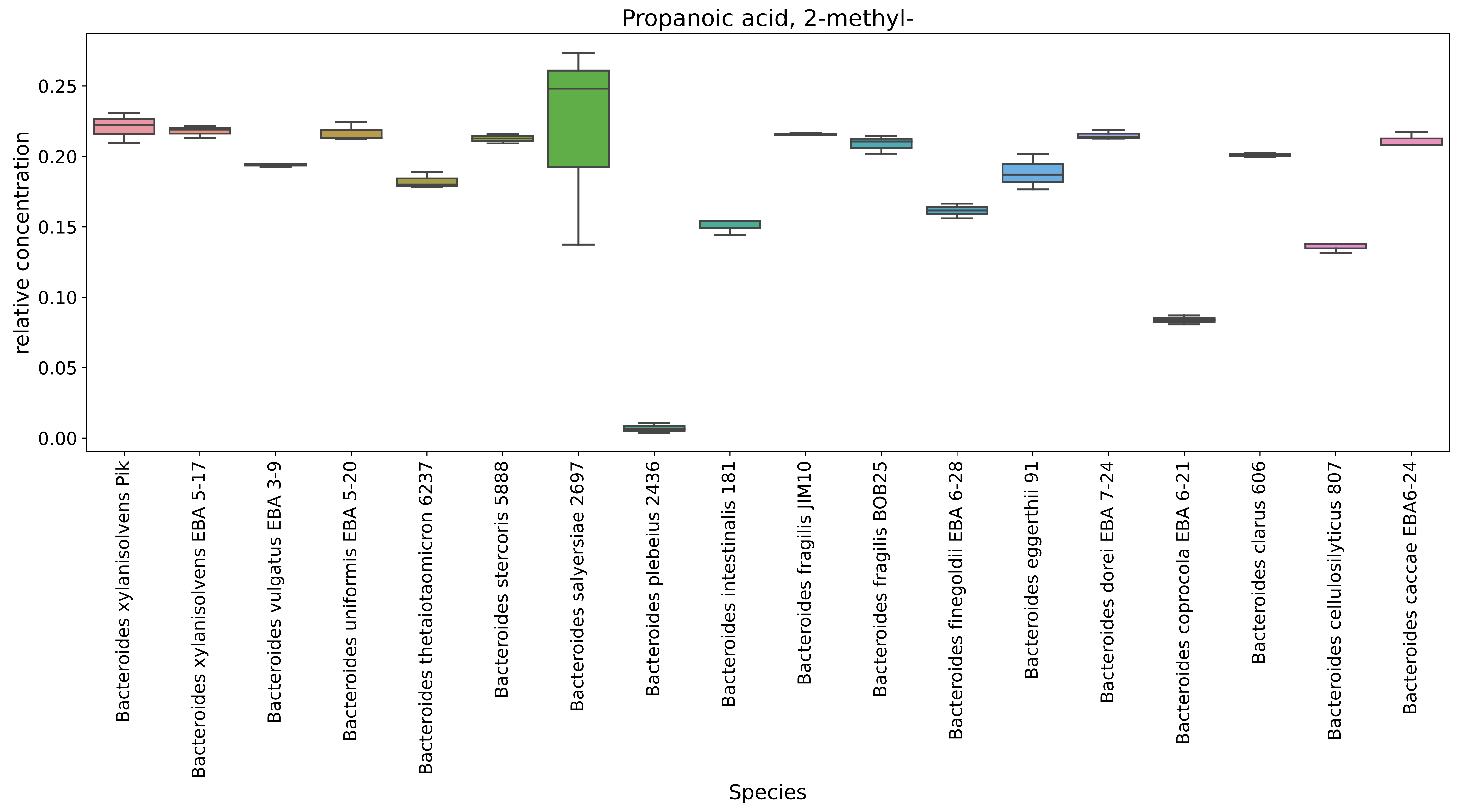
Propanoic acid, 2-methyl-
relative concentration
0.00
0.05
0.10
0.15
0.20
0.25
Species
Bacteroides xylanisolvens Pik
Bacteroides xylanisolvens EBA 5-17
Bacteroides vulgatus EBA 3-9
Bacteroides uniformis EBA 5-20
Bacteroides thetaiotaomicron 6237
Bacteroides stercoris 5888
Bacteroides salyersiae 2697
Bacteroides plebeius 2436
Bacteroides intestinalis 181
Bacteroides fragilis JIM10
Bacteroides fragilis BOB25
Bacteroides finegoldii EBA 6-28
Bacteroides eggerthii 91
Bacteroides dorei EBA 7-24
Bacteroides coprocola EBA 6-21
Bacteroides clarus 606
Bacteroides cellulosilyticus 807
Bacteroides caccae EBA6-24

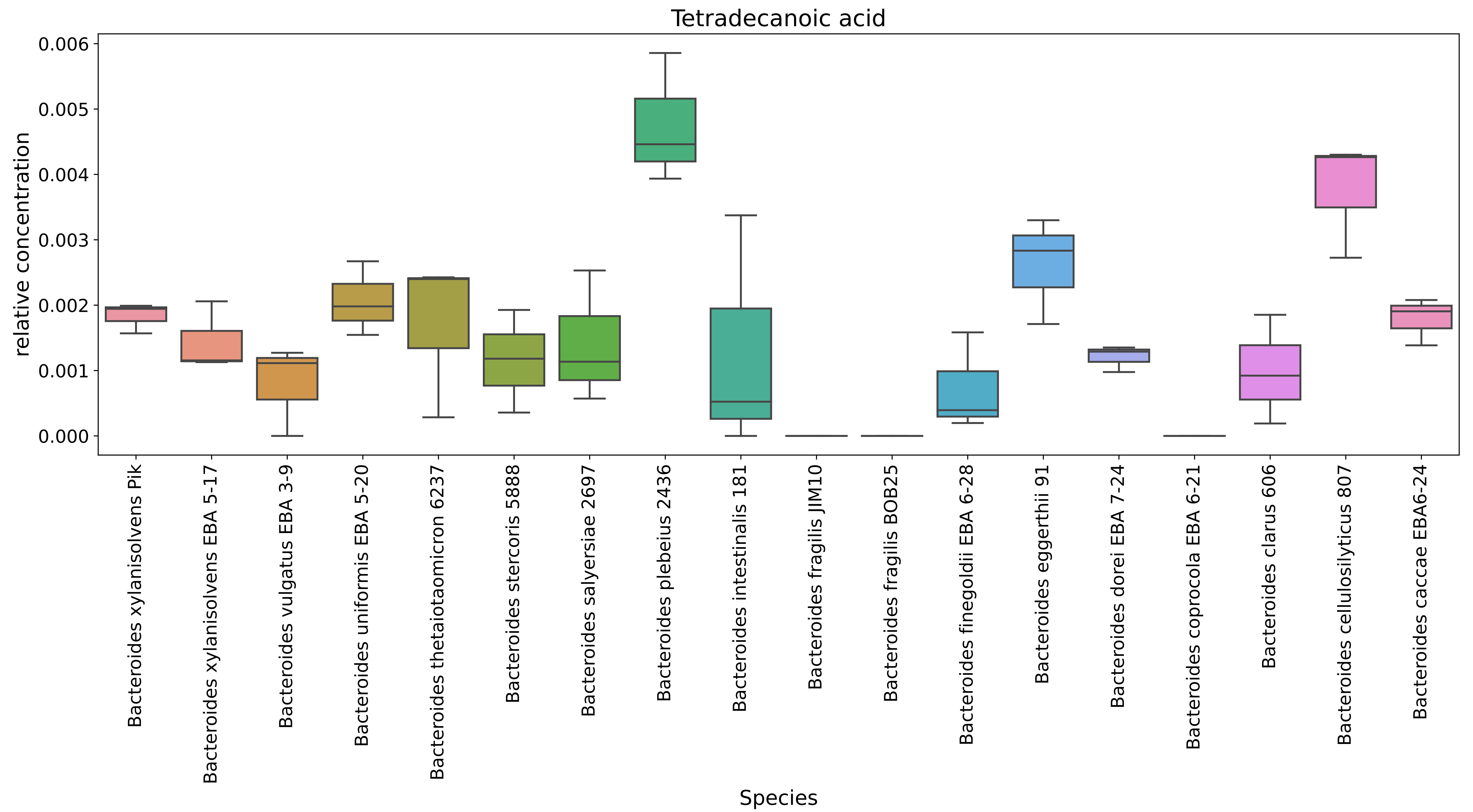
Tetradecanoic acid
relative concentration
0.006
0.005
0.004
0.003
0.002
0.001
0.000
Bacteroides xylanisolvens Pik
Bacteroides xylanisolvens EBA 5-17
Bacteroides vulgatus EBA 3-9
Bacteroides uniformis EBA 5-20
Bacteroides thetaiotaomicron 6237
Bacteroides stercoris 5888
Bacteroides salyersiae 2697
Bacteroides plebeius 2436
Bacteroides intestinalis 181
Bacteroides fragilis JIM10
Bacteroides fragilis BOB25
Bacteroides finegoldii EBA 6-28
Bacteroides eggerthii 91
Bacteroides dorei EBA 7-24
Bacteroides coprocola EBA 6-21
Bacteroides clarus 606
Bacteroides cellulosilyticus 807
Bacteroides caccae EBA6-24
Species

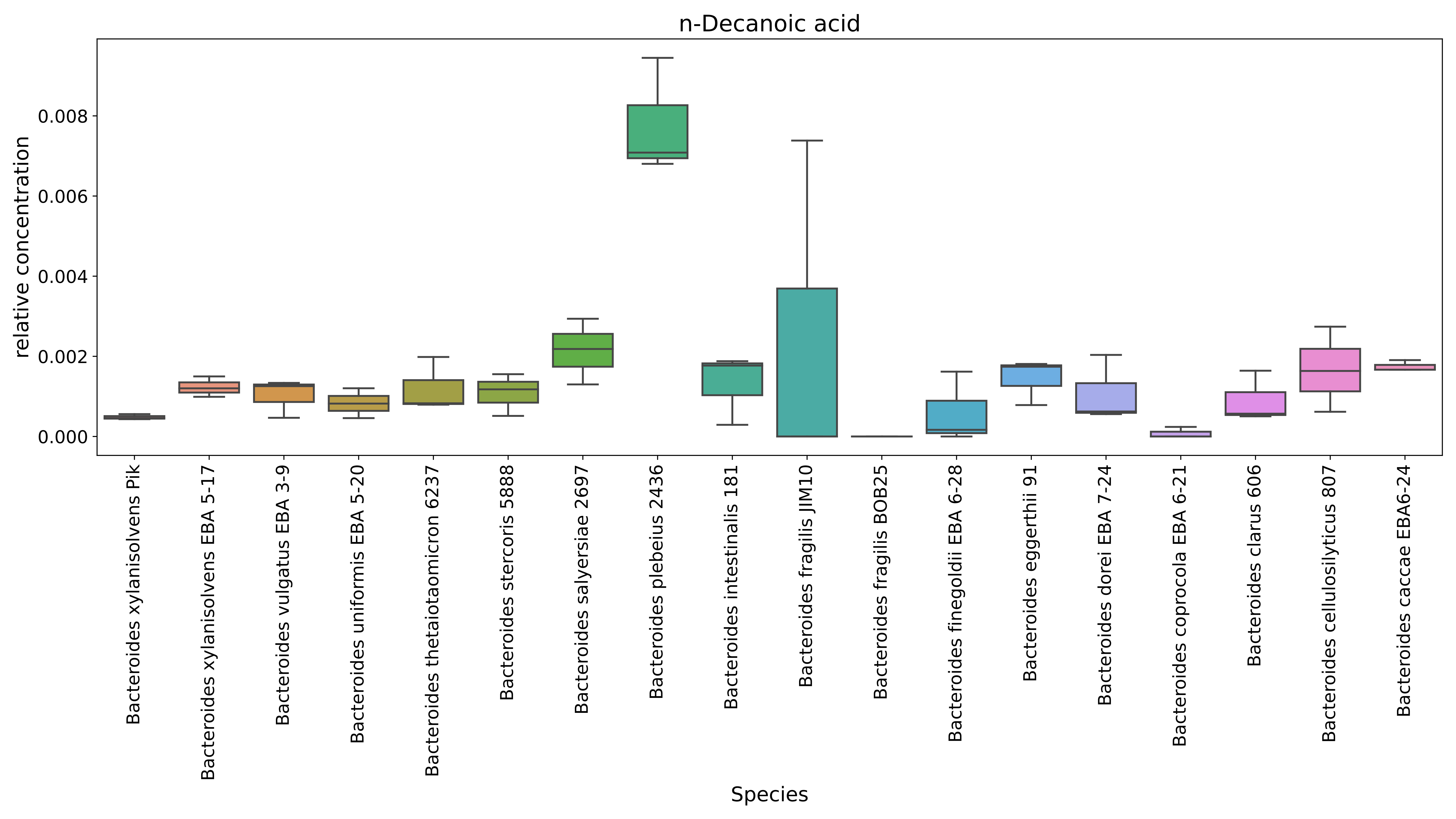
n-Decanoic acid
relative concentration
0.008
0.006
0.004
0.002
0.000
Bacteroides xylanisolvens Pik
Bacteroides xylanisolvens EBA 5-17
Bacteroides vulgatus EBA 3-9
Bacteroides uniformis EBA 5-20
Bacteroides thetaiotaomicron 6237
Bacteroides stercoris 5888
Bacteroides salyersiae 2697
Bacteroides plebeius 2436
Bacteroides intestinalis 181
Bacteroides fragilis JIM10
Bacteroides fragilis BOB25
Bacteroides finegoldii EBA 6-28
Bacteroides eggerthii 91
Bacteroides dorei EBA 7-24
Bacteroides coprocola EBA 6-21
Bacteroides clarus 606
Bacteroides cellulosilyticus 807
Bacteroides caccae EBA6-24
Species

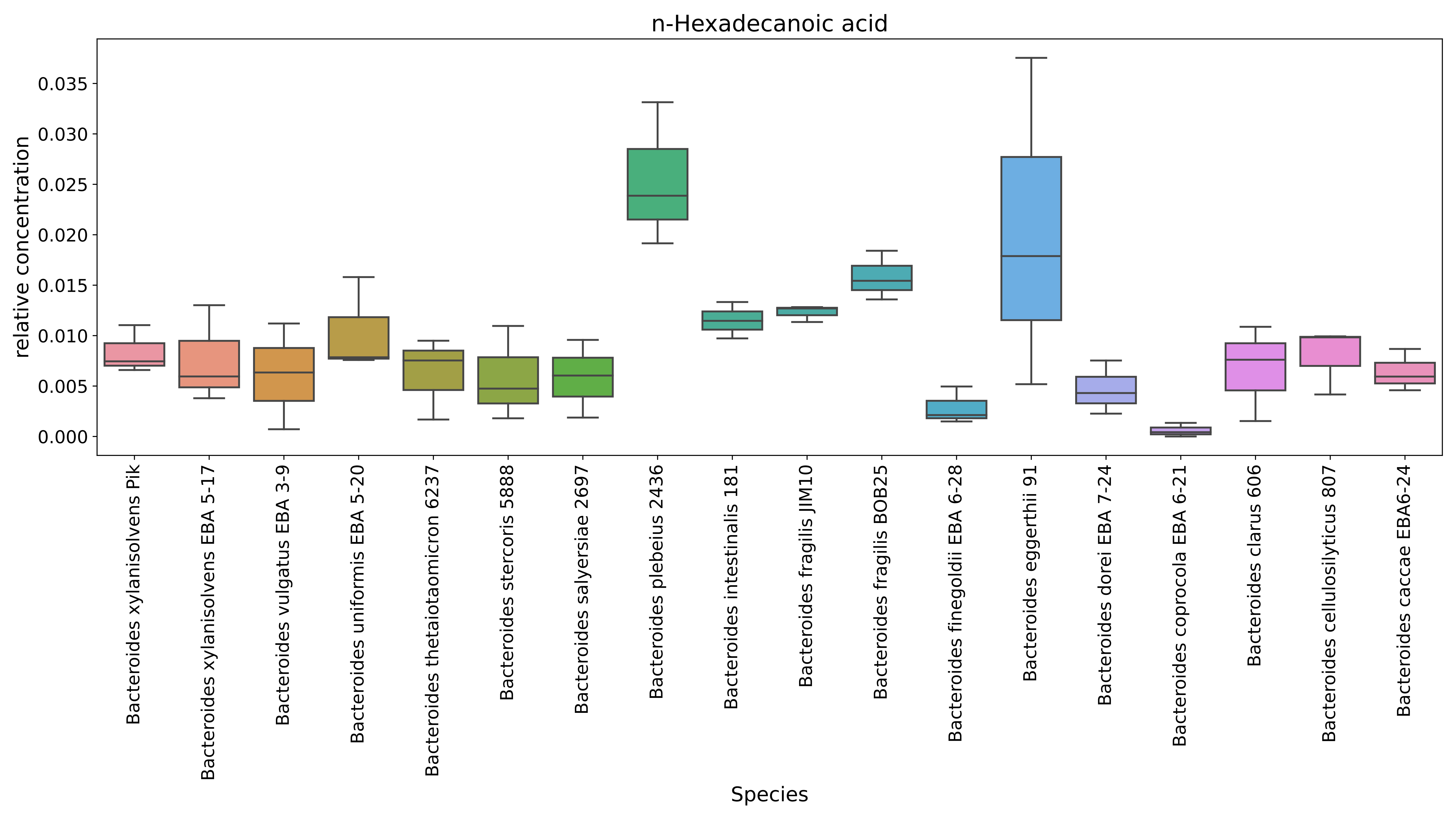
n-Hexadecanoic acid
relative concentration
0.000
0.005
0.010
0.015
0.020
0.025
0.030
0.035
Bacteroides xylanisolvens Pik
Bacteroides xylanisolvens EBA 5-17
Bacteroides vulgatus EBA 3-9
Bacteroides uniformis EBA 5-20
Bacteroides thetaiotaomicron 6237
Bacteroides stercoris 5888
Bacteroides salyersiae 2697
Bacteroides plebeius 2436
Bacteroides intestinalis 181
Bacteroides fragilis JIM10
Bacteroides fragilis BOB25
Bacteroides finegoldii EBA 6-28
Bacteroides eggerthii 91
Bacteroides dorei EBA 7-24
Bacteroides coprocola EBA 6-21
Bacteroides clarus 606
Bacteroides cellulosilyticus 807
Bacteroides caccae EBA6-24
Species

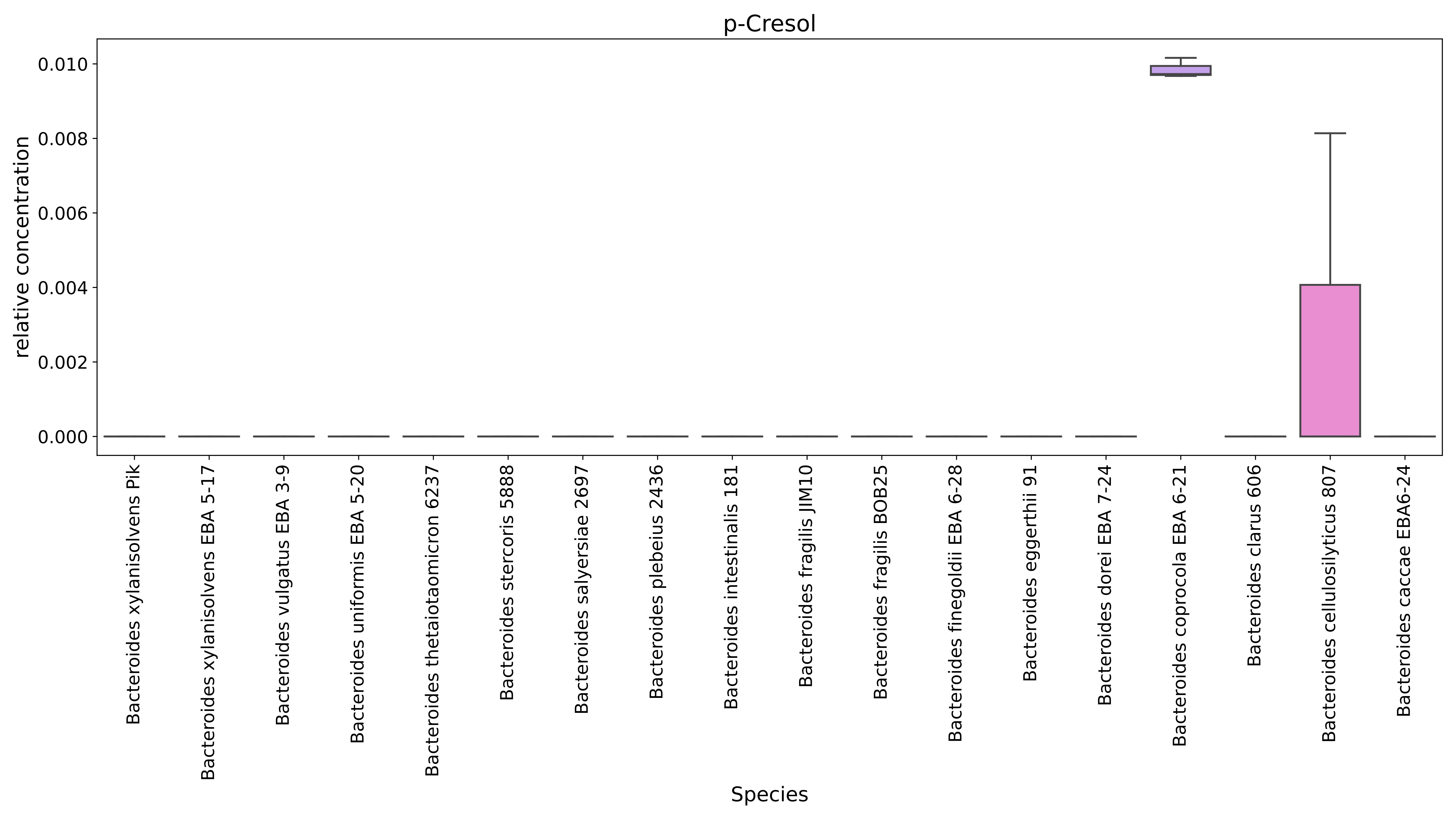
p-Cresol
relative concentration
0.010
0.008
0.006
0.004
0.002
0.000
Bacteroides xylanisolvens Pik
Bacteroides xylanisolvens EBA 5-17
Bacteroides vulgatus EBA 3-9
Bacteroides uniformis EBA 5-20
Bacteroides thetaiotaomicron 6237
Bacteroides stercoris 5888
Bacteroides salyersiae 2697
Bacteroides plebeius 2436
Bacteroides intestinalis 181
Bacteroides fragilis JIM10
Bacteroides fragilis BOB25
Bacteroides finegoldii EBA 6-28
Bacteroides eggerthii 91
Bacteroides dorei EBA 7-24
Bacteroides coprocola EBA 6-21
Bacteroides clarus 606
Bacteroides cellulosilyticus 807
Bacteroides caccae EBA6-24
Species
