# Supplementary material for: Investigating volatile compounds in the Bacteroides secretome
Source: Front Microbiol. 2023 May 3;14:1164877. doi: 10.3389/fmicb.2023.1164877 (PMC10189065; doi:10.3389/fmicb.2023.1164877)
Supplement: Supplementary file 4 [file Data_Sheet_2.PDF]

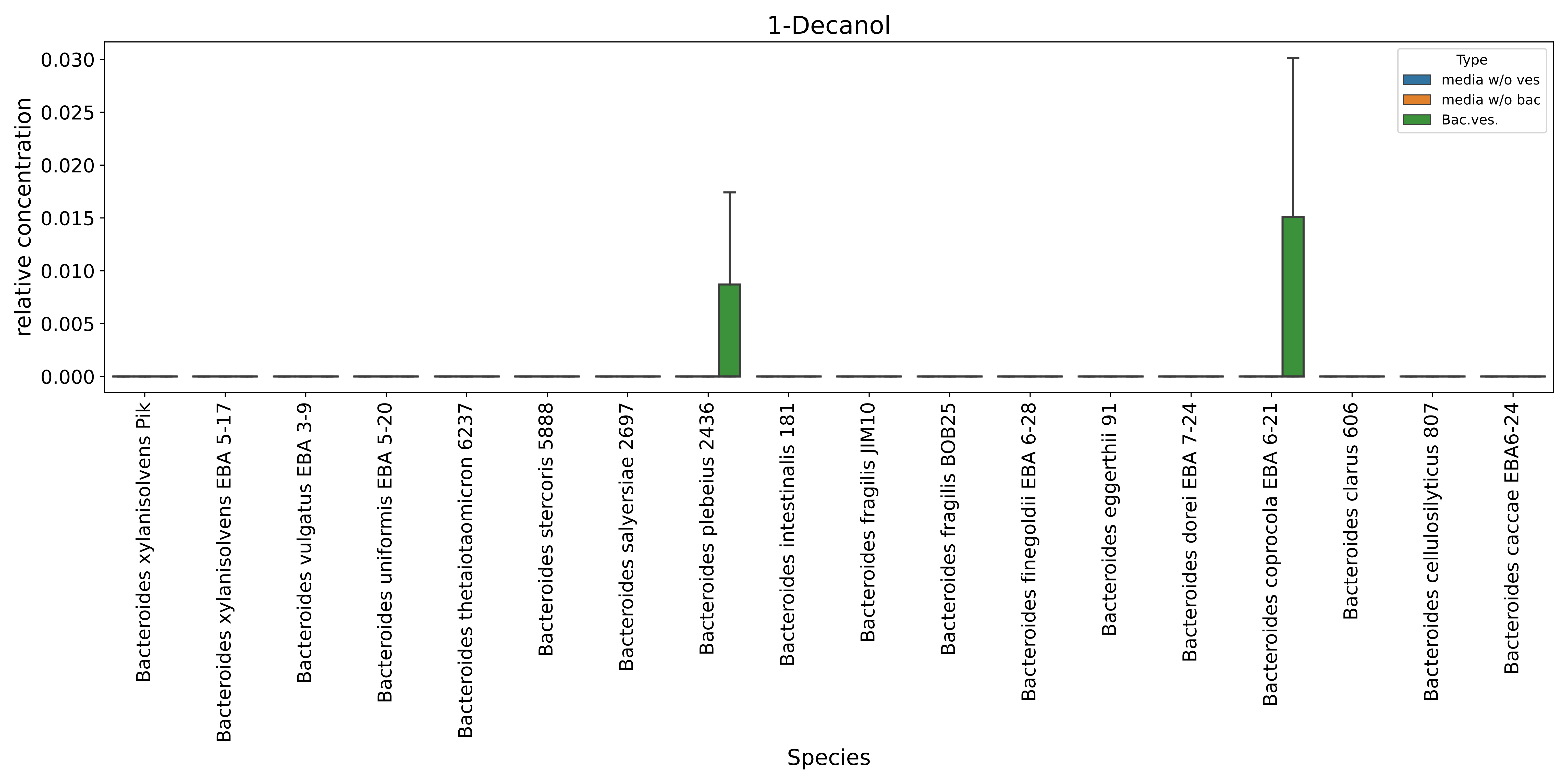

1-Decanol
relative concentration
0.030
0.025
0.020
0.015
0.010
0.005
0.000
Type
media w/o ves
media w/o bac
Bac.ves.
Bacteroides xylanisolvens Pik
Bacteroides xylanisolvens EBA 5-17
Bacteroides vulgatus EBA 3-9
Bacteroides uniformis EBA 5-20
Bacteroides thetaiotaomicron 6237
Bacteroides stercoris 5888
Bacteroides salyersiae 2697
Bacteroides plebeius 2436
Bacteroides intestinalis 181
Bacteroides fragilis JIM10
Bacteroides fragilis BOB25
Bacteroides finegoldii EBA 6-28
Bacteroides eggerthii 91
Bacteroides dorei EBA 7-24
Bacteroides coprocola EBA 6-21
Bacteroides clarus 606
Bacteroides cellulosilyticus 807
Bacteroides caccae EBA6-24
Species

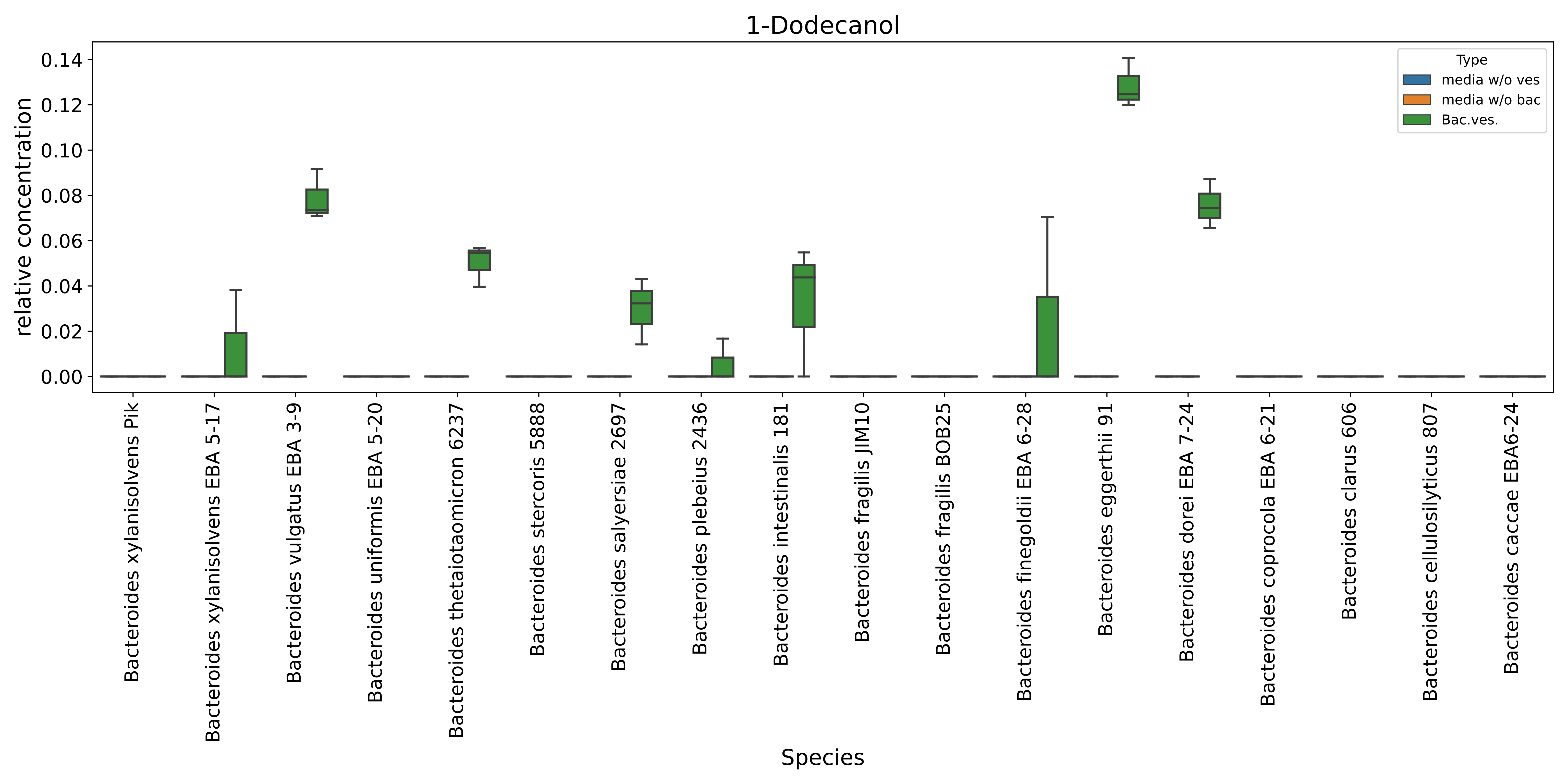
1-Dodecanol
relative concentration
0.14
0.12
0.10
0.08
0.06
0.04
0.02
0.00
Type
media w/o ves
media w/o bac
Bac.ves.
Bacteroides xylanisolvens Pik
Bacteroides xylanisolvens EBA 5-17
Bacteroides vulgatus EBA 3-9
Bacteroides uniformis EBA 5-20
Bacteroides thetaiotaomicron 6237
Bacteroides stercoris 5888
Bacteroides salyersiae 2697
Bacteroides plebeius 2436
Bacteroides intestinalis 181
Bacteroides fragilis JIM10
Bacteroides fragilis BOB25
Bacteroides finegoldii EBA 6-28
Bacteroides eggerthii 91
Bacteroides dorei EBA 7-24
Bacteroides coprocola EBA 6-21
Bacteroides clarus 606
Bacteroides cellulosilyticus 807
Bacteroides caccae EBA6-24
Species

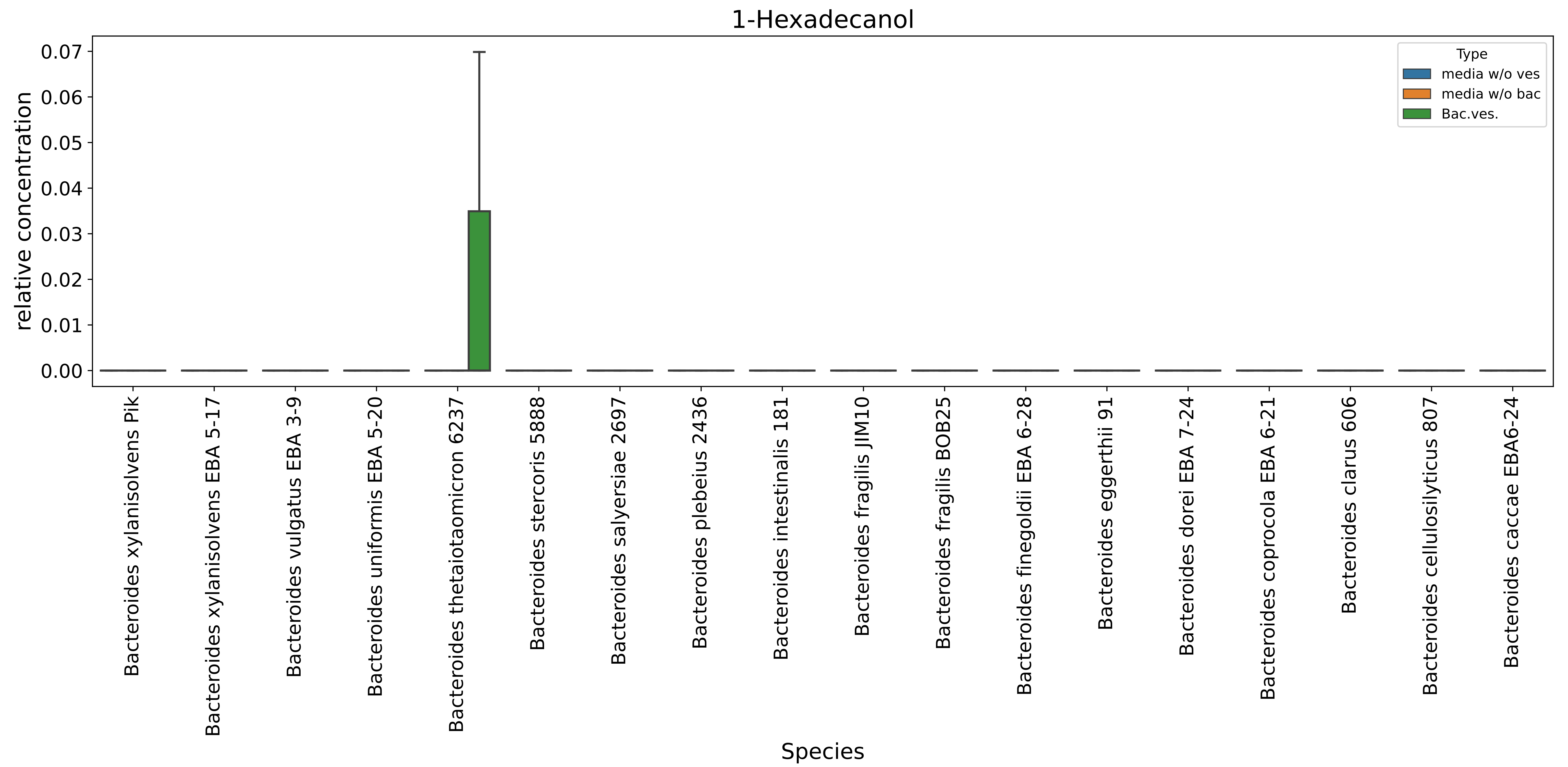

1-Hexadecanol
relative concentration
0.07
0.06
0.05
0.04
0.03
0.02
0.01
0.00
Type
media w/o ves
media w/o bac
Bac.ves.
Bacteroides xylanisolvens Pik
Bacteroides xylanisolvens EBA 5-17
Bacteroides vulgatus EBA 3-9
Bacteroides uniformis EBA 5-20
Bacteroides thetaiotaomicron 6237
Bacteroides stercoris 5888
Bacteroides salyersiae 2697
Bacteroides plebeius 2436
Bacteroides intestinalis 181
Bacteroides fragilis JIM10
Bacteroides fragilis BOB25
Bacteroides finegoldii EBA 6-28
Bacteroides eggerthii 91
Bacteroides dorei EBA 7-24
Bacteroides coprocola EBA 6-21
Bacteroides clarus 606
Bacteroides cellulosilyticus 807
Bacteroides caccae EBA6-24
Species

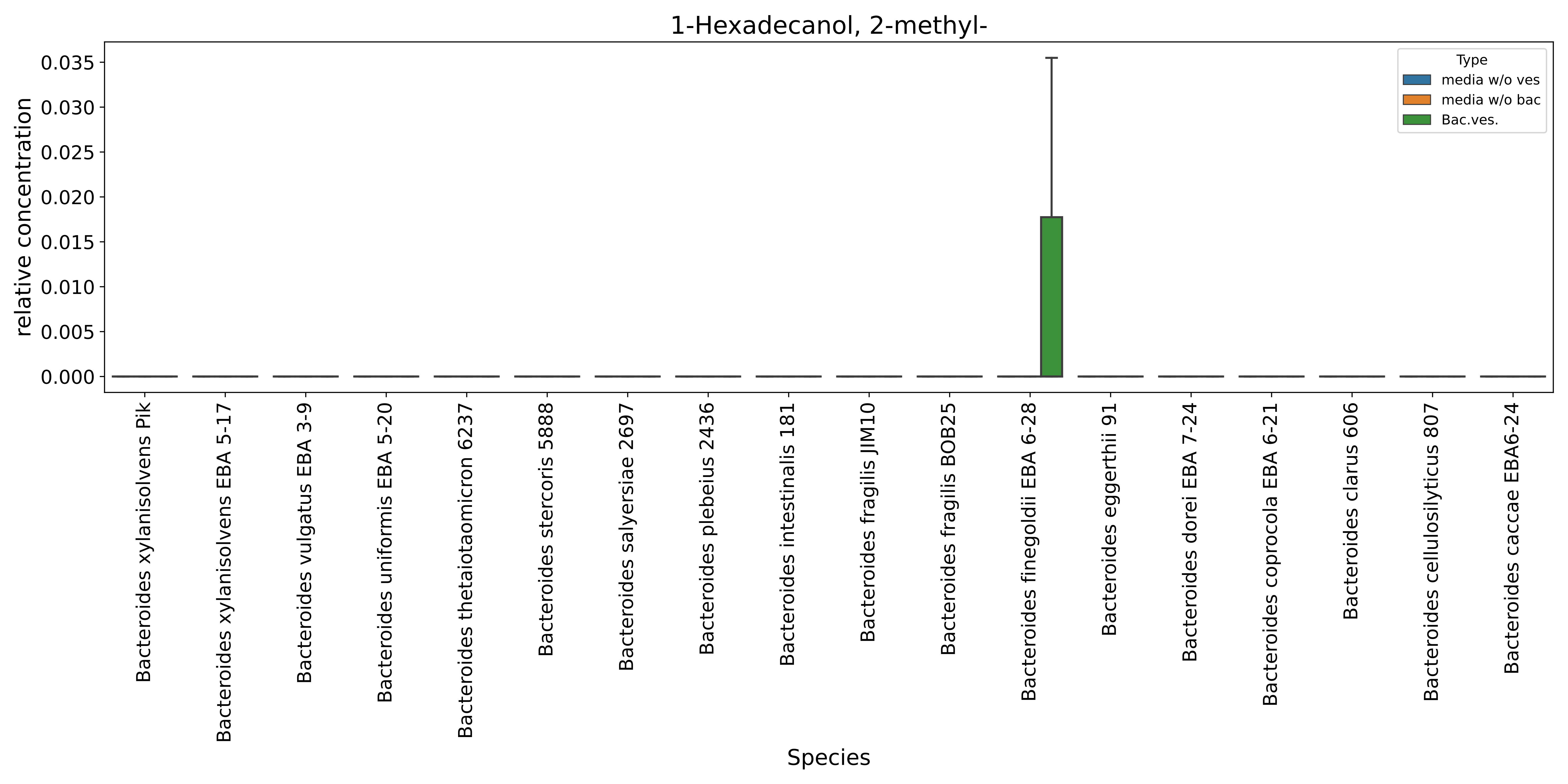

1-Hexadecanol, 2-methyl-
relative concentration
0.035
0.030
0.025
0.020
0.015
0.010
0.005
0.000
Type
media w/o ves
media w/o bac
Bac.ves.
Bacteroides xylanisolvens Pik
Bacteroides xylanisolvens EBA 5-17
Bacteroides vulgatus EBA 3-9
Bacteroides uniformis EBA 5-20
Bacteroides thetaiotaomicron 6237
Bacteroides stercoris 5888
Bacteroides salyersiae 2697
Bacteroides plebeius 2436
Bacteroides intestinalis 181
Bacteroides fragilis JIM10
Bacteroides fragilis BOB25
Bacteroides finegoldii EBA 6-28
Bacteroides eggerthii 91
Bacteroides dorei EBA 7-24
Bacteroides coprocola EBA 6-21
Bacteroides clarus 606
Bacteroides cellulosilyticus 807
Bacteroides caccae EBA6-24
Species

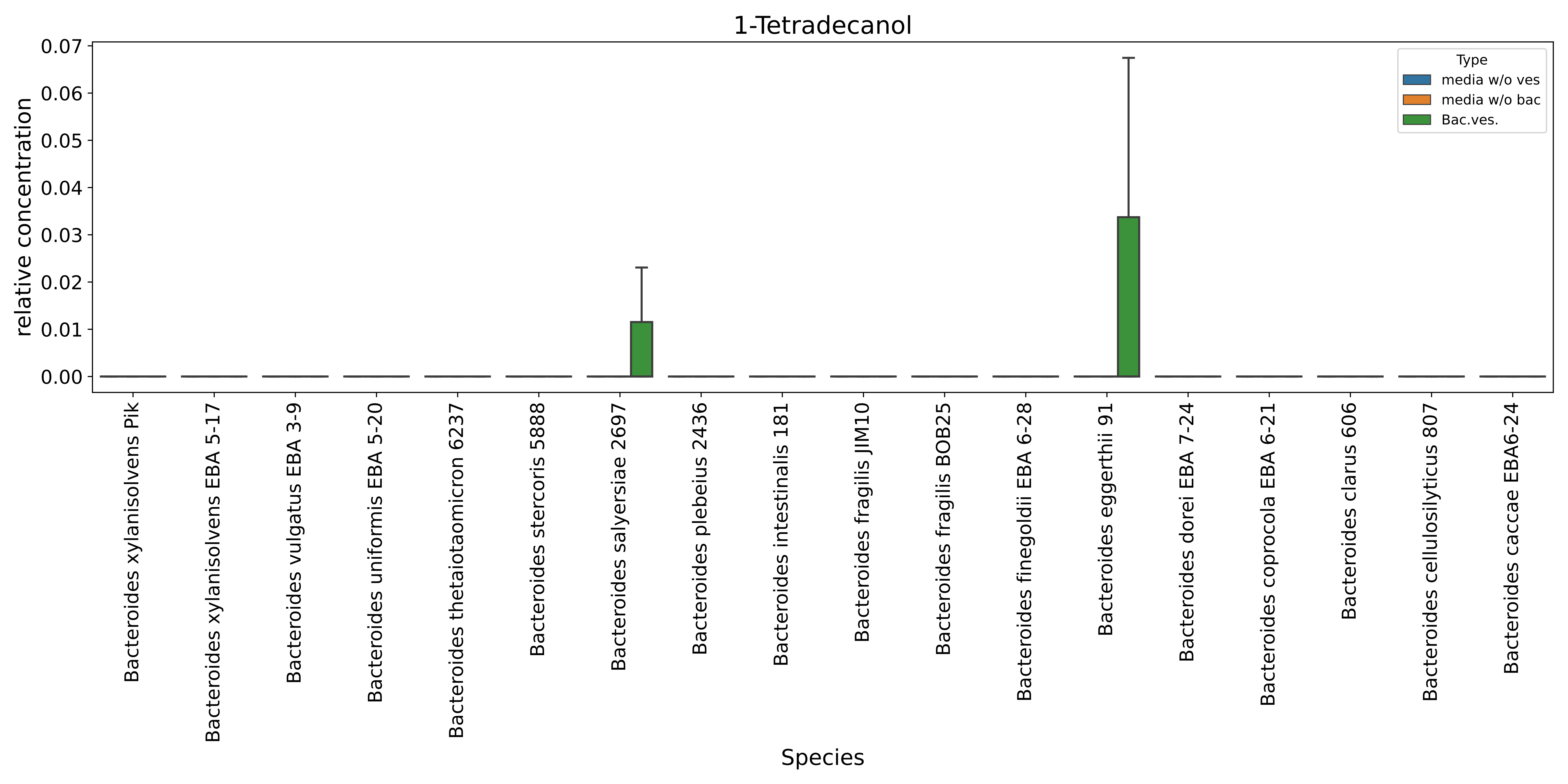

1-Tetradecanol
relative concentration
0.07
0.06
0.05
0.04
0.03
0.02
0.01
0.00
Type
media w/o ves
media w/o bac
Bac.ves.
Bacteroides xylanisolvens Pik
Bacteroides xylanisolvens EBA 5-17
Bacteroides vulgatus EBA 3-9
Bacteroides uniformis EBA 5-20
Bacteroides thetaiotaomicron 6237
Bacteroides stercoris 5888
Bacteroides salyersiae 2697
Bacteroides plebeius 2436
Bacteroides intestinalis 181
Bacteroides fragilis JIM10
Bacteroides fragilis BOB25
Bacteroides finegoldii EBA 6-28
Bacteroides eggerthii 91
Bacteroides dorei EBA 7-24
Bacteroides coprocola EBA 6-21
Bacteroides clarus 606
Bacteroides cellulosilyticus 807
Bacteroides caccae EBA6-24
Species

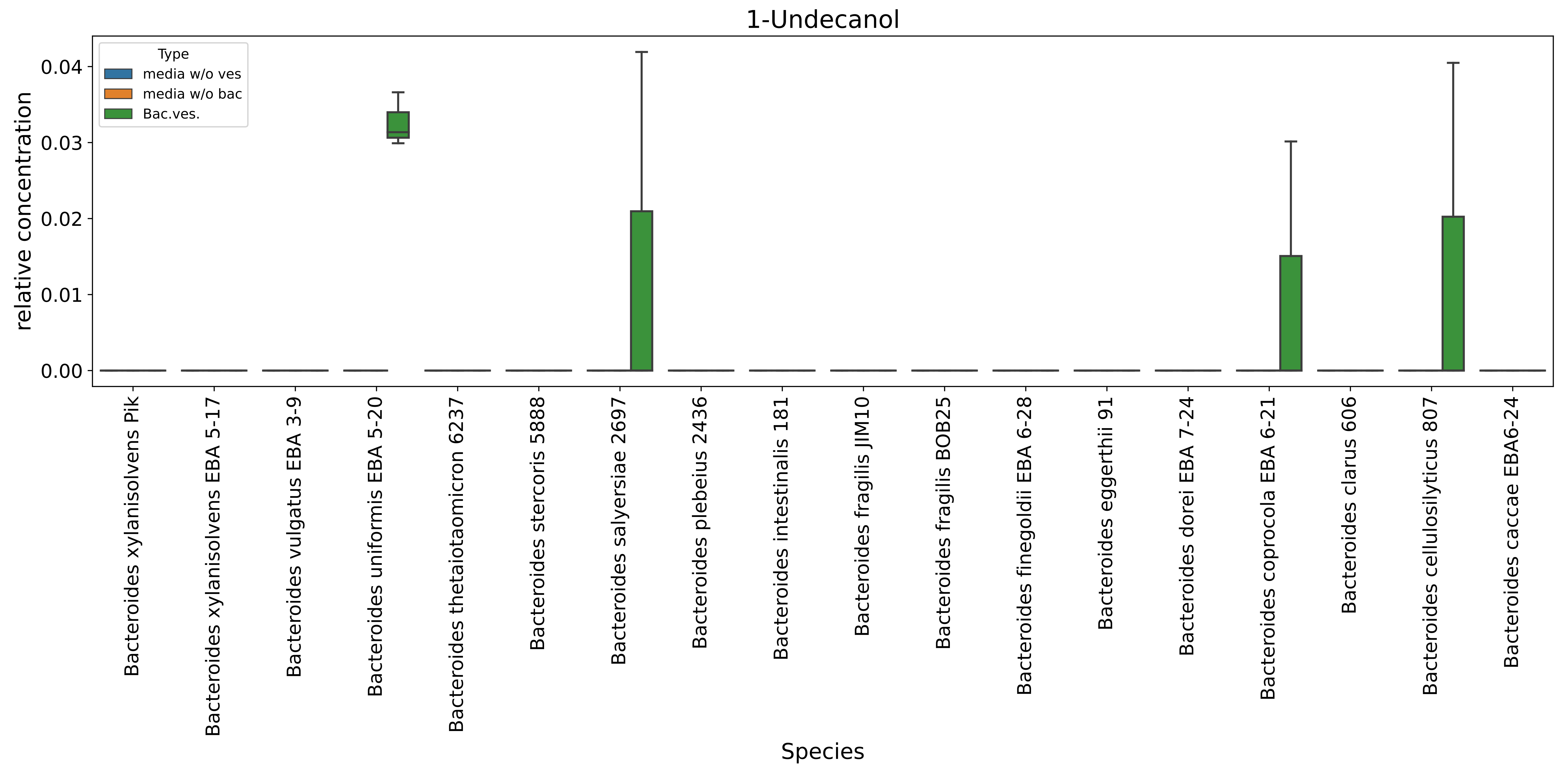
1-Undecanol
Type
media w/o ves
media w/o bac
Bac.ves.
relative concentration
0.04
0.03
0.02
0.01
0.00
Bacteroides xylanisolvens Pik
Bacteroides xylanisolvens EBA 5-17
Bacteroides vulgatus EBA 3-9
Bacteroides uniformis EBA 5-20
Bacteroides thetaiotaomicron 6237
Bacteroides stercoris 5888
Bacteroides salyersiae 2697
Bacteroides plebeius 2436
Bacteroides intestinalis 181
Bacteroides fragilis JIM10
Bacteroides fragilis BOB25
Bacteroides finegoldii EBA 6-28
Bacteroides eggerthii 91
Bacteroides dorei EBA 7-24
Bacteroides coprocola EBA 6-21
Bacteroides clarus 606
Bacteroides cellulosilyticus 807
Bacteroides caccae EBA6-24
Species

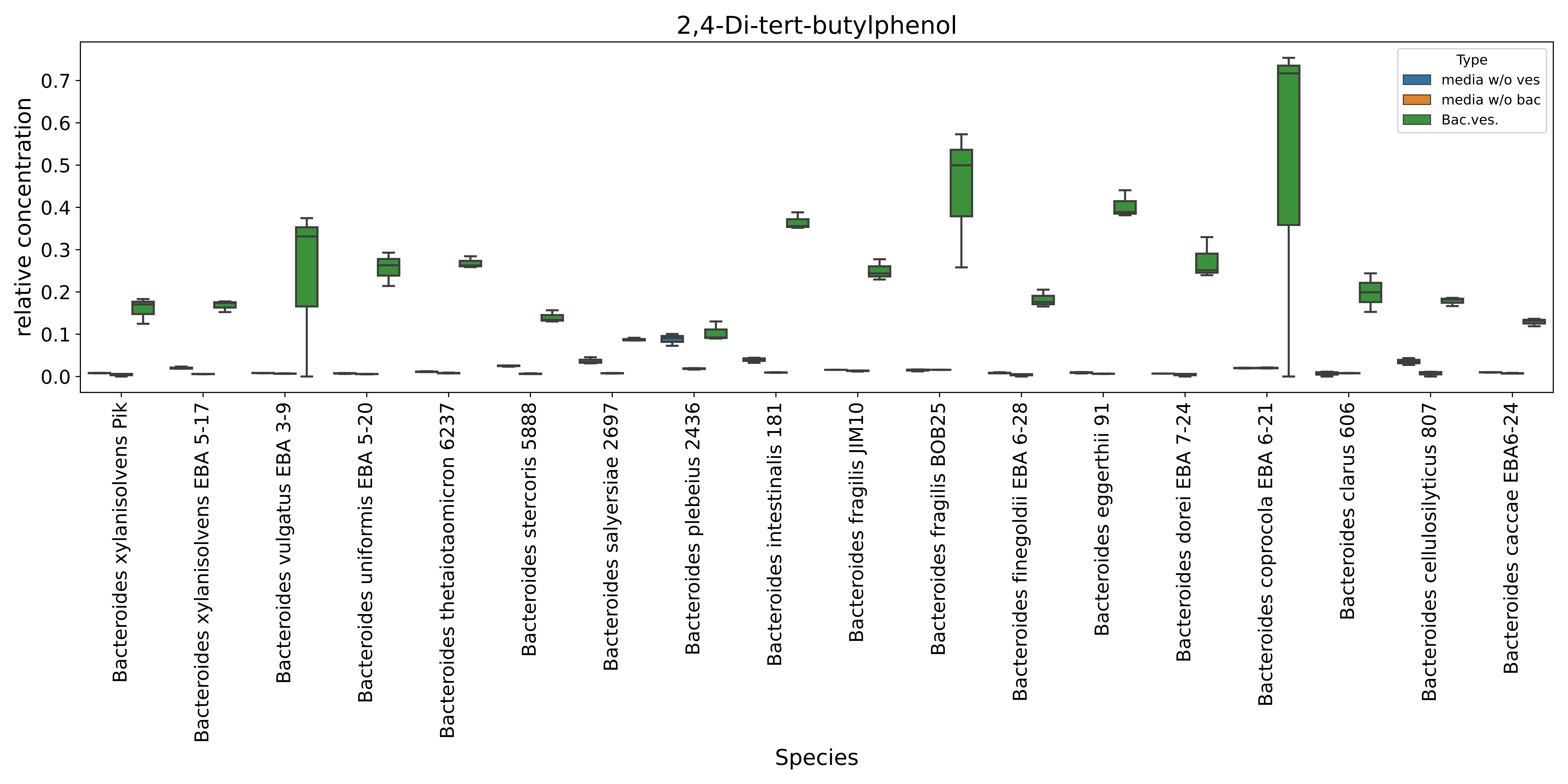
2,4-Di-tert-butylphenol
relative concentration
0.0
0.1
0.2
0.3
0.4
0.5
0.6
0.7
Type
media w/o ves
media w/o bac
Bac.ves.
Bacteroides xylanisolvens Pik
Bacteroides xylanisolvens EBA 5-17
Bacteroides vulgatus EBA 3-9
Bacteroides uniformis EBA 5-20
Bacteroides thetaiotaomicron 6237
Bacteroides stercoris 5888
Bacteroides salyersiae 2697
Bacteroides plebeius 2436
Bacteroides intestinalis 181
Bacteroides fragilis JIM10
Bacteroides fragilis BOB25
Bacteroides finegoldii EBA 6-28
Bacteroides eggerthii 91
Bacteroides dorei EBA 7-24
Bacteroides coprocola EBA 6-21
Bacteroides clarus 606
Bacteroides cellulosilyticus 807
Bacteroides caccae EBA6-24
Species

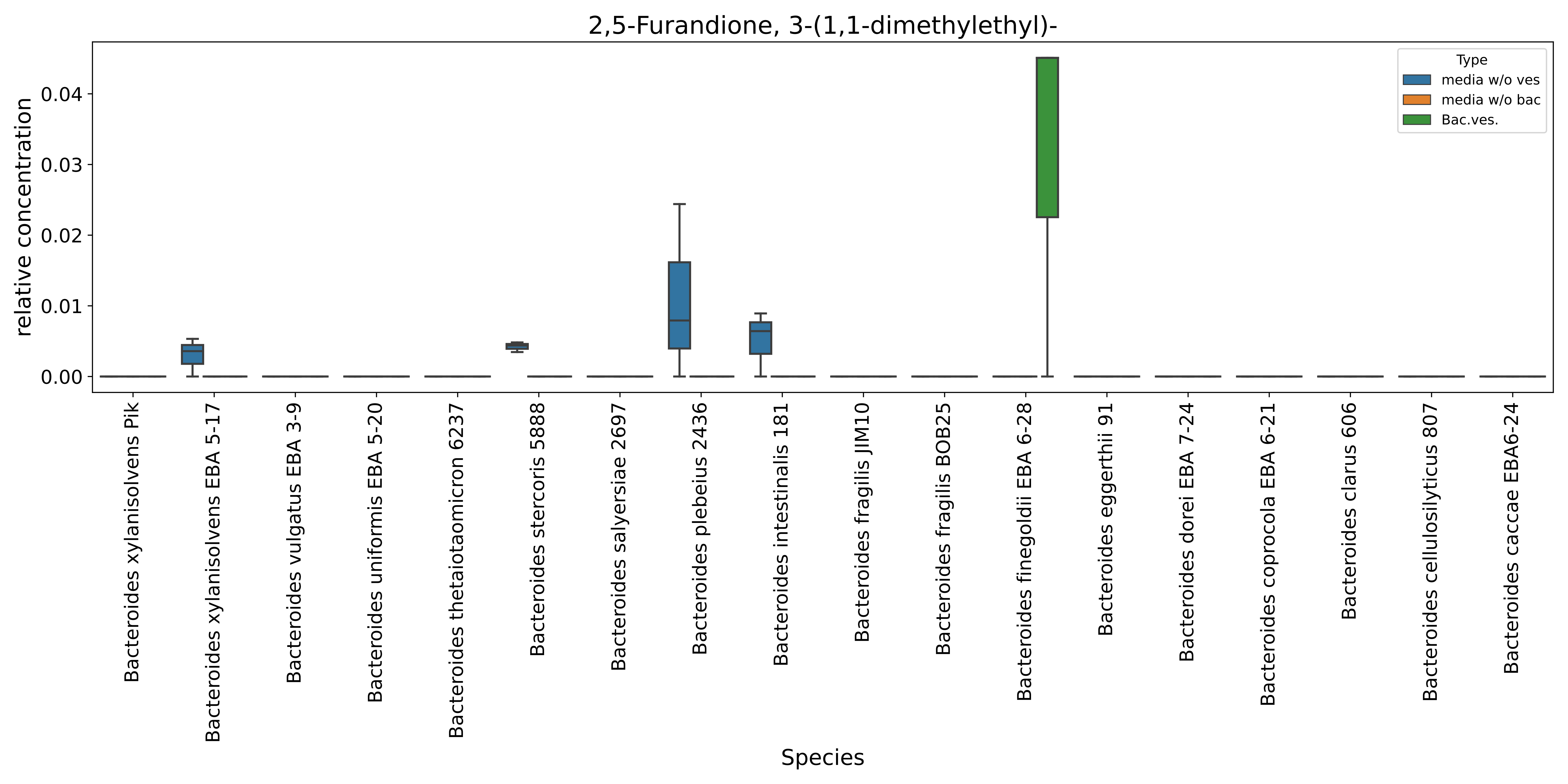
2,5-Furandione, 3-(1,1-dimethylethyl)-
relative concentration
0.04
0.03
0.02
0.01
0.00
Type
media w/o ves
media w/o bac
Bac.ves.
Bacteroides xylanisolvens Pik
Bacteroides xylanisolvens EBA 5-17
Bacteroides vulgatus EBA 3-9
Bacteroides uniformis EBA 5-20
Bacteroides thetaiotaomicron 6237
Bacteroides stercoris 5888
Bacteroides salyersiae 2697
Bacteroides plebeius 2436
Bacteroides intestinalis 181
Bacteroides fragilis JIM10
Bacteroides fragilis BOB25
Bacteroides finegoldii EBA 6-28
Bacteroides eggerthii 91
Bacteroides dorei EBA 7-24
Bacteroides coprocola EBA 6-21
Bacteroides clarus 606
Bacteroides cellulosilyticus 807
Bacteroides caccae EBA6-24
Species

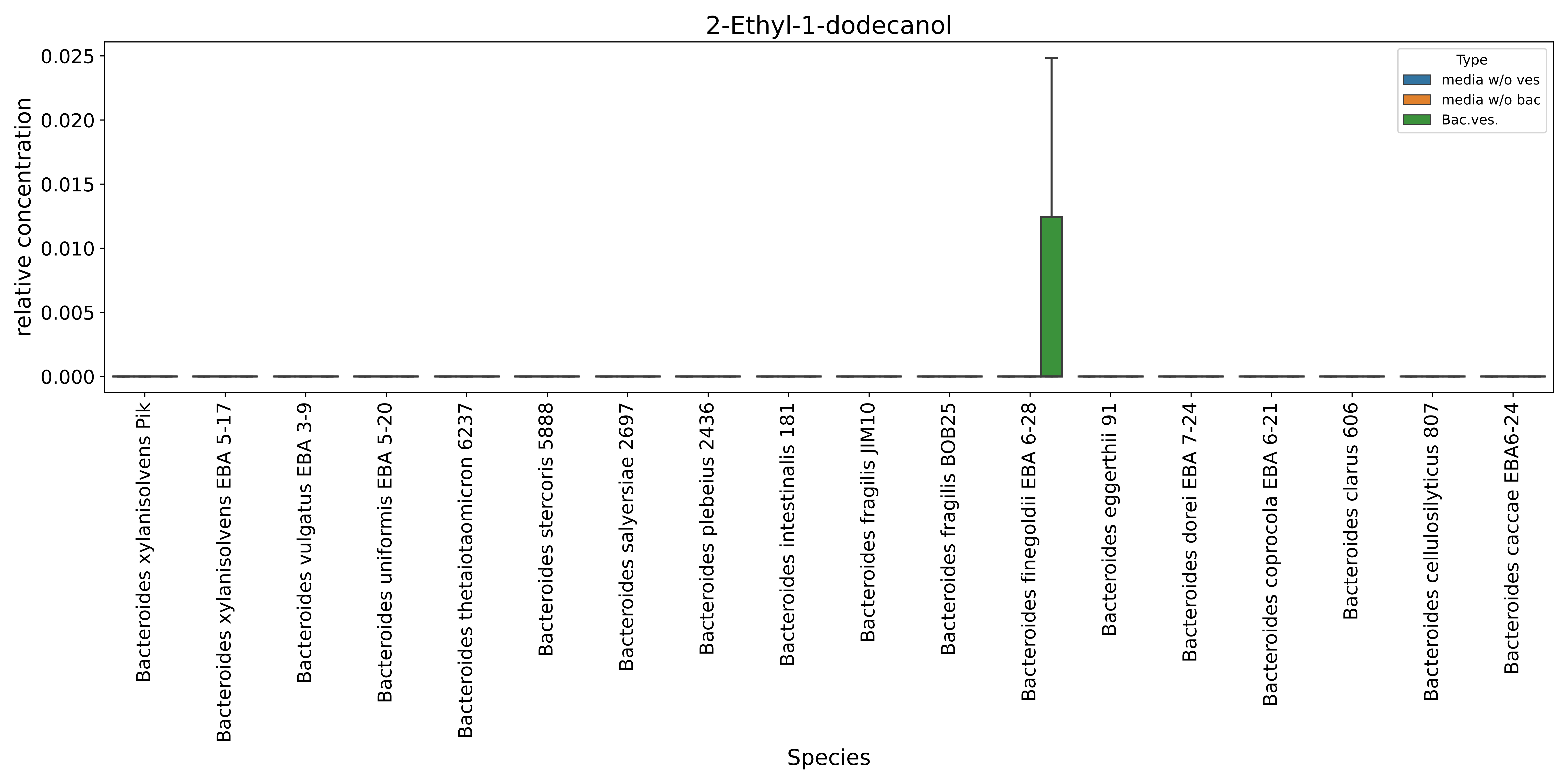

2-Ethyl-1-dodecanol
relative concentration
0.025
0.020
0.015
0.010
0.005
0.000
Type
media w/o ves
media w/o bac
Bac.ves.
Bacteroides xylanisolvens Pik
Bacteroides xylanisolvens EBA 5-17
Bacteroides vulgatus EBA 3-9
Bacteroides uniformis EBA 5-20
Bacteroides thetaiotaomicron 6237
Bacteroides stercoris 5888
Bacteroides salyersiae 2697
Bacteroides plebeius 2436
Bacteroides intestinalis 181
Bacteroides fragilis JIM10
Bacteroides fragilis BOB25
Bacteroides finegoldii EBA 6-28
Bacteroides eggerthii 91
Bacteroides dorei EBA 7-24
Bacteroides coprocola EBA 6-21
Bacteroides clarus 606
Bacteroides cellulosilyticus 807
Bacteroides caccae EBA6-24
Species

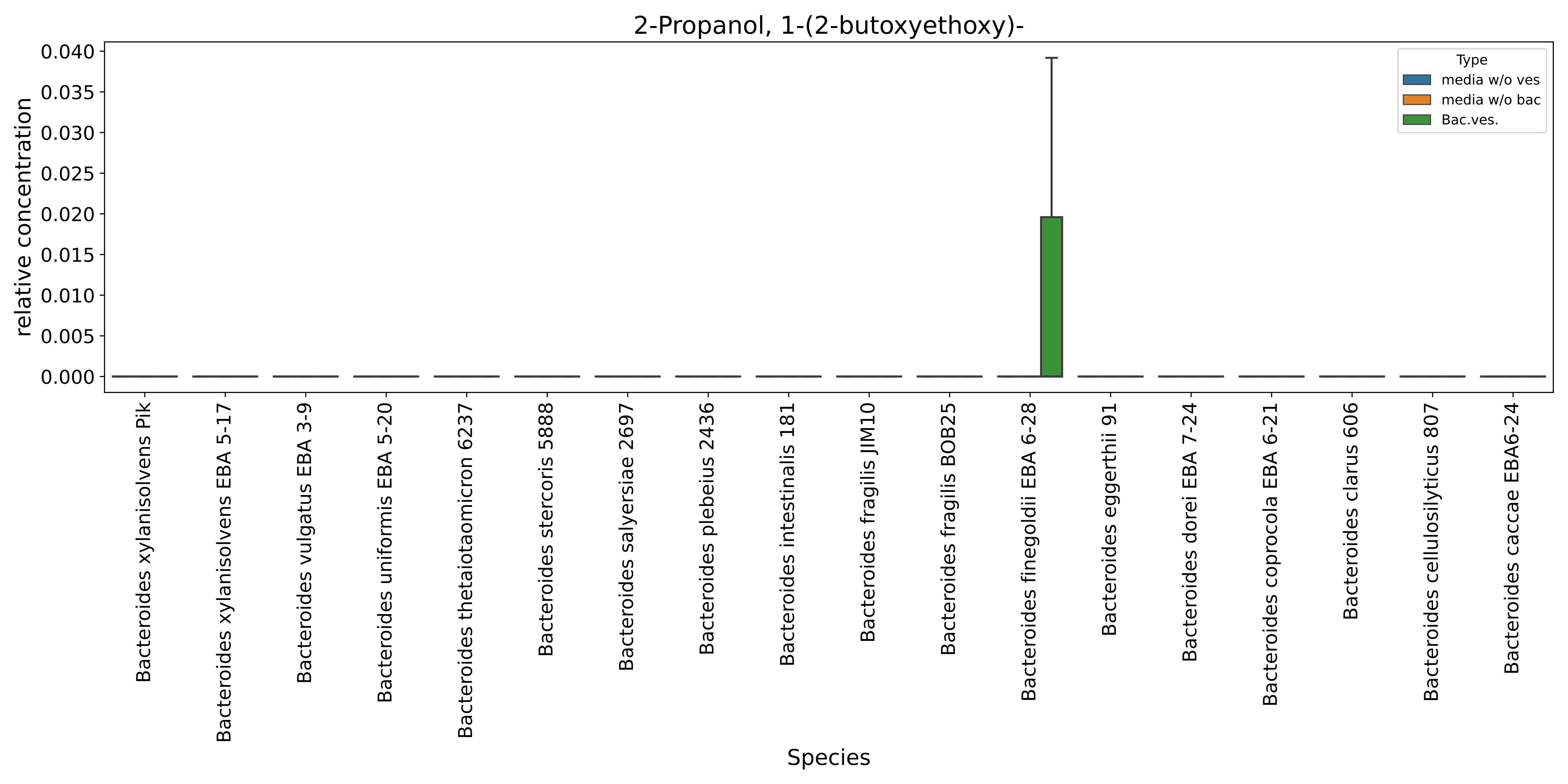
2-Propanol, 1-(2-butoxyethoxy)-
relative concentration
0.040
0.035
0.030
0.025
0.020
0.015
0.010
0.005
0.000
Type
media w/o ves
media w/o bac
Bac.ves.
Bacteroides xylanisolvens Pik
Bacteroides xylanisolvens EBA 5-17
Bacteroides vulgatus EBA 3-9
Bacteroides uniformis EBA 5-20
Bacteroides thetaiotaomicron 6237
Bacteroides stercoris 5888
Bacteroides salyersiae 2697
Bacteroides plebeius 2436
Bacteroides intestinalis 181
Bacteroides fragilis JIM10
Bacteroides fragilis BOB25
Bacteroides finegoldii EBA 6-28
Bacteroides eggerthii 91
Bacteroides dorei EBA 7-24
Bacteroides coprocola EBA 6-21
Bacteroides clarus 606
Bacteroides cellulosilyticus 807
Bacteroides caccae EBA6-24
Species

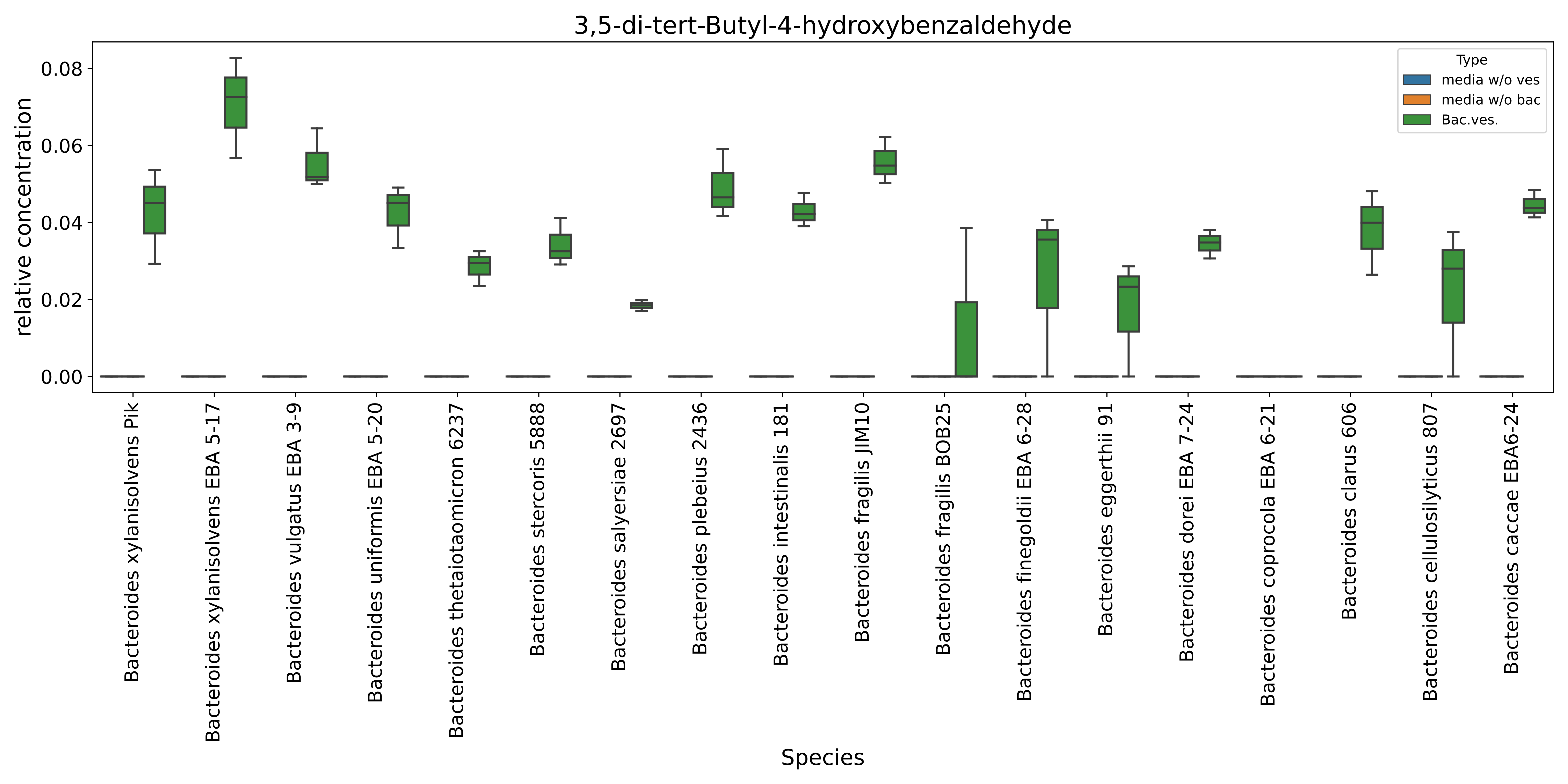
3,5-di-tert-Butyl-4-hydroxybenzaldehyde
relative concentration
0.00
0.02
0.04
0.06
0.08
Type
media w/o ves
media w/o bac
Bac.ves.
Bacteroides xylanisolvens Pik
Bacteroides xylanisolvens EBA 5-17
Bacteroides vulgatus EBA 3-9
Bacteroides uniformis EBA 5-20
Bacteroides thetaiotaomicron 6237
Bacteroides stercoris 5888
Bacteroides salyersiae 2697
Bacteroides plebeius 2436
Bacteroides intestinalis 181
Bacteroides fragilis JIM10
Bacteroides fragilis BOB25
Bacteroides finegoldii EBA 6-28
Bacteroides eggerthii 91
Bacteroides dorei EBA 7-24
Bacteroides coprocola EBA 6-21
Bacteroides clarus 606
Bacteroides cellulosilyticus 807
Bacteroides caccae EBA6-24
Species

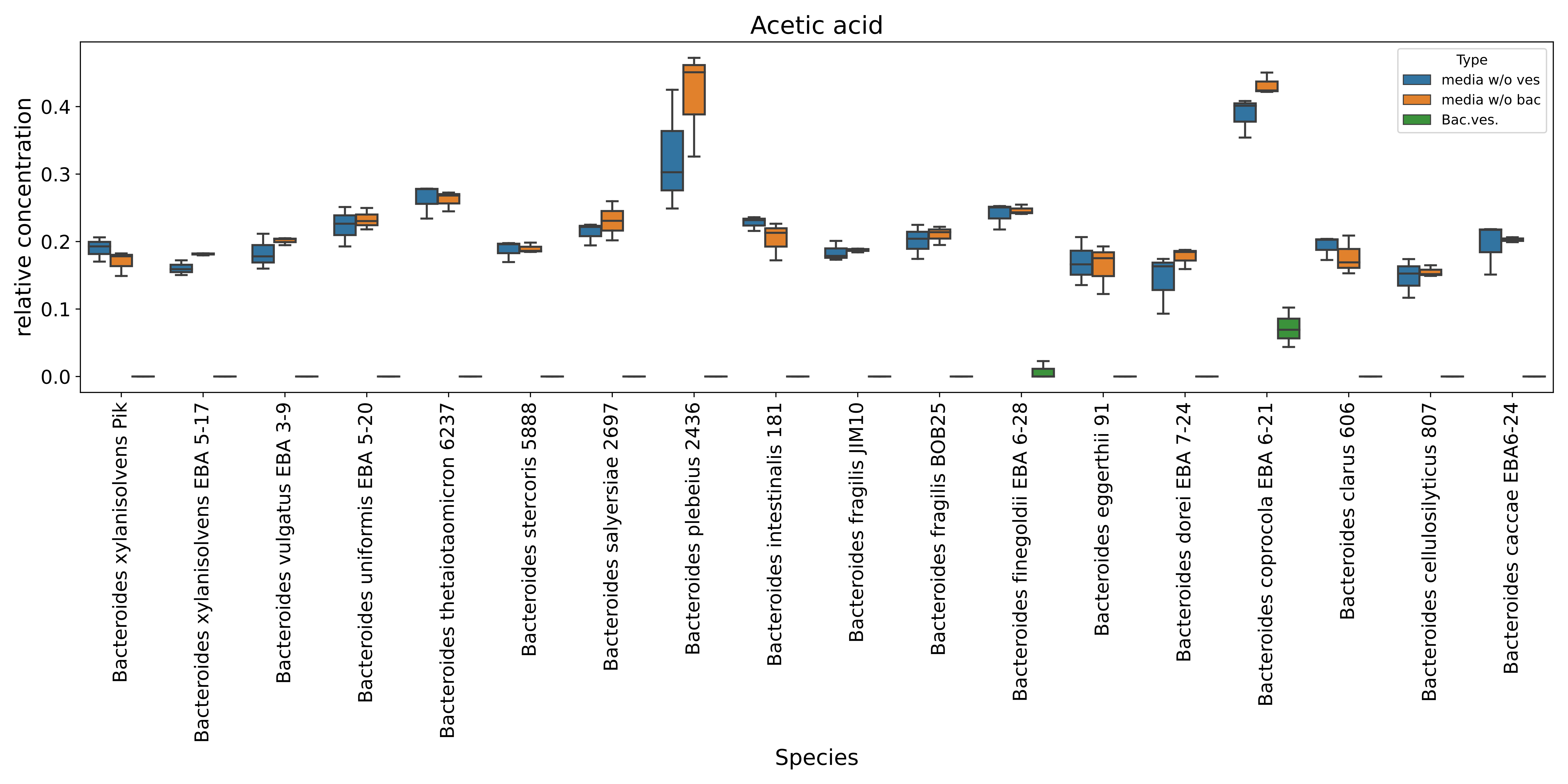

Acetic acid
relative concentration
0.0
0.1
0.2
0.3
0.4
Type
media w/o ves
media w/o bac
Bac.ves.
Species
Bacteroides xylanisolvens Pik
Bacteroides xylanisolvens EBA 5-17
Bacteroides vulgatus EBA 3-9
Bacteroides uniformis EBA 5-20
Bacteroides thetaiotaomicron 6237
Bacteroides stercoris 5888
Bacteroides salyersiae 2697
Bacteroides plebeius 2436
Bacteroides intestinalis 181
Bacteroides fragilis JIM10
Bacteroides fragilis BOB25
Bacteroides finegoldii EBA 6-28
Bacteroides eggerthii 91
Bacteroides dorei EBA 7-24
Bacteroides coprocola EBA 6-21
Bacteroides clarus 606
Bacteroides cellulosilyticus 807
Bacteroides caccae EBA6-24

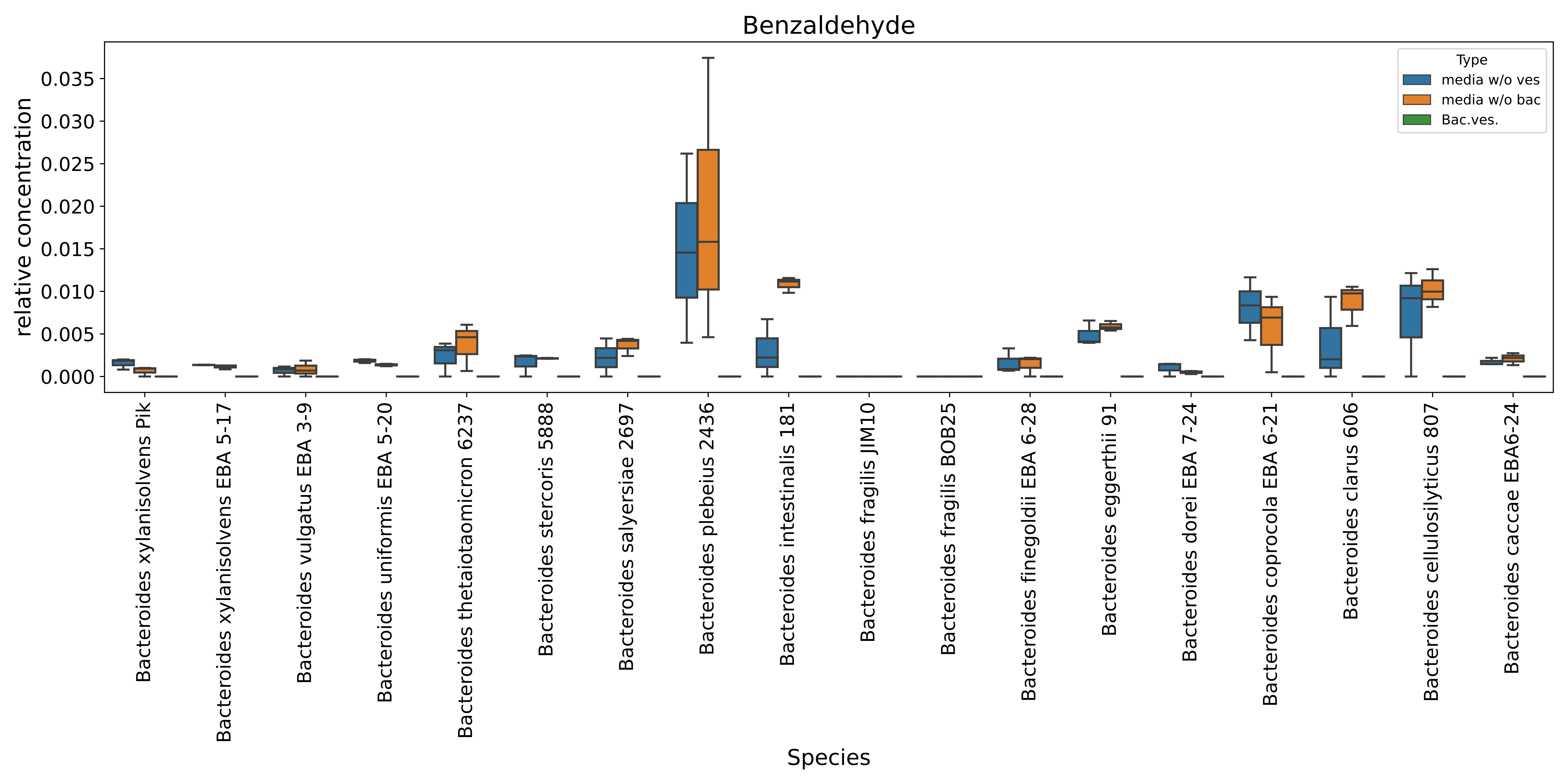

Benzaldehyde
relative concentration
0.035
0.030
0.025
0.020
0.015
0.010
0.005
0.000
Type
media w/o ves
media w/o bac
Bac.ves.
Bacteroides xylanisolvens Pik
Bacteroides xylanisolvens EBA 5-17
Bacteroides vulgatus EBA 3-9
Bacteroides uniformis EBA 5-20
Bacteroides thetaiotaomicron 6237
Bacteroides stercoris 5888
Bacteroides salyersiae 2697
Bacteroides plebeius 2436
Bacteroides intestinalis 181
Bacteroides fragilis JIM10
Bacteroides fragilis BOB25
Bacteroides finegoldii EBA 6-28
Bacteroides eggerthii 91
Bacteroides dorei EBA 7-24
Bacteroides coprocola EBA 6-21
Bacteroides clarus 606
Bacteroides cellulosilyticus 807
Bacteroides caccae EBA6-24
Species

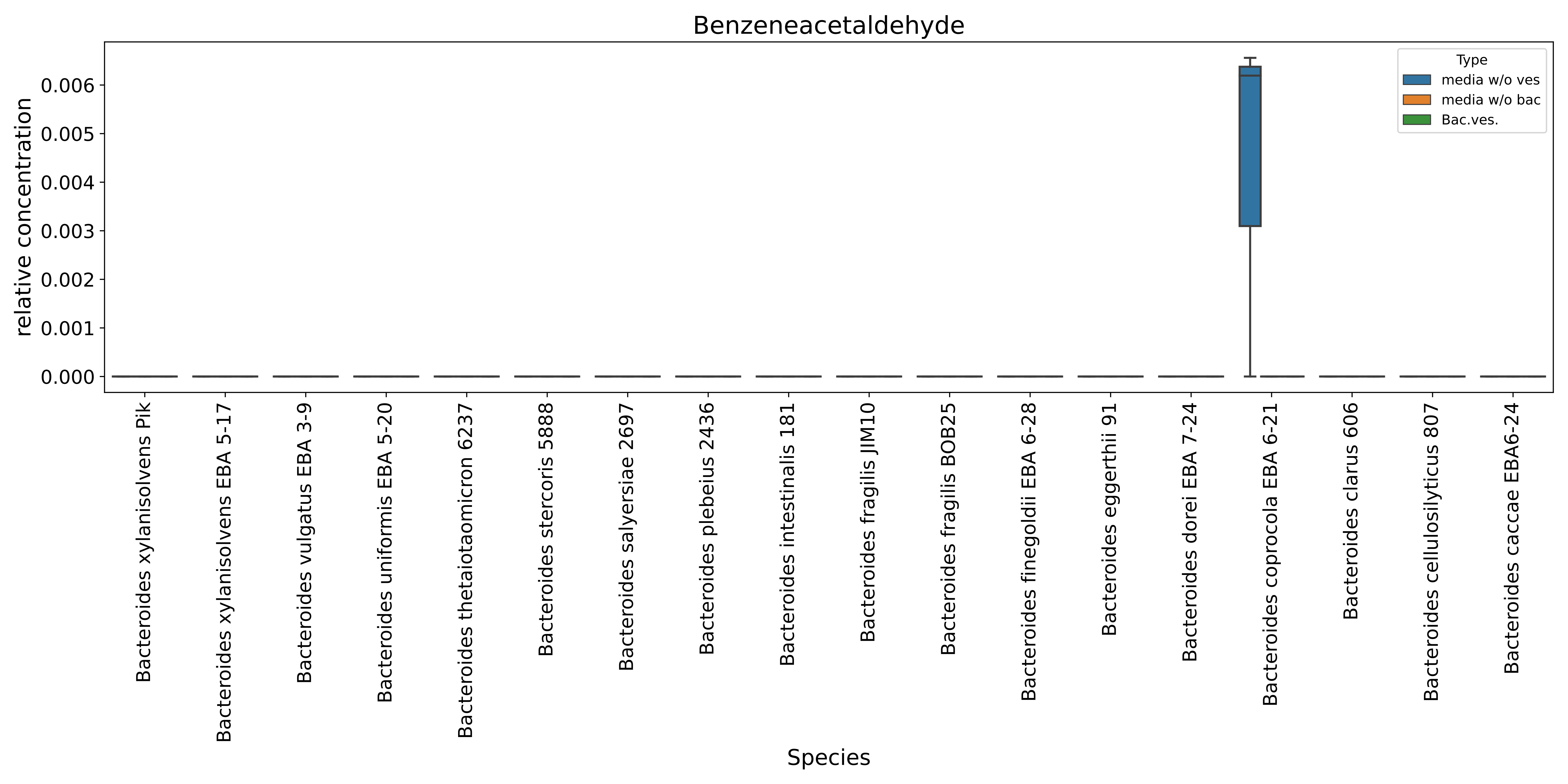

Benzeneacetaldehyde
relative concentration
0.006
0.005
0.004
0.003
0.002
0.001
0.000
Type
media w/o ves
media w/o bac
Bac.ves.
Bacteroides xylanisolvens Pik
Bacteroides xylanisolvens EBA 5-17
Bacteroides vulgatus EBA 3-9
Bacteroides uniformis EBA 5-20
Bacteroides thetaiotaomicron 6237
Bacteroides stercoris 5888
Bacteroides salyersiae 2697
Bacteroides plebeius 2436
Bacteroides intestinalis 181
Bacteroides fragilis JIM10
Bacteroides fragilis BOB25
Bacteroides finegoldii EBA 6-28
Bacteroides eggerthii 91
Bacteroides dorei EBA 7-24
Bacteroides coprocola EBA 6-21
Bacteroides clarus 606
Bacteroides cellulosilyticus 807
Bacteroides caccae EBA6-24
Species

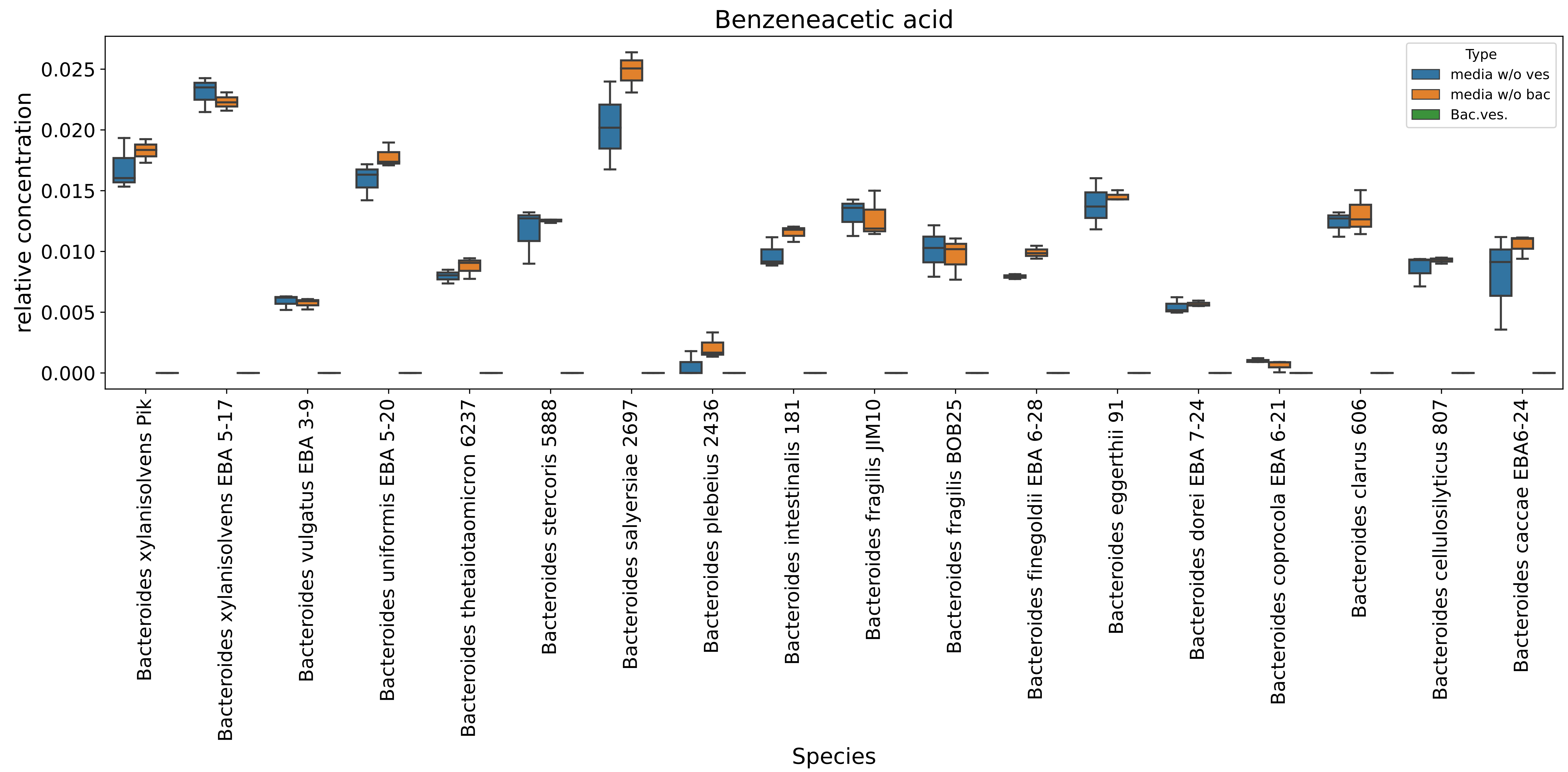
Benzeneacetic acid
relative concentration
0.025
0.020
0.015
0.010
0.005
0.000
Type
media w/o ves
media w/o bac
Bac.ves.
Bacteroides xylanisolvens Pik
Bacteroides xylanisolvens EBA 5-17
Bacteroides vulgatus EBA 3-9
Bacteroides uniformis EBA 5-20
Bacteroides thetaiotaomicron 6237
Bacteroides stercoris 5888
Bacteroides salyersiae 2697
Bacteroides plebeius 2436
Bacteroides intestinalis 181
Bacteroides fragilis JIM10
Bacteroides fragilis BOB25
Bacteroides finegoldii EBA 6-28
Bacteroides eggerthii 91
Bacteroides dorei EBA 7-24
Bacteroides coprocola EBA 6-21
Bacteroides clarus 606
Bacteroides cellulosilyticus 807
Bacteroides caccae EBA6-24
Species

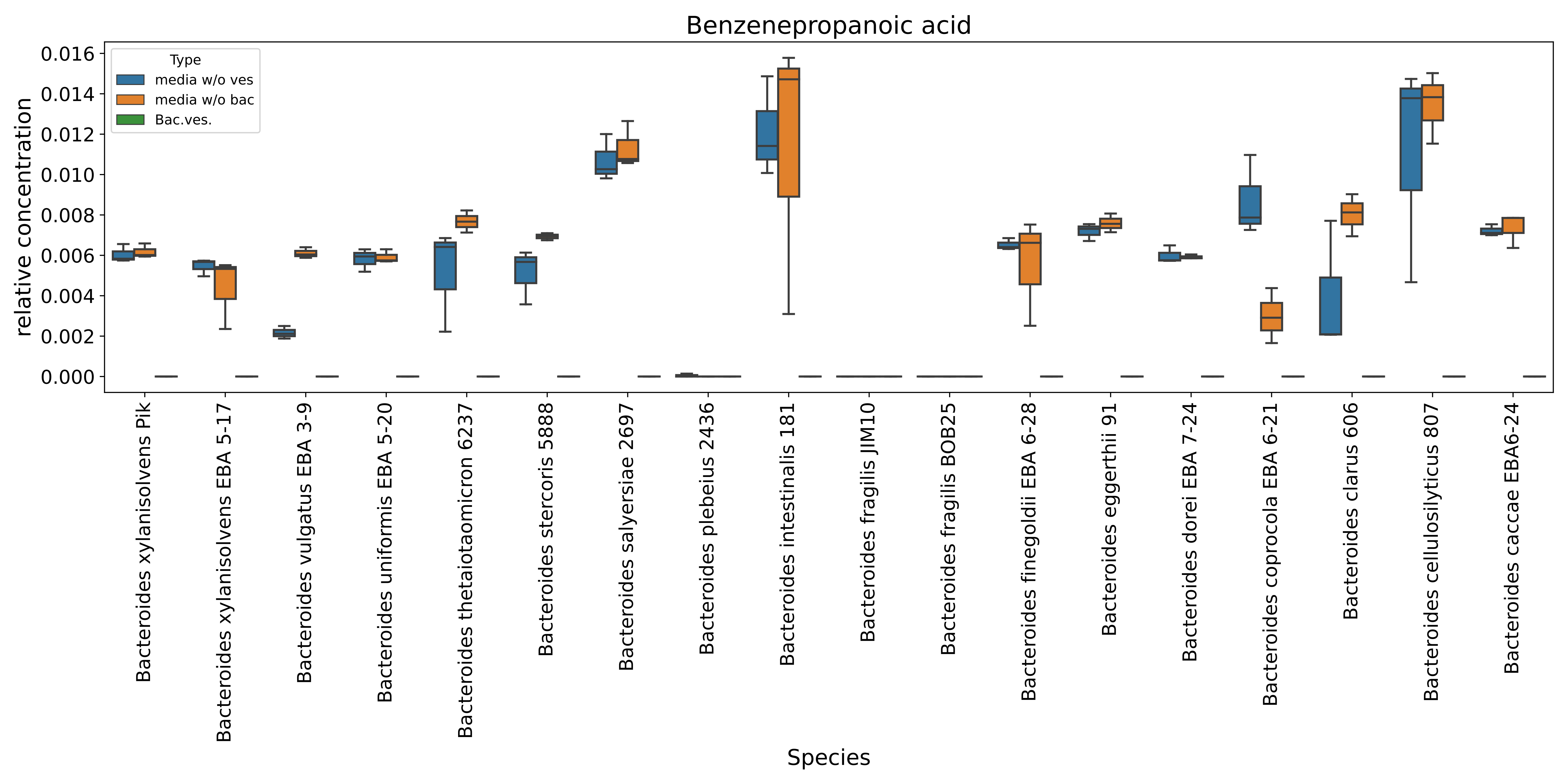
Benzenepropanoic acid
relative concentration
0.016
0.014
0.012
0.010
0.008
0.006
0.004
0.002
0.000
Type
media w/o ves
media w/o bac
Bac.ves.
Bacteroides xylanisolvens Pik
Bacteroides xylanisolvens EBA 5-17
Bacteroides vulgatus EBA 3-9
Bacteroides uniformis EBA 5-20
Bacteroides thetaiotaomicron 6237
Bacteroides stercoris 5888
Bacteroides salyersiae 2697
Bacteroides plebeius 2436
Bacteroides intestinalis 181
Bacteroides fragilis JIM10
Bacteroides fragilis BOB25
Bacteroides finegoldii EBA 6-28
Bacteroides eggerthii 91
Bacteroides dorei EBA 7-24
Bacteroides coprocola EBA 6-21
Bacteroides clarus 606
Bacteroides cellulosilyticus 807
Bacteroides caccae EBA6-24
Species

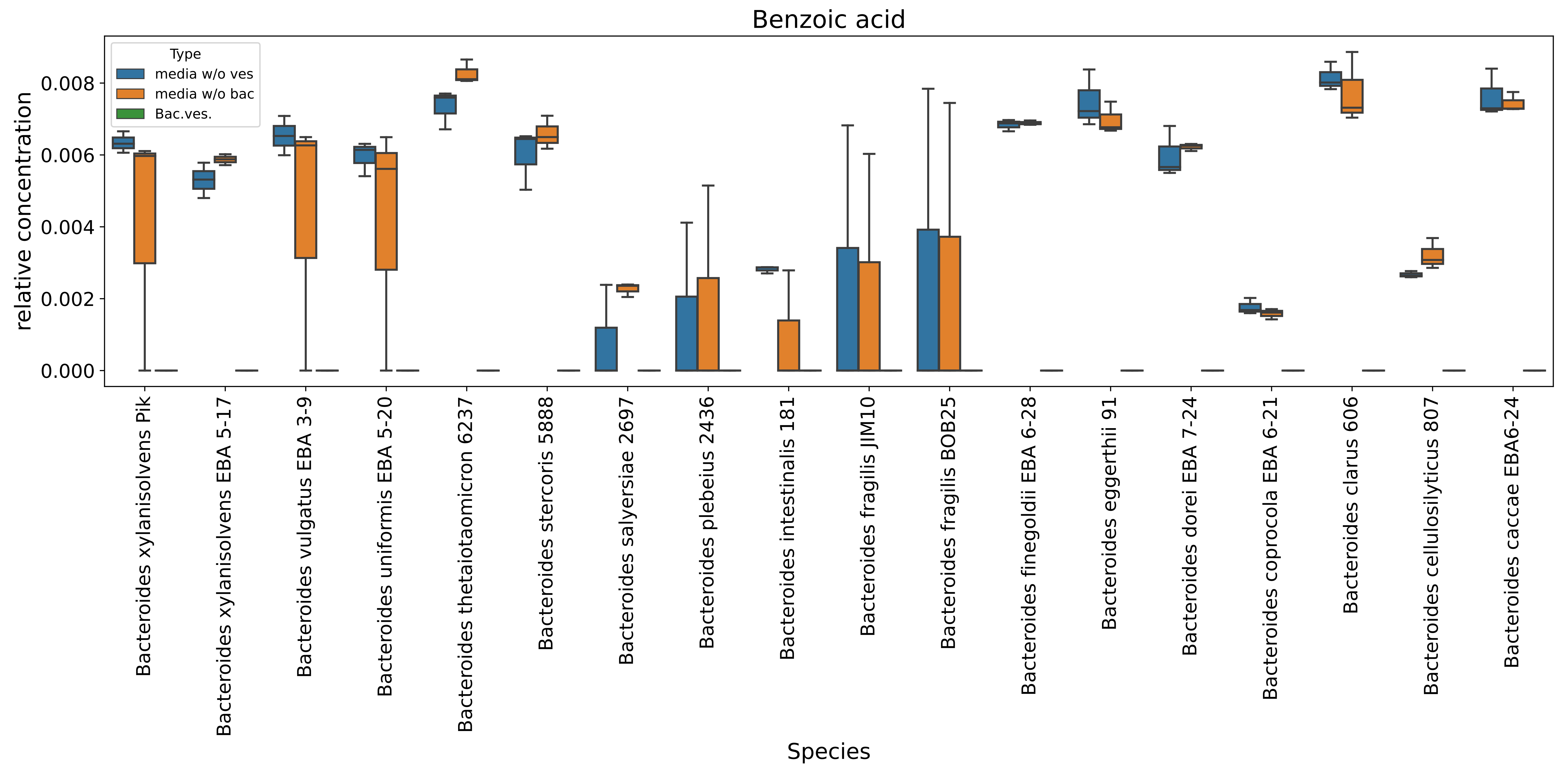
Benzoic acid
Type
media w/o ves
media w/o bac
Bac.ves.
relative concentration
0.000
0.002
0.004
0.006
0.008
Species
Bacteroides xylanisolvens Pik
Bacteroides xylanisolvens EBA 5-17
Bacteroides vulgatus EBA 3-9
Bacteroides uniformis EBA 5-20
Bacteroides thetaiotaomicron 6237
Bacteroides stercoris 5888
Bacteroides salyersiae 2697
Bacteroides plebeius 2436
Bacteroides intestinalis 181
Bacteroides fragilis JIM10
Bacteroides fragilis BOB25
Bacteroides finegoldii EBA 6-28
Bacteroides eggerthii 91
Bacteroides dorei EBA 7-24
Bacteroides coprocola EBA 6-21
Bacteroides clarus 606
Bacteroides cellulosilyticus 807
Bacteroides caccae EBA6-24

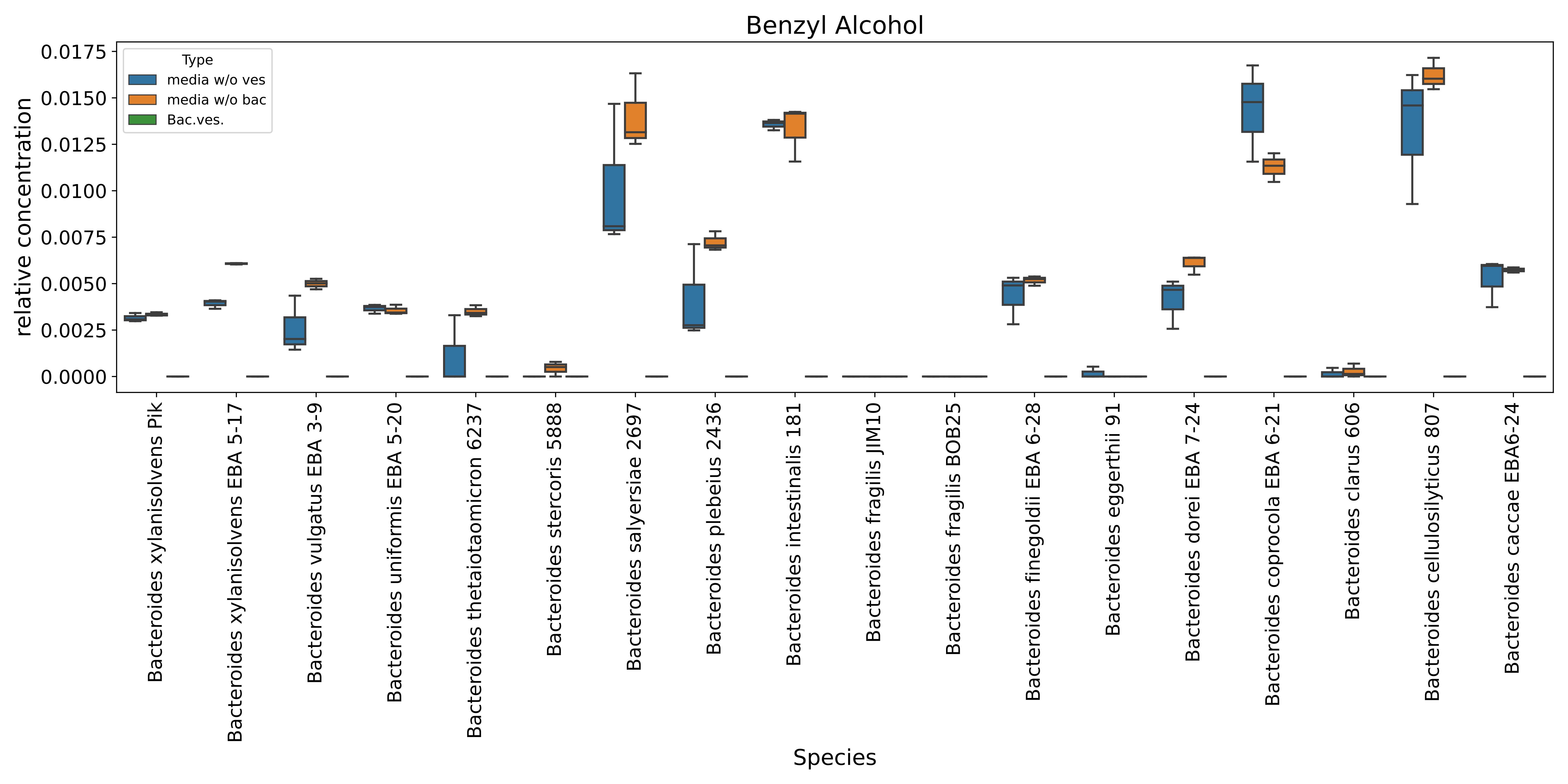

Benzyl Alcohol
Type
media w/o ves
media w/o bac
Bac.ves.
relative concentration
0.0175
0.0150
0.0125
0.0100
0.0075
0.0050
0.0025
0.0000
Bacteroides xylanisolvens Pik
Bacteroides xylanisolvens EBA 5-17
Bacteroides vulgatus EBA 3-9
Bacteroides uniformis EBA 5-20
Bacteroides thetaiotaomicron 6237
Bacteroides stercoris 5888
Bacteroides salyersiae 2697
Bacteroides plebeius 2436
Bacteroides intestinalis 181
Bacteroides fragilis JIM10
Bacteroides fragilis BOB25
Bacteroides finegoldii EBA 6-28
Bacteroides eggerthii 91
Bacteroides dorei EBA 7-24
Bacteroides coprocola EBA 6-21
Bacteroides clarus 606
Bacteroides cellulosilyticus 807
Bacteroides caccae EBA6-24
Species

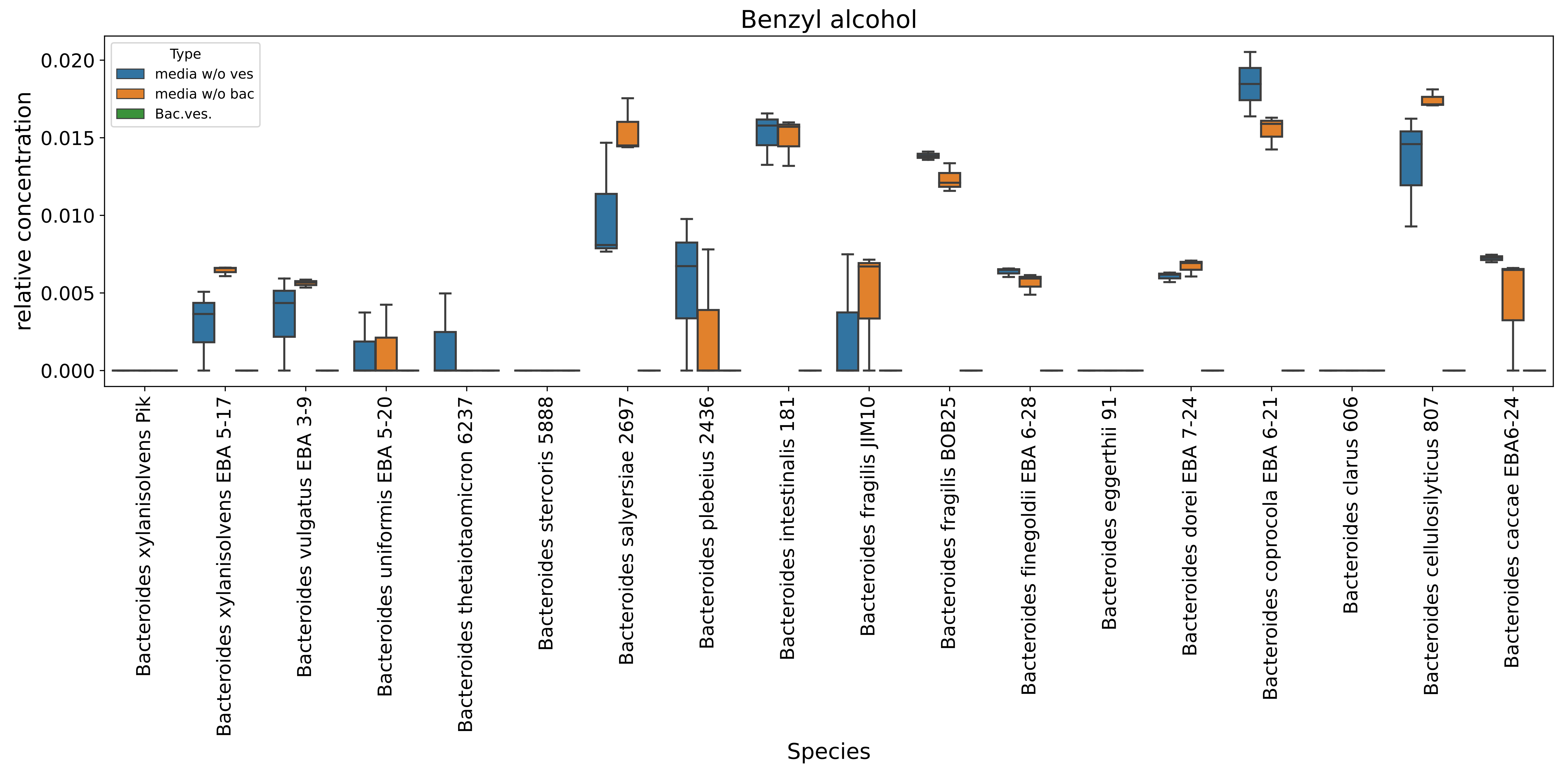

Benzyl alcohol
Type
media w/o ves
media w/o bac
Bac.ves.
relative concentration
0.020
0.015
0.010
0.005
0.000
Bacteroides xylanisolvens Pik
Bacteroides xylanisolvens EBA 5-17
Bacteroides vulgatus EBA 3-9
Bacteroides uniformis EBA 5-20
Bacteroides thetaiotaomicron 6237
Bacteroides stercoris 5888
Bacteroides salyersiae 2697
Bacteroides plebeius 2436
Bacteroides intestinalis 181
Bacteroides fragilis JIM10
Bacteroides fragilis BOB25
Bacteroides finegoldii EBA 6-28
Bacteroides eggerthii 91
Bacteroides dorei EBA 7-24
Bacteroides coprocola EBA 6-21
Bacteroides clarus 606
Bacteroides cellulosilyticus 807
Bacteroides caccae EBA6-24
Species

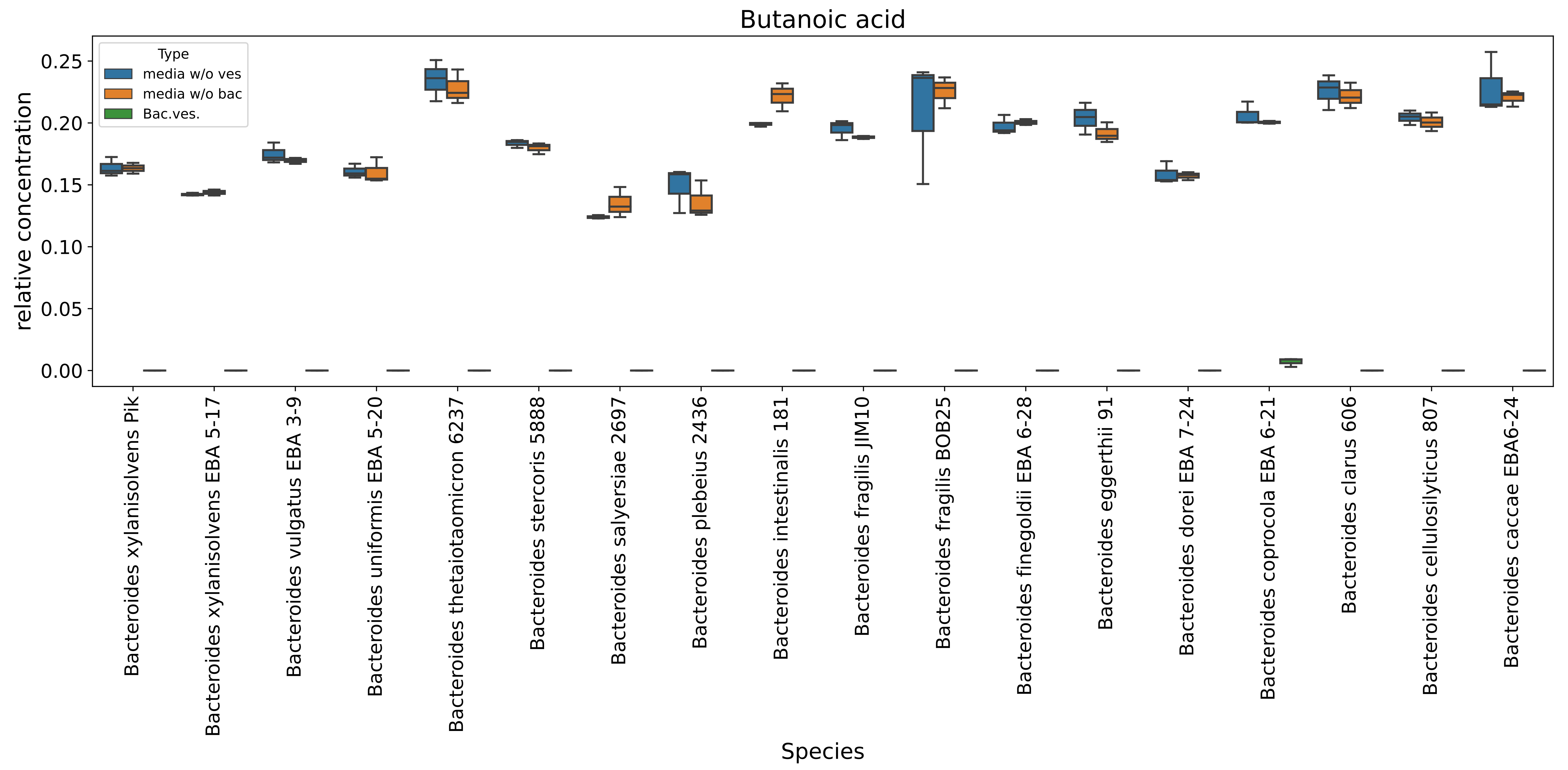

Butanoic acid
Type
media w/o ves
media w/o bac
Bac.ves.
relative concentration
0.25
0.20
0.15
0.10
0.05
0.00
Bacteroides xylanisolvens Pik
Bacteroides xylanisolvens EBA 5-17
Bacteroides vulgatus EBA 3-9
Bacteroides uniformis EBA 5-20
Bacteroides thetaiotaomicron 6237
Bacteroides stercoris 5888
Bacteroides salyersiae 2697
Bacteroides plebeius 2436
Bacteroides intestinalis 181
Bacteroides fragilis JIM10
Bacteroides fragilis BOB25
Bacteroides finegoldii EBA 6-28
Bacteroides eggerthii 91
Bacteroides dorei EBA 7-24
Bacteroides coprocola EBA 6-21
Bacteroides clarus 606
Bacteroides cellulosilyticus 807
Bacteroides caccae EBA6-24
Species

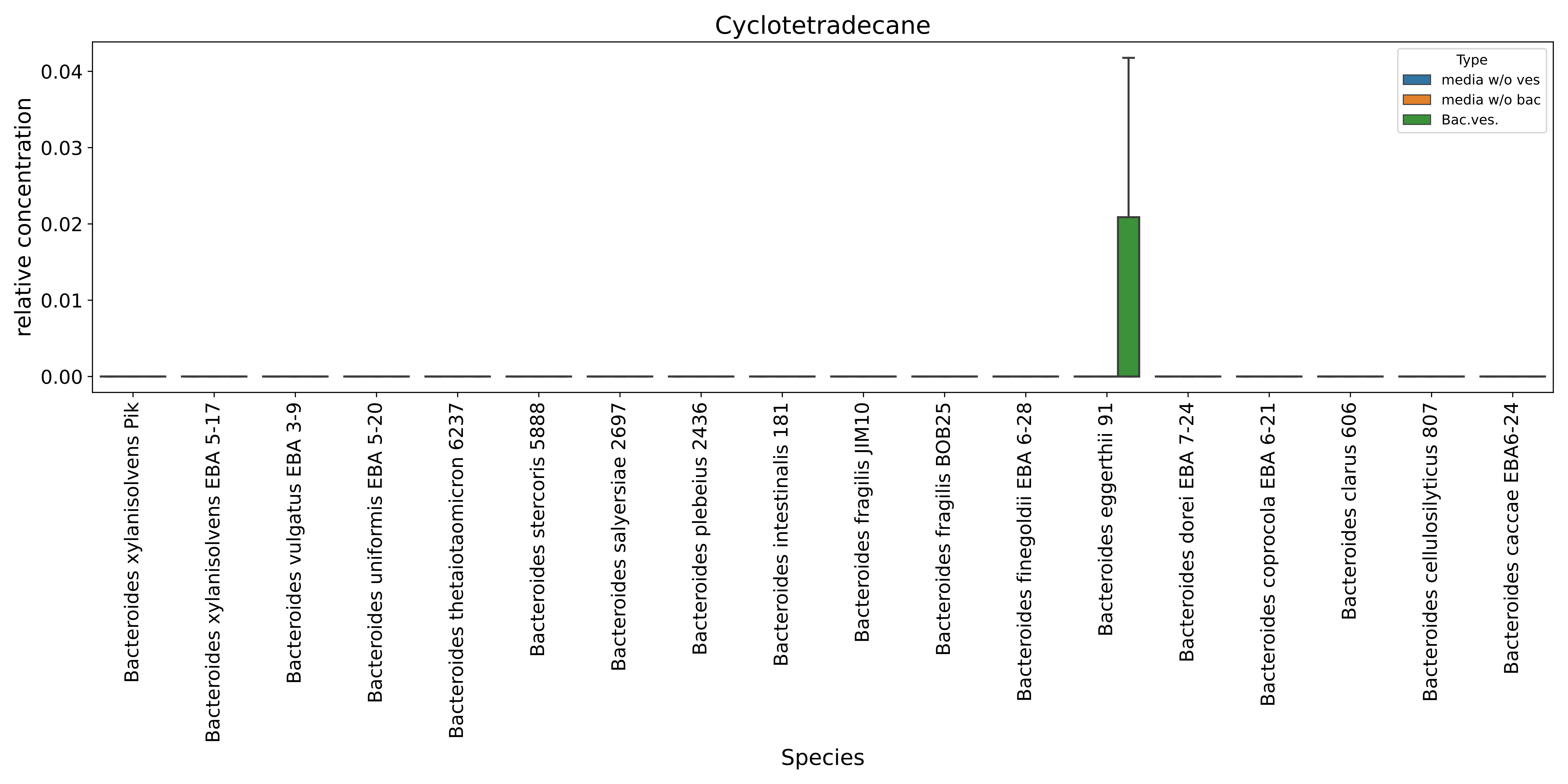
Cyclotetradecane
relative concentration
0.04
0.03
0.02
0.01
0.00
Type
media w/o ves
media w/o bac
Bac.ves.
Bacteroides xylanisolvens Pik
Bacteroides xylanisolvens EBA 5-17
Bacteroides vulgatus EBA 3-9
Bacteroides uniformis EBA 5-20
Bacteroides thetaiotaomicron 6237
Bacteroides stercoris 5888
Bacteroides salyersiae 2697
Bacteroides plebeius 2436
Bacteroides intestinalis 181
Bacteroides fragilis JIM10
Bacteroides fragilis BOB25
Bacteroides finegoldii EBA 6-28
Bacteroides eggerthii 91
Bacteroides dorei EBA 7-24
Bacteroides coprocola EBA 6-21
Bacteroides clarus 606
Bacteroides cellulosilyticus 807
Bacteroides caccae EBA6-24
Species

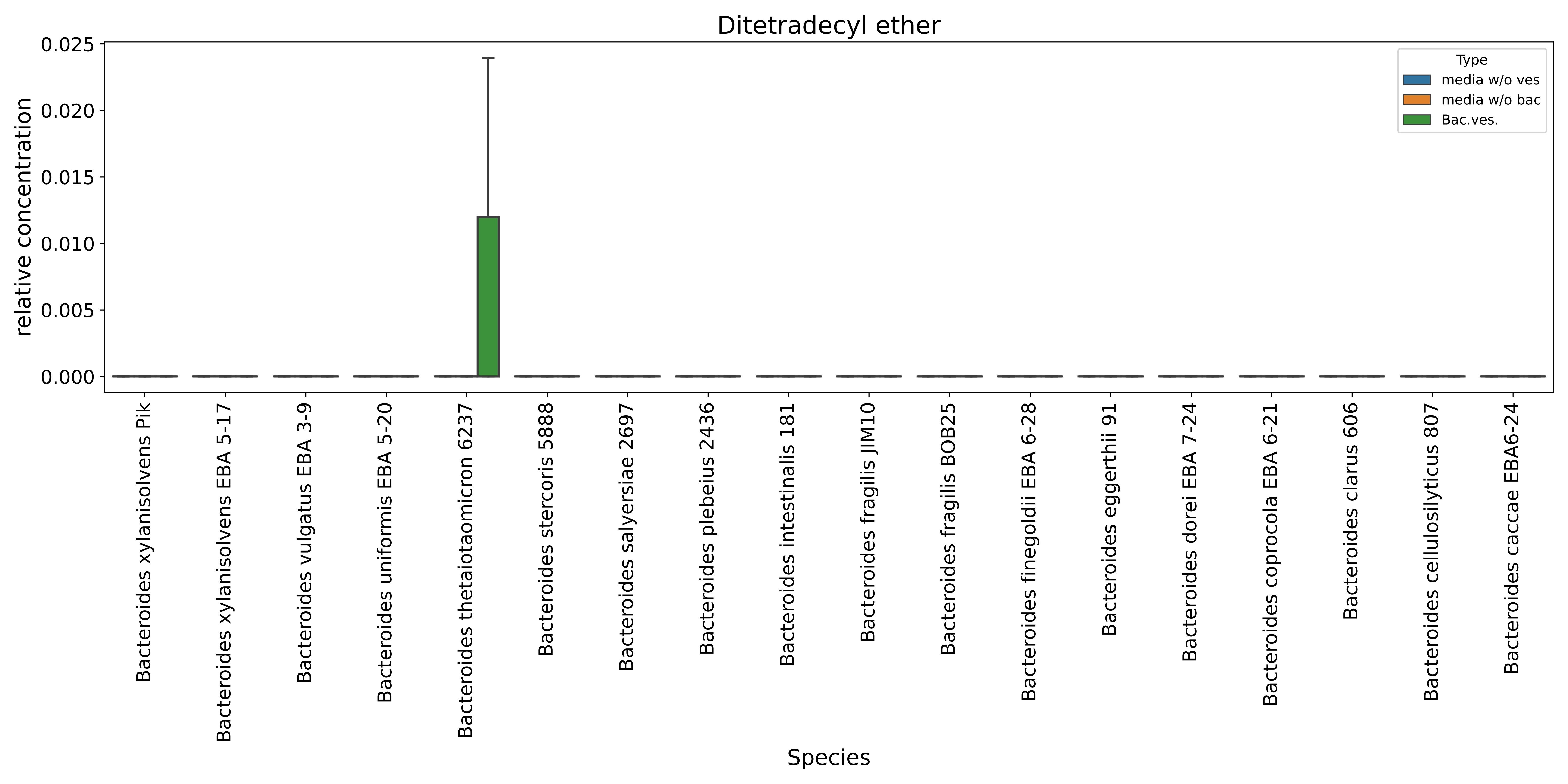

Ditetradecyl ether
relative concentration
0.025
0.020
0.015
0.010
0.005
0.000
Type
media w/o ves
media w/o bac
Bac.ves.
Bacteroides xylanisolvens Pik
Bacteroides xylanisolvens EBA 5-17
Bacteroides vulgatus EBA 3-9
Bacteroides uniformis EBA 5-20
Bacteroides thetaiotaomicron 6237
Bacteroides stercoris 5888
Bacteroides salyersiae 2697
Bacteroides plebeius 2436
Bacteroides intestinalis 181
Bacteroides fragilis JIM10
Bacteroides fragilis BOB25
Bacteroides finegoldii EBA 6-28
Bacteroides eggerthii 91
Bacteroides dorei EBA 7-24
Bacteroides coprocola EBA 6-21
Bacteroides clarus 606
Bacteroides cellulosilyticus 807
Bacteroides caccae EBA6-24
Species

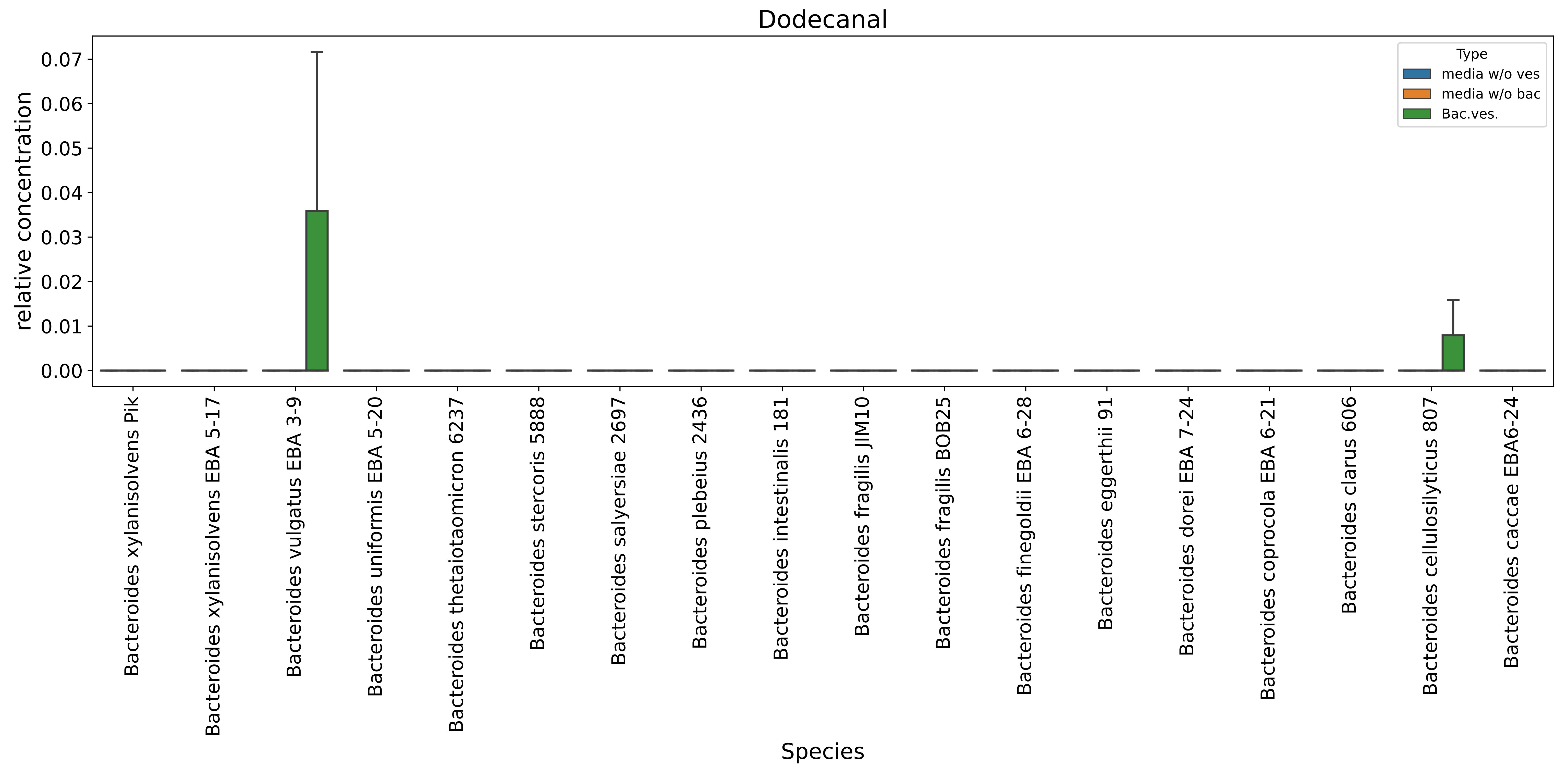

Dodecanal
relative concentration
0.07
0.06
0.05
0.04
0.03
0.02
0.01
0.00
Type
media w/o ves
media w/o bac
Bac.ves.
Bacteroides xylanisolvens Pik
Bacteroides xylanisolvens EBA 5-17
Bacteroides vulgatus EBA 3-9
Bacteroides uniformis EBA 5-20
Bacteroides thetaiotaomicron 6237
Bacteroides stercoris 5888
Bacteroides salyersiae 2697
Bacteroides plebeius 2436
Bacteroides intestinalis 181
Bacteroides fragilis JIM10
Bacteroides fragilis BOB25
Bacteroides finegoldii EBA 6-28
Bacteroides eggerthii 91
Bacteroides dorei EBA 7-24
Bacteroides coprocola EBA 6-21
Bacteroides clarus 606
Bacteroides cellulosilyticus 807
Bacteroides caccae EBA6-24
Species

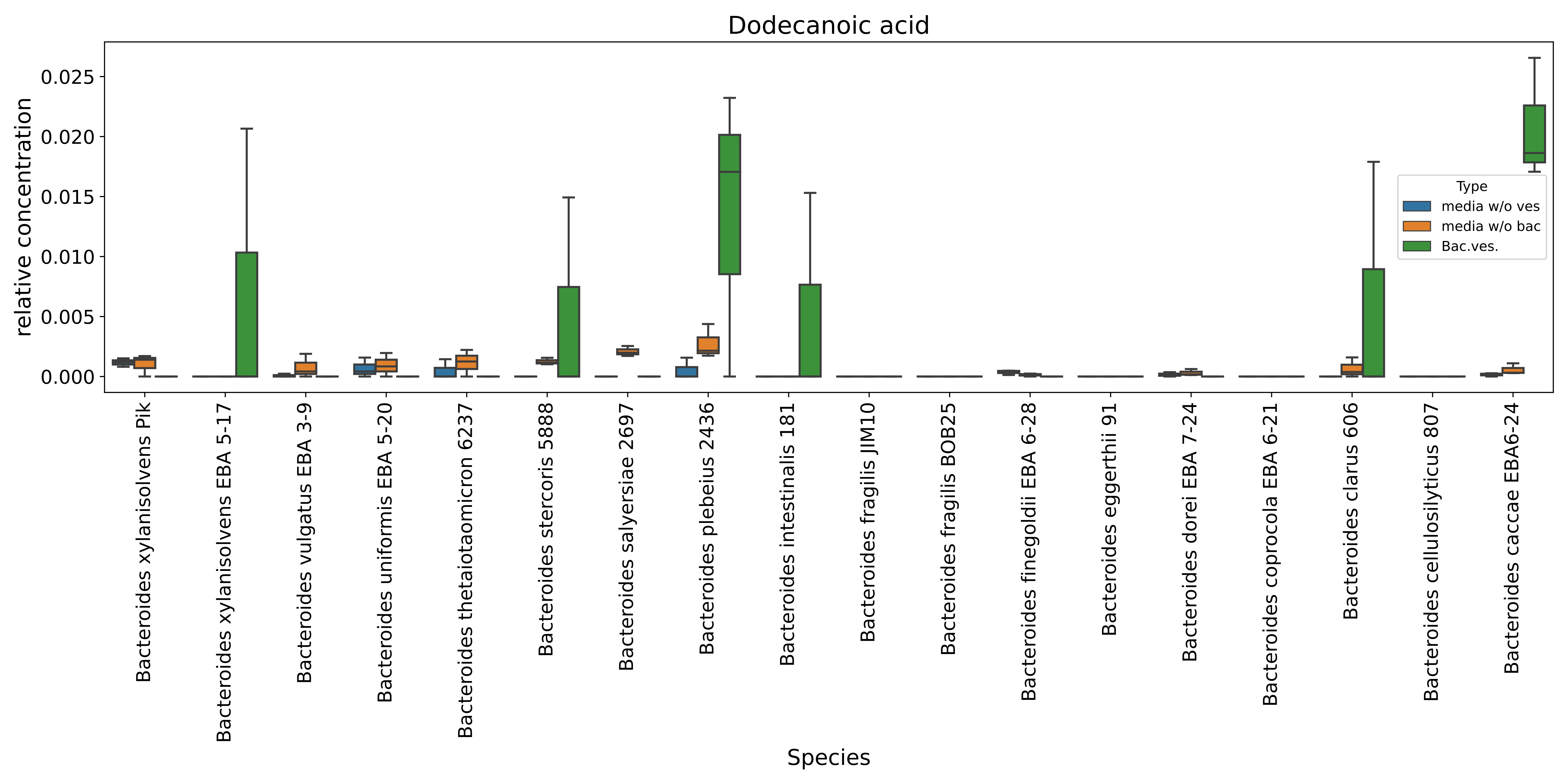
Dodecanoic acid
relative concentration
0.025
0.020
0.015
0.010
0.005
0.000
Type
media w/o ves
media w/o bac
Bac.ves.
Bacteroides xylanisolvens Pik
Bacteroides xylanisolvens EBA 5-17
Bacteroides vulgatus EBA 3-9
Bacteroides uniformis EBA 5-20
Bacteroides thetaiotaomicron 6237
Bacteroides stercoris 5888
Bacteroides salyersiae 2697
Bacteroides plebeius 2436
Bacteroides intestinalis 181
Bacteroides fragilis JIM10
Bacteroides fragilis BOB25
Bacteroides finegoldii EBA 6-28
Bacteroides eggerthii 91
Bacteroides dorei EBA 7-24
Bacteroides coprocola EBA 6-21
Bacteroides clarus 606
Bacteroides cellulosilyticus 807
Bacteroides caccae EBA6-24
Species

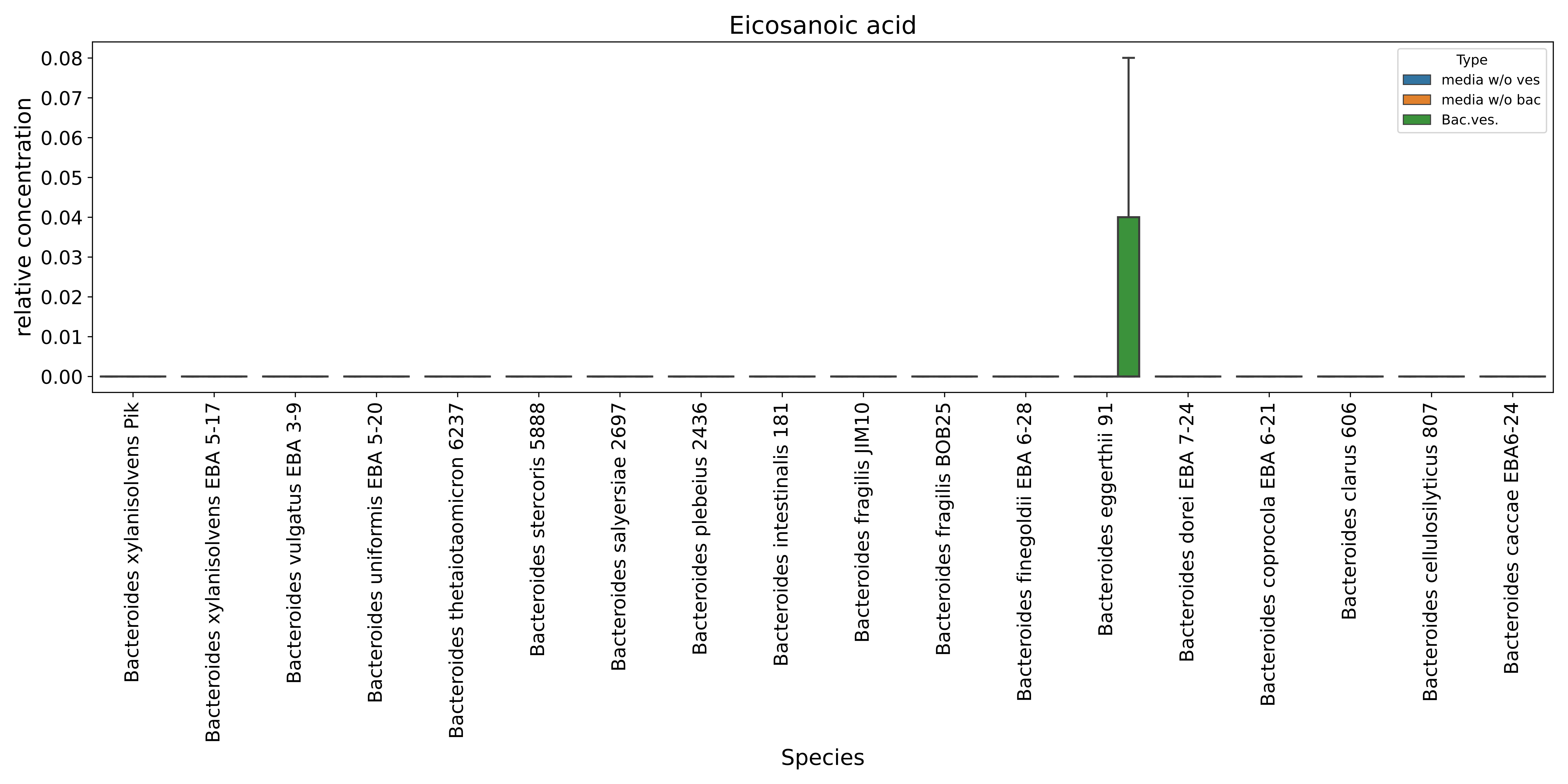
Eicosanoic acid
relative concentration
0.08
0.07
0.06
0.05
0.04
0.03
0.02
0.01
0.00
Type
media w/o ves
media w/o bac
Bac.ves.
Bacteroides xylanisolvens Pik
Bacteroides xylanisolvens EBA 5-17
Bacteroides vulgatus EBA 3-9
Bacteroides uniformis EBA 5-20
Bacteroides thetaiotaomicron 6237
Bacteroides stercoris 5888
Bacteroides salyersiae 2697
Bacteroides plebeius 2436
Bacteroides intestinalis 181
Bacteroides fragilis JIM10
Bacteroides fragilis BOB25
Bacteroides finegoldii EBA 6-28
Bacteroides eggerthii 91
Bacteroides dorei EBA 7-24
Bacteroides coprocola EBA 6-21
Bacteroides clarus 606
Bacteroides cellulosilyticus 807
Bacteroides caccae EBA6-24
Species

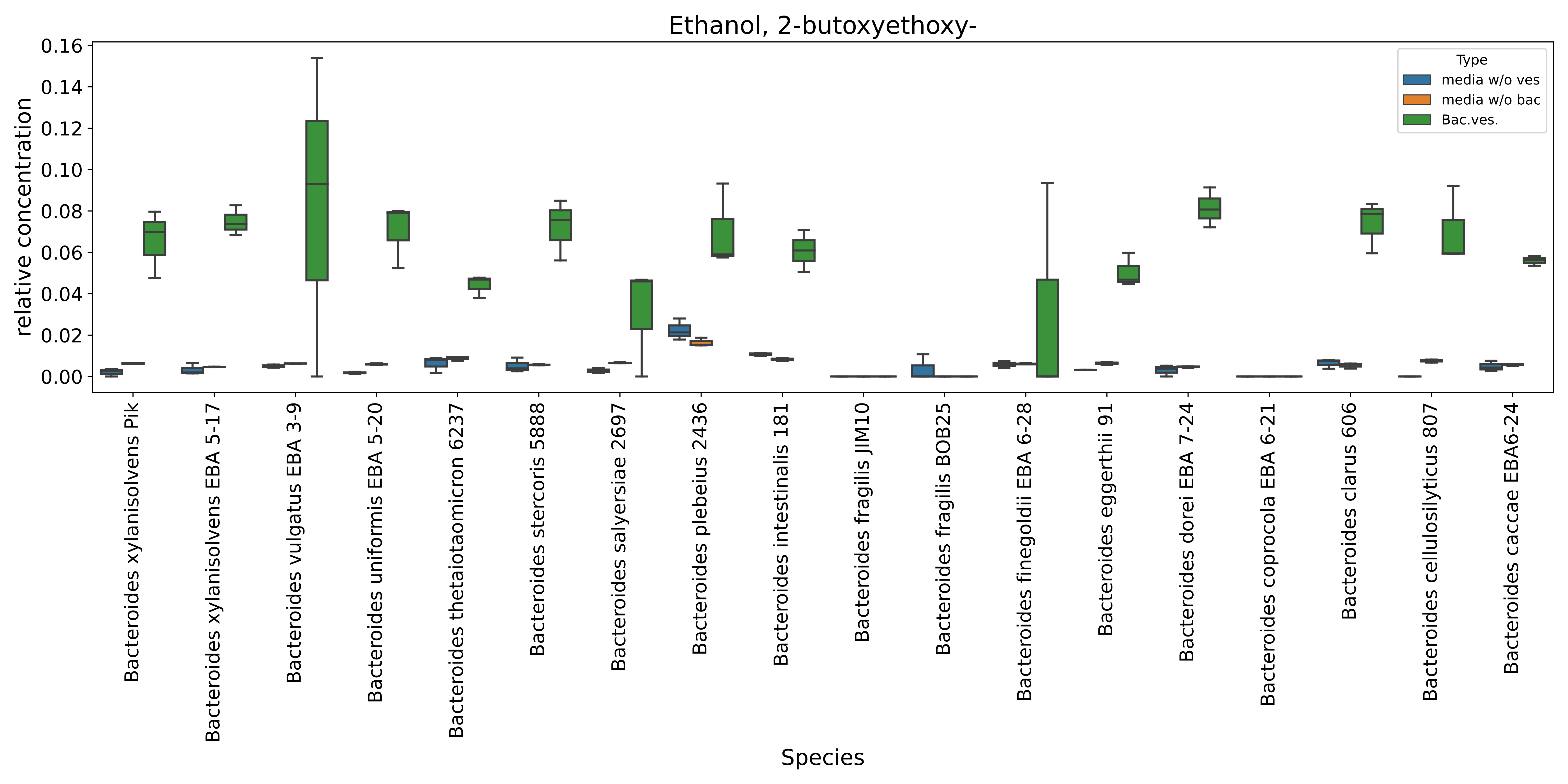

Ethanol, 2-butoxyethoxy-
relative concentration
0.16
0.14
0.12
0.10
0.08
0.06
0.04
0.02
0.00
Type
media w/o ves
media w/o bac
Bac.ves.
Bacteroides xylanisolvens Pik
Bacteroides xylanisolvens EBA 5-17
Bacteroides vulgatus EBA 3-9
Bacteroides uniformis EBA 5-20
Bacteroides thetaiotaomicron 6237
Bacteroides stercoris 5888
Bacteroides salyersiae 2697
Bacteroides plebeius 2436
Bacteroides intestinalis 181
Bacteroides fragilis JIM10
Bacteroides fragilis BOB25
Bacteroides finegoldii EBA 6-28
Bacteroides eggerthii 91
Bacteroides dorei EBA 7-24
Bacteroides coprocola EBA 6-21
Bacteroides clarus 606
Bacteroides cellulosilyticus 807
Bacteroides caccae EBA6-24
Species

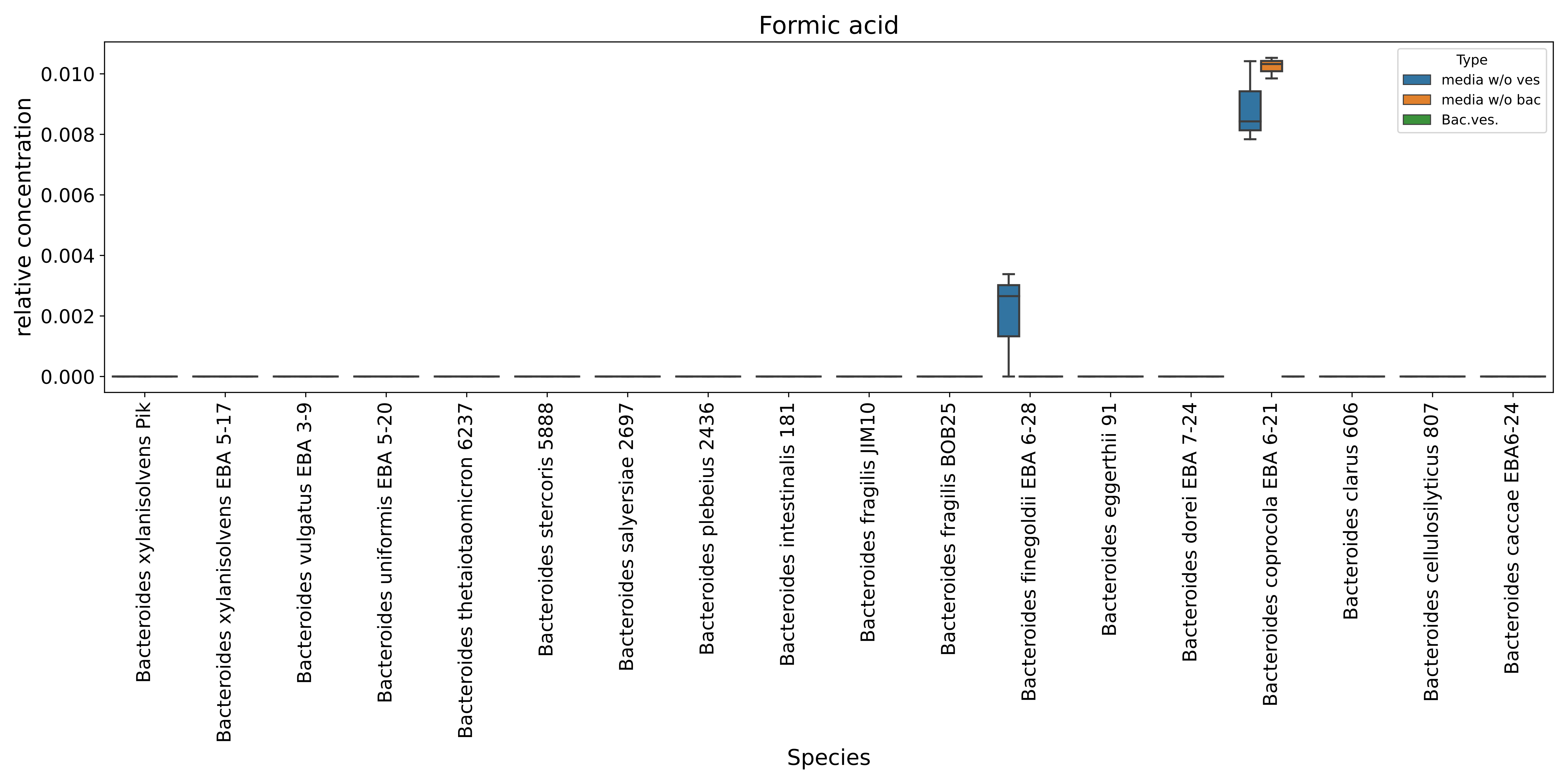

Formic acid
relative concentration
0.010
0.008
0.006
0.004
0.002
0.000
Type
media w/o ves
media w/o bac
Bac.ves.
Bacteroides xylanisolvens Pik
Bacteroides xylanisolvens EBA 5-17
Bacteroides vulgatus EBA 3-9
Bacteroides uniformis EBA 5-20
Bacteroides thetaiotaomicron 6237
Bacteroides stercoris 5888
Bacteroides salyersiae 2697
Bacteroides plebeius 2436
Bacteroides intestinalis 181
Bacteroides fragilis JIM10
Bacteroides fragilis BOB25
Bacteroides finegoldii EBA 6-28
Bacteroides eggerthii 91
Bacteroides dorei EBA 7-24
Bacteroides coprocola EBA 6-21
Bacteroides clarus 606
Bacteroides cellulosilyticus 807
Bacteroides caccae EBA6-24
Species

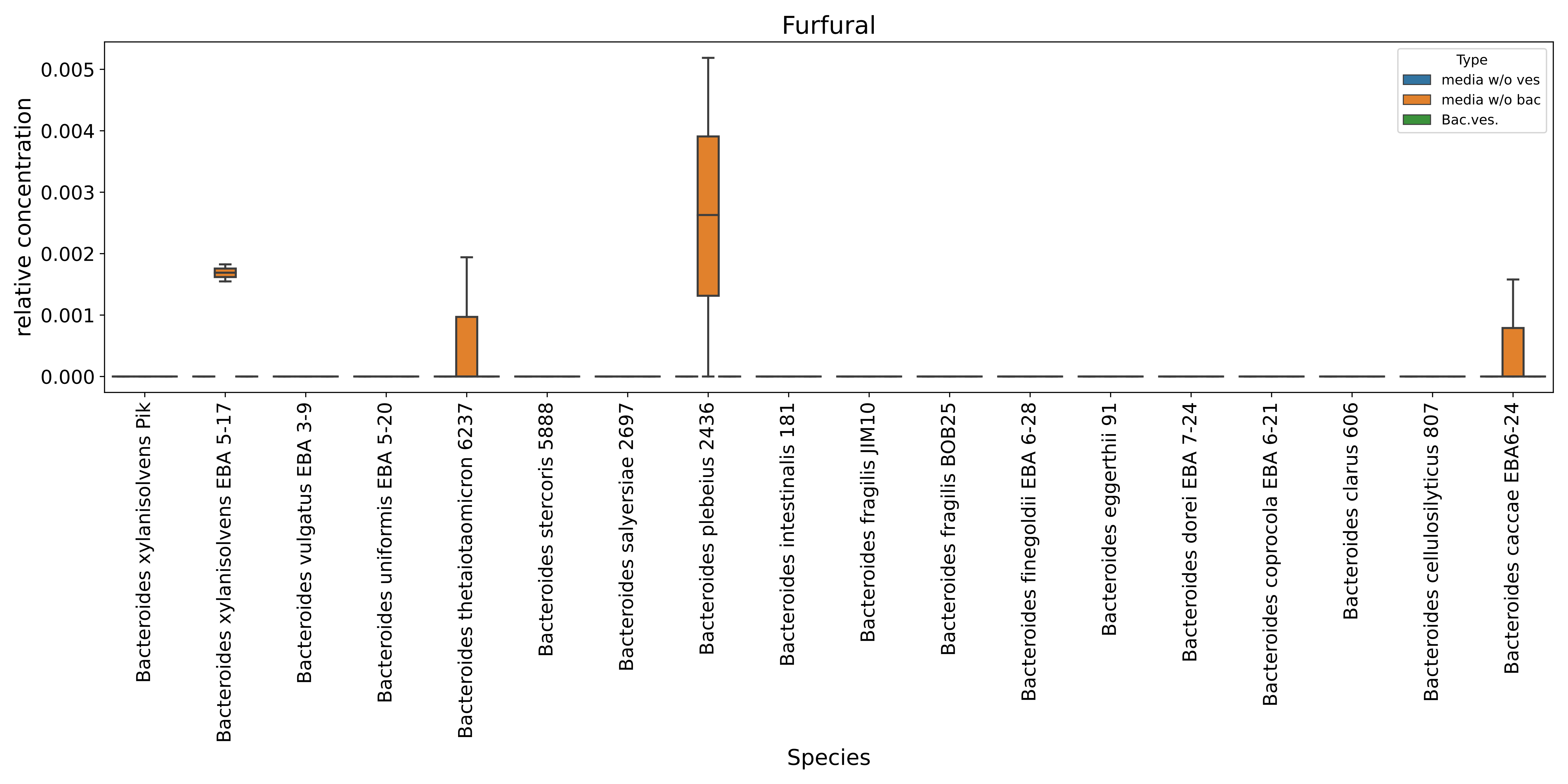
Furfural
relative concentration
0.005
0.004
0.003
0.002
0.001
0.000
Type
media w/o ves
media w/o bac
Bac.ves.
Bacteroides xylanisolvens Pik
Bacteroides xylanisolvens EBA 5-17
Bacteroides vulgatus EBA 3-9
Bacteroides uniformis EBA 5-20
Bacteroides thetaiotaomicron 6237
Bacteroides stercoris 5888
Bacteroides salyersiae 2697
Bacteroides plebeius 2436
Bacteroides intestinalis 181
Bacteroides fragilis JIM10
Bacteroides fragilis BOB25
Bacteroides finegoldii EBA 6-28
Bacteroides eggerthii 91
Bacteroides dorei EBA 7-24
Bacteroides coprocola EBA 6-21
Bacteroides clarus 606
Bacteroides cellulosilyticus 807
Bacteroides caccae EBA6-24
Species

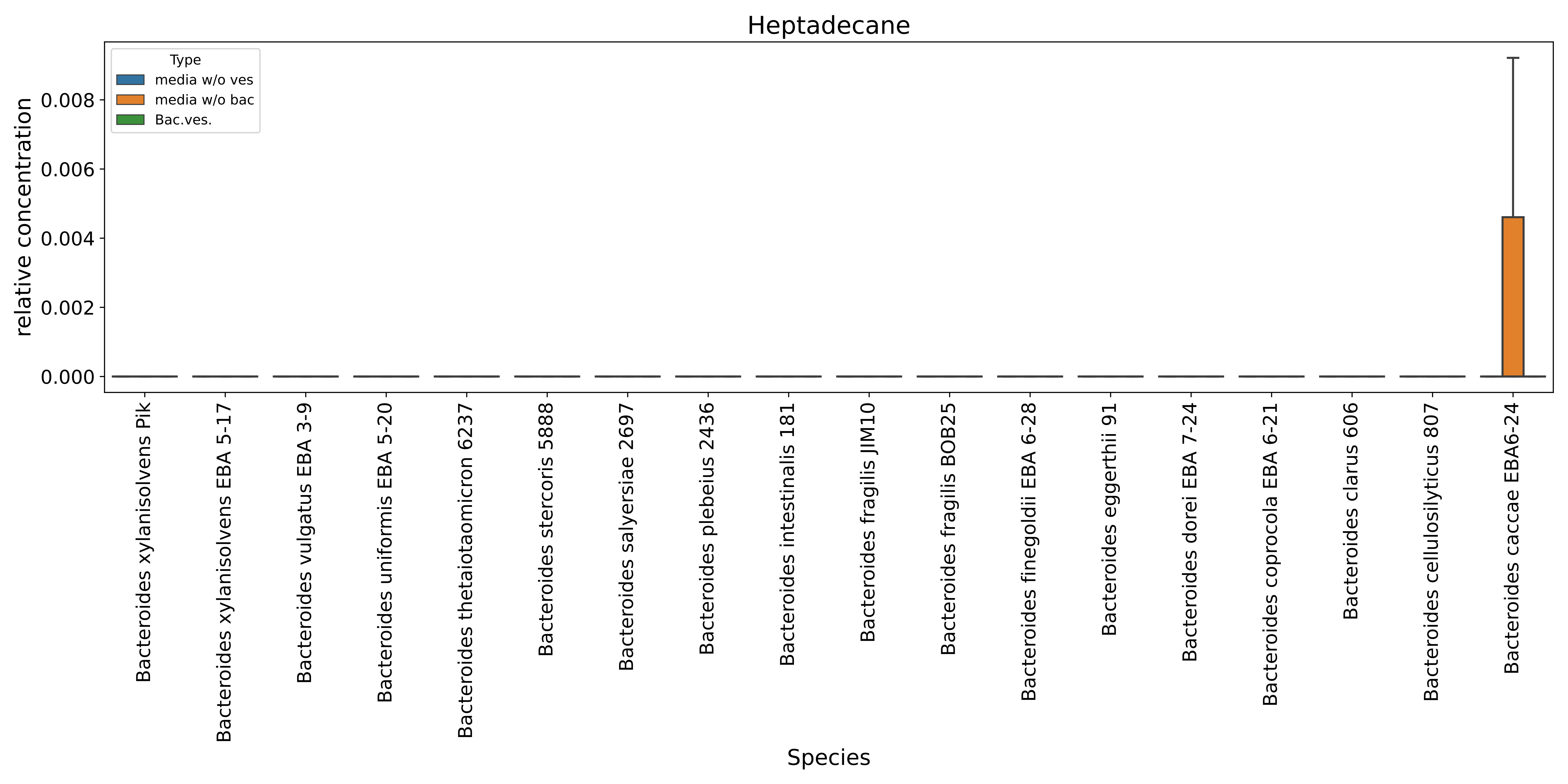

Heptadecane
Type
media w/o ves
media w/o bac
Bac.ves.
relative concentration
0.008
0.006
0.004
0.002
0.000
Bacteroides xylanisolvens Pik
Bacteroides xylanisolvens EBA 5-17
Bacteroides vulgatus EBA 3-9
Bacteroides uniformis EBA 5-20
Bacteroides thetaiotaomicron 6237
Bacteroides stercoris 5888
Bacteroides salyersiae 2697
Bacteroides plebeius 2436
Bacteroides intestinalis 181
Bacteroides fragilis JIM10
Bacteroides fragilis BOB25
Bacteroides finegoldii EBA 6-28
Bacteroides eggerthii 91
Bacteroides dorei EBA 7-24
Bacteroides coprocola EBA 6-21
Bacteroides clarus 606
Bacteroides cellulosilyticus 807
Bacteroides caccae EBA6-24
Species

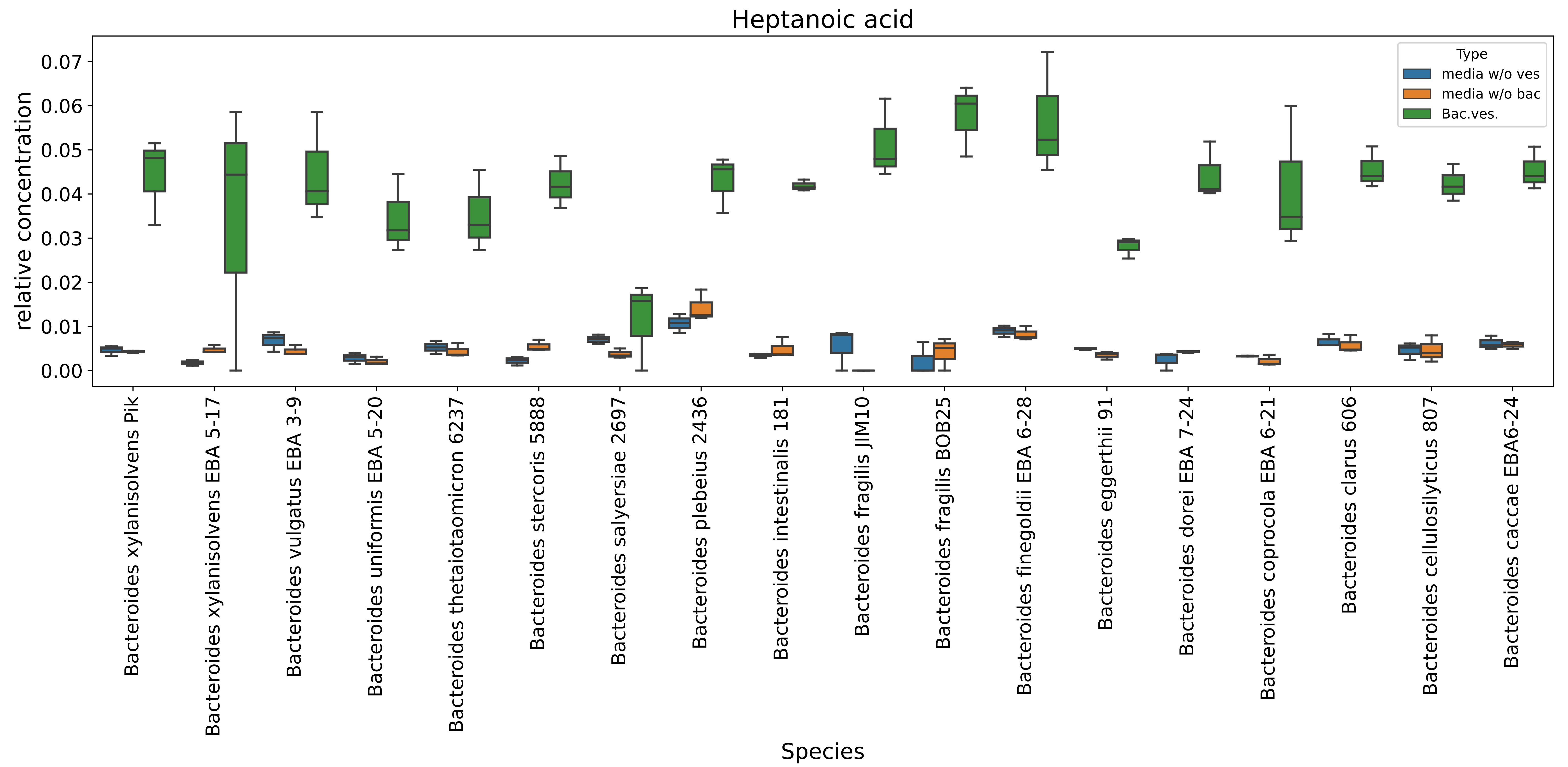

Heptanoic acid
relative concentration
0.00
0.01
0.02
0.03
0.04
0.05
0.06
0.07
Type
media w/o ves
media w/o bac
Bac.ves.
Bacteroides xylanisolvens Pik
Bacteroides xylanisolvens EBA 5-17
Bacteroides vulgatus EBA 3-9
Bacteroides uniformis EBA 5-20
Bacteroides thetaiotaomicron 6237
Bacteroides stercoris 5888
Bacteroides salyersiae 2697
Bacteroides plebeius 2436
Bacteroides intestinalis 181
Bacteroides fragilis JIM10
Bacteroides fragilis BOB25
Bacteroides finegoldii EBA 6-28
Bacteroides eggerthii 91
Bacteroides dorei EBA 7-24
Bacteroides coprocola EBA 6-21
Bacteroides clarus 606
Bacteroides cellulosilyticus 807
Bacteroides caccae EBA6-24
Species

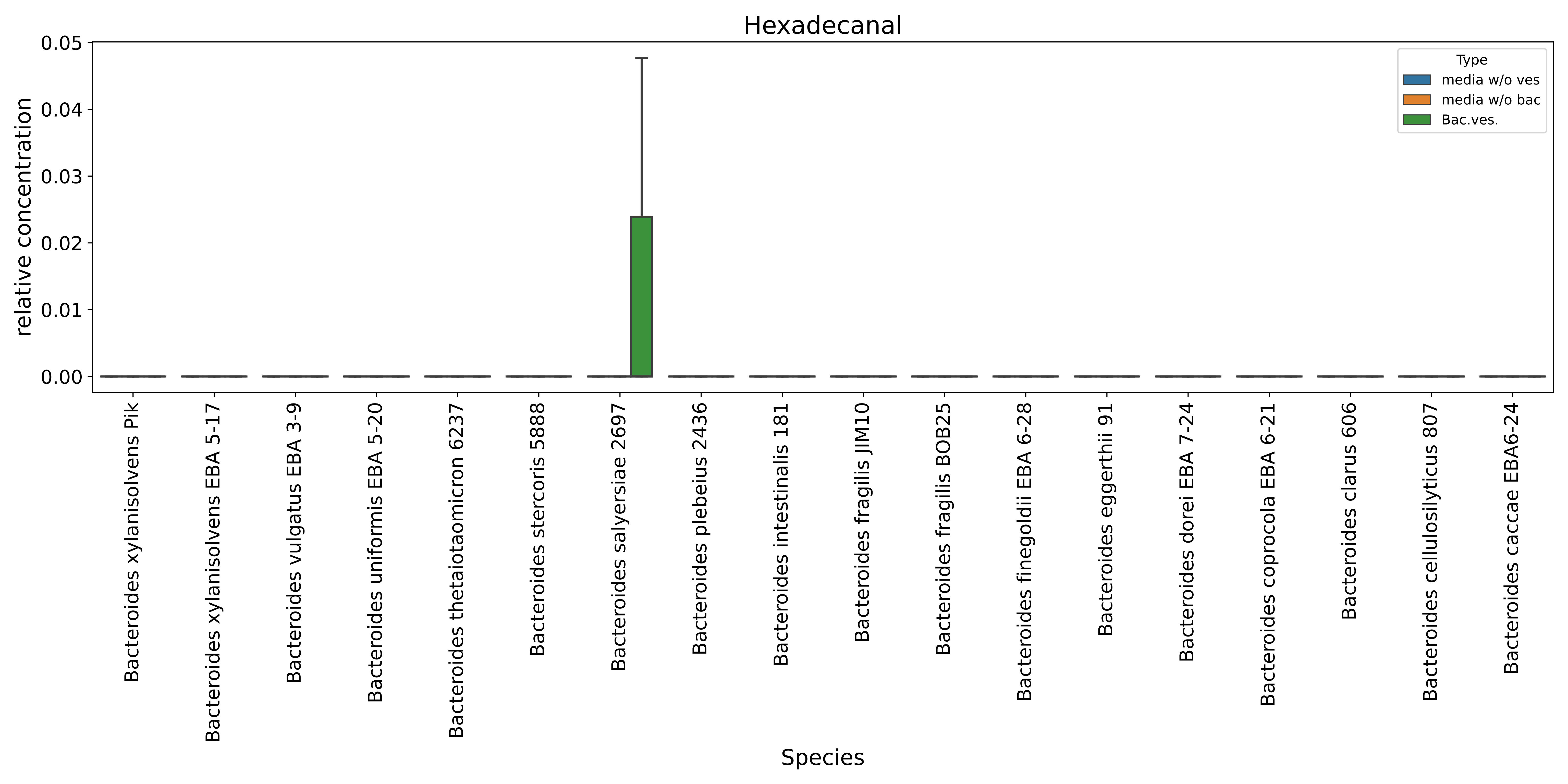
Hexadecanal
relative concentration
0.05
0.04
0.03
0.02
0.01
0.00
Type
media w/o ves
media w/o bac
Bac.ves.
Bacteroides xylanisolvens Pik
Bacteroides xylanisolvens EBA 5-17
Bacteroides vulgatus EBA 3-9
Bacteroides uniformis EBA 5-20
Bacteroides thetaiotaomicron 6237
Bacteroides stercoris 5888
Bacteroides salyersiae 2697
Bacteroides plebeius 2436
Bacteroides intestinalis 181
Bacteroides fragilis JIM10
Bacteroides fragilis BOB25
Bacteroides finegoldii EBA 6-28
Bacteroides eggerthii 91
Bacteroides dorei EBA 7-24
Bacteroides coprocola EBA 6-21
Bacteroides clarus 606
Bacteroides cellulosilyticus 807
Bacteroides caccae EBA6-24
Species

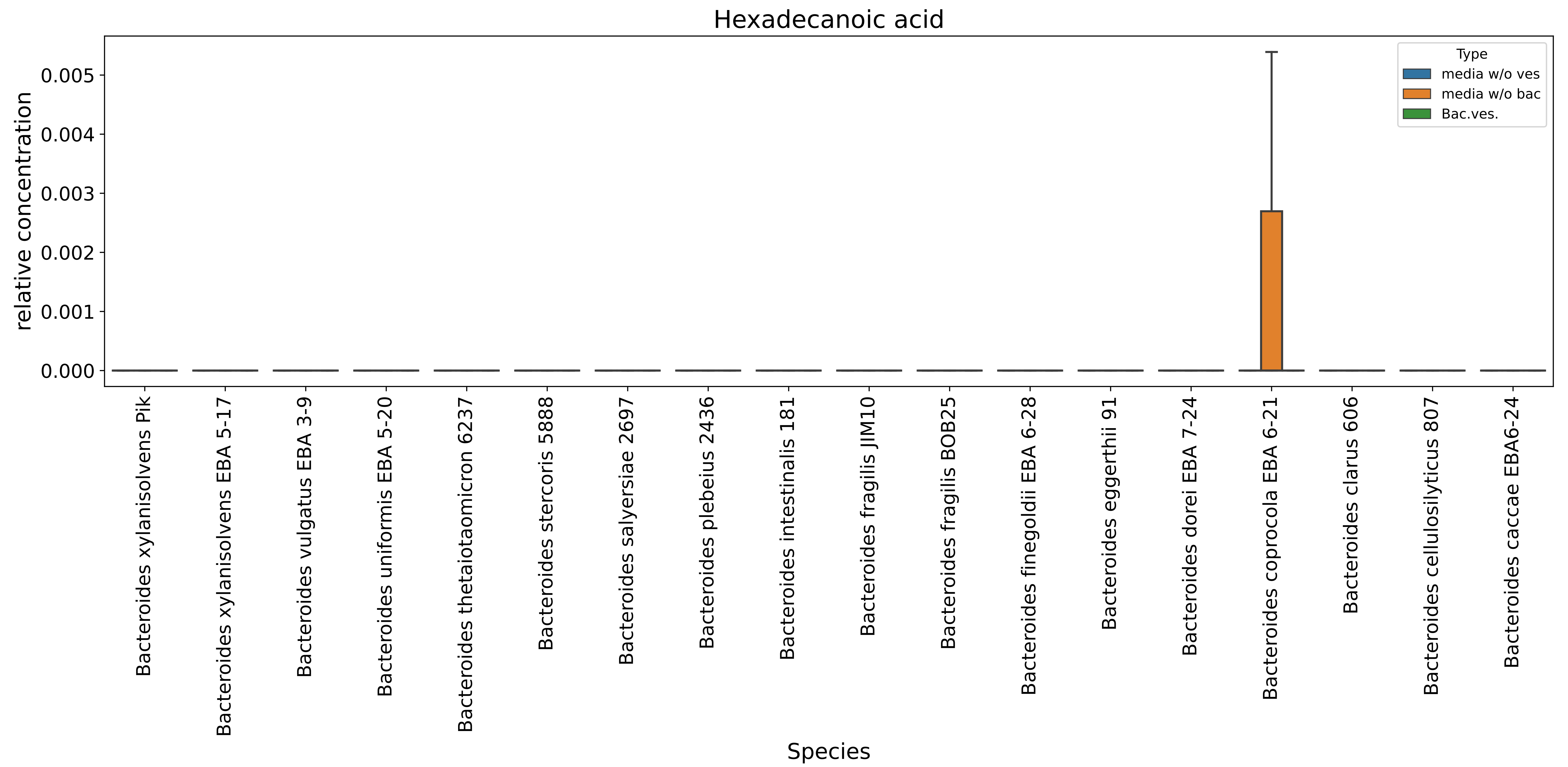

Hexadecanoic acid
relative concentration
0.005
0.004
0.003
0.002
0.001
0.000
Type
media w/o ves
media w/o bac
Bac.ves.
Bacteroides xylanisolvens Pik
Bacteroides xylanisolvens EBA 5-17
Bacteroides vulgatus EBA 3-9
Bacteroides uniformis EBA 5-20
Bacteroides thetaiotaomicron 6237
Bacteroides stercoris 5888
Bacteroides salyersiae 2697
Bacteroides plebeius 2436
Bacteroides intestinalis 181
Bacteroides fragilis JIM10
Bacteroides fragilis BOB25
Bacteroides finegoldii EBA 6-28
Bacteroides eggerthii 91
Bacteroides dorei EBA 7-24
Bacteroides coprocola EBA 6-21
Bacteroides clarus 606
Bacteroides cellulosilyticus 807
Bacteroides caccae EBA6-24
Species

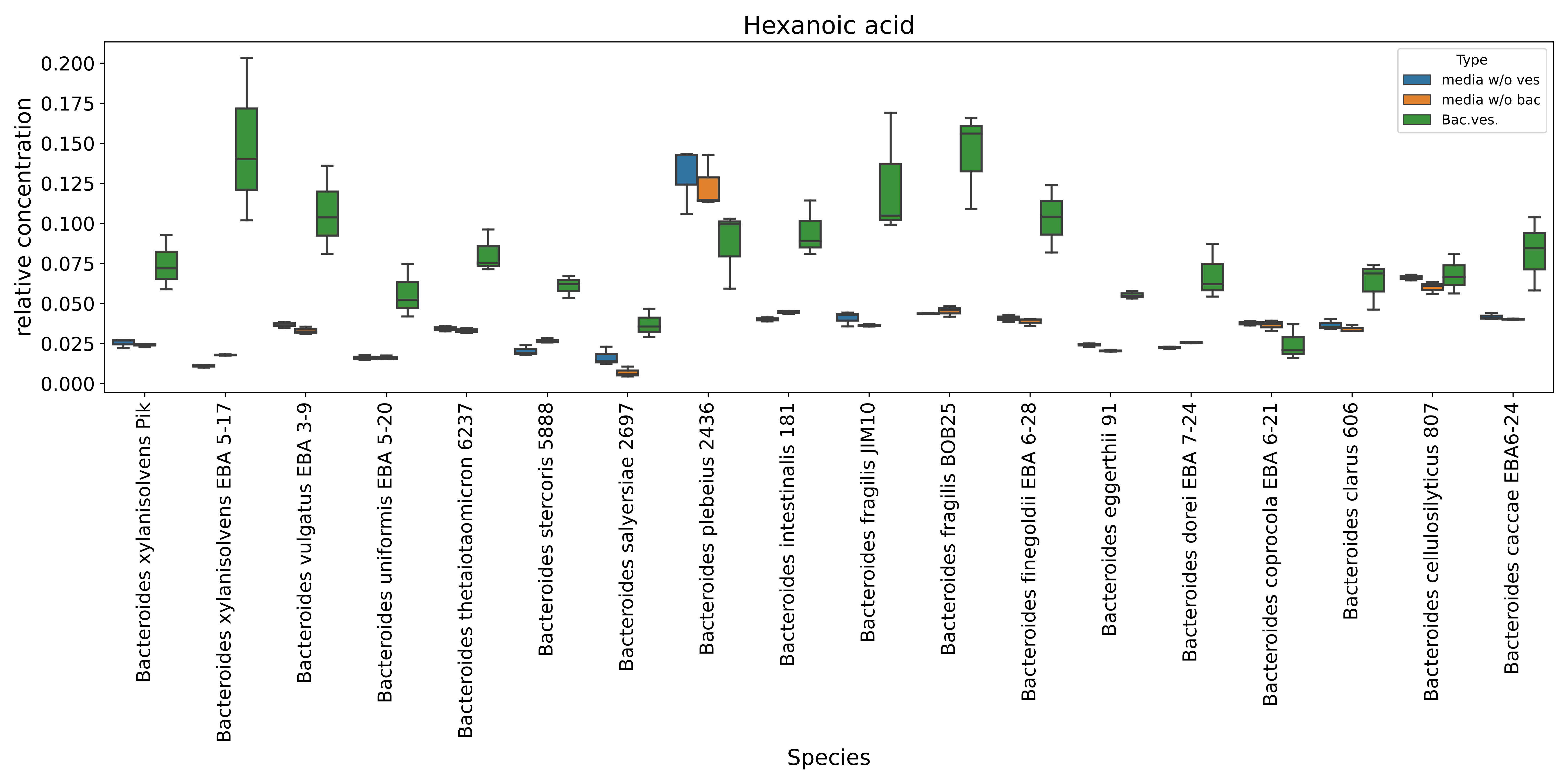

Hexanoic acid
relative concentration
0.200
0.175
0.150
0.125
0.100
0.075
0.050
0.025
0.000
Type
media w/o ves
media w/o bac
Bac.ves.
Bacteroides xylanisolvens Pik
Bacteroides xylanisolvens EBA 5-17
Bacteroides vulgatus EBA 3-9
Bacteroides uniformis EBA 5-20
Bacteroides thetaiotaomicron 6237
Bacteroides stercoris 5888
Bacteroides salyersiae 2697
Bacteroides plebeius 2436
Bacteroides intestinalis 181
Bacteroides fragilis JIM10
Bacteroides fragilis BOB25
Bacteroides finegoldii EBA 6-28
Bacteroides eggerthii 91
Bacteroides dorei EBA 7-24
Bacteroides coprocola EBA 6-21
Bacteroides clarus 606
Bacteroides cellulosilyticus 807
Bacteroides caccae EBA6-24
Species

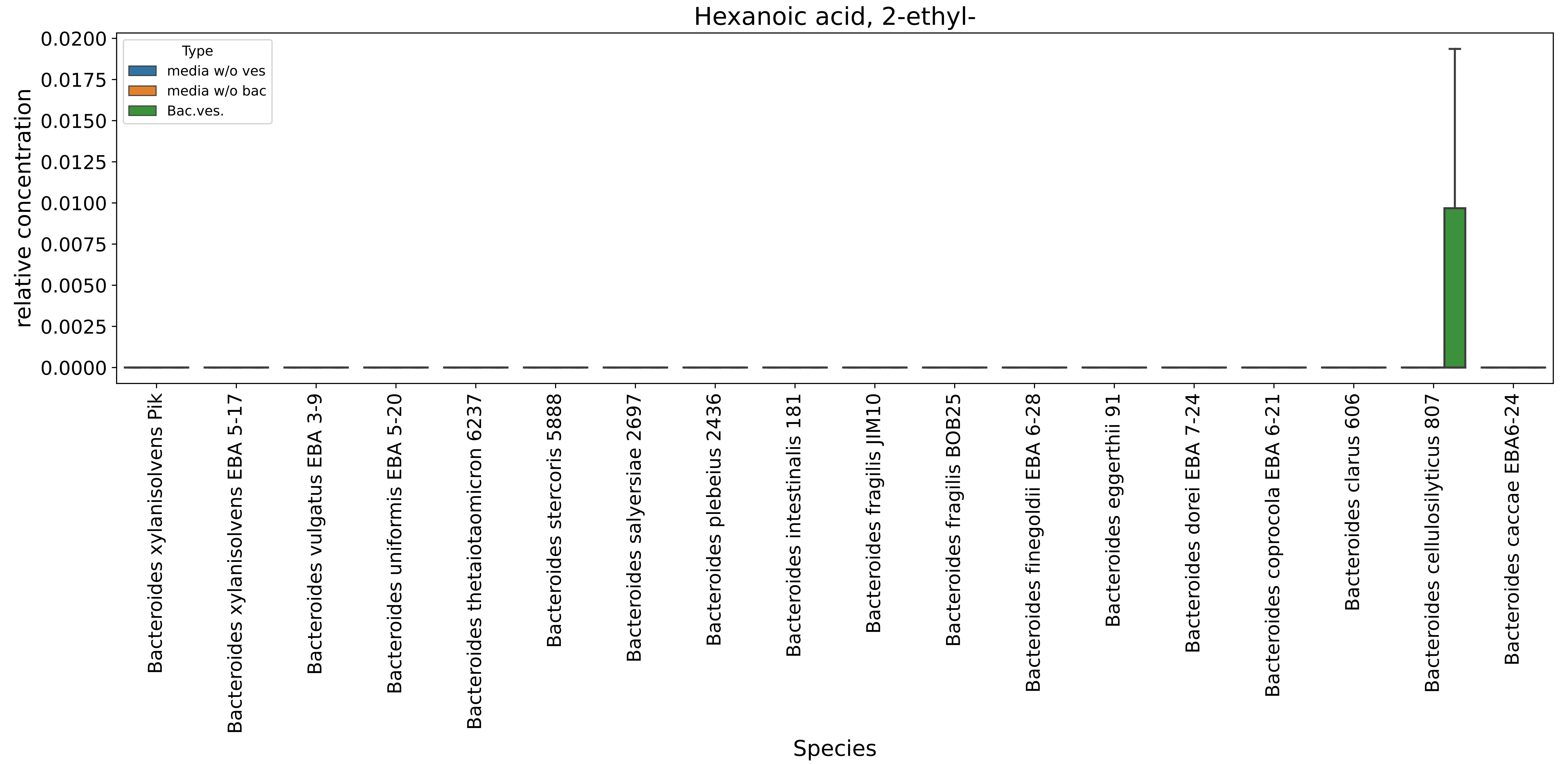

Hexanoic acid, 2-ethyl-
relative concentration
0.0200
0.0175
0.0150
0.0125
0.0100
0.0075
0.0050
0.0025
0.0000
Type
media w/o ves
media w/o bac
Bac.ves.
Bacteroides xylanisolvens Pik
Bacteroides xylanisolvens EBA 5-17
Bacteroides vulgatus EBA 3-9
Bacteroides uniformis EBA 5-20
Bacteroides thetaiotaomicron 6237
Bacteroides stercoris 5888
Bacteroides salyersiae 2697
Bacteroides plebeius 2436
Bacteroides intestinalis 181
Bacteroides fragilis JIM10
Bacteroides fragilis BOB25
Bacteroides finegoldii EBA 6-28
Bacteroides eggerthii 91
Bacteroides dorei EBA 7-24
Bacteroides coprocola EBA 6-21
Bacteroides clarus 606
Bacteroides cellulosilyticus 807
Bacteroides caccae EBA6-24
Species

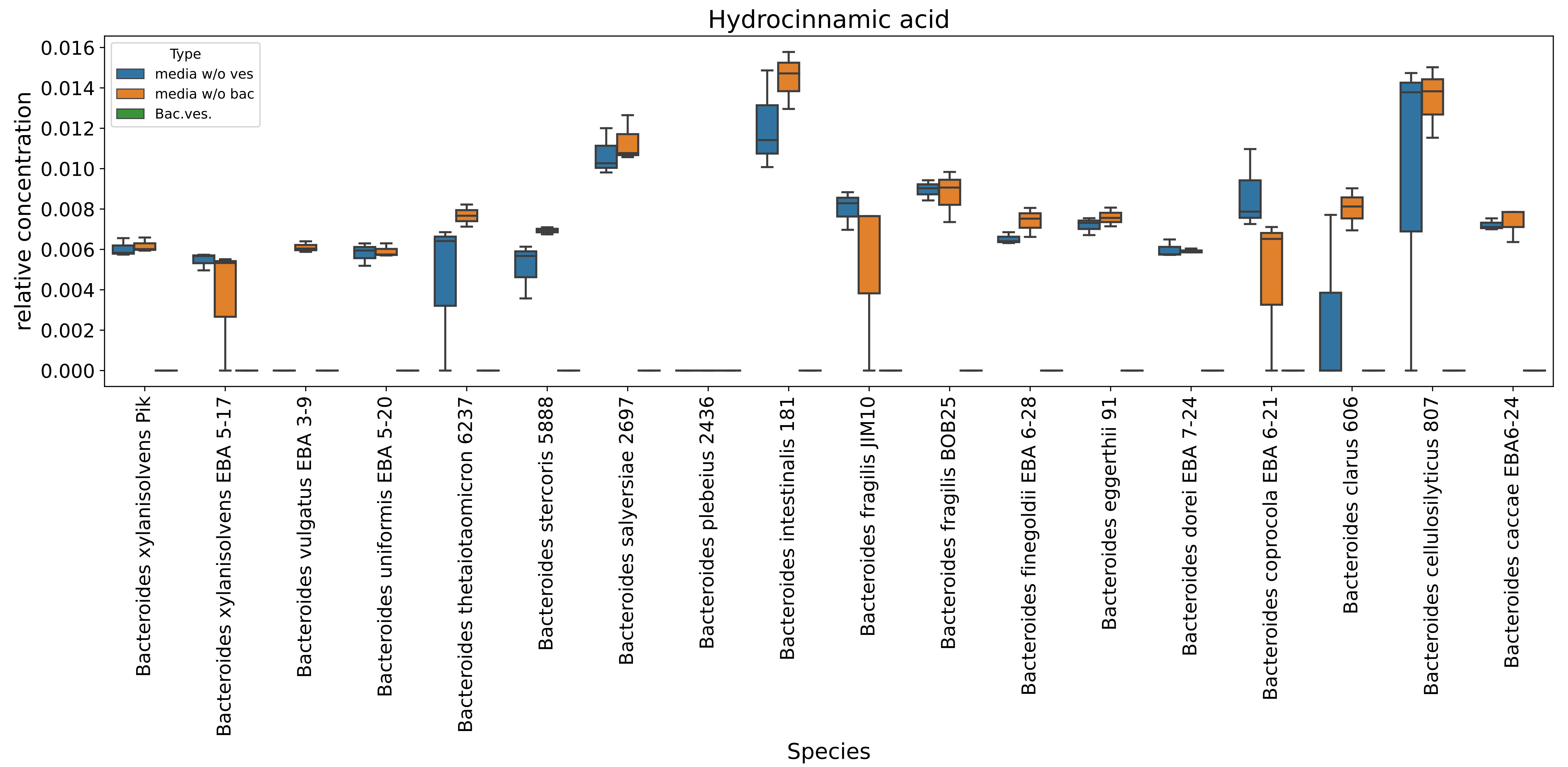
Hydrocinnamic acid
relative concentration
0.016
0.014
0.012
0.010
0.008
0.006
0.004
0.002
0.000
Type
media w/o ves
media w/o bac
Bac.ves.
Bacteroides xylanisolvens Pik
Bacteroides xylanisolvens EBA 5-17
Bacteroides vulgatus EBA 3-9
Bacteroides uniformis EBA 5-20
Bacteroides thetaiotaomicron 6237
Bacteroides stercoris 5888
Bacteroides salyersiae 2697
Bacteroides plebeius 2436
Bacteroides intestinalis 181
Bacteroides fragilis JIM10
Bacteroides fragilis BOB25
Bacteroides finegoldii EBA 6-28
Bacteroides eggerthii 91
Bacteroides dorei EBA 7-24
Bacteroides coprocola EBA 6-21
Bacteroides clarus 606
Bacteroides cellulosilyticus 807
Bacteroides caccae EBA6-24
Species

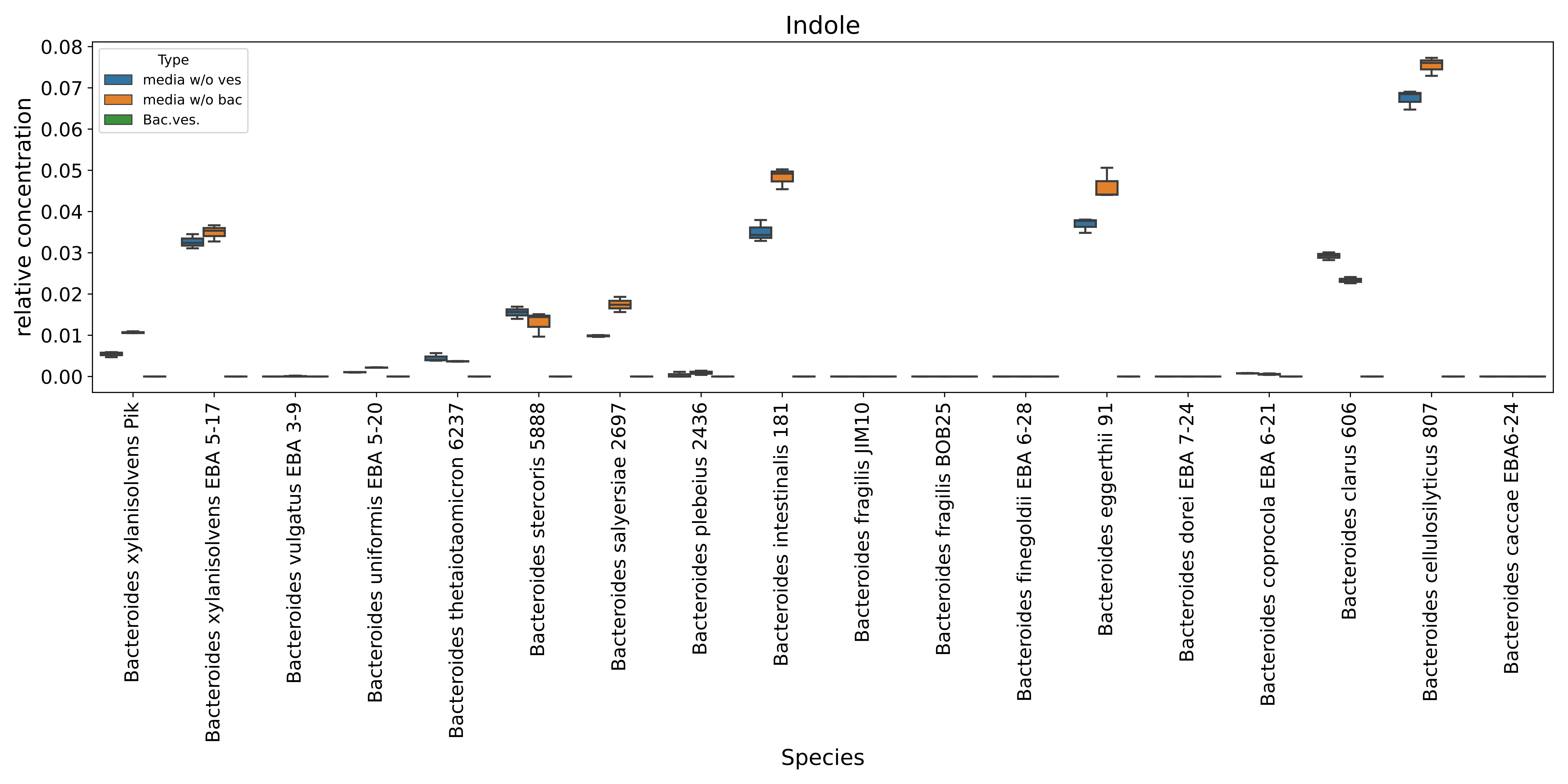

Indole
Type
media w/o ves
media w/o bac
Bac.ves.
relative concentration
0.00
0.01
0.02
0.03
0.04
0.05
0.06
0.07
0.08
Bacteroides xylanisolvens Pik
Bacteroides xylanisolvens EBA 5-17
Bacteroides vulgatus EBA 3-9
Bacteroides uniformis EBA 5-20
Bacteroides thetaiotaomicron 6237
Bacteroides stercoris 5888
Bacteroides salyersiae 2697
Bacteroides plebeius 2436
Bacteroides intestinalis 181
Bacteroides fragilis JIM10
Bacteroides fragilis BOB25
Bacteroides finegoldii EBA 6-28
Bacteroides eggerthii 91
Bacteroides dorei EBA 7-24
Bacteroides coprocola EBA 6-21
Bacteroides clarus 606
Bacteroides cellulosilyticus 807
Bacteroides caccae EBA6-24
Species

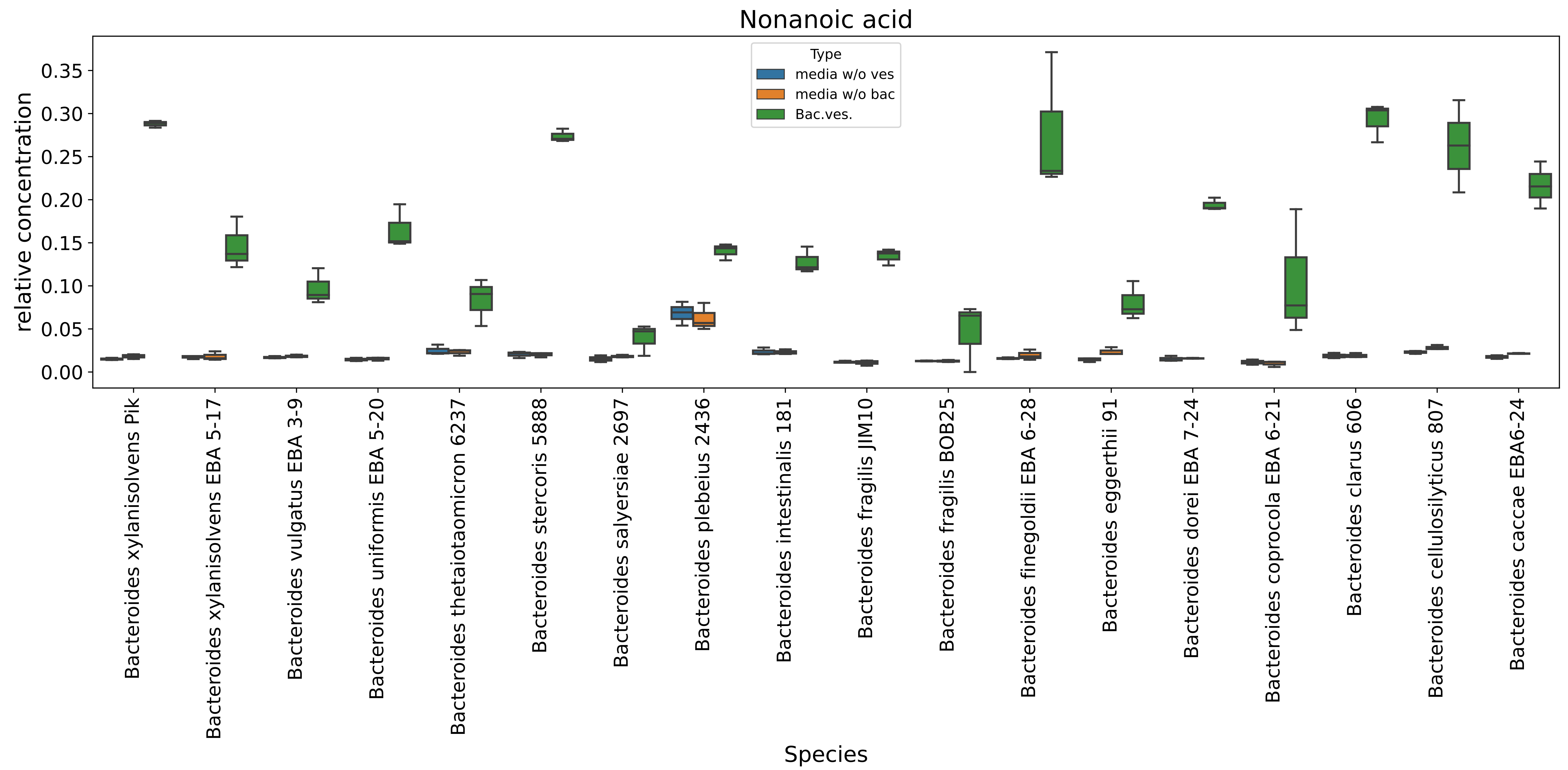

Nonanoic acid
relative concentration
0.00
0.05
0.10
0.15
0.20
0.25
0.30
0.35
Type
media w/o ves
media w/o bac
Bac.ves.
Species
Bacteroides xylanisolvens Pik
Bacteroides xylanisolvens EBA 5-17
Bacteroides vulgatus EBA 3-9
Bacteroides uniformis EBA 5-20
Bacteroides thetaiotaomicron 6237
Bacteroides stercoris 5888
Bacteroides salyersiae 2697
Bacteroides plebeius 2436
Bacteroides intestinalis 181
Bacteroides fragilis JIM10
Bacteroides fragilis BOB25
Bacteroides finegoldii EBA 6-28
Bacteroides eggerthii 91
Bacteroides dorei EBA 7-24
Bacteroides coprocola EBA 6-21
Bacteroides clarus 606
Bacteroides cellulosilyticus 807
Bacteroides caccae EBA6-24

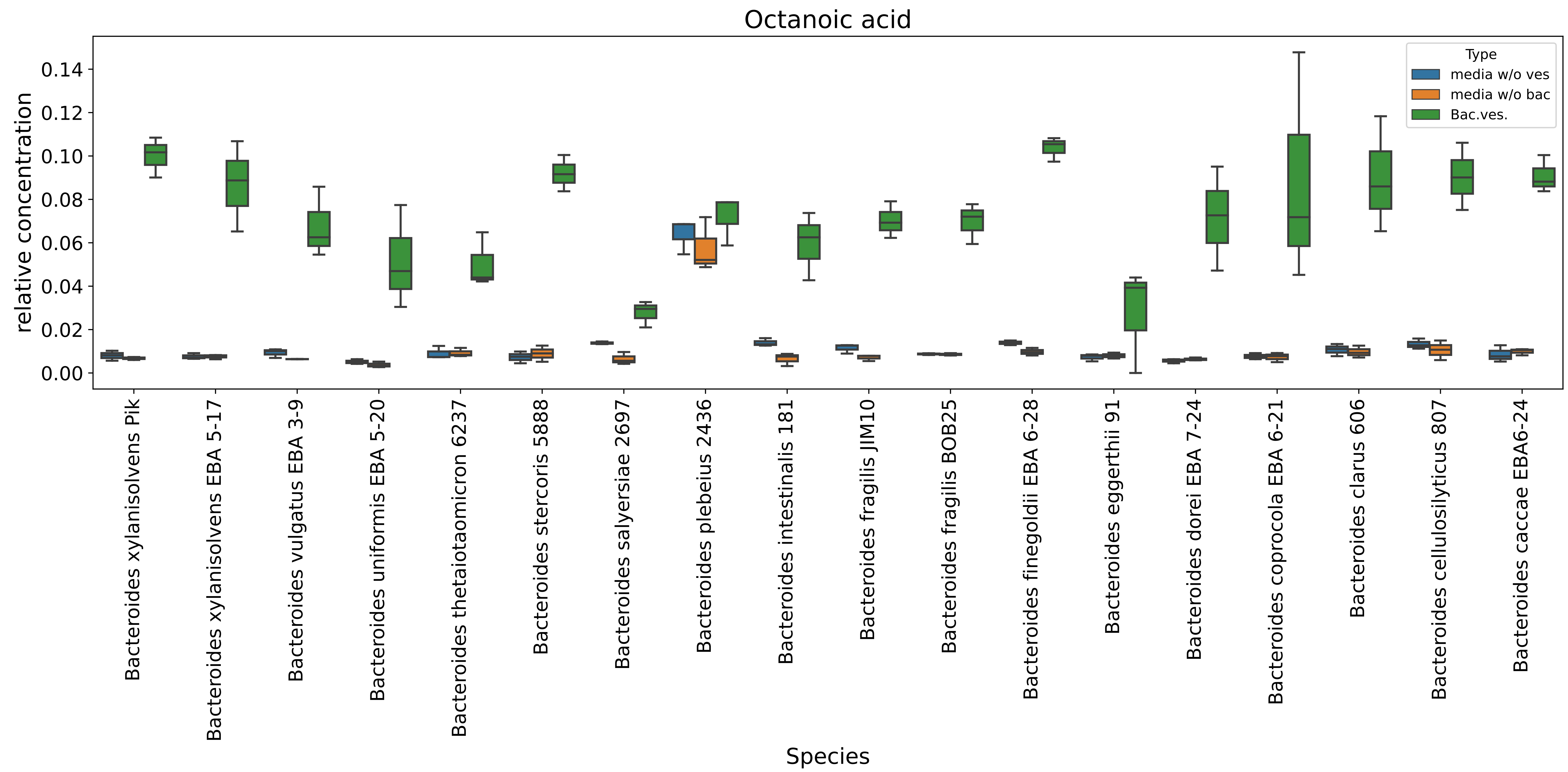

Octanoic acid
relative concentration
0.00
0.02
0.04
0.06
0.08
0.10
0.12
0.14
Type
media w/o ves
media w/o bac
Bac.ves.
Bacteroides xylanisolvens Pik
Bacteroides xylanisolvens EBA 5-17
Bacteroides vulgatus EBA 3-9
Bacteroides uniformis EBA 5-20
Bacteroides thetaiotaomicron 6237
Bacteroides stercoris 5888
Bacteroides salyersiae 2697
Bacteroides plebeius 2436
Bacteroides intestinalis 181
Bacteroides fragilis JIM10
Bacteroides fragilis BOB25
Bacteroides finegoldii EBA 6-28
Bacteroides eggerthii 91
Bacteroides dorei EBA 7-24
Bacteroides coprocola EBA 6-21
Bacteroides clarus 606
Bacteroides cellulosilyticus 807
Bacteroides caccae EBA6-24
Species

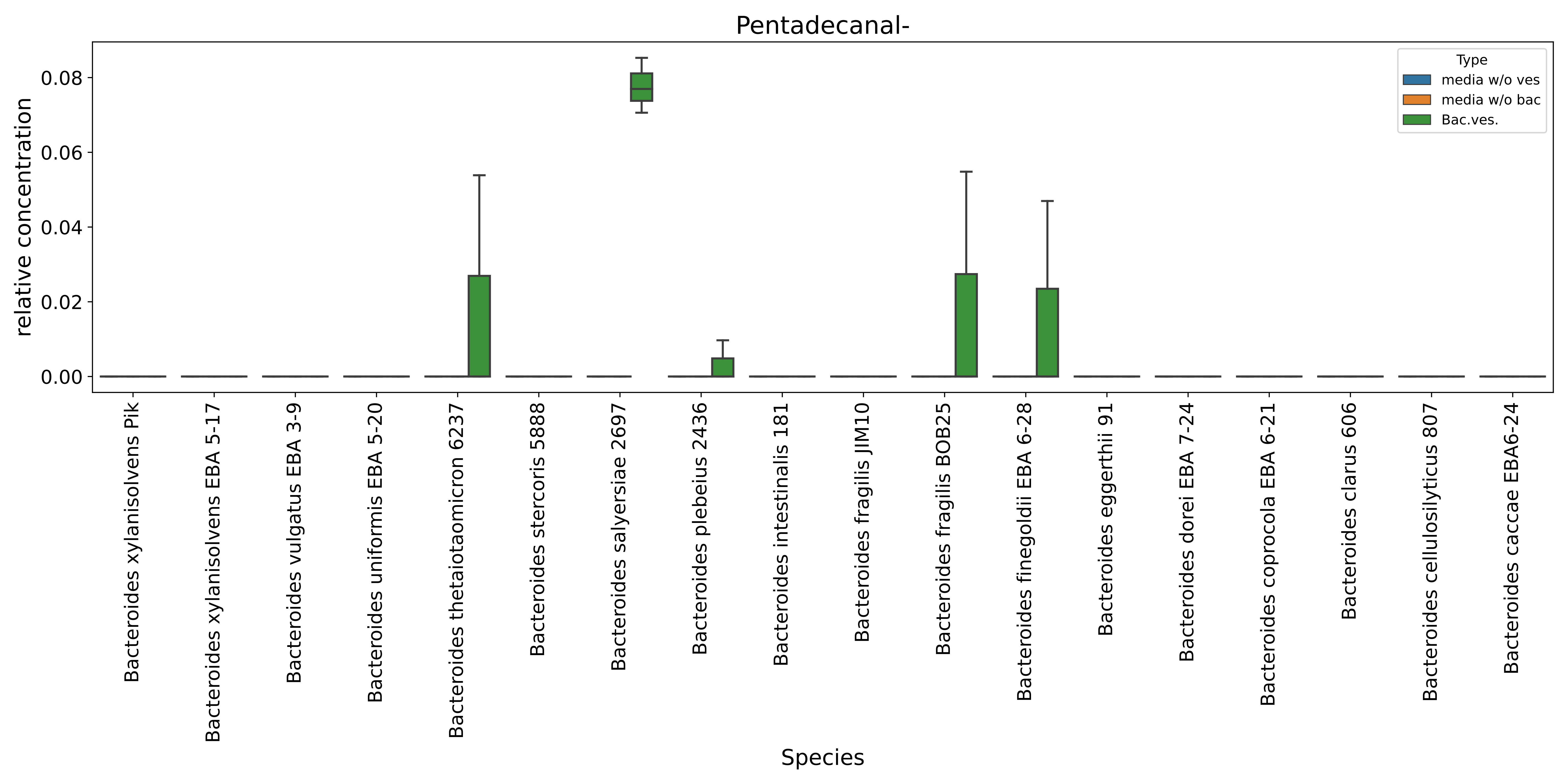

Pentadecanal-
relative concentration
0.08
0.06
0.04
0.02
0.00
Type
media w/o ves
media w/o bac
Bac.ves.
Bacteroides xylanisolvens Pik
Bacteroides xylanisolvens EBA 5-17
Bacteroides vulgatus EBA 3-9
Bacteroides uniformis EBA 5-20
Bacteroides thetaiotaomicron 6237
Bacteroides stercoris 5888
Bacteroides salyersiae 2697
Bacteroides plebeius 2436
Bacteroides intestinalis 181
Bacteroides fragilis JIM10
Bacteroides fragilis BOB25
Bacteroides finegoldii EBA 6-28
Bacteroides eggerthii 91
Bacteroides dorei EBA 7-24
Bacteroides coprocola EBA 6-21
Bacteroides clarus 606
Bacteroides cellulosilyticus 807
Bacteroides caccae EBA6-24
Species

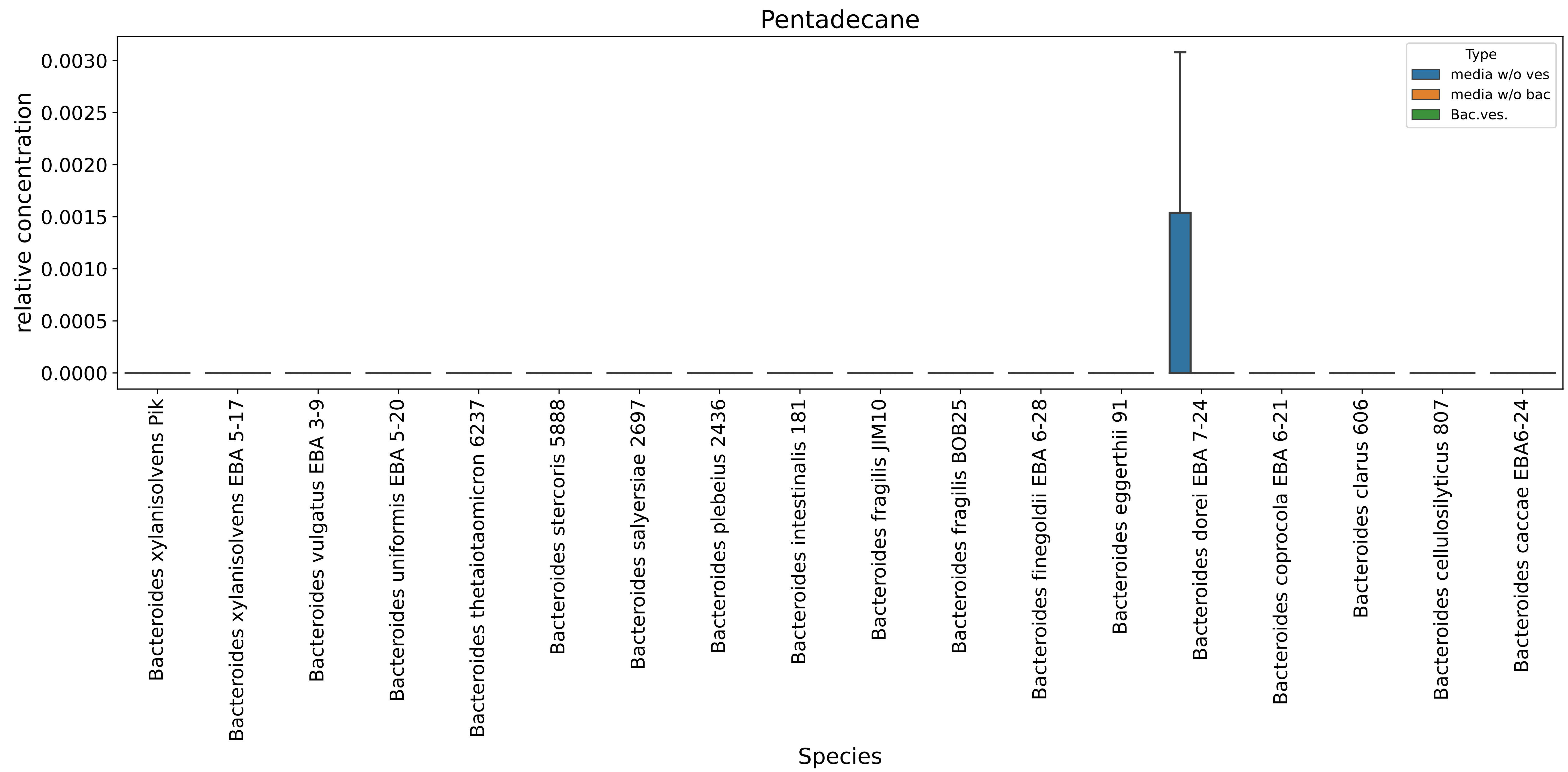
Pentadecane
relative concentration
0.0030
0.0025
0.0020
0.0015
0.0010
0.0005
0.0000
Type
media w/o ves
media w/o bac
Bac.ves.
Bacteroides xylanisolvens Pik
Bacteroides xylanisolvens EBA 5-17
Bacteroides vulgatus EBA 3-9
Bacteroides uniformis EBA 5-20
Bacteroides thetaiotaomicron 6237
Bacteroides stercoris 5888
Bacteroides salyersiae 2697
Bacteroides plebeius 2436
Bacteroides intestinalis 181
Bacteroides fragilis JIM10
Bacteroides fragilis BOB25
Bacteroides finegoldii EBA 6-28
Bacteroides eggerthii 91
Bacteroides dorei EBA 7-24
Bacteroides coprocola EBA 6-21
Bacteroides clarus 606
Bacteroides cellulosilyticus 807
Bacteroides caccae EBA6-24
Species

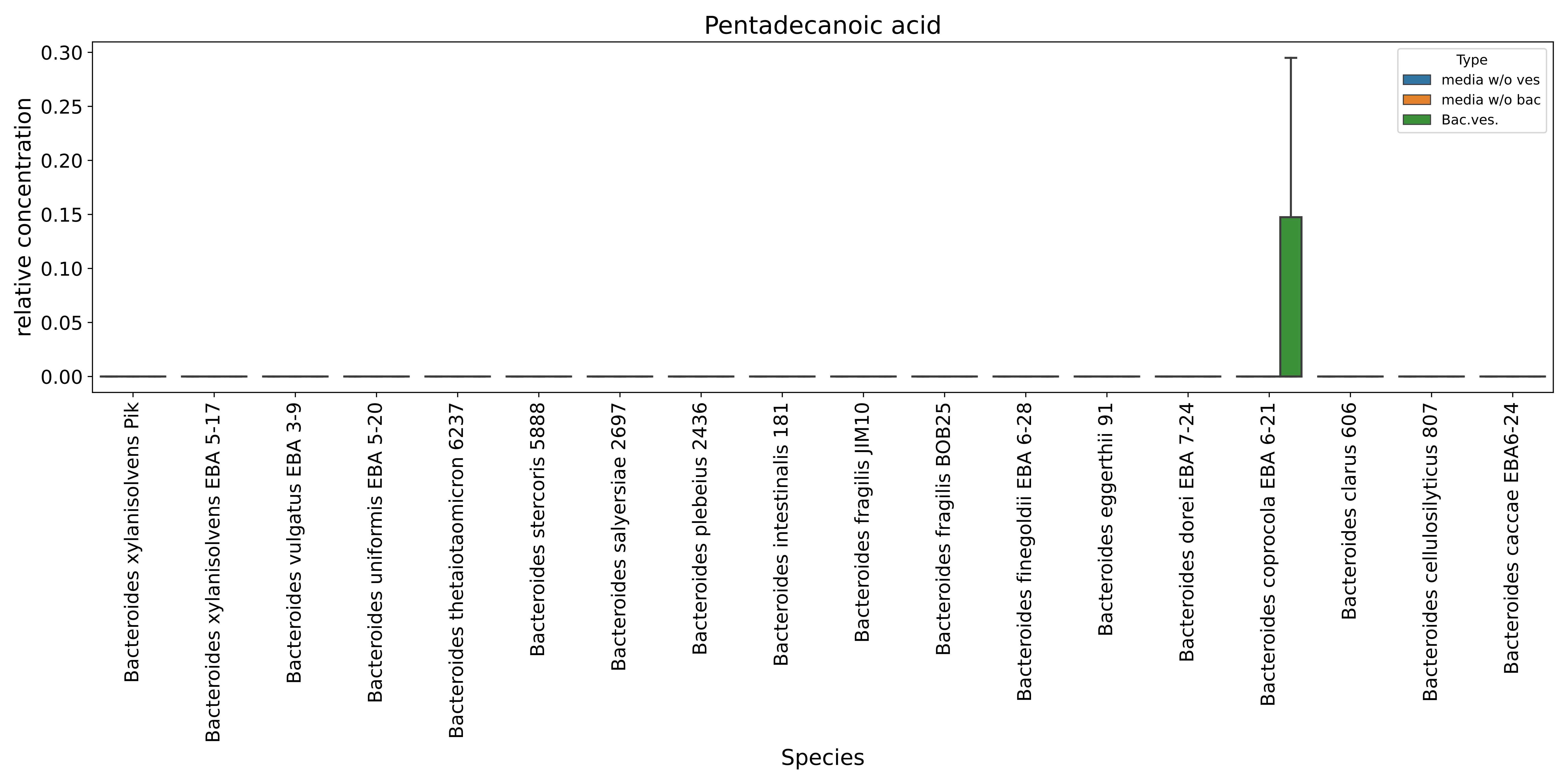
Pentadecanoic acid
relative concentration
0.30
0.25
0.20
0.15
0.10
0.05
0.00
Type
media w/o ves
media w/o bac
Bac.ves.
Bacteroides xylanisolvens Pik
Bacteroides xylanisolvens EBA 5-17
Bacteroides vulgatus EBA 3-9
Bacteroides uniformis EBA 5-20
Bacteroides thetaiotaomicron 6237
Bacteroides stercoris 5888
Bacteroides salyersiae 2697
Bacteroides plebeius 2436
Bacteroides intestinalis 181
Bacteroides fragilis JIM10
Bacteroides fragilis BOB25
Bacteroides finegoldii EBA 6-28
Bacteroides eggerthii 91
Bacteroides dorei EBA 7-24
Bacteroides coprocola EBA 6-21
Bacteroides clarus 606
Bacteroides cellulosilyticus 807
Bacteroides caccae EBA6-24
Species

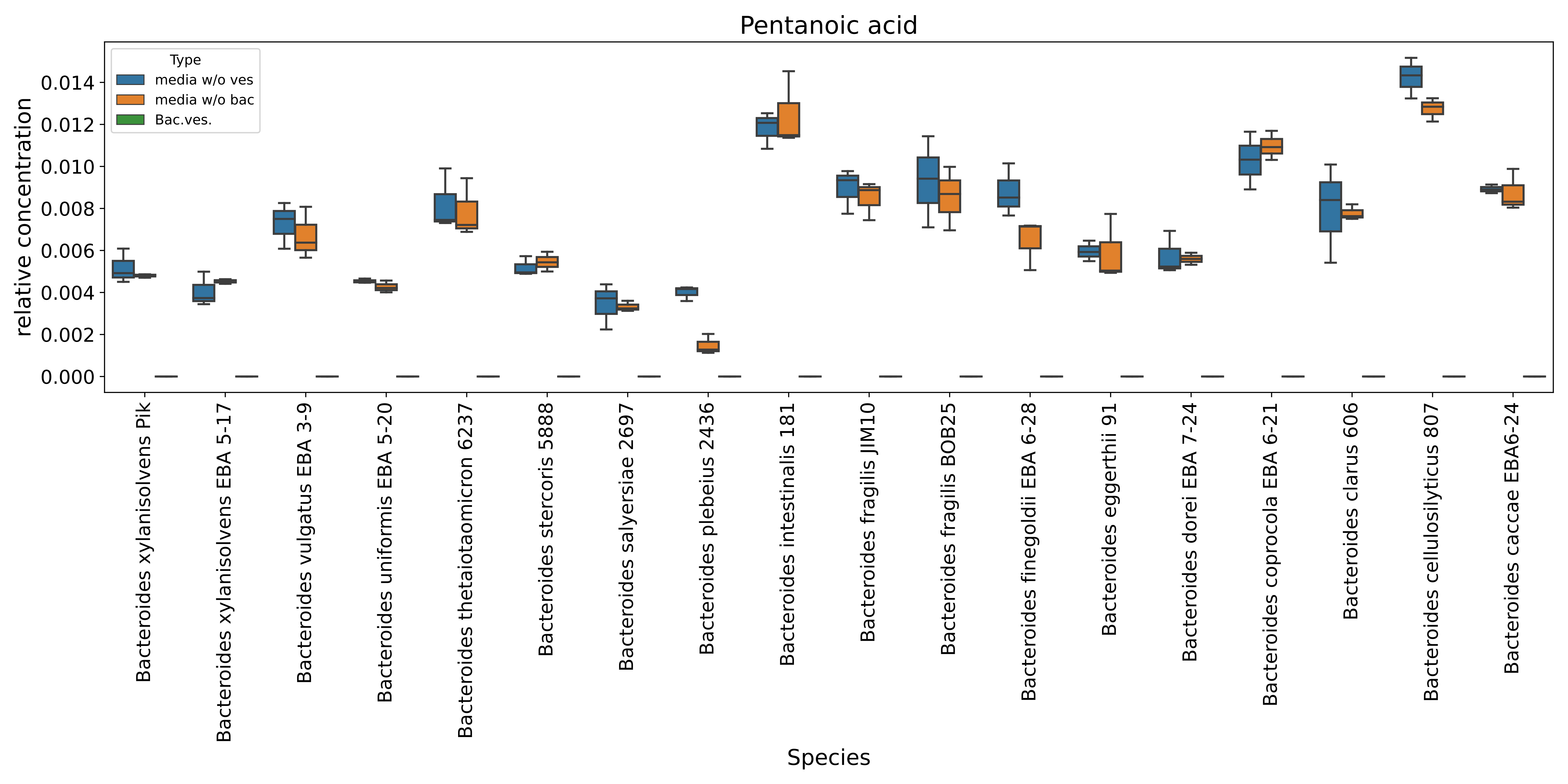

Pentanoic acid
Type
media w/o ves
media w/o bac
Bac.ves.
relative concentration
0.014
0.012
0.010
0.008
0.006
0.004
0.002
0.000
Bacteroides xylanisolvens Pik
Bacteroides xylanisolvens EBA 5-17
Bacteroides vulgatus EBA 3-9
Bacteroides uniformis EBA 5-20
Bacteroides thetaiotaomicron 6237
Bacteroides stercoris 5888
Bacteroides salyersiae 2697
Bacteroides plebeius 2436
Bacteroides intestinalis 181
Bacteroides fragilis JIM10
Bacteroides fragilis BOB25
Bacteroides finegoldii EBA 6-28
Bacteroides eggerthii 91
Bacteroides dorei EBA 7-24
Bacteroides coprocola EBA 6-21
Bacteroides clarus 606
Bacteroides cellulosilyticus 807
Bacteroides caccae EBA6-24
Species

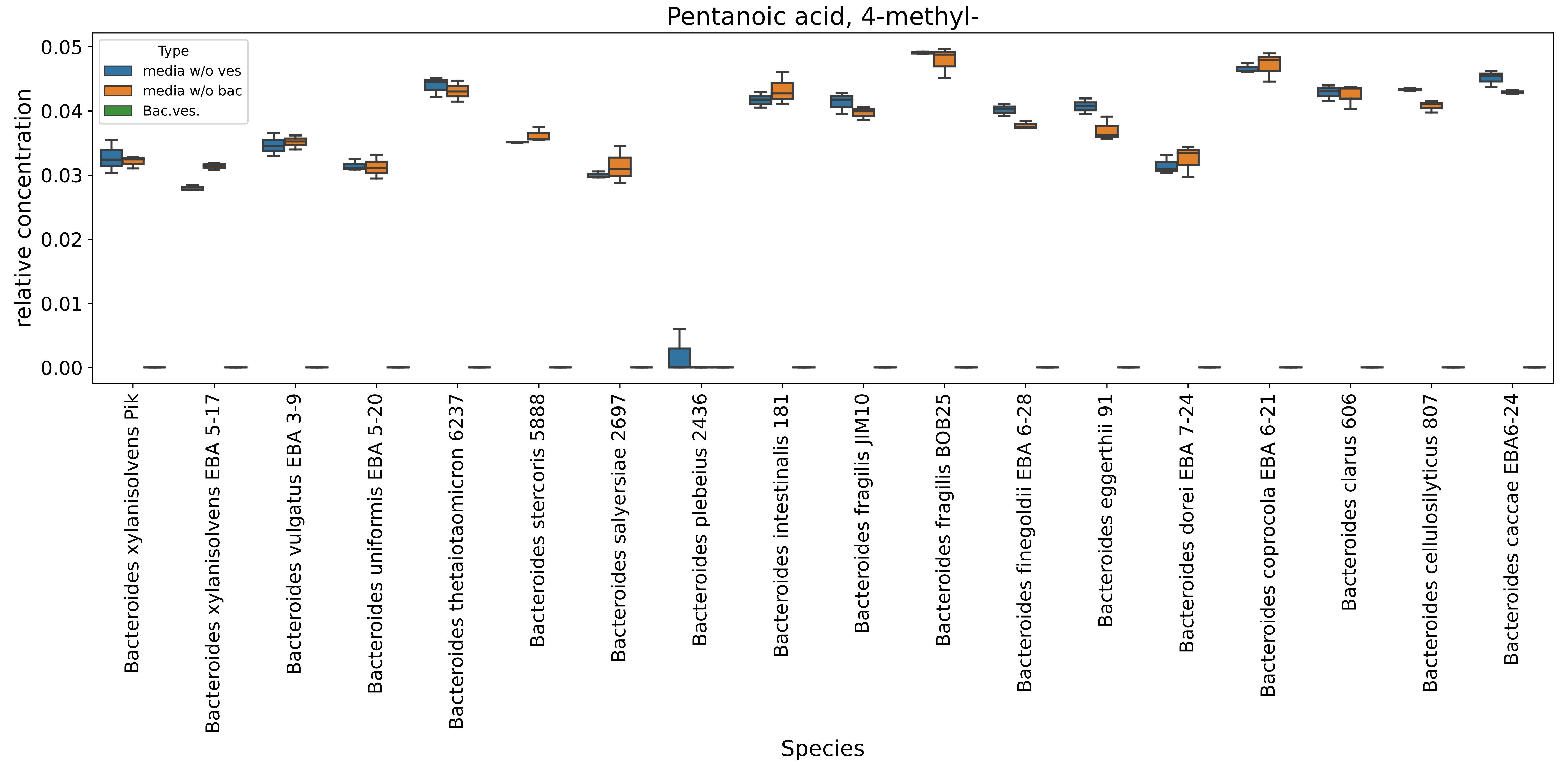

Pentanoic acid, 4-methyl-
Type
media w/o ves
media w/o bac
Bac.ves.
relative concentration
0.05
0.04
0.03
0.02
0.01
0.00
Bacteroides xylanisolvens Pik
Bacteroides xylanisolvens EBA 5-17
Bacteroides vulgatus EBA 3-9
Bacteroides uniformis EBA 5-20
Bacteroides thetaiotaomicron 6237
Bacteroides stercoris 5888
Bacteroides salyersiae 2697
Bacteroides plebeius 2436
Bacteroides intestinalis 181
Bacteroides fragilis JIM10
Bacteroides fragilis BOB25
Bacteroides finegoldii EBA 6-28
Bacteroides eggerthii 91
Bacteroides dorei EBA 7-24
Bacteroides coprocola EBA 6-21
Bacteroides clarus 606
Bacteroides cellulosilyticus 807
Bacteroides caccae EBA6-24
Species

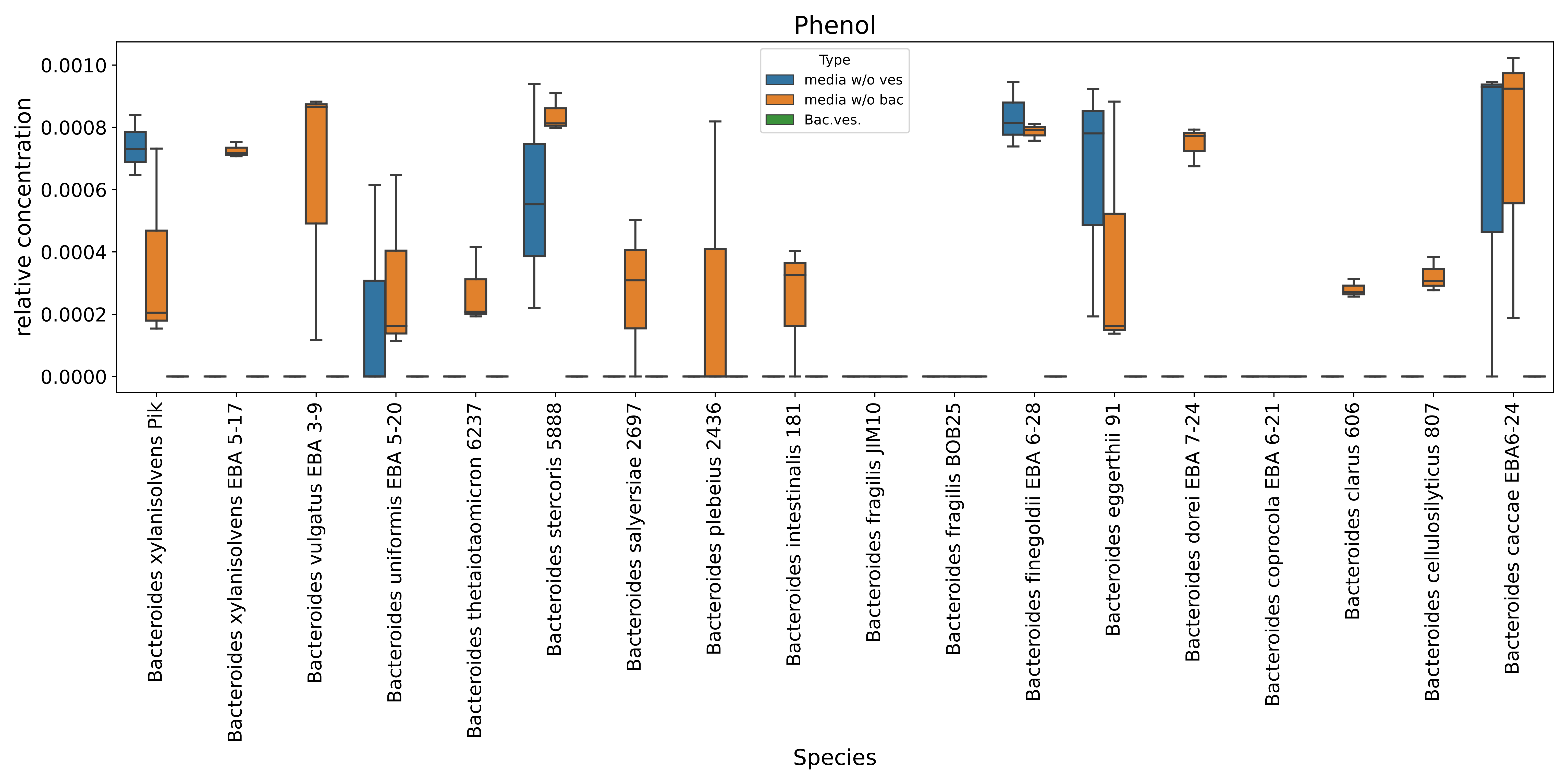

Phenol
Type
media w/o ves
media w/o bac
Bac.ves.
relative concentration
0.0010
0.0008
0.0006
0.0004
0.0002
0.0000
Bacteroides xylanisolvens Pik
Bacteroides xylanisolvens EBA 5-17
Bacteroides vulgatus EBA 3-9
Bacteroides uniformis EBA 5-20
Bacteroides thetaiotaomicron 6237
Bacteroides stercoris 5888
Bacteroides salyersiae 2697
Bacteroides plebeius 2436
Bacteroides intestinalis 181
Bacteroides fragilis JIM10
Bacteroides fragilis BOB25
Bacteroides finegoldii EBA 6-28
Bacteroides eggerthii 91
Bacteroides dorei EBA 7-24
Bacteroides coprocola EBA 6-21
Bacteroides clarus 606
Bacteroides cellulosilyticus 807
Bacteroides caccae EBA6-24
Species

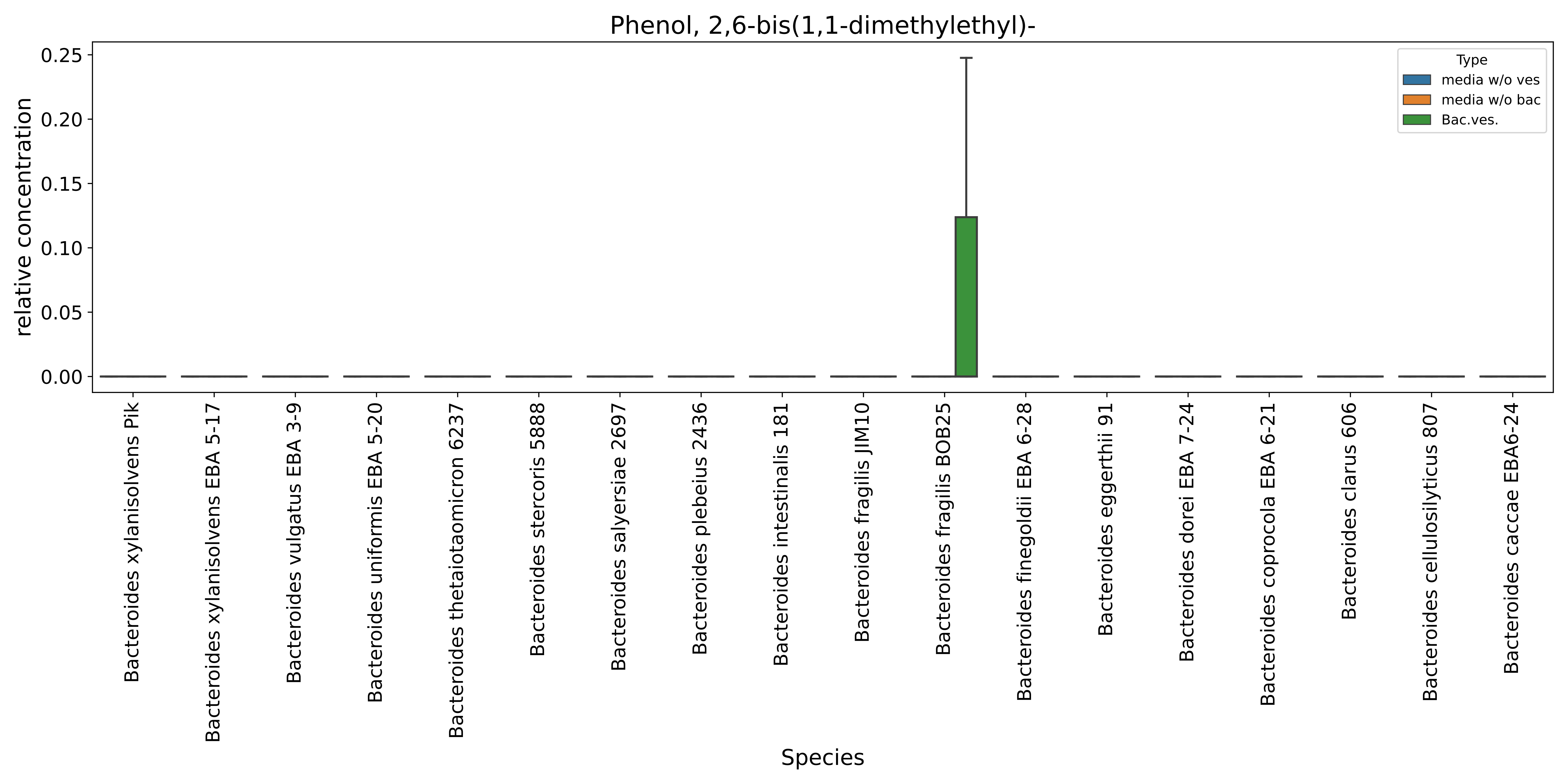
Phenol, 2,6-bis(1,1-dimethylethyl)-
relative concentration
0.25
0.20
0.15
0.10
0.05
0.00
Type
media w/o ves
media w/o bac
Bac.ves.
Bacteroides xylanisolvens Pik
Bacteroides xylanisolvens EBA 5-17
Bacteroides vulgatus EBA 3-9
Bacteroides uniformis EBA 5-20
Bacteroides thetaiotaomicron 6237
Bacteroides stercoris 5888
Bacteroides salyersiae 2697
Bacteroides plebeius 2436
Bacteroides intestinalis 181
Bacteroides fragilis JIM10
Bacteroides fragilis BOB25
Bacteroides finegoldii EBA 6-28
Bacteroides eggerthii 91
Bacteroides dorei EBA 7-24
Bacteroides coprocola EBA 6-21
Bacteroides clarus 606
Bacteroides cellulosilyticus 807
Bacteroides caccae EBA6-24
Species

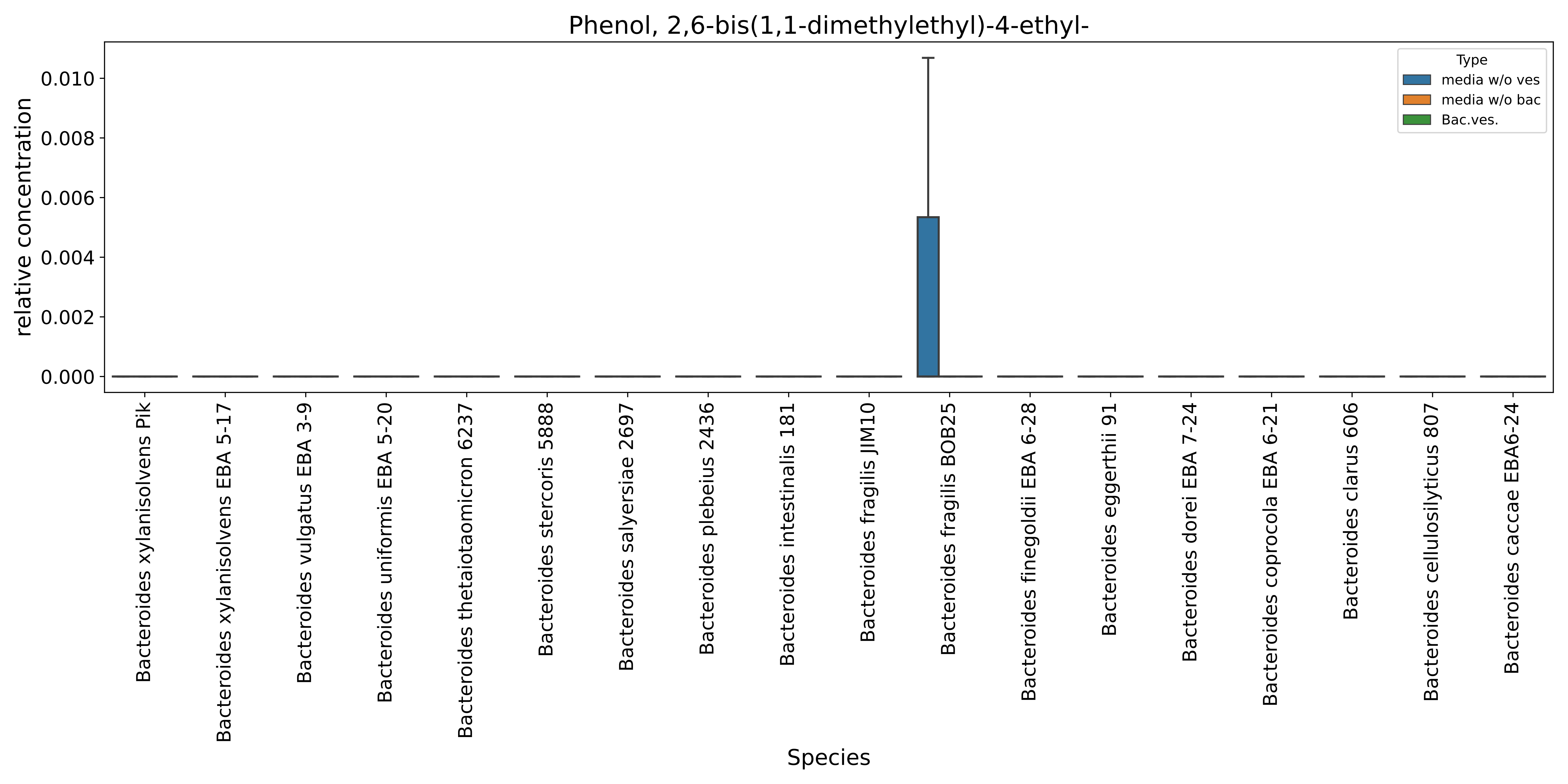

Phenol, 2,6-bis(1,1-dimethylethyl)-4-ethyl-
relative concentration
0.010
0.008
0.006
0.004
0.002
0.000
Type
media w/o ves
media w/o bac
Bac.ves.
Bacteroides xylanisolvens Pik
Bacteroides xylanisolvens EBA 5-17
Bacteroides vulgatus EBA 3-9
Bacteroides uniformis EBA 5-20
Bacteroides thetaiotaomicron 6237
Bacteroides stercoris 5888
Bacteroides salyersiae 2697
Bacteroides plebeius 2436
Bacteroides intestinalis 181
Bacteroides fragilis JIM10
Bacteroides fragilis BOB25
Bacteroides finegoldii EBA 6-28
Bacteroides eggerthii 91
Bacteroides dorei EBA 7-24
Bacteroides coprocola EBA 6-21
Bacteroides clarus 606
Bacteroides cellulosilyticus 807
Bacteroides caccae EBA6-24
Species

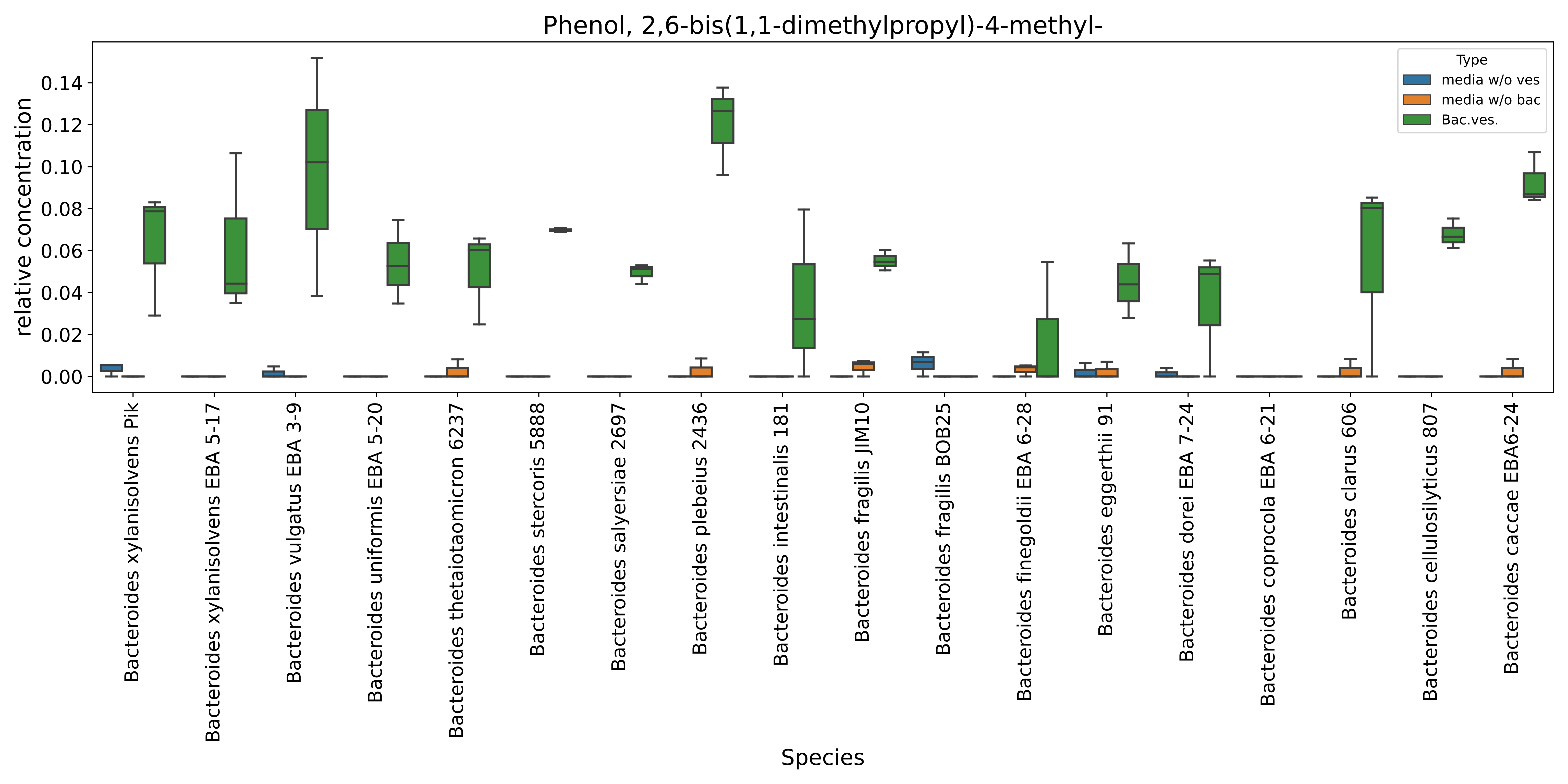
Phenol, 2,6-bis(1,1-dimethylpropyl)-4-methyl-
relative concentration
0.00
0.02
0.04
0.06
0.08
0.10
0.12
0.14
Type
media w/o ves
media w/o bac
Bac.ves.
Bacteroides xylanisolvens Pik
Bacteroides xylanisolvens EBA 5-17
Bacteroides vulgatus EBA 3-9
Bacteroides uniformis EBA 5-20
Bacteroides thetaiotaomicron 6237
Bacteroides stercoris 5888
Bacteroides salyersiae 2697
Bacteroides plebeius 2436
Bacteroides intestinalis 181
Bacteroides fragilis JIM10
Bacteroides fragilis BOB25
Bacteroides finegoldii EBA 6-28
Bacteroides eggerthii 91
Bacteroides dorei EBA 7-24
Bacteroides coprocola EBA 6-21
Bacteroides clarus 606
Bacteroides cellulosilyticus 807
Bacteroides caccae EBA6-24
Species

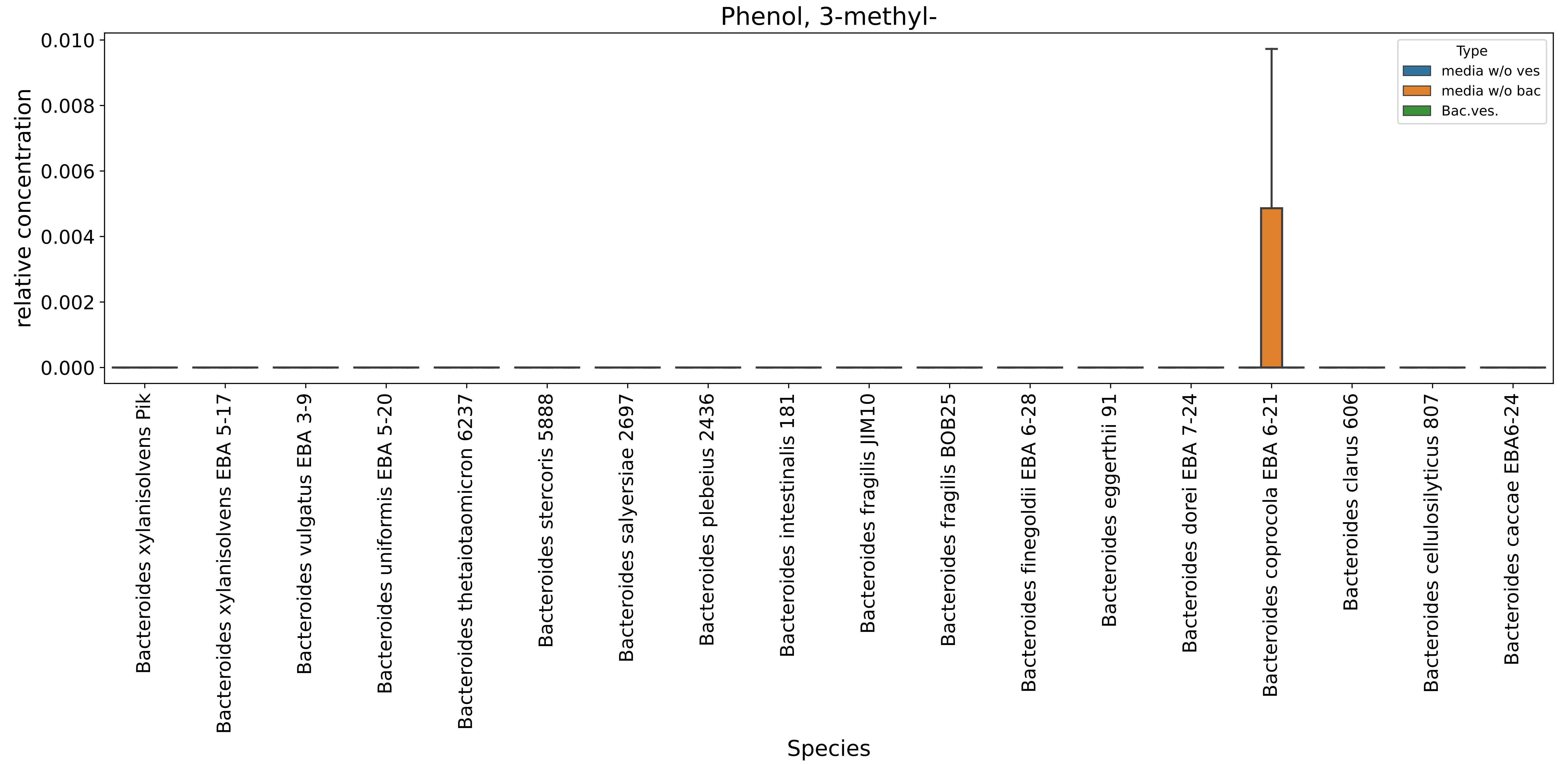

Phenol, 3-methyl-
relative concentration
0.010
0.008
0.006
0.004
0.002
0.000
Type
media w/o ves
media w/o bac
Bac.ves.
Bacteroides xylanisolvens Pik
Bacteroides xylanisolvens EBA 5-17
Bacteroides vulgatus EBA 3-9
Bacteroides uniformis EBA 5-20
Bacteroides thetaiotaomicron 6237
Bacteroides stercoris 5888
Bacteroides salyersiae 2697
Bacteroides plebeius 2436
Bacteroides intestinalis 181
Bacteroides fragilis JIM10
Bacteroides fragilis BOB25
Bacteroides finegoldii EBA 6-28
Bacteroides eggerthii 91
Bacteroides dorei EBA 7-24
Bacteroides coprocola EBA 6-21
Bacteroides clarus 606
Bacteroides cellulosilyticus 807
Bacteroides caccae EBA6-24
Species

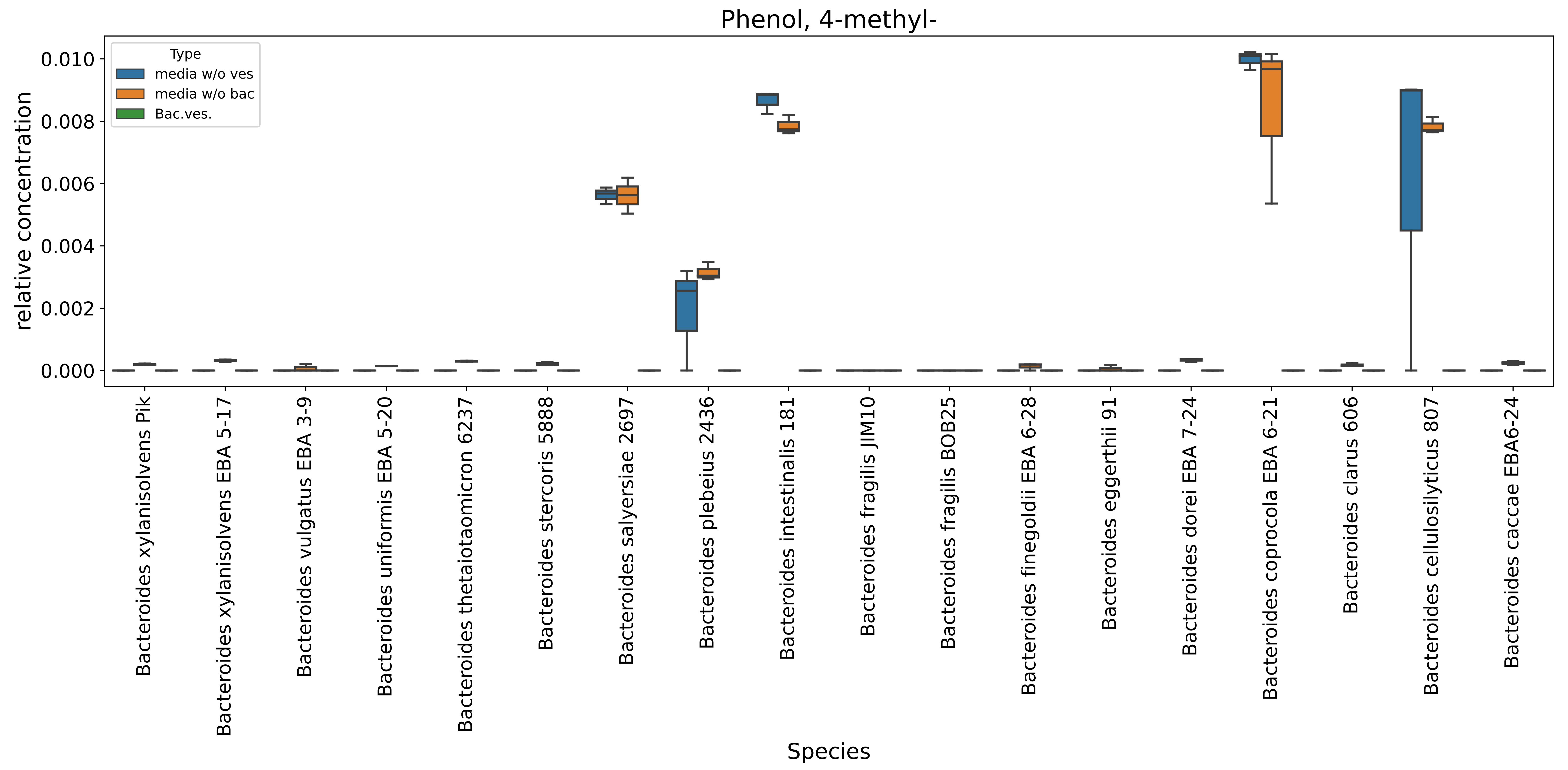

Phenol, 4-methyl-
Type
media w/o ves
media w/o bac
Bac.ves.
relative concentration
0.010
0.008
0.006
0.004
0.002
0.000
Bacteroides xylanisolvens Pik
Bacteroides xylanisolvens EBA 5-17
Bacteroides vulgatus EBA 3-9
Bacteroides uniformis EBA 5-20
Bacteroides thetaiotaomicron 6237
Bacteroides stercoris 5888
Bacteroides salyersiae 2697
Bacteroides plebeius 2436
Bacteroides intestinalis 181
Bacteroides fragilis JIM10
Bacteroides fragilis BOB25
Bacteroides finegoldii EBA 6-28
Bacteroides eggerthii 91
Bacteroides dorei EBA 7-24
Bacteroides coprocola EBA 6-21
Bacteroides clarus 606
Bacteroides cellulosilyticus 807
Bacteroides caccae EBA6-24
Species

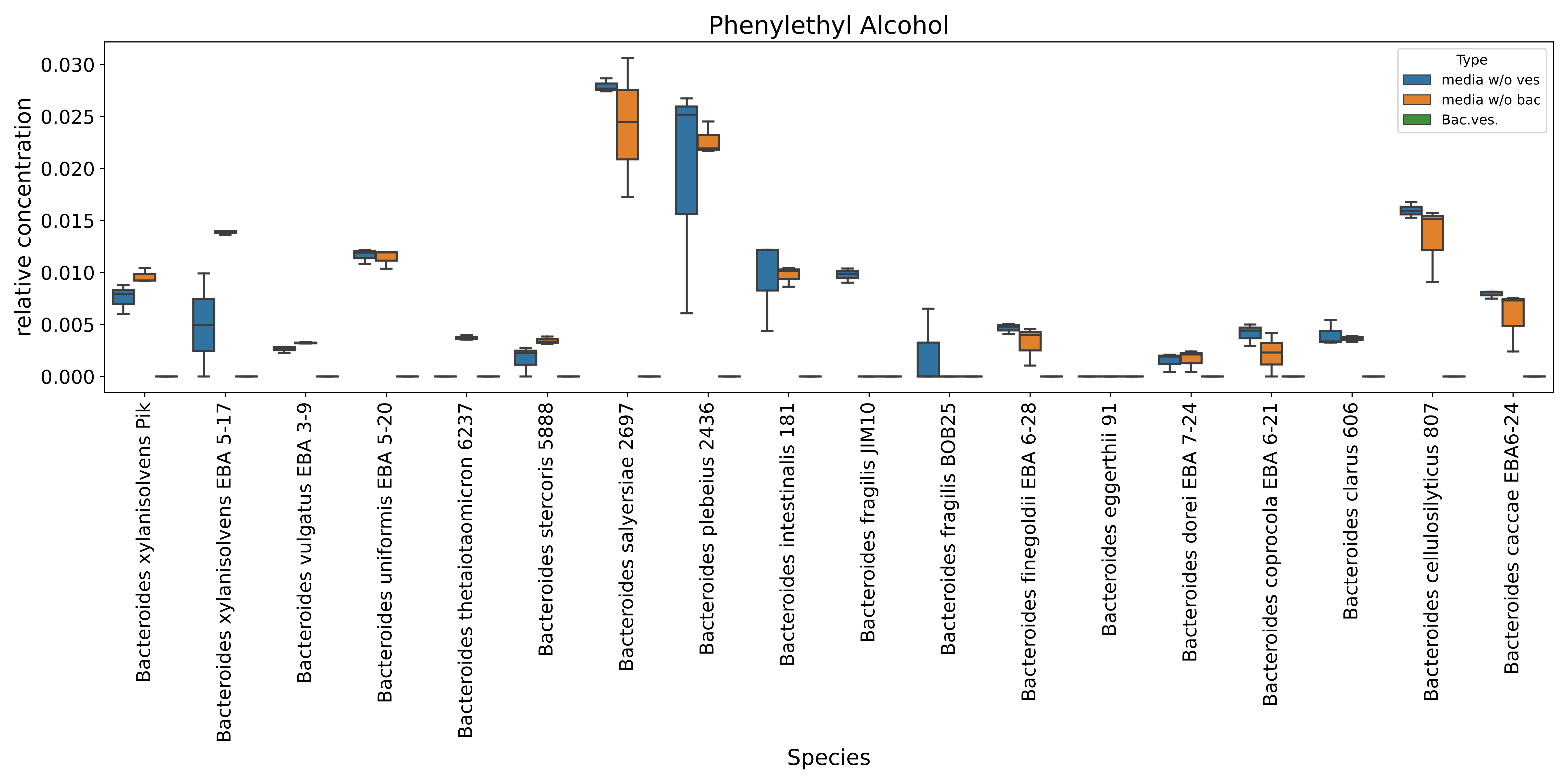
Phenylethyl Alcohol
relative concentration
0.030
0.025
0.020
0.015
0.010
0.005
0.000
Type
media w/o ves
media w/o bac
Bac.ves.
Bacteroides xylanisolvens Pik
Bacteroides xylanisolvens EBA 5-17
Bacteroides vulgatus EBA 3-9
Bacteroides uniformis EBA 5-20
Bacteroides thetaiotaomicron 6237
Bacteroides stercoris 5888
Bacteroides salyersiae 2697
Bacteroides plebeius 2436
Bacteroides intestinalis 181
Bacteroides fragilis JIM10
Bacteroides fragilis BOB25
Bacteroides finegoldii EBA 6-28
Bacteroides eggerthii 91
Bacteroides dorei EBA 7-24
Bacteroides coprocola EBA 6-21
Bacteroides clarus 606
Bacteroides cellulosilyticus 807
Bacteroides caccae EBA6-24
Species

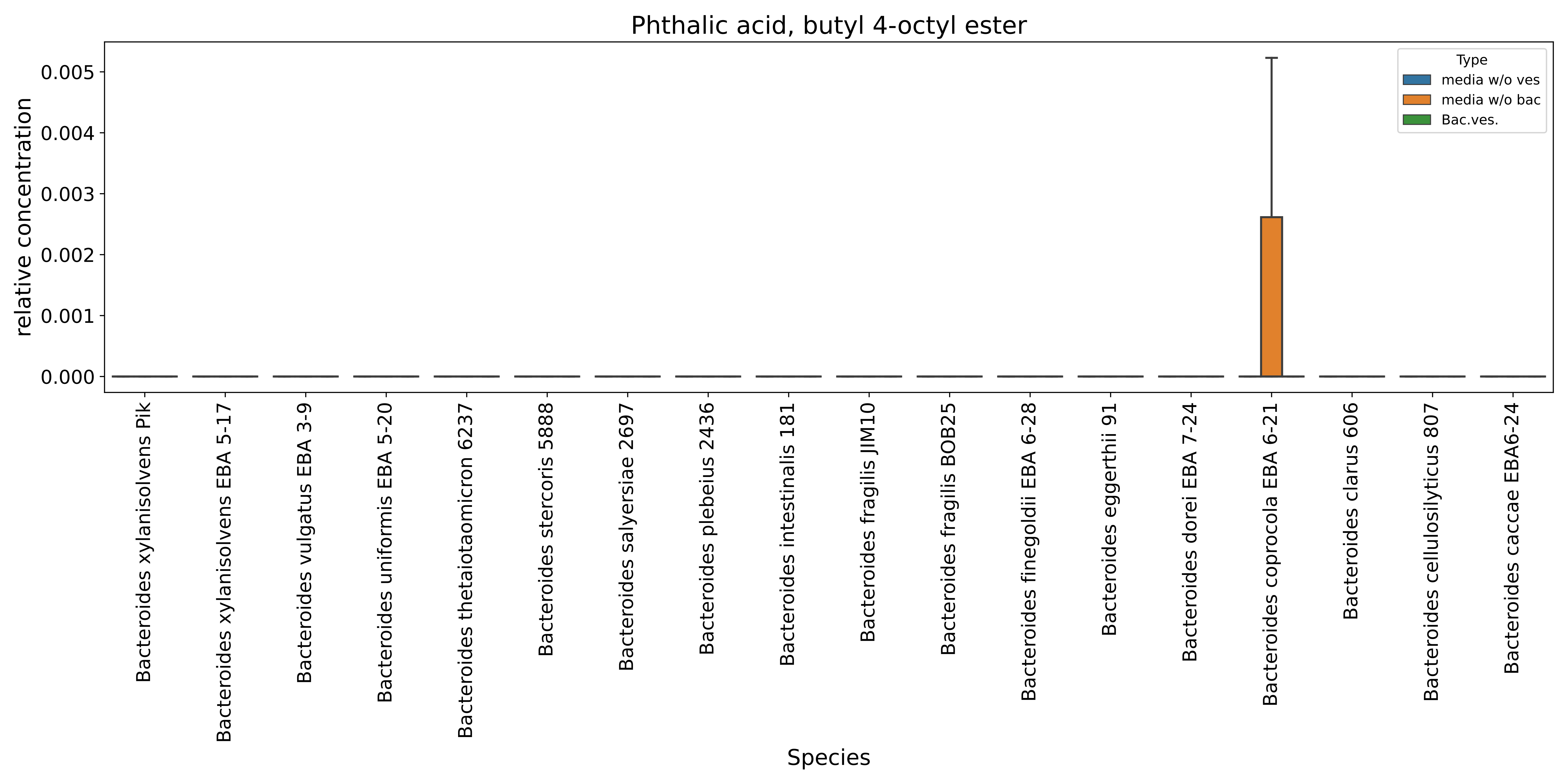

Phthalic acid, butyl 4-octyl ester
relative concentration
0.005
0.004
0.003
0.002
0.001
0.000
Type
media w/o ves
media w/o bac
Bac.ves.
Bacteroides xylanisolvens Pik
Bacteroides xylanisolvens EBA 5-17
Bacteroides vulgatus EBA 3-9
Bacteroides uniformis EBA 5-20
Bacteroides thetaiotaomicron 6237
Bacteroides stercoris 5888
Bacteroides salyersiae 2697
Bacteroides plebeius 2436
Bacteroides intestinalis 181
Bacteroides fragilis JIM10
Bacteroides fragilis BOB25
Bacteroides finegoldii EBA 6-28
Bacteroides eggerthii 91
Bacteroides dorei EBA 7-24
Bacteroides coprocola EBA 6-21
Bacteroides clarus 606
Bacteroides cellulosilyticus 807
Bacteroides caccae EBA6-24
Species

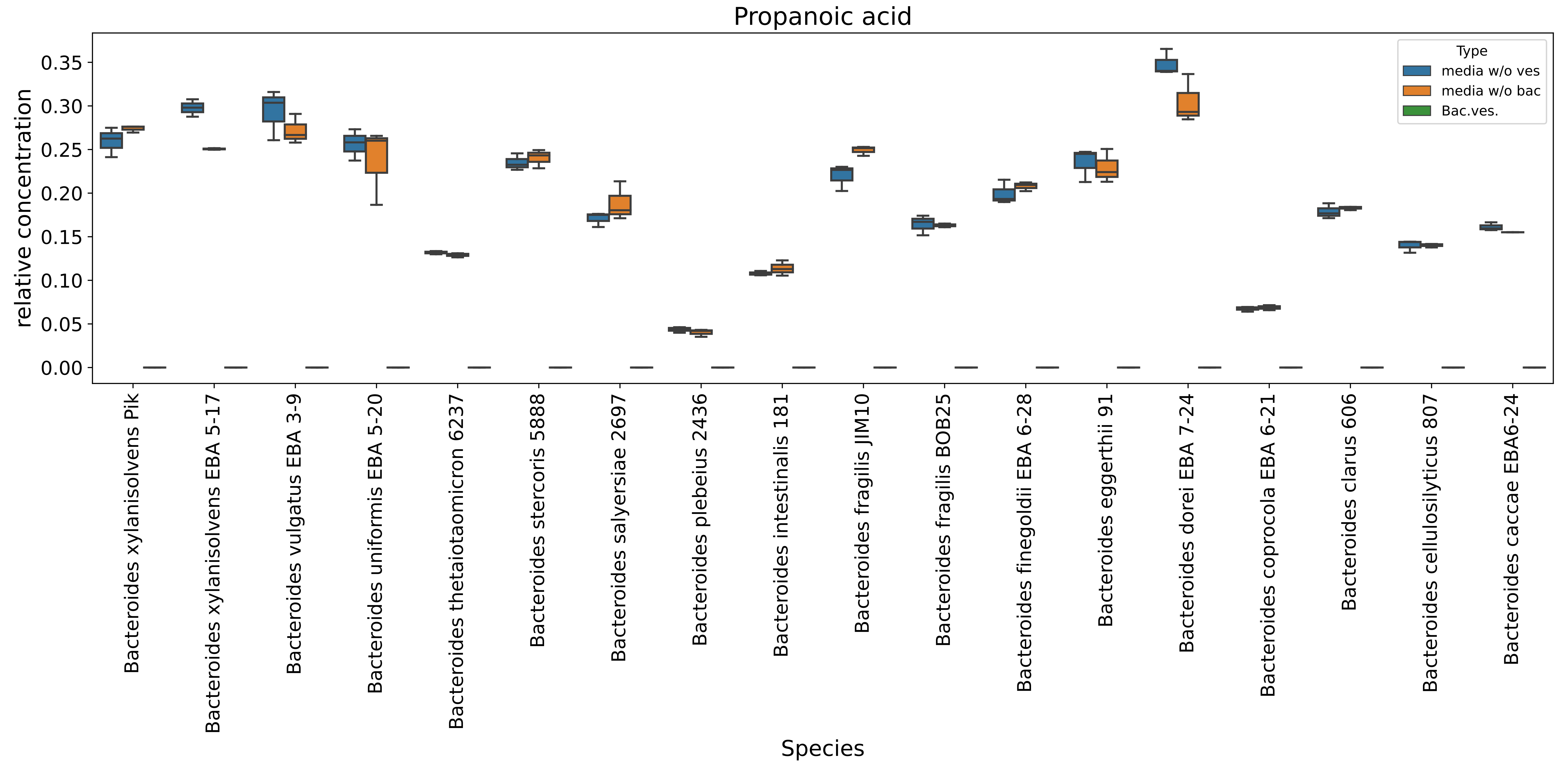

Propanoic acid
relative concentration
0.35
0.30
0.25
0.20
0.15
0.10
0.05
0.00
Type
media w/o ves
media w/o bac
Bac.ves.
Bacteroides xylanisolvens Pik
Bacteroides xylanisolvens EBA 5-17
Bacteroides vulgatus EBA 3-9
Bacteroides uniformis EBA 5-20
Bacteroides thetaiotaomicron 6237
Bacteroides stercoris 5888
Bacteroides salyersiae 2697
Bacteroides plebeius 2436
Bacteroides intestinalis 181
Bacteroides fragilis JIM10
Bacteroides fragilis BOB25
Bacteroides finegoldii EBA 6-28
Bacteroides eggerthii 91
Bacteroides dorei EBA 7-24
Bacteroides coprocola EBA 6-21
Bacteroides clarus 606
Bacteroides cellulosilyticus 807
Bacteroides caccae EBA6-24
Species

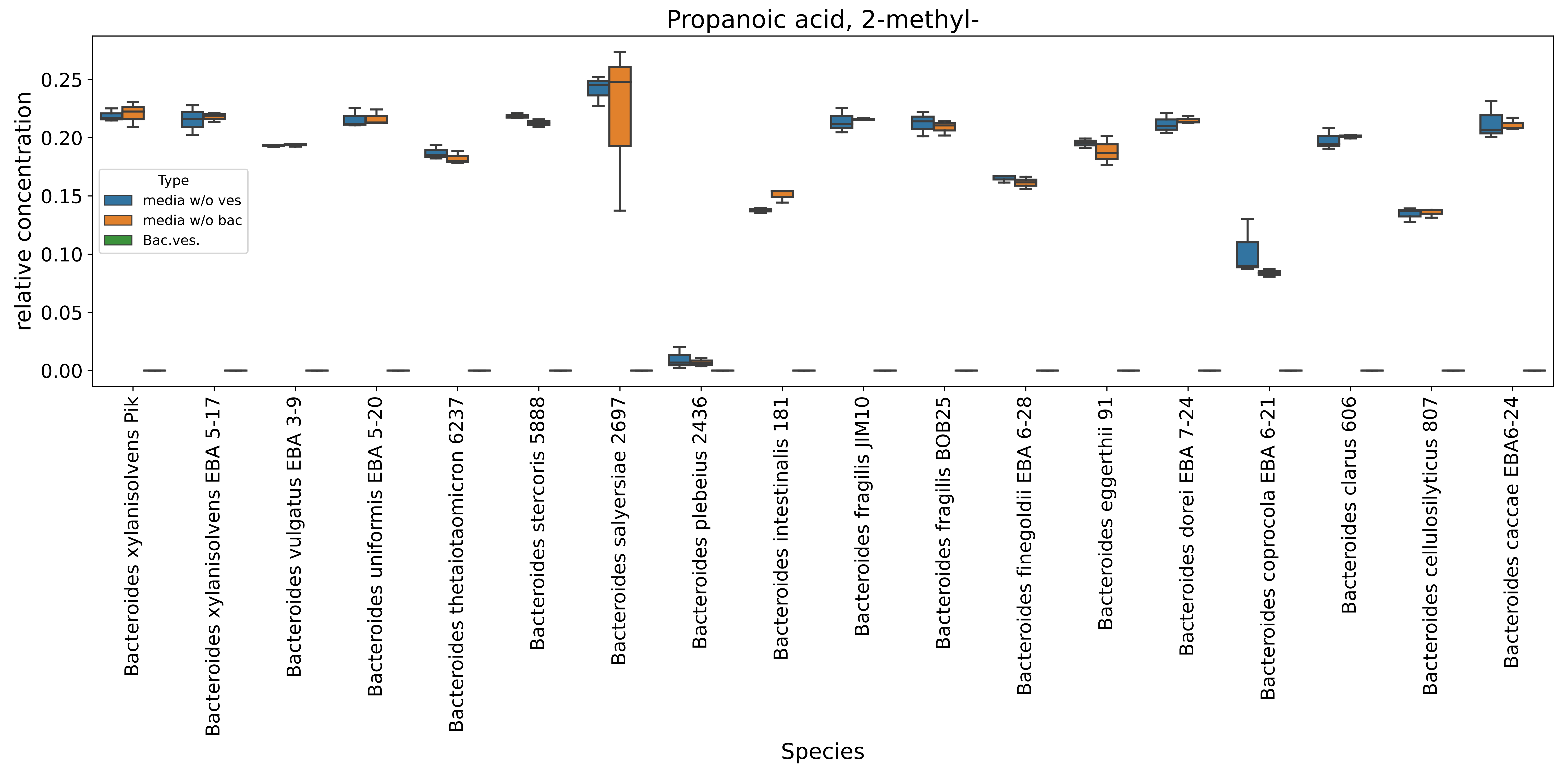

Propanoic acid, 2-methyl-
relative concentration
0.25
0.20
0.15
0.10
0.05
0.00
Type
media w/o ves
media w/o bac
Bac.ves.
Bacteroides xylanisolvens Pik
Bacteroides xylanisolvens EBA 5-17
Bacteroides vulgatus EBA 3-9
Bacteroides uniformis EBA 5-20
Bacteroides thetaiotaomicron 6237
Bacteroides stercoris 5888
Bacteroides salyersiae 2697
Bacteroides plebeius 2436
Bacteroides intestinalis 181
Bacteroides fragilis JIM10
Bacteroides fragilis BOB25
Bacteroides finegoldii EBA 6-28
Bacteroides eggerthii 91
Bacteroides dorei EBA 7-24
Bacteroides coprocola EBA 6-21
Bacteroides clarus 606
Bacteroides cellulosilyticus 807
Bacteroides caccae EBA6-24
Species

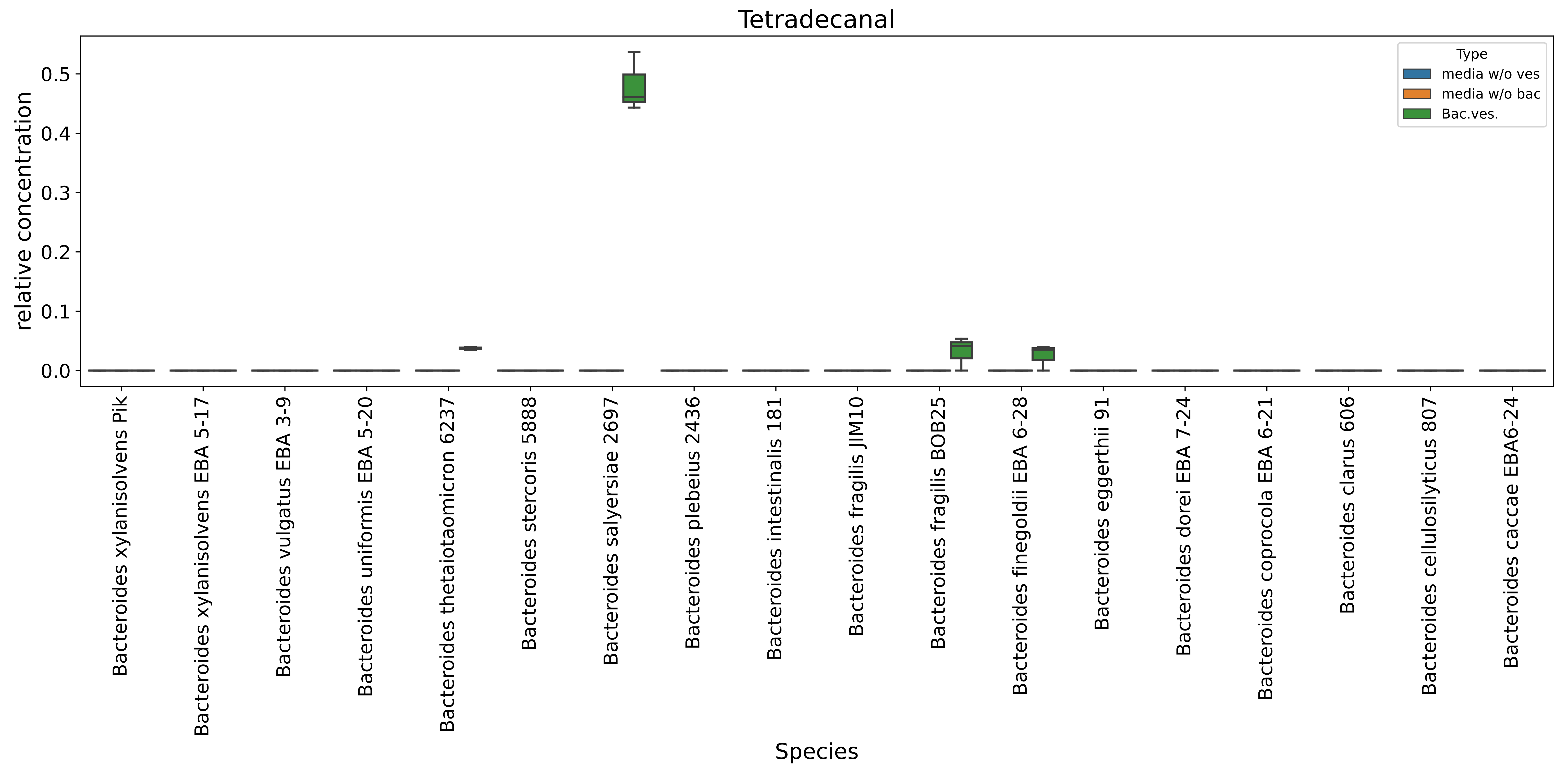
Tetradecanal
relative concentration
0.0
0.1
0.2
0.3
0.4
0.5
Type
media w/o ves
media w/o bac
Bac.ves.
Bacteroides xylanisolvens Pik
Bacteroides xylanisolvens EBA 5-17
Bacteroides vulgatus EBA 3-9
Bacteroides uniformis EBA 5-20
Bacteroides thetaiotaomicron 6237
Bacteroides stercoris 5888
Bacteroides salyersiae 2697
Bacteroides plebeius 2436
Bacteroides intestinalis 181
Bacteroides fragilis JIM10
Bacteroides fragilis BOB25
Bacteroides finegoldii EBA 6-28
Bacteroides eggerthii 91
Bacteroides dorei EBA 7-24
Bacteroides coprocola EBA 6-21
Bacteroides clarus 606
Bacteroides cellulosilyticus 807
Bacteroides caccae EBA6-24
Species

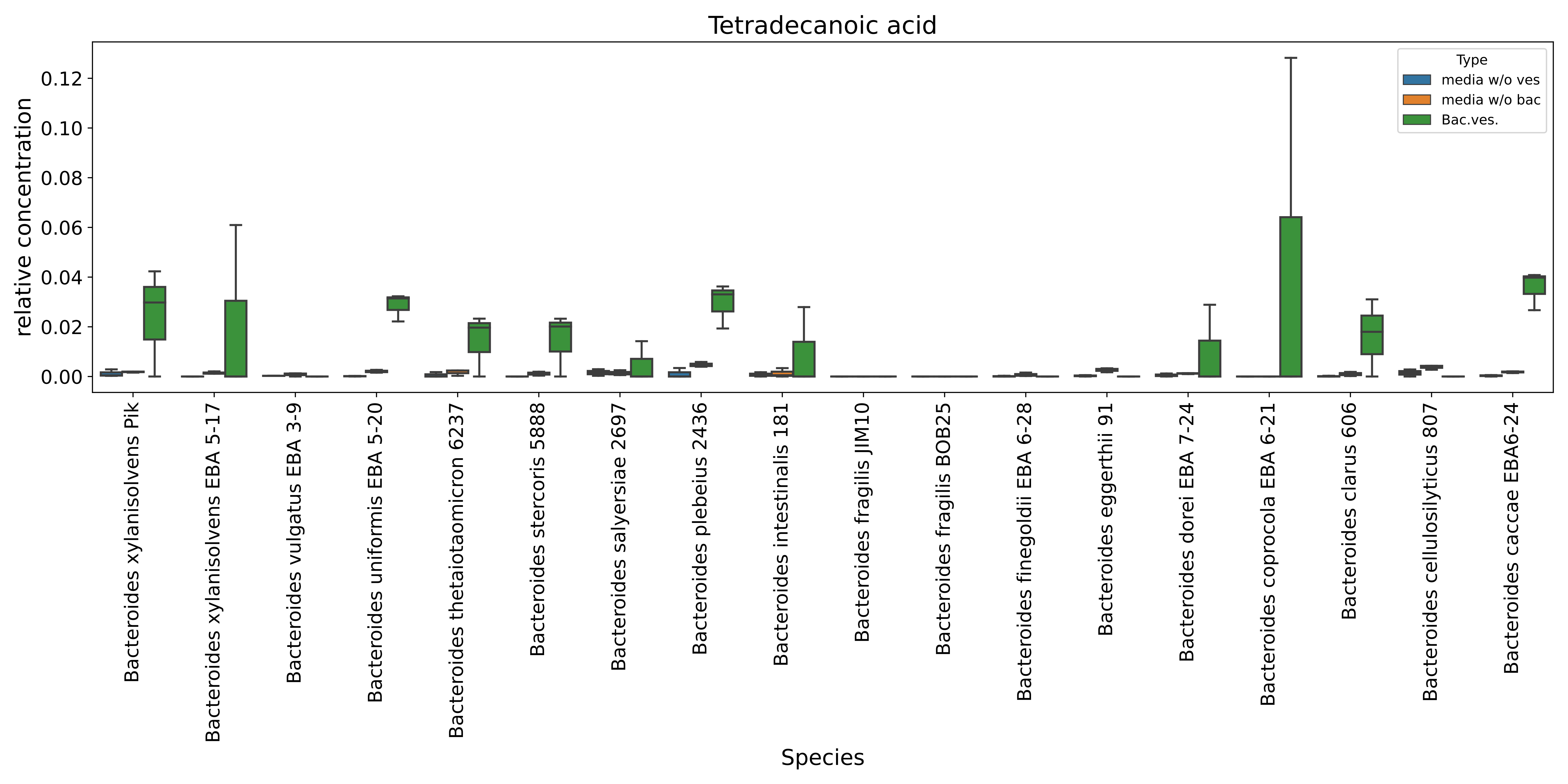
Tetradecanoic acid
relative concentration
0.12
0.10
0.08
0.06
0.04
0.02
0.00
Type
media w/o ves
media w/o bac
Bac.ves.
Bacteroides xylanisolvens Pik
Bacteroides xylanisolvens EBA 5-17
Bacteroides vulgatus EBA 3-9
Bacteroides uniformis EBA 5-20
Bacteroides thetaiotaomicron 6237
Bacteroides stercoris 5888
Bacteroides salyersiae 2697
Bacteroides plebeius 2436
Bacteroides intestinalis 181
Bacteroides fragilis JIM10
Bacteroides fragilis BOB25
Bacteroides finegoldii EBA 6-28
Bacteroides eggerthii 91
Bacteroides dorei EBA 7-24
Bacteroides coprocola EBA 6-21
Bacteroides clarus 606
Bacteroides cellulosilyticus 807
Bacteroides caccae EBA6-24
Species

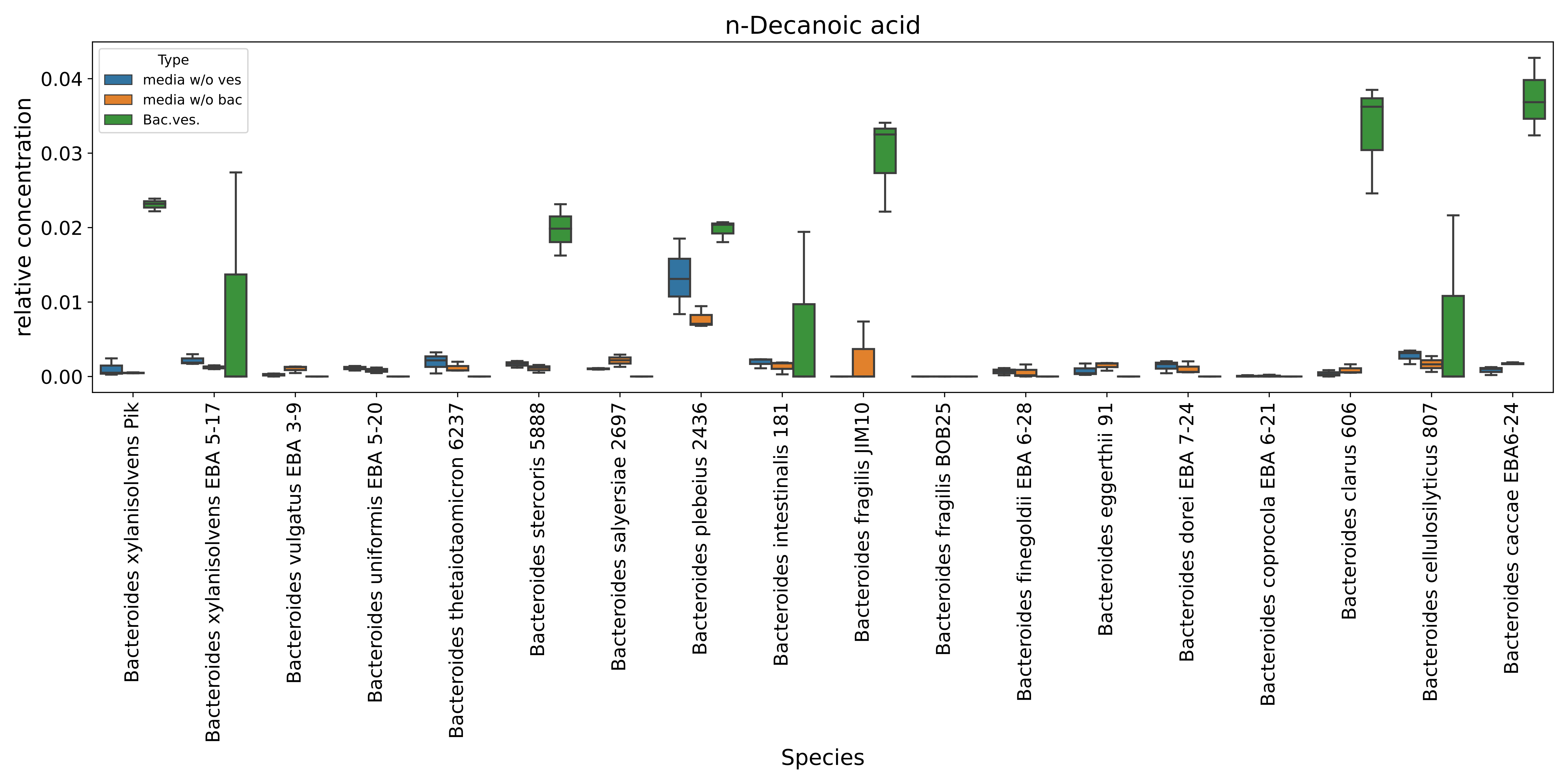

n-Decanoic acid
Type
media w/o ves
media w/o bac
Bac.ves.
relative concentration
0.04
0.03
0.02
0.01
0.00
Bacteroides xylanisolvens Pik
Bacteroides xylanisolvens EBA 5-17
Bacteroides vulgatus EBA 3-9
Bacteroides uniformis EBA 5-20
Bacteroides thetaiotaomicron 6237
Bacteroides stercoris 5888
Bacteroides salyersiae 2697
Bacteroides plebeius 2436
Bacteroides intestinalis 181
Bacteroides fragilis JIM10
Bacteroides fragilis BOB25
Bacteroides finegoldii EBA 6-28
Bacteroides eggerthii 91
Bacteroides dorei EBA 7-24
Bacteroides coprocola EBA 6-21
Bacteroides clarus 606
Bacteroides cellulosilyticus 807
Bacteroides caccae EBA6-24
Species

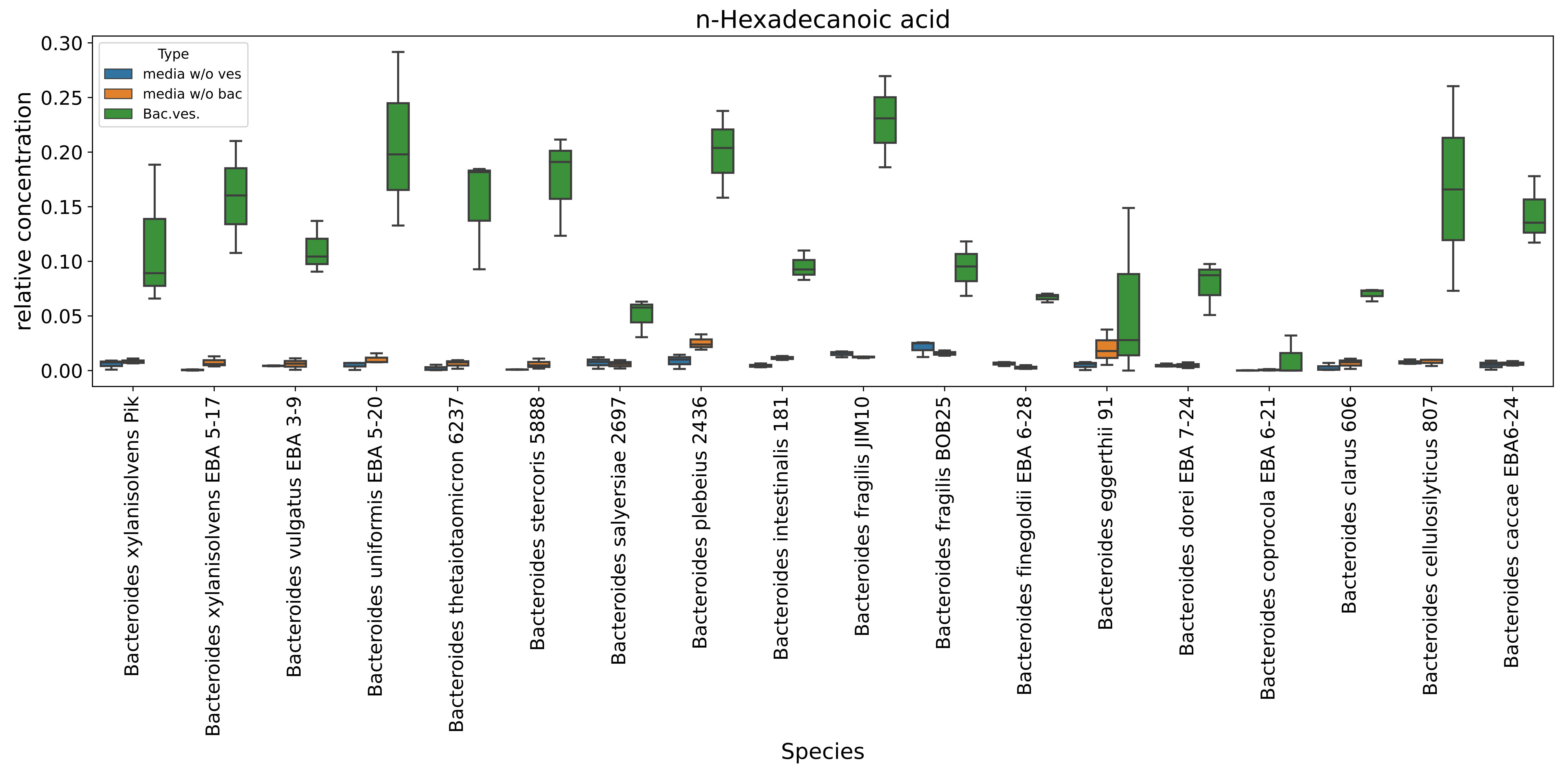

n-Hexadecanoic acid
Type
media w/o ves
media w/o bac
Bac.ves.
relative concentration
0.00
0.05
0.10
0.15
0.20
0.25
0.30
Bacteroides xylanisolvens Pik
Bacteroides xylanisolvens EBA 5-17
Bacteroides vulgatus EBA 3-9
Bacteroides uniformis EBA 5-20
Bacteroides thetaiotaomicron 6237
Bacteroides stercoris 5888
Bacteroides salyersiae 2697
Bacteroides plebeius 2436
Bacteroides intestinalis 181
Bacteroides fragilis JIM10
Bacteroides fragilis BOB25
Bacteroides finegoldii EBA 6-28
Bacteroides eggerthii 91
Bacteroides dorei EBA 7-24
Bacteroides coprocola EBA 6-21
Bacteroides clarus 606
Bacteroides cellulosilyticus 807
Bacteroides caccae EBA6-24
Species

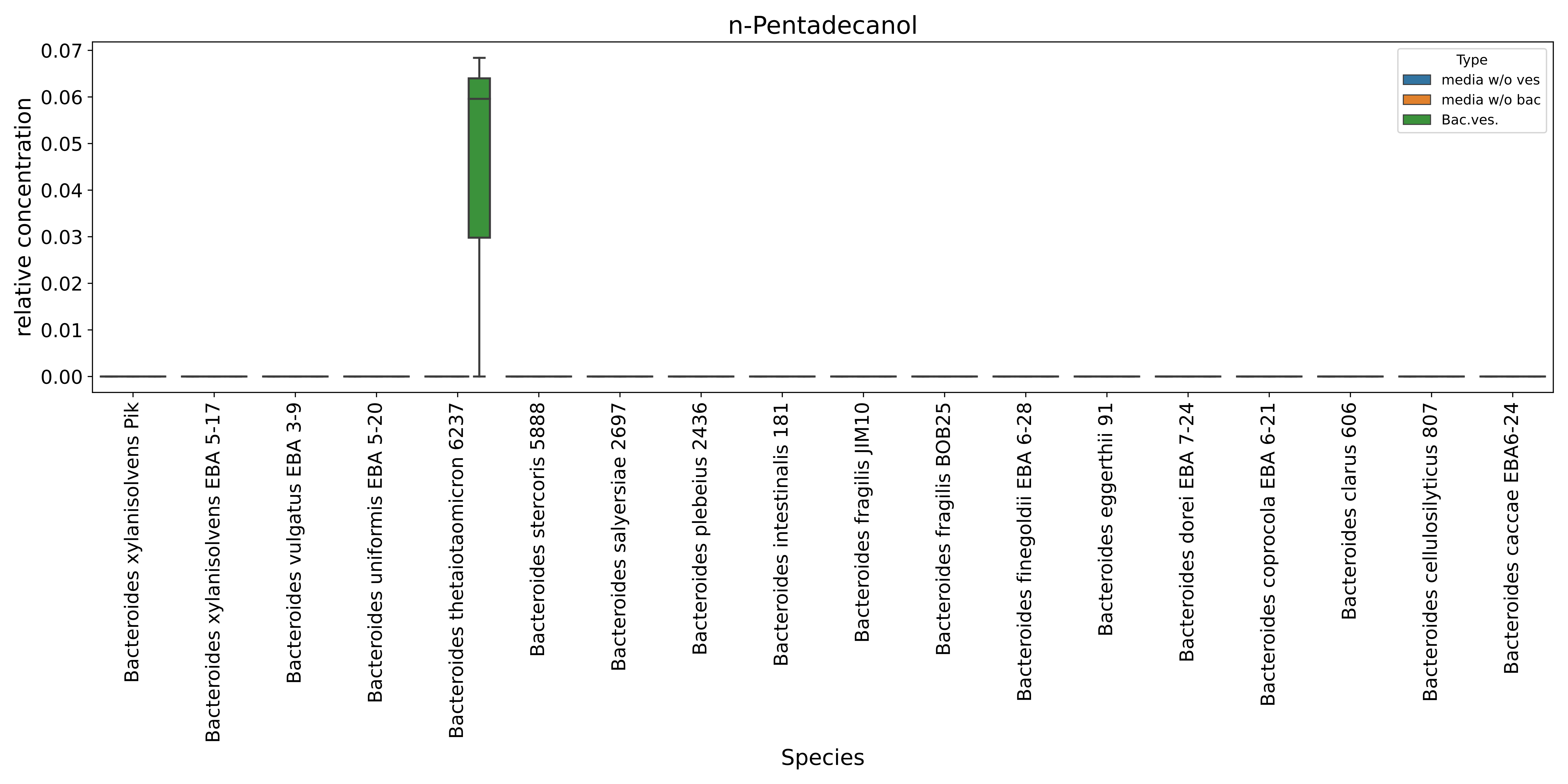

n-Pentadecanol
relative concentration
0.07
0.06
0.05
0.04
0.03
0.02
0.01
0.00
Type
media w/o ves
media w/o bac
Bac.ves.
Bacteroides xylanisolvens Pik
Bacteroides xylanisolvens EBA 5-17
Bacteroides vulgatus EBA 3-9
Bacteroides uniformis EBA 5-20
Bacteroides thetaiotaomicron 6237
Bacteroides stercoris 5888
Bacteroides salyersiae 2697
Bacteroides plebeius 2436
Bacteroides intestinalis 181
Bacteroides fragilis JIM10
Bacteroides fragilis BOB25
Bacteroides finegoldii EBA 6-28
Bacteroides eggerthii 91
Bacteroides dorei EBA 7-24
Bacteroides coprocola EBA 6-21
Bacteroides clarus 606
Bacteroides cellulosilyticus 807
Bacteroides caccae EBA6-24
Species

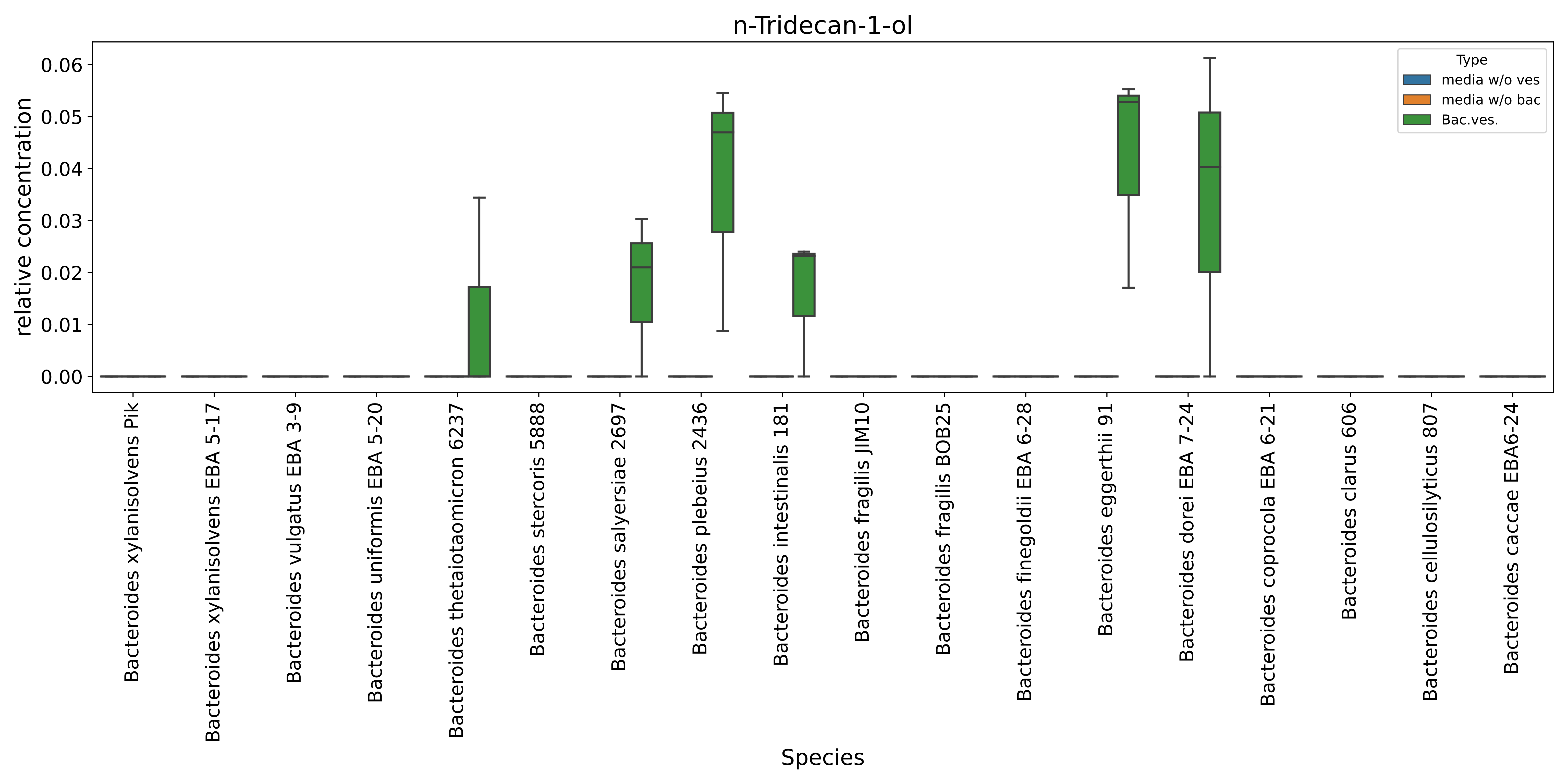
n-Tridecan-1-ol
relative concentration
0.06
0.05
0.04
0.03
0.02
0.01
0.00
Type
media w/o ves
media w/o bac
Bac.ves.
Bacteroides xylanisolvens Pik
Bacteroides xylanisolvens EBA 5-17
Bacteroides vulgatus EBA 3-9
Bacteroides uniformis EBA 5-20
Bacteroides thetaiotaomicron 6237
Bacteroides stercoris 5888
Bacteroides salyersiae 2697
Bacteroides plebeius 2436
Bacteroides intestinalis 181
Bacteroides fragilis JIM10
Bacteroides fragilis BOB25
Bacteroides finegoldii EBA 6-28
Bacteroides eggerthii 91
Bacteroides dorei EBA 7-24
Bacteroides coprocola EBA 6-21
Bacteroides clarus 606
Bacteroides cellulosilyticus 807
Bacteroides caccae EBA6-24
Species

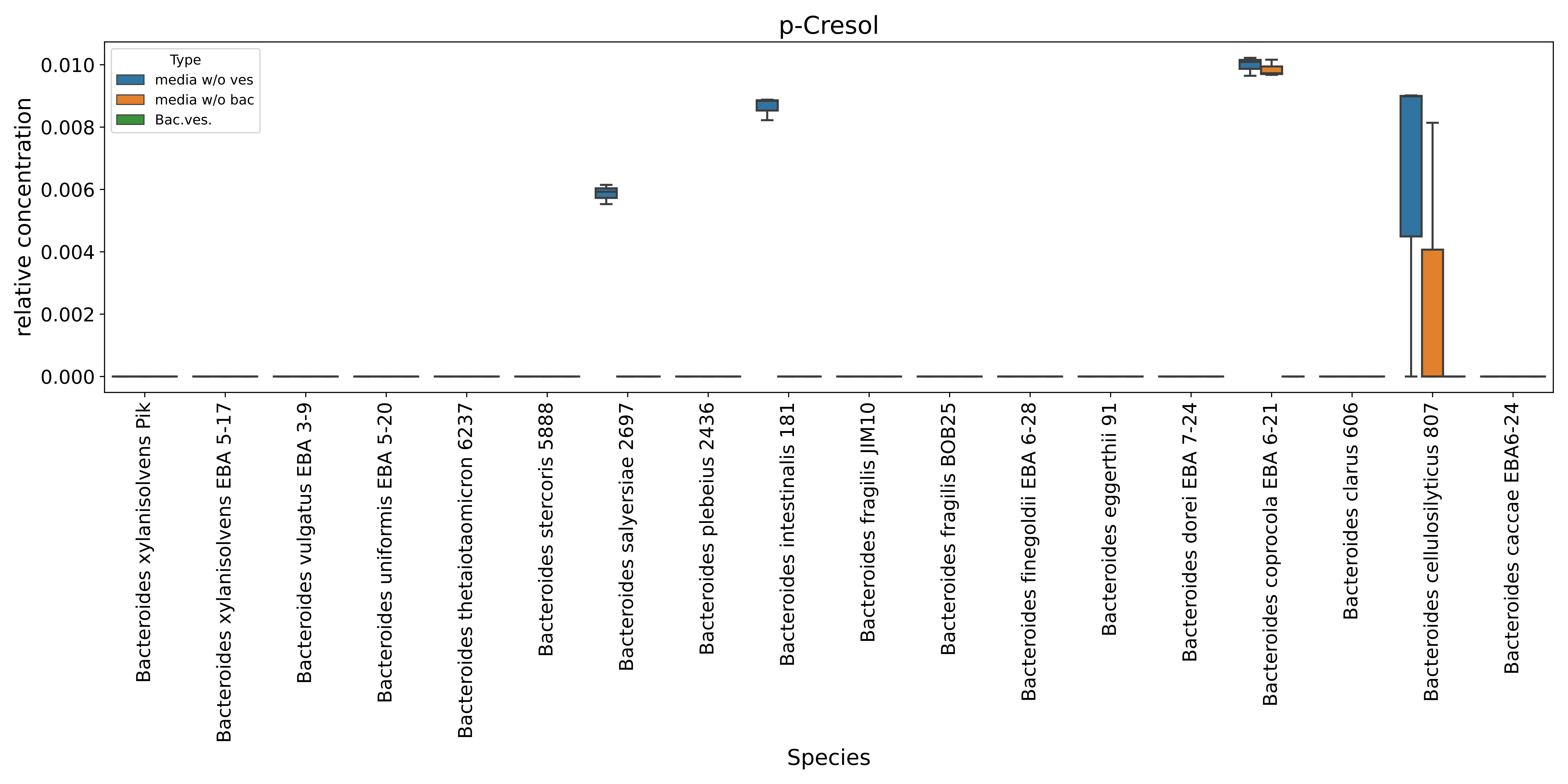

p-Cresol
Type
media w/o ves
media w/o bac
Bac.ves.
relative concentration
0.010
0.008
0.006
0.004
0.002
0.000
Bacteroides xylanisolvens Pik
Bacteroides xylanisolvens EBA 5-17
Bacteroides vulgatus EBA 3-9
Bacteroides uniformis EBA 5-20
Bacteroides thetaiotaomicron 6237
Bacteroides stercoris 5888
Bacteroides salyersiae 2697
Bacteroides plebeius 2436
Bacteroides intestinalis 181
Bacteroides fragilis JIM10
Bacteroides fragilis BOB25
Bacteroides finegoldii EBA 6-28
Bacteroides eggerthii 91
Bacteroides dorei EBA 7-24
Bacteroides coprocola EBA 6-21
Bacteroides clarus 606
Bacteroides cellulosilyticus 807
Bacteroides caccae EBA6-24
Species

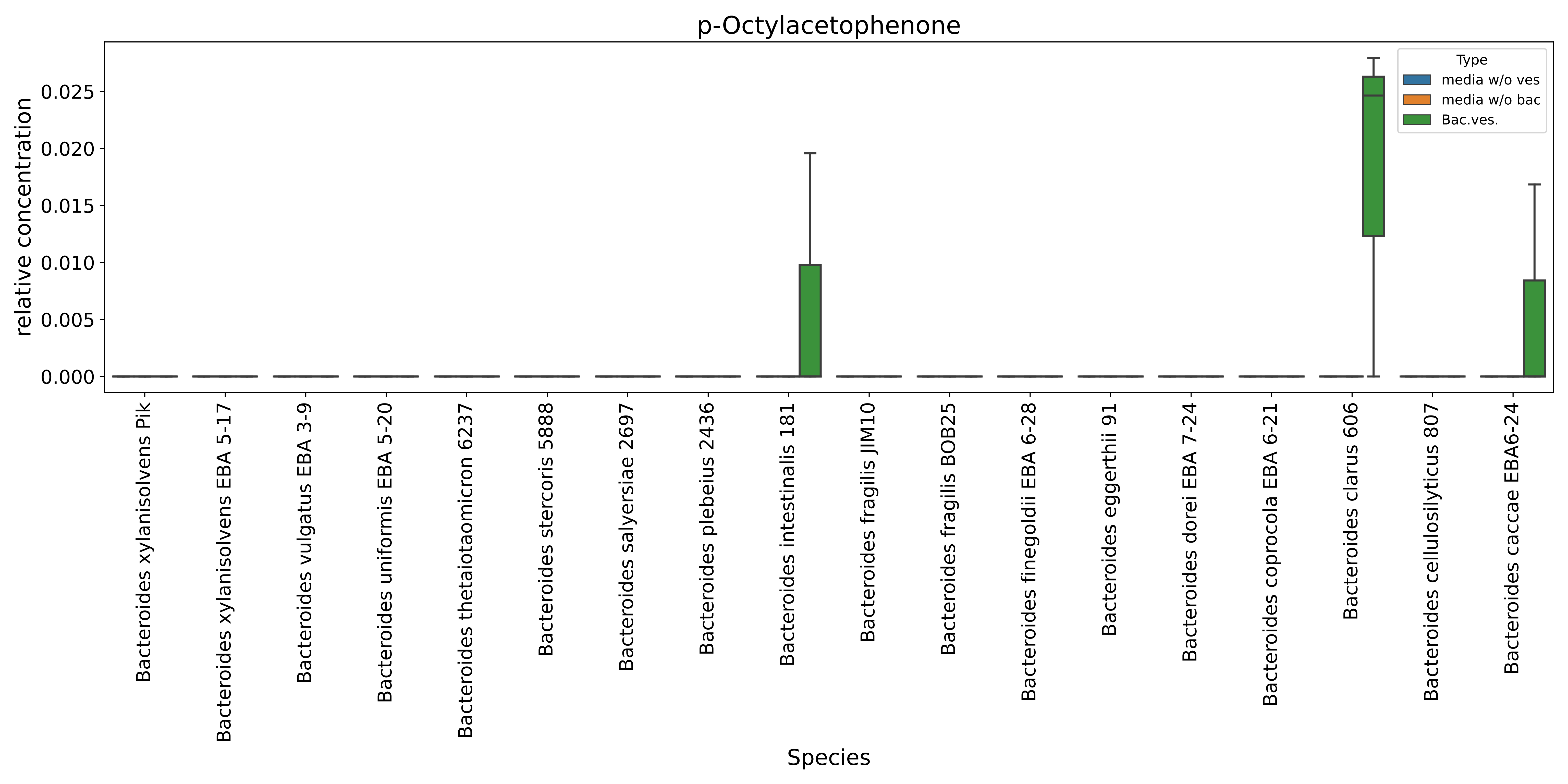

p-Octylacetophenone
relative concentration
0.025
0.020
0.015
0.010
0.005
0.000
Type
media w/o ves
media w/o bac
Bac.ves.
Bacteroides xylanisolvens Pik
Bacteroides xylanisolvens EBA 5-17
Bacteroides vulgatus EBA 3-9
Bacteroides uniformis EBA 5-20
Bacteroides thetaiotaomicron 6237
Bacteroides stercoris 5888
Bacteroides salyersiae 2697
Bacteroides plebeius 2436
Bacteroides intestinalis 181
Bacteroides fragilis JIM10
Bacteroides fragilis BOB25
Bacteroides finegoldii EBA 6-28
Bacteroides eggerthii 91
Bacteroides dorei EBA 7-24
Bacteroides coprocola EBA 6-21
Bacteroides clarus 606
Bacteroides cellulosilyticus 807
Bacteroides caccae EBA6-24
Species
