# Supplementary figures and images for: Investigating volatile compounds in the Bacteroides secretome
Source: Front Microbiol. 2023 May 3;14:1164877. doi: 10.3389/fmicb.2023.1164877 (PMC10189065; doi:10.3389/fmicb.2023.1164877)

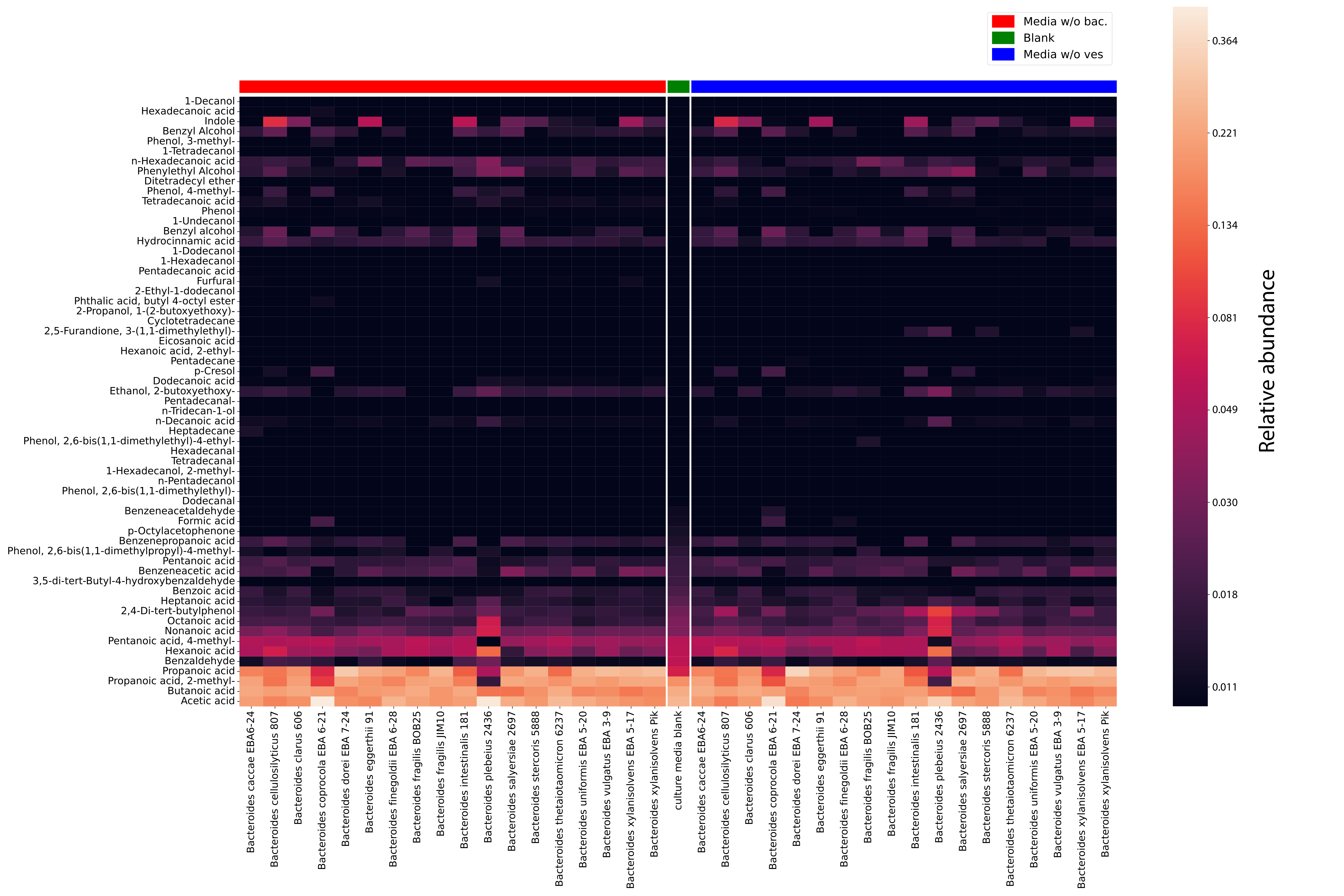

Supplement: Supplementary file 5 [file Image_1.TIFF]
